# Age of acquisition impacts the brain differently depending on neuroanatomical metric

**DOI:** 10.1002/hbm.24817

**Published:** 2019-10-10

**Authors:** Hannah Claussenius‐Kalman, Kelly A. Vaughn, Pilar Archila‐Suerte, Arturo E. Hernandez

**Affiliations:** ^1^ Department of Psychology University of Houston Houston Texas

**Keywords:** age of acquisition, bilingualism, gray matter, language development, magnetic resonance imaging, neuroanatomy, replication crisis

## Abstract

Although researchers generally agree that a certain set of brain areas underlie bilingual language processing, there is discrepancy regarding what effect timing of language acquisition has on these regions. We aimed to investigate the neuroanatomical correlates of age of acquisition (AoA), which has been examined previously, but with inconsistent results, likely influenced by methodological differences across studies. We analyzed gray matter density, volume, and thickness using whole‐brain linear models in 334 bilinguals and monolinguals. Neuroanatomical correlates of AoA differed depending on gray matter metric. Relative to early bilinguals, late bilinguals had thicker cortex in language processing and cognitive control regions, and greater density in multiple frontal areas and the right middle temporal and supramarginal gyri. Early bilinguals had greater volume than late bilinguals in the left middle temporal gyrus. Overall, volume was the least sensitive to AoA‐related differences. Multiple regions not classically implicated in dual‐language processing were also found, which highlights the important role of whole‐brain analyses in neuroscience. This is the first study to investigate AoA and gray matter thickness, volume, and density all in the same sample. We conclude that cognitive models of bilingualism should consider the roles of development and neuroanatomical metric in driving our understanding of bilingual and monolingual language organization.

## 1 INTRODUCTION

Although researchers generally agree that a certain set of brain areas are involved in bilingual language processing, it is unclear what effect timing of language acquisition has on these regions. Twelve peer‐reviewed studies have examined the relationship between brain structure and age of acquisition (AoA), and results of these studies are conflicting. We suggest that there are three main reasons for differing conclusions across previous studies (see Table 1 for summary): different measures of brain structure (four measure white matter, two measure GM thickness, three measure GM volume, and three measure GM density), different parameterizations of AoA (as a continuous vs. categorical variable with bins that vary across studies), and small group sizes (between *n* = 12 and *n* = 39), all of which increase variability in results. The present study aimed to understand the neuroanatomical correlates of AoA and increase comparability to previous studies through the use of multiple measures of gray matter (GM) structure and larger group sizes.

**Table 1:** Studies on structural correlates of age of acquisition (AoA)

Study	Measurement	Groups	Association with AoA
Abutalebi, Canini, Della Rosa, Green, and Weekes (2015)	Volume (GM)	30 older Bi 30 older Mo	No AoA effect
Berken, Gracco, Chen, and Klein (2016b)	Density (GM)	16 Bi_Sim_ 18 Bi_Seq_	Bi_Sim_ > Bi_Seq_: Putamen, PFC, insula, occipital Bi_Seq_ > Bi_Sim_: Bilateral premotor
Felton et al. (2017)	Cortical thickness (GM)	39 Mo 39 Bi	No AoA effect
Grogan et al. (2012)	Density (GM)	30 Bi 31 Multi	− L pars opercularis
Kaiser et al. (2015)	Volume (GM)	24 Bi_Sim_ 20 Bi_Seq_	Bi_Sim_ < Bi_Seq_: L ITG, L IFG, R IFG, R MTG, R IPPG, Bilateral MFG
Klein, Mok, Chen, and Watkins (2014)	Cortical thickness (GM)	22 Mo 12 Bi_Sim_ 25 Bi_Early_ 29 Bi_Late_	Bi_Late_ > Mo, Bi_Early_ > Mo: L IFG Bi_Late_ < Mo, Bi_Early_ < Mo Bi_Late_ < Bi_Early,_ Bi_Late_ < Bi_Sim_: R IFG + L IFG, L SPL − R IFG
Mechelli et al. (2004)	Density (GM)	22 Bi	− L IPL
Mohades et al. (2012)	DTI (WM)	10 Mo 15 Bi_Sim_ 15 Bi_Seq_	Bi_Sim_ > Bi_Seq_, Bi_Sim_ > Mo: L IFOF tracts Bi_Sim_ < Mo: AC‐OL fibers
Nichols and Joanisse (2016)	DTI (WM)	23 Bi	+ L corpus callosum, L arcuate fasciculus, L and R ILF
Pliatsikas, DeLuca, Moschopoulou, and Saddy (2017)	VBM (WM)	25 Mo 20 Bi	No AoA effect
Rossi, Cheng, Kroll, Diaz, and Newman (2017)	DTI (WM)	24 Mo 25 Bi	− Anterior–posterior corona radiata
Wei et al. (2015)	Volume and area (GM)	36 Bi	+ R pars orbitalis − R SPL

*Note*: Parameters for inclusion: studies had to examine structural correlates of AoA within bilinguals. For instance, a study that compares only simultaneous bilinguals to monolinguals would not be included because AoA effects cannot be explained; However, a study that examines structural effects of AoA within bilinguals would be included. Measures: DTI, diffusion tensor imaging; FA, fractional anisotropy; GM, gray matter; VBM, voxel‐based morphometry; WM, White matter. Groups: Mo, monolingual; Bi, bilingual; Multi, multilingual. Bi_Sim_, simultaneous bilingual (acquired L1 and L2 from birth). Bi_Seq_, sequential bilingual (acquired L2 after L1). Bi_Early_, early bilingual (AoA 4–7 years). Bi_Late_, late bilingual (AoA >7 years). Results: + indicates that a region increased as AoA increased; − indicates that a region decreased as AoA increased. < and > indicate which group had greater density/volume/thickness in a region. AC‐OL, anterior part of corpus callosum projecting to orbital lobe; IFG, inferior frontal gyrus; IFOF, inferior occipitofrontal fasciculus; ILF, inferior longitudinal fasciculus; IPL, inferior parietal lobule; IPPG, inferior posterior parietal gyrus; ITG, inferior temporal gyrus. L, left hemisphere; MFG, middle frontal gyrus; MTG, middle temporal gyrus; PFC, prefrontal cortex; R, right hemisphere; SPL, superior parietal lobule.

It has been well‐established that brain structure reflects the accumulation of genetic influences and life‐long experiences (Alexander‐Bloch, Giedd, & Bullmore, 2013). Dual language processing is one skill that should have especially far‐reaching effects on the brain because it draws on many domains, including motor skills, emotional processing, and higher‐level thinking (Friederici, 2011). This idea has been demonstrated through neuroimaging research, which shows that speaking two languages is related to regions involved in language processing as well as cognitive control regions (Price, 2010) and is reflected in structural changes to these regions (García‐Pentón, García, Costello, Duñabeitia, & Carreiras, 2016; Olulade et al., 2016).

Given that there is a sensitive period for processing of sensations such as vision and sound (Brainard & Knudsen, 1998; Harrison, Gordon, & Mount, 2005), it follows that the neural substrates recruited to support L2 acquisition may differ depending on the developmental stage of the individual (Hartshorne, Tenenbaum, & Pinker, 2018). For instance, native‐like accent production, phonemic perception, and implicit knowledge of syntax are thought to have an early sensitive period (i.e., before age 7; Johnson & Newport, 1989; Tokowicz & MacWhinney, 2005) due to higher plasticity of certain brain structures (e.g., motor/speech pathways) early in development (Hernandez & Li, 2007). Therefore, it is likely that neuroplasticity manifests differently in relation to age of L2 acquisition (Berken, Gracco, & Klein, 2017), but its exact manifestation differs between studies. Some studies (e.g., Grogan et al., 2012) suggest that the plasticity of certain structures facilitates high language proficiency in bilinguals who learned the L2 early in development. One piece of evidence for this idea comes from a study by Mohades et al. (2012), who found that simultaneous bilinguals (i.e., those who acquired the first and second language before age 3) had higher fractional anisotropy in the left inferior frontal‐occipital fasciculus compared to sequential bilinguals and monolinguals. They suggested that this enhanced connectivity permits faster transmission of semantic information. Additionally, Mechelli et al. (2004) found that bilinguals had increased GM density in the left inferior parietal lobule (IPL) compared to monolinguals, and that those with the earliest AoA had the highest density. Finally, Grogan et al. (2012) showed that simultaneous bilinguals have greater adaptations to cortical structure than monolinguals and other bilinguals. Specifically, earlier AoA and greater lexical efficiency were both related to greater GM density in the left pars opercularis, possibly due to increased demand for phonological processing (see Table 1 for summary of all structural studies that examine AoA).

On the other hand, some studies suggest that structural changes are more likely to occur in late bilinguals. Berken et al. (2017) suggest that bilinguals with an AoA that is later than the optimal period for language (i.e., nonsimultaneous bilinguals) depend on compensatory processes beyond the activity patterns for a single L1. Based on the sensorimotor hypothesis (Hernandez & Li, 2007), one could surmise that the later an individual begins learning a second language, the more likely it is that structures involved in higher‐level cognition (e.g., dorsolateral prefrontal cortex [DLPFC] and parietal regions) will be recruited to support the L2, as opposed to sensorimotor areas such as the basal ganglia. For instance, Berken et al. (2016b) found that GM density was greater in simultaneous compared to sequential bilinguals in areas related to articulation, such as the left putamen, prefrontal, insular, and occipital cortices, whereas sequential bilinguals had greater GM density in the bilateral premotor cortex. Furthermore, the sequential bilinguals with more native‐like accent showed greater GM density in the left premotor cortex, left inferior frontal gyrus (IFG), right primary motor cortex, right Heschl's gyrus, right lateral occipital cortex (LOC), right cerebellar vermis, and bilateral IPL. The authors interpreted these results to mean that sequential bilinguals recruit different neural substrates to achieve proficiency than simultaneous bilinguals. This is in line with functional neuroimaging research showing that, whereas simultaneous bilinguals demonstrate stronger resting‐state functional connectivity between the bilateral IFG and language control areas (i.e., DLPFC, IPL, and cerebellum), sequential bilinguals show stronger functional connectivity between the left IFG, right IFG, and right IPL (Berken, Chai, Chen, Gracco, & Klein, 2016aa).

Given these mixed hypotheses and results, it is necessary to consider how exactly previous studies operationalize brain structure. Cortical thickness is the measure of the distance between the white matter surface and pial surface. One advantage of this measure is that it is not confounded by head size (unlike volume, which is influenced by surface area; Panizzon et al., 2009). Although the term volume is often used interchangeably with density, these metrics are distinct, with the main difference being that density can be calculated using normalized unmodulated data, whereas volume and CT are calculated using modulated data (Ashburner & Friston, 2000). Modulation accounts for the changes in brain volume that occurred as a result of spatial normalization (Good et al., 2001). Normalization requires the original brain volume to morph (some areas expand and others compress). To account for these changes the voxel intensities are multiplied by a Jacobian determinant. Analyzing unmodulated data yields information about concentration (i.e., density) differences per unit volume in native space; analyzing modulated data yields information about the absolute amount of GM (i.e., volume) (Ashburner & Friston, 2000). The key point here is that MRI‐derived structural metrics each reflect unique aspects of experience‐dependent plasticity; However, research is still in the process of illuminating how each metric uniquely reflects function, so it is important to consider that a neuroanatomical profile of any demographic variable is likely best explained by examining multiple metrics.

On top of methodological differences, high variability across findings is also likely influenced by small sample sizes, which is a common issue in neuroimaging studies because of the highly exclusive participation eligibility and high time/cost commitments. The research published thus far has advanced our understanding of bilingualism. At the same time, if power is low there is a decreased ability to detect population effects, which “also reduces the likelihood that a statistically significant result reflects a true effect” (Button et al., 2013, p. 365). If it is a true effect, this can result in an exaggerated estimate of the effect's magnitude (Kellmeyer, 2017). One example of this problem comes from a study by Cremers, Wager, and Yarkoni (2017), who demonstrated that inference in functional MRI is disproportionately influenced by small sample sizes. Specifically, the authors took 10,000 horizontal brain slices (i.e., one slice from each of 10,000 subjects) and drew 2,000 random subsamples that ranged between 10 and 150 slices. They found that sample sizes below 30 displayed disproportionately low statistical power, did a poor job of representing true effects that existed in the full sample, and were highly inconsistent in their ability to replicate. This may be one reason for varying results across previous studies, in which the sample size per group was between *n* = 12 and *n* = 39 participants (see Table 1).

## 2 THE PRESENT STUDY

The aim of the present study was to investigate the relationship between AoA and structural GM, as well as increase clarity across previous studies. This objective was accomplished by: comparing cortical thickness, volume, and density in the same sample; using both categorical and continuous methods to measure AoA; and doing so in a larger‐than‐average sample of bilinguals and monolinguals. Based on the bilingual literature at large, we hypothesized that both continuous (regression) and categorical (ANOVA using orthogonal contrasts) analyses would demonstrate that as AoA increases, so does GM volume, thickness, and density, and that these changes would occur specifically in regions related to language processing (Price, 2010) and cognitive control (Abutalebi & Green, 2007; Green & Abutalebi, 2013). Evidence in favor of this hypothesis would support the idea that late L2 acquisition is an effortful process that requires top‐down processing. Although a priori hypothesis are not necessary for whole‐brain analyses, we expected that these changes would be seen in the following regions of the cortex: those implicated in cognitive control (DLPFC), those implicated in language processing (insula; inferior temporal gyrus, ITG; middle temporal gyrus, MTG; superior temporal gyrus, STG), and those thought to be involved in both cognitive control and language processing (anterior cingulate cortex, ACC; IPL, IFG; Green & Abutalebi, 2013; Price, 2010).

## 3 METHOD

### 3.1 Participants

This study analyzed magnetic resonance imaging (MRI) scans from eight studies conducted by the Laboratory for the Neural Bases of Bilingualism at University of Houston from 2007 to 2014, each of which included Spanish‐English bilingual and English‐speaking monolingual participants. Fifteen brain scans were removed from analyses (six brain scans were removed due to reconstruction issues, five due to technical MRI scanning issues, three due to not reporting AoA, and one due to brain trauma). This resulted in a final group of 334 brain scans (189 bilingual, 145 monolingual).

Inclusion criteria was based on participants reporting that they were right‐handed, had normal or corrected‐to‐normal vision, and had no history of neurological disorders. Participants were excluded if they were unable to enter the MRI safely (e.g., due to a metal or electronic medical implant or claustrophobia). Participants were males and females living in Houston, Texas (71.34% women and 28.66% men). Ages ranged between 18 and 45 years (*M =* 23.37, *SD =* 4.83; see Table 2 for descriptive statistics).

**Table 2:** Descriptive statistics of bilingual and monolingual participants

	Monolingual	Early bilingual (AoA 4–7 years)	Late bilingual (AoA > 7 years)	All bilingual
Sample size	145	121	68	189
Age mean (*SD*)	23.31 (4.94)	22.09 (3.65)	25.75 (5.56)	23.41 (4.75)
Age range in years	18–45	18–38	19–40	18–40
% women	65.51%	77.57%	60.29%	71.35%
Mean AoA (*SD*)	N/A	5.19 (0.97)	13.84 (5.00)	8.00 (5.19)
Mean E prof % correct (*SD*)	81.37 (7.50)	73.82 (7.65)	68.43 (10.32)	71.11 (9.08)
Mean S prof % correct (*SD*)	N/A	70.02 (10.60)	76.61 (8.77)	71.42 (11.10)
Mean % daily E use (*SD*)^a^	100 (0)	68.71 (17.77)	62.97 (21.86)	66.36 (19.69)
Mean % daily S use (*SD*)^a^	0 (0)	30.01 (16.70)	38.97 (22.52)	33.67 (19.73)
Mean SES (*SD*)^b^	4.27 (.95)	2.49 (1.40)	2.73 (1.49)	2.59 (1.44)

a Based on self‐report.

b Socioeconomic status (SES) based on parental education history, scale of 1–6 (1 = no education, 6 = graduate degree). AoA, age of acquisition; E, English; S, Spanish; Prof, Proficiency.

Individuals were considered monolingual if they reported no knowledge of languages other than English and fewer than 2 years of foreign language classes. They were asked to self‐rate proficiency on a scale of 1–7 (1 = no knowledge, 7 = like a native) their knowledge of English and other languages in which they may have taken classes, and if they self‐rated themselves above a 2 on any language other than English, they were excluded from the study.

All bilinguals reported acquiring Spanish as a first language. Bilingual participants were excluded if they reported knowledge of additional languages beyond English and Spanish using the same parameters as monolinguals listed above. AoA ranged from 4 to 27 years. Out of 189 bilinguals, 88 were born in the United States, 74 reported their birthplace as being outside of the United States (Colombia, El Salvador, Mexico, Venezuela, Puerto Rico, Peru, Ecuador, and Honduras were reported), and 27 did not report birthplace. Bilingual and monolingual groups had approximately equivalent ages and gender ratios. Monolinguals had overall higher overall English proficiency and higher self‐reported socioeducational status (SES; see Table 2). However, a Pearson's *r* data analysis revealed that SES was not significantly correlated with AoA among bilinguals (*r* = .11, *p =* .16), and a Student's *t* test revealed that SES was not significantly different between early (*M* = 2.50, *SD* = 1.40) and late (*M* = 2.73, *SD* = 1.49) bilinguals; *t*(160) = −1.04, *p* = .3.

### 3.2 Materials and procedure

This study, which gathered and analyzed structural MRI data from eight individual studies, was approved by the University of Houston Institutional Review Board. All participants completed informed consent, background information questionnaires, language history questionnaires, and language proficiency assessments, as well as confirmation that the participant would be able to safely enter the MRI scanner, prior to beginning MRI scanning.

#### 3.2.1 Background information survey

Participants completed a paper questionnaire on background information that asked about age, gender, birthplace, bilingual/monolingual designation, and SES. SES was determined by coding parents' education status on a scale of 1–6 (1 = no education, 6 = graduate degree) and averaging the two parents' scores. When only one parent's education status was reported, that sole score was used to estimate SES.

#### 3.2.2 Language history questionnaire

Participants were asked to complete a language history questionnaire, which was used to ensure that they qualified as bilingual or monolingual. The language history questionnaire asked participants to self‐report age of L2 acquisition, daily language use of English and Spanish, proficiency level in each language (1 = poor, 7 = like a native) and knowledge of languages other than Spanish and English. A Student's *t* test revealed that percent of daily language use in English (as opposed to Spanish) was not significantly different between early (*M* = 68.71, *SD* = 17.70) and late bilinguals (*M* = 62.97, *SD* = 21.86; *t*[157] = 1.96, *p* = .07). Information for these parameters can be seen in Table 2, with the exception of knowledge of languages other than Spanish or English because bilinguals who had knowledge of other languages did not qualify for the study.

#### 3.2.3 English and Spanish proficiency measures

Monolinguals, all of whom spoke English as a first language, were asked to complete the Boston Naming Test (Kaplan, Goodglass, & Weintraub, 1983) and/or the following subtests of the *Woodcock‐Muñoz Language Survey—Revised*: picture vocabulary, followed by either passage comprehension or listening comprehension (for detailed explanation of each subtest see Woodcock, Muñoz‐Sandova, Ruef, & Alvarado, 2005). Spanish‐English bilinguals were asked to complete the above measures both in English and Spanish to ensure qualification as a bilingual participant. The Boston Naming Test and the picture vocabulary subtest are both measures of vocabulary that require naming a series of nouns represented as pictures. The passage comprehension subtest requires filling in a missing word in a series of sentences. The listening comprehension subtest requires listening to an incomplete sentence and filling in the blank. No verbal assessments of phonological production (i.e., nativelikeness of accent) were conducted, nor were speech samples collected. We calculated proficiency scores for each participant by determining the percent correct on each subtest, then averaging these scores. Because data were from eight MRI studies, an overall proficiency score was determined for each participant by averaging the proficiency measures that each respective study obtained. For instance, Study 1 (named “Cognitive Control”) used the picture vocabulary and passage comprehension measures, so proficiency for individuals in this study was calculated by averaging the percentage correct on these two scores (see Supporting Information for further details). Monolinguals had higher English proficiency scores than bilinguals (*t*[325] = 10.84, *p* < .0001). Early bilinguals had higher English proficiency (*t*[187] = 3.92, *p* = .0001) and Spanish proficiency (*t*[186] = 4.82, *p <* .0001) than late bilinguals. Proficiency was entered as a covariate when conducting regression analyses of AoA.

### 3.3 Design

Within the 12 previous studies that examined this relationship, no two sets of results are the same, which is influenced by the use of different neuroanatomical metrics and parameterizations of AoA across studies. For comparison purposes, we chose to focus on the eight studies that examine GM structure, reasoning that bilingualism is likely to have profound effects on cortical organization, and to permit evaluation of multiple metrics within one type of tissue. We analyzed three different metrics of GM structure and used multiple parameterizations of AoA in our sample in order to increase comparability to previous findings.

This study had a between‐subjects design in which participants were grouped as monolingual or bilingual, and bilingual participants were further separated according to AoA (early: AoA = 4–7 years; late: AoA > 7 years), following the parameters used by Klein et al. (2014). The bilingual sample included 121 early and 68 late bilinguals. Because treating AoA as a categorical variable does not necessarily reflect any concrete age cut‐off in development, a regression analysis was also conducted in which AoA was treated as a continuous variable. Examining AoA according to multiple sets of parameters increased the number of previous studies to which we could compare our results.

The dependent variable in this study was GM structure. Three commonly‐used metrics of GM include density, volume, and CT. Given that the previous studies on the relationship between AoA and brain structure each use different measures individually (see Table 1), this study is the first to include all three measures in the same sample of bilinguals and monolinguals. Each variable was treated separately to answer three research questions: What is the relationship between AoA (using all above‐mentioned grouping methods) and CT; What is the relationship between AoA and GM volume; and what is the relationship between AoA and GM density.

### 3.4 MRI acquisition

All MRI scans were collected at the Center for Advanced Magnetic Resonance Imaging at Baylor College of Medicine in Houston, Texas. Brain scans were acquired using a 3.0 Tesla Magnetom Trio (Siemens, Germany). T1‐weighted MPRAGE scans were collected using the following parameters for all eight studies: repetition time (TR), 1,200 ms; echo time (TE), 2.66 ms; flip angle (FA), 12°; voxel size, 0.479 × 0.479 × 1.0 mm; 192 slices.

### 3.5 MR image processing

#### 3.5.1 Image processing in Freesurfer

Both volume and CT analyses were conducted using Freesurfer, version 5.3.0 (available online at http://surfer.nmr.mgh.harvard.edu). Freesurfer is equipped with semi‐automatic preprocessing abilities. Brain images were preprocessed using cortical‐based geometry to determine CT and folding patterns. The program was then used to preprocess images for motion correction and skull‐stripping, and to complete the segmentation of cerebrospinal fluid, white matter, and GM. Surface‐based registration was used to align cortical folds with a brain template and then volumetric registration was used to reconstruct images. Following the reconstruction process, every MR image was visually inspected slice by slice in horizontal, coronal, and sagittal views using Freeview. Images that had errors were fixed manually, white matter and pial segmentation were reconstructed, and then were inspected once again. At that point, images were either edited again and reconstructed, or processed through a final reconstruction before analysis.

#### 3.5.2 Image processing in SPM

Density and volume analyses were conducted using Statistical Parametric Mapping software (SPM12; 6,906). Density was analyzed with general linear modeling in SPM12 using the Computational Anatomy Toolbox (CAT12). Following realignment, a normalization function was used to manipulate T1 images into Montreal Neurological Institute (MNI) space. Data were normalized to create tissue class images that correspond to the template space and interpret results about GM concentration (density). This step was used in place of modulated normalization in order to obtain density results. Volume results were obtained using the same steps as above, except that volume was calculated from modulated data. SPM12 uses unified segmentation to model segmentation of different tissue types, bias correction (for removing intensity differences within one image), and spatial normalization all in one step. It was ensured that all images followed the same orientation. Following preprocessing, a quality check was done via the CAT12 toolbox options to display one slice for all images and check sample homogeneity.

### 3.6 Statistical approach

Discrepancy in results from previous studies is likely driven by methodological differences. To increase clarity across previous studies we analyzed three neuroanatomical metrics in the same sample and used both categorical and continuous methods to measure AoA, all in a larger‐than‐average sample of bilinguals and monolinguals. Based on the previous studies outlined in Table 1, we conducted separate analyses that treated AoA as continuous (regression) and categorical (ANOVA/ANCOVA). We specifically chose to analyze group differences with ANOVA using orthogonal contrasts (and not test every possible *t*‐test combination) in order examine monolingual, early bilingual, and late bilingual effects using a straightforward and holistic approach. It was important to simplify the group analysis method as much as possible because the present paper examines a complex set of parameters (neuroanatomical metric, software, and AoA categorization method). This statistical choice has the benefit of partitioning the sum of squares so that they are nonoverlapping, thereby diminishing any losses in power that would occur from conducing multiple *t*‐tests for every possible combination between groups and/or tangling effects between bilinguals and monolinguals across some *t*‐tests and not others. We chose to include monolinguals in the ANOVA/ANCOVA to make the group‐level analyses more comparable to previous studies that examine AoA, and because it is typical in the bilingual literature to include monolinguals as a comparison group (e.g., Bennett & Verney, 2019; Pliatsikas et al., 2017). As outlined below, we conducted an ANOVA, an ANCOVA, and a regression for each of the three neuroanatomical metrics that we analyzed (thickness, density, volume).

#### 3.6.1 FreeSurfer—Surface‐based cortical thickness and volume

All analyses were conducted using whole brain analyses. A one‐way ANOVA with AoA group as the factor was conducted using FreeSurfer's mri_glmfit command. Additionally, a one‐way ANCOVA with proficiency included as a covariate was conducted using the same command. To interpret the results, orthogonal contrasts were created with monolingual, early bilingual (AoA = 4–7 years), and late bilingual (AoA > 7 years) groups. This led to the following contrasts: monolinguals versus bilinguals (i.e., early and late bilinguals), and early versus late bilinguals. The ANOVA and ANCOVA were computed for thickness and volume in each hemisphere. One characteristic of whole‐brain analyses is that many voxels will appear significant simply by chance due to the fact that thousands of voxels are being analyzed. Conventional multiple hypothesis testing (e.g., Bonferroni) is often not sensitive enough in brain analyses (see Genovese, Lazar, & Nichols, 2002). This “multiple comparisons problem” was corrected for by setting a voxel‐wise false discovery rate (FDR) = 0.05 (see https://surfer.nmr.mgh.harvard.edu/fswiki for further details on implementation of FDR). This same correction method was applied to the SPM analyses as well (described below) in order to maintain consistency across programs. Both cortical thickness and volume were extracted from FreeSurfer.

Based on findings that AoA across all bilinguals as a continuous variable predicts GM structure (Klein et al., 2014; Nichols & Joanisse, 2016; Wei et al., 2015), we also conducted a regression with AoA as a predictor of brain structure (in one regression the dependent variable was cortical thickness; in a separate regression the dependent variable was volume) across each hemisphere using an FDR of 0.05. A regression was also conducted with AoA as a predictor of brain structure controlling for English proficiency and percent daily Spanish use. For all ANOVA, ANCOVA, and regression analyses, results that survived the FDR correction were displayed and examined on the three‐dimensional brain template that is built into Freesurfer, which was then parceled according to the Desikan–Killiany atlas (Desikan et al., 2006).

#### 3.6.2 SPM CAT12—Voxel‐based volume and density

The same one‐way ANOVA and ANCOVA computed in FreeSurfer were used in SPM12, with AoA group as the factor and proficiency as the covariate. The same orthogonal contrasts were used for interpretation: monolinguals versus bilinguals (i.e., early and late bilinguals), and early bilinguals versus late bilinguals. The same regressions conducted in Freesurfer were used in SPM12, with AoA as a predictor of each measure of brain structure (volume or density), and English proficiency and daily Spanish use as nuisance variables. Both volume (modulated) and density (unmodulated) results were extracted from SPM. Results were displayed using xjview, a neuroanatomical viewing toolbox for SPM12. FDR‐correction was applied in SPM, and results with a cluster size larger than or equal to 10 voxels are presented here.

## 4 RESULTS

**Figure 1:**
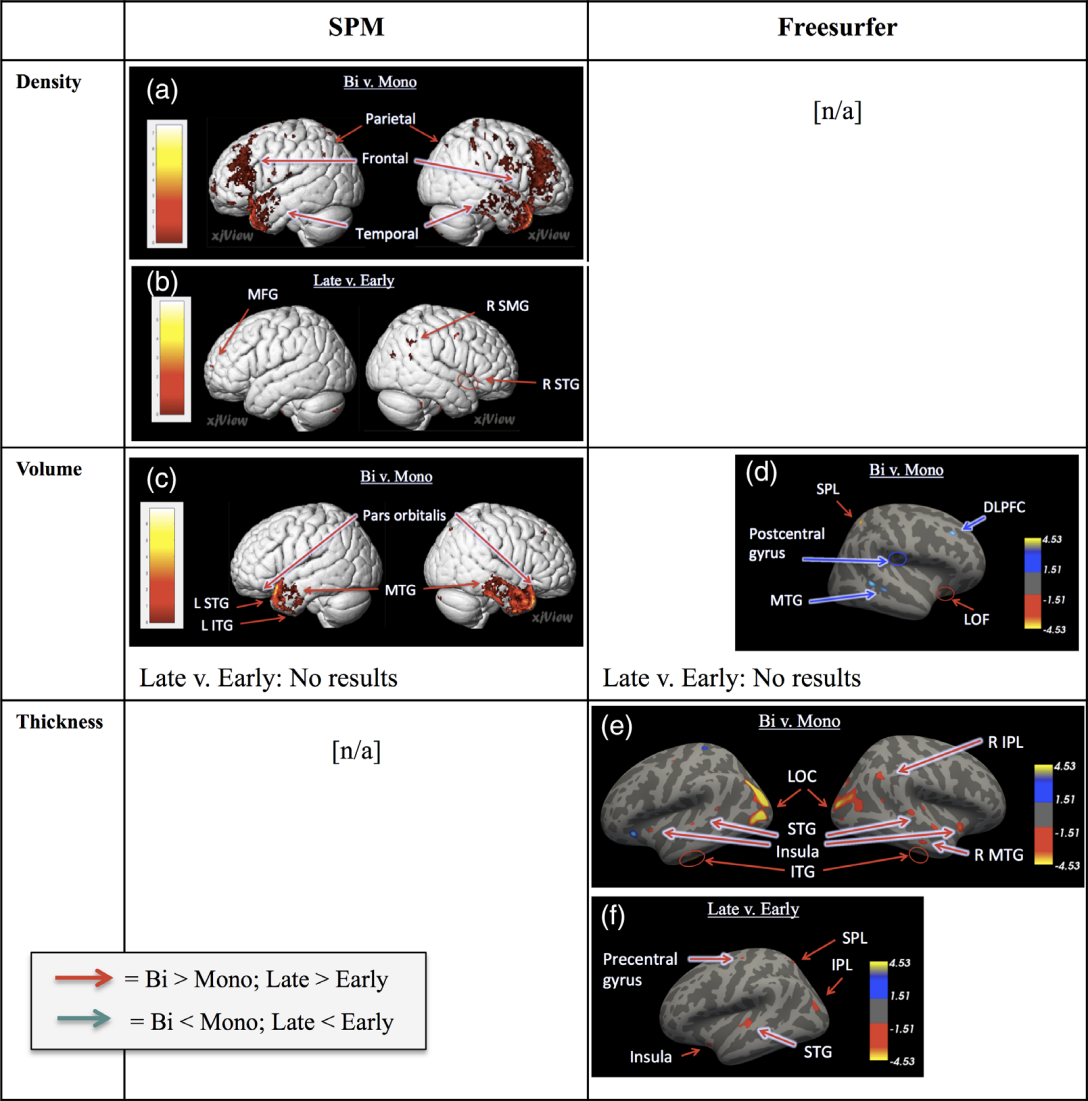
Comparison of ANOVA results showing relationship between AoA and neuroanatomical metric. Three neuroanatomical metrics (gray matter density, volume, and thickness) were analyzed using ANOVA with age of acquisition (AoA) group (monolingual, early bilingual, or late bilingual) as the factor. Warm colors indicate greater density/volume/thickness (bi > mono or late > early) and cold colors indicate lesser density/volume/thickness (bi < mono or late < early). FDR‐corrected *p* < .05. DLPFC, dorsolateral prefrontal cortex; IPL, inferior parietal lobule; ITG, inferior frontal gyrus; L, left; LOC, lateral occipital cortex; LOF, lateral orbitofrontal cortex; MFG, middle frontal gyrus; MTG, middle temporal gyrus; R, right; SMG, supramarginal gyrus; SPL, superior parietal lobule; STG, superior temporal gyrus [Color figure can be viewed at http://wileyonlinelibrary.com]

**Figure 2:**
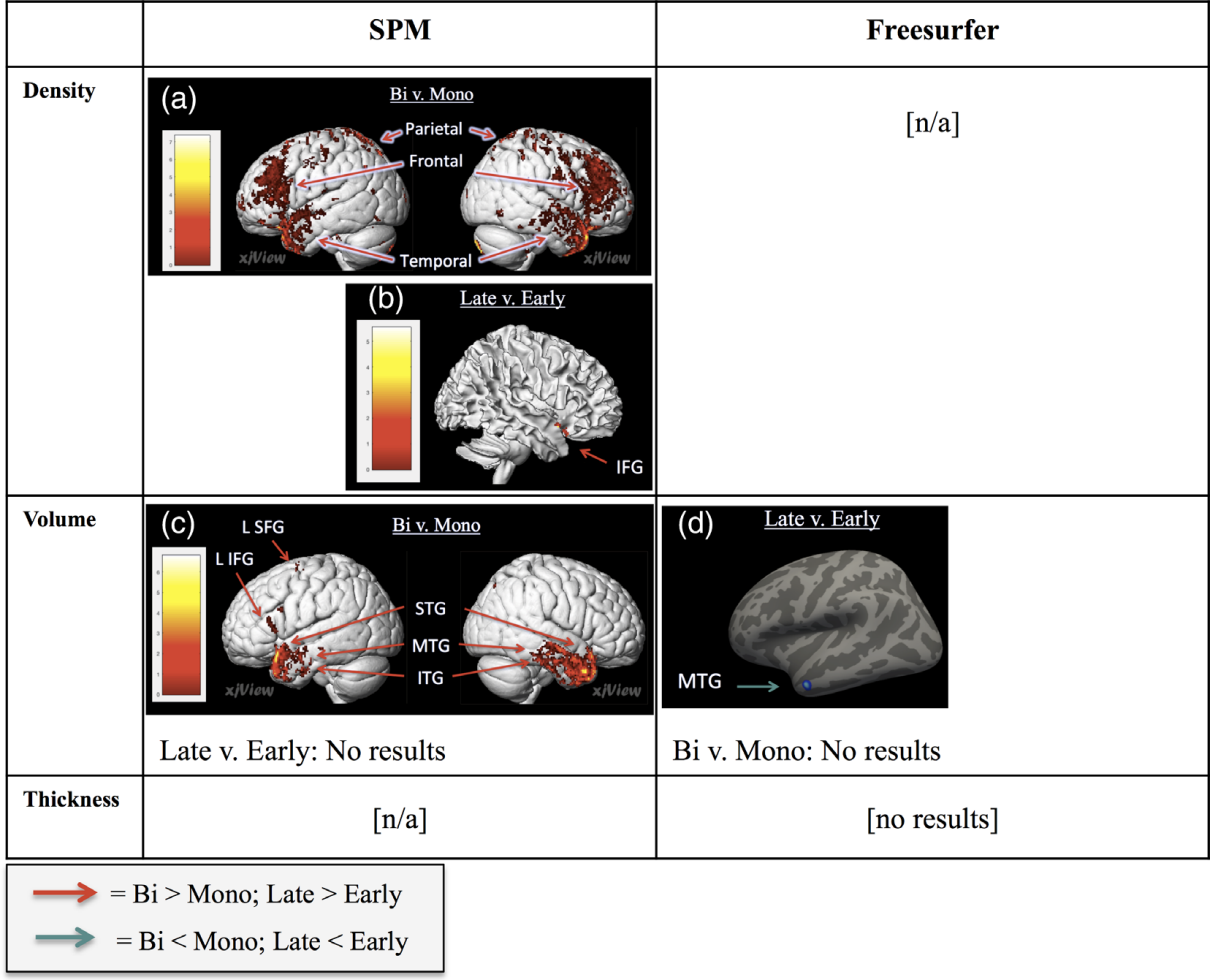
Comparison of ANCOVA results with proficiency as a covariate. Three neuroanatomical metrics (gray matter density, volume, and thickness) were analyzed using ANCOVA with AoA group (monolingual, early bilingual, or late bilingual) as the factor and English proficiency as a covariate. Warm colors indicate greater density/volume/thickness (bi > mono or late > early) and cold colors indicate lesser density/volume/thickness (bi < mono or late < early). FDR‐corrected *p* < .05. IFG, inferior frontal gyrus; ITG, inferior frontal gyrus; L, left; MTG, middle temporal gyrus; SFG, superior frontal gyrus; STG, superior temporal gyrus [Color figure can be viewed at http://wileyonlinelibrary.com]

Whole‐brain analyses were conducted on 334 brain scans using general linear modeling to determine differences in brain structure relative to AoA. Because previous studies have found neuroanatomical effects associated with AoA by conducting analyses that treat AoA as categorical (i.e., Berken et al., 2016b; Kaiser et al., 2015; Klein et al., 2014; Mohades et al., 2012) and continuous (i.e., Klein et al., 2014; Nichols & Joanisse, 2016; Wei et al., 2015), this study used both group comparisons (ANOVA) and regression analyses (in which AoA was treated as a continuous variable). Analyses of cortical thickness, volume, and density were conducted. A summary of all contrasts that were significant can be seen in Table 3. Results from analyses conducted in Freesurfer (cortical thickness and volume) were displayed on Freesurfer's inflated brain template and labeled following the Desikan–Killiany atlas (Desikan et al., 2006). Results from analyses conducted in SPM12 (volume and density) were displayed using the “Render View” option in the xjview toolbox. The resulting significant clusters were then compared to the hypothesized areas. Results have been summarized in Figures 1 and 2.

**Table 3:** Summary of contrasts that were significant

(a) ANOVA		
**Metric**	**SPM**	**Freesurfer**
**Density**	Bi versus mono Late versus early	n/a
**Volume**	Bi versus mono	Bi versus mono Late versus early
**Thickness**	n/a	Bi versus mono Late versus early
**(b) ANCOVA**		
**Metric**	**SPM**	**Freesurfer**
**Density**	Bi versus mono Late versus early	n/a
**Volume**	Bi versus mono	Late versus early
**Thickness**	n/a	(No results)

*Note*: Orthogonal contrasts were created with monolingual, early bilingual (AoA = 4–7 years), and late bilingual (AoA ≥8 years) groups as the factor. This led to two comparisons tested by ANOVA: Late versus early, and bi versus mono. SPM was used to test gray matter density and gray matter volume; Freesurfer was used to test gray matter volume and cortical thickness. Tables show a summary of contrasts that were significant. (a) One‐way ANOVAs with AoA group as the factor (monolingual, early bilingual, or late bilingual) and gray matter metric (density, volume, or thickness) as the dependent variable. (b) One‐way ANCOVAs with AoA group as the factor, English proficiency as the covariate, and gray matter metric as the dependent variable.

As mentioned above, AoA is one of the strongest predictors of proficiency. Parsing these variables out from one another does not always lead to the most informative analyses. In the present study, we include both an ANOVA with AoA as a predictor of brain structure, and an ANCOVA with AoA as a predictor of brain structure and English proficiency as a covariate. This is different from matching participants' proficiency, which would result in a sample that is not representative of the true population. That is, in order to match simultaneous bilinguals (who tend to have the highest proficiency in both languages; Birdsong, 2018) and late bilinguals (who tend to have lower proficiency), it would be necessary to pick only the highest‐performing late bilinguals (who are outliers because they are experts at language beyond what would be expected given their language history) and the lowest‐performing simultaneous bilinguals (who are outliers and likely have some other reason that they have low proficiency, such as a lapse in education or low SES). Instead, the focus of the current study was to examine the structural changes associated with AoA as potential factors that explain why AoA is such a strong predictor of proficiency. As such, we include findings both with and without English proficiency as a covariate.

### 4.1 Cortical thickness

#### 4.1.1 ANOVA

An one‐way ANOVA was carried out to test the difference between groups (monolingual, early bilingual, late bilingual), where the null hypothesis was that brain structure was equal across the three groups. This was accomplished with the following set of two orthogonal contrasts: early versus late bilinguals and monolinguals versus all (i.e., early and late) bilinguals. The ANOVA of cortical thickness was significant (FDR‐corrected *p* < .05). Significance tests for the first contrast showed that late bilinguals had thicker cortex than early bilinguals in six clusters, including the left SPL, IPL, precentral gyrus, STG, and insula. Significance tests for the second contrast showed that bilinguals had thicker cortex than monolinguals in 45 clusters, especially the bilateral LOC, insula, STG, ITG, fusiform, right MTG and lingual gyrus (FDR‐corrected *p* < .05; see Figure 1; see Table 4 for the 10 largest cortical clusters in each contrast; see Appendix A for detailed information on all significant clusters).

**Table 4:** Largest gray matter clusters where bilingualism related to thickness––Freesurfer

Group	Hem	Region	*t*	# Vertices in cluster	Peak MNI coordinates *x y z*
*Late versus early*					
L > E	L	Superior parietal lobule	4.69	181	−18.3, −60.9, 57.3
	L	Precentral gyrus	4.43	221	−9, −24.4, 67.3
	L	Inferior parietal lobule	4.41	202	−41.3, −74.4, 19.3
	L	Superior temporal gyrus	4.40	429	−58.6, −18.8, 2.1
	L	Insula	4.07	118	−33.7, 4, −12.3
	L	Precentral gyrus	3.80	100	−36.9, −16.9, 56
*Bilingual versus monolingual*					
B > M	L	Inferior parietal lobule	6.73	1915	−35.4, −79.3, 24.9
	L	Precuneus	4.53	919	−18.6, −56.2, 18
	L	Lateral occipital cortex	6.46	901	−41.1, −80.4, 7.9
	L	Medial orbitofrontal	5.49	381	−4.3, 37.2, −19.7
	L	Paracentral gyrus	4.28	271	−3.5, −29.9, 61
	R	Lateral occipital cortex	5.30	1,588	29.7, −85.8, 16.7
	R	Paracentral gyrus	8.02	544	3.7, −29.1, 60.7
	R	Lingual gyrus	4.24	403	6.9, −64, 6
	R	Insula	4.26	258	37.1, 6.2, −5.3
B < M	L	Lateral orbitofrontal	−4.37	261	−31.8, 28.6, −4.7

*Note*: Hem, hemisphere. MNI, Montreal Neurological Institute. Regions are significant at FDR‐corrected *p* < .05. L, late bilingual (AoA > 7 years); E, early bilingual (AoA 4–7 years); B, bilingual; M, monolingual. For the late versus early contrast only 8 total clusters were significant. The bilingual versus monolingual contrast lists the top 10 clusters of 47 total clusters. See Appendix for full set of clusters.

#### 4.1.2 ANCOVA

An ANCOVA with AoA group as the factor, English proficiency as a covariate, and cortical thickness as the dependent variable did not yield significant results.

### 4.2 Density

#### 4.2.1 ANOVA

Late bilinguals had greater density than early bilinguals in a set of clusters that included the bilateral MFG, IFG, right MTG, supramarginal gyrus (SMG), and left SFG. Bilinguals had greater density than monolinguals in a broad set of cortical regions that included bilateral temporal, frontal, and parietal regions (FDR‐corrected *p* < .05, see Figure 1 and Table 5; see Appendix B for details on all clusters).

**Table 5:** Ten largest gray matter clusters where bilingualism related to density––SPM

Group	Hem	Region	*t*	# Vertices in cluster	Peak MNI coordinates *x y z*
*Late versus early*					
L > E	L	Middle frontal gyrus	4.99	39	−6, 16.5, −22.5
	L	Inferior frontal gyrus	4.77	21	−13.5, 12, −22.5
	R	Middle frontal gyrus	5.48	42	6, 16.5, −18
	R	Inferior frontal gyrus	5.11	38	34.5, 10.5, −13.5
	R	Supramarginal gyrus	4.26	30	61.5, −54, 36
	R	Middle frontal gyrus	4.53	19	48, 1.5, 46.5
	R	Superior frontal gyrus	5.05	18	21, 52.5, −3
	R	Middle temporal gyrus	4.50	13	51, −73.5, 22.5
	R	Superior temporal gyrus	3.91	12	63, −54, 22.5
	R	Inferior frontal gyrus	4.855	11	15, 16.5, −22.5
*Bilingual versus monolingual*					
B > M	L	Frontal lobe	7.46	35,941	−46.5, 18, −36
	L	Parietal lobe	4.57	472	−28.5, −45, 70.5
	L	Precentral gyrus	4.61	283	−66, 3, 18
	L	Temporal lobe	5.10	282	−31.5, −27, −31.5
	L	Postcentral gyrus	4.58	242	−55.5, −6, 51
	L	Superior temporal gyrus	4.20	184	−51, −31.5, 7.5
	L	Occipital lobe	4.92	137	−4.5, −102, 3
	R	Parietal lobe	4.59	409	16.5, −49.5, 54
	R	Paracentral lobule	4.67	307	9, −33, 64.5
	R	Frontal lobe	5.04	164	13.5, 55.5, 7.5

*Note*: Significant at FDR‐corrected *p* < .05. Hem, hemisphere; MNI, Montreal Neurological Institute. Regions are significant at FDR‐corrected *p* < .05. L, late bilingual (AoA > 7 years). E, early bilingual (AoA 4–7 years). B, bilingual. M, monolingual. This table only lists the top 10 largest clusters for each contrast. See Appendix for full set of clusters.

#### 4.2.2 ANCOVA

An ANCOVA with English proficiency as the covariate revealed that late bilinguals had greater density than early bilinguals in the right IFG. The ANCOVA also revealed that bilinguals had greater density than monolinguals in a broad set of regions that included bilateral frontal, parietal, and temporal regions (see Figure 2; see Appendix B for details on all clusters).

### 4.3 Volume

Both SPM12 and Freesurfer are capable of yielding volume results. Because some discrepancies among previous studies' results may have to do with use of different programs, we present results from both programs here. The results across the two volume analyses (Freesurfer and SPM) were not identical, likely because of differences between Freesurfer's surface‐based calculation and SPM's voxel‐based calculation. Because these differences may inform future research, we include both sets of results here.

#### 4.3.1 ANOVA

An ANOVA in Freesurfer showed that bilinguals had significantly different cortical volume from monolinguals in eight clusters, with bilinguals showing greater volume than monolinguals in the right cuneus, precuneus, and SPL, and lesser volume in the right MTG, DLPFC, and postcentral gyrus (Figure 1, Table 6; see Appendix C for further details). The same ANOVA in SPM12 showed that bilinguals had greater volume than monolinguals in 41 clusters that were especially concentrated in the temporal lobe; bilinguals had lesser volume than monolinguals in 29 clusters, including the left IFG, MFG, and SFG (FDR‐corrected *p* < .05; Figure 1; see Table 7 for 10 largest clusters; see Appendix D for all clusters).

**Table 6:** Clusters where bilingualism related to volume––Freesurfer

Group	Hem	Region	*t*	# Vertices in cluster	Peak MNI coordinates *x y z*
*Bilingual versus monolingual*					
B > M	R	Precuneus	6.90	309	22, −54.8, 20.4
	R	Superior parietal lobule	4.40	118	35.6, −43.7, 57.6
	R	Lateral orbitofrontal	4.16	70	27.6, 17.7, −17.8
	R	Cuneus	4.00	53	4.2, −75.3, 27.9
B < M	R	Superior temporal sulcus	−4.53	220	45.3, −38.8, 0.4
	R	Dorsolateral prefrontal cortex	−4.40	104	37.8, 32.4, 26.9
	R	Postcentral gyrus	−3.91	78	46.2, −14.7, 19.1
	R	Middle temporal gyrus	−4.14	77	44.7, −28.1, −5

*Note*: Hem, hemisphere; MNI, Montreal Neurological Institute; B, bilingual; M, monolingual. Regions are significant at FDR‐corrected *p* < .05. See Appendix for full set of clusters.

**Table 7:** Ten largest gray matter clusters where bilingualism related to volume––SPM

Group	Hem	Region	*t*	# vertices in cluster	Peak MNI coordinates *x y z*
*Bilingual versus Monolingual*					
B > M	L	Superior temporal gyrus	6.43	699	−45, 21, −25.5
	L	Precuneus	6.91	266	−16.5, −64.5, 16.5
	L	Parahippocampal gyrus	4.66	230	−24, −3, −22.5
	L	Pars orbitalis	6.32	230	−37.5, 18, −18
	L	Middle temporal gyrus	4.63	104	−63, −6, −25.5
	L	Inferior temporal gyrus	4.74	100	−54, −7.4, −37.5
	R	Middle temporal gyrus	6.29	2,224	42, 22.5, −36
	R	Precuneus	6.66	453	19.5, −61.5, 18
	R	Parahippocampal gyrus	4.80	325	24, −4.5, −21
	R	Pars orbitalis	5.67	159	21, 13.5, −25.5

*Note*: Hem, hemisphere; MNI, Montreal Neurological Institute; B, bilingual; M, monolingual. Regions are significant at FDR‐corrected *p* < .05. This table only lists the top 10 largest clusters. See Appendix for full set of clusters.

#### 4.3.2 ANCOVA

An ANCOVA in Freesurfer with English proficiency as the covariate revealed that late bilinguals had lesser volume than early bilinguals in the left MTG. The same ANCOVA in SPM12 showed that bilinguals had greater volume than monolinguals in 39 clusters, including the bilateral STG, MTG, ITG, MFG, left IFG, SFG, occipital lobe, cuneus, and right lingual gyrus (FDR‐corrected *p* < .05; Figure 2; see Appendix C for all clusters).

### 4.4 Regression

None of the regression results were significant for thickness, volume, or density.

## 5 DISCUSSION

**Figure 3:**
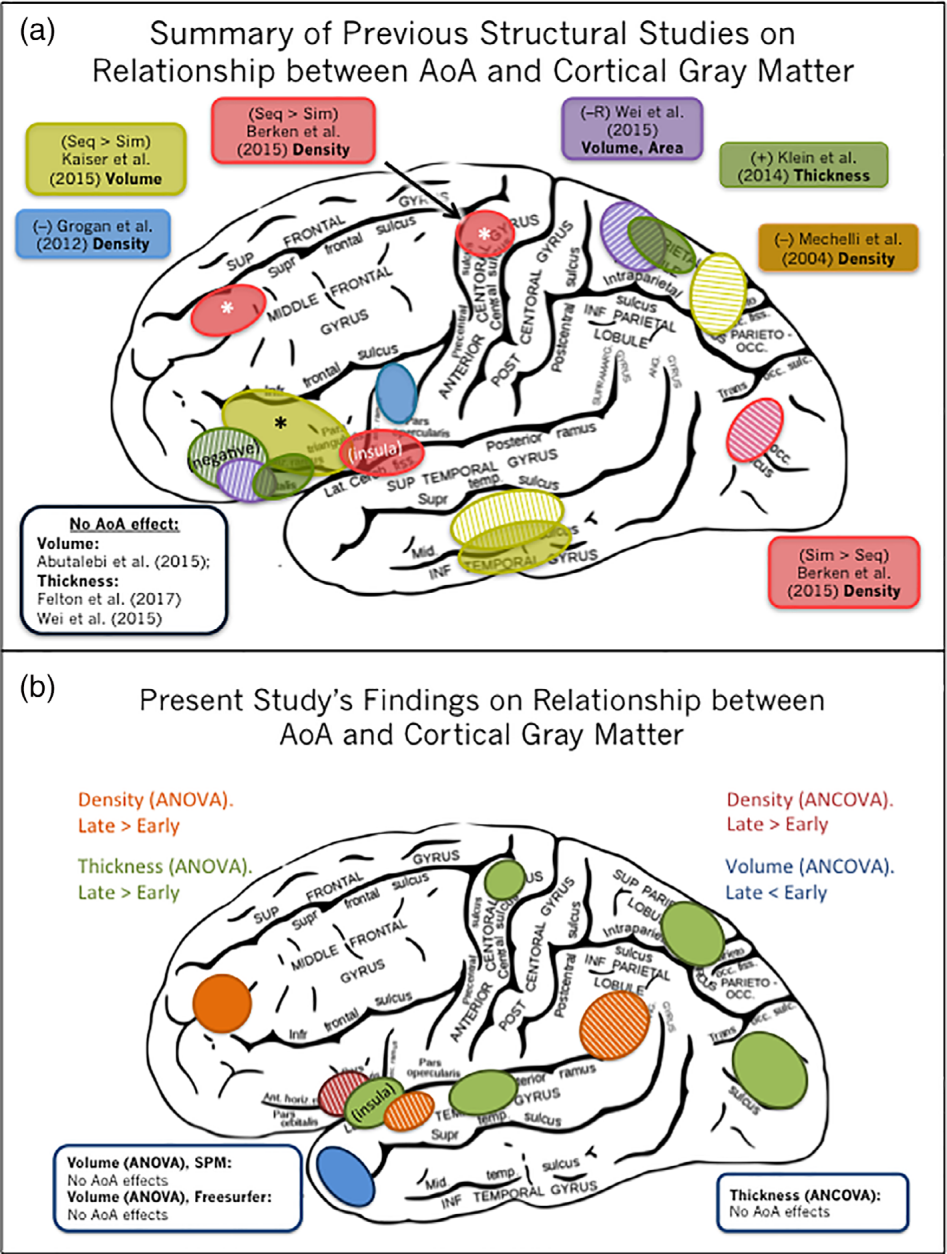
Summary of previous findings versus present results. 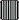 = right hemisphere. * = bilateral. Otherwise left hemisphere. +/− indicates positive or negative correlation with AoA. “R” indicates regression. “Seq” = sequential bilingual. “Sim” = simultaneous bilingual. (a) Localizations are for visualization purposes only; Actual size/specificity of cluster may vary. Medial sections not shown. (b) Summary of present findings. Left cerebral hemisphere outline: Gray (1918; public domain) [Color figure can be viewed at http://wileyonlinelibrary.com]

Although researchers generally agree that a certain set of brain areas underlie bilingual language processing, it is unclear what effect timing of language acquisition has on these regions. Discrepancy among previous findings may be driven by methodological differences, so this study aimed to elucidate this relationship by analyzing a set of three neuroanatomical correlates (GM density, volume, and thickness) of AoA. In the present sample, analyses of GM density revealed AoA‐related differences in regions associated with execution of speech and articulation that may occur within a fronto‐parietal control network for top‐down selection of words, analyses of thickness revealed changes to areas involved in language control, and analyses of volume revealed a smaller set of clusters localized to either the temporal lobes (when analyzed in SPM) or the MTG and DLPFC (when analyzed in Freesurfer). These differences were found when AoA was analyzed categorically in ANOVA analyses (no linear regression results were significant), which implies that language development is better characterized by optimal windows of development than a linear trajectory. Our results demonstrate two main points: (a) models of bilingualism that focus on the neural underpinnings of language control should consider the effects of development on language organization, (b) given that the present findings differed according to neuroanatomical metric and neuroimaging software, neuroscience researchers should consider the role that these parameters play when designing analyses, and (c) although results from previous studies are discrepant, they may each be capturing smaller pieces of a larger picture (see Figure 3).

At the present time, research in the field of bilingualism that has examined the neuroanatomical correlates of AoA has tended to analyze only one metric of brain structure at a time. This is important to acknowledge because in the present study, our findings were driven by which neuroanatomical metric of AoA was used. Changes to neuropil, glia, dendrites, number of soma, and/or underlying white matter structure may all influence each metric (Wenger, Brozzoli, Lindenberg, & Lövdén, 2017), and likely do so in a unique manner. For example, Mechelli and colleagues note that GM density as measured by voxels, “should not be confused with cell packing density measured cytoarchitectonically” (Mechelli, Price, Friston, & Ashburner, 2005, p. 8) because it estimates the overall dimension of an area rather than which cellular adaptations cause that area to expand or compress. Therefore, it is important to avoid characterizing the neuroanatomical correlates of AoA according to only one metric of GM until researchers are able to elucidate the developmental trajectory of each metric.

### 5.1 Effects of AoA differ according to neuroanatomical metric

Cortical thickness analyses revealed that late bilinguals had thicker cortex than early bilinguals in regions that have been classically implicated in language control. These regions included those that have been implicated in prelexical speech (left STG; Price, 2010), as well as verbal working memory and integrating auditory perception with motor production (left IPL and precentral gyrus; Alain, Arnott, Gillingham, Leung, & Wong, 2015; Simonyan & Fuertinger, 2015). Late bilinguals also had thicker cortex in the left SPL, which is implicated in top‐down attentional orienting (Shomstein, 2012). This is in line with Klein et al. (2014), who also found that later AoA relates to thicker left SPL, although in their study this was a continuous (not categorical) relationship. The findings of the left SPL, IPL, and STG fit with the argument that late bilinguals in particular may achieve L2 resonance via metacognitive processes that rely on nonlanguage areas (e.g., recoding, rehearsal, and imagery) in order to defend against L1 entrenchment (Berken et al., 2017; Hernandez, Claussenius‐Kalman, Ronderos, & Vaughn, 2018; Hernandez, Li, & MacWhinney, 2005).

Density analyses revealed that late bilinguals had greater density (i.e., higher voxel concentration) than early bilinguals in regions related to speech and articulation more so than language control, including the bilateral MFG, IFG, right SMG, and left SFG. The MFG is activated when participants are asked to generate semantically‐ or phonologically‐related words to a stimulus word (Heim, Eickhoff, & Amunts, 2009). Regarding the bilateral IFG, Price (2010) notes that the left pars opercularis of the IFG is involved in processing speech and syntax, but that activation increases in both the left and right pars opercularis when extraction of semantic content in a sentence is particularly difficult. This means that greater use of the pars opercularis may be a product of using top‐down predictions for plausible events. Learning to make predictions about the meanings of sentences without understanding every part of the sentence is a common experience for late bilinguals, so it is possible that they use context‐based mental predictions in order to process the L2.

Volume analyses showed that late bilinguals had lesser volume than early bilinguals in the left MTG. Interestingly, Kaiser et al. (2015) also found AoA effects of volume, where sequential bilinguals had greater volume than simultaneous bilinguals in the MTG, although their findings were right‐lateralized and more posterior than our finding. Price (2010) notes that the middle temporal lobe is likely implicated in making meaning of and integrating multiple semantic concepts in both written and auditory form. For example, activation in the MTG has been shown to occur in response to hearing grammatically correct sentences with plausible (vs. implausible) meanings (Rogalsky & Hickok, 2009), and the anterior MTG has been implicated in reading content words with high semantic associations (Davis & Gaskell, 2009; Diaz & McCarthy, 2009). The fact that late bilinguals in this sample showed greater volume in this area may have to do with learning to read in the L2 at the same time as learning the L2 itself, whereas earlier bilinguals on average may have more time to begin speaking the language before learning to read (similar to monolinguals).

Of all three measures, GM volume was overall less sensitive to AoA than density or thickness. It may be the case that GM density and thickness are more sensitive metrics to AoA‐related cortical adaptations. Whereas Freesurfer analyses of GM volume revealed that early bilinguals had greater volume than late bilinguals in the left MTG when controlling for English proficiency, SPM analyses did not show any significant difference between these groups. This may be due to differences between Freesurfer's surface‐based calculation and SPM's voxel‐based calculation. Had the present study only examined GM volume in SPM, it likely would have been concluded that AoA does not have a significant effect on brain structure. In fact, the lack of significant AoA‐related volume results in SPM is in line with results from Abutalebi et al. (2015). As far as choice of specific neuroanatomical metric, volume may be less sensitive to AoA than other metrics. Unlike thickness, volume is influenced by surface area, which is the measure of differences in regional expansion across subjects (Ronan & Fletcher, 2015) and is affected by both the width of sulci and the number of gyrifications in a given area (Im et al., 2008). Given that surface area is included in calculations of volume (and not in calculations of cortical thickness), one possible explanation for the lack of findings is that cortical adaptations in response to timing of language adaptation are less likely to occur in the direction of surface area and more likely to occur in the direction of thickness.

### 5.2 Development and its role in language organization

Cognitive control (Abutalebi & Green, 2007) and developmental (Hernandez & Li, 2007) models make separate predictions regarding the role of development in determining language organization. The adaptive control hypothesis (Abutalebi & Green, 2007; Green & Abutalebi, 2013) provides an account for how bilinguals control their two languages: the left basal ganglia and ACC modulate left prefrontal cortex activity, which permits the prefrontal cortex and IPL to work in tandem as a top‐down control system when executing word selection (Abutalebi & Green, 2007; Green & Abutalebi, 2013). This model accounts for bilingual language control, but further models are needed to predict the effects of development on these cognitive processes. The sensorimotor hypothesis (Hernandez & Li, 2007) posits that skill acquisition occurs through recruitment of brain structures that are in the midst of development at the time of acquisition. Specifically, skill acquisition in children may occur through sensorimotor processes involving the basal ganglia due to its high plasticity in early development, whereas skill acquisition in adults may be facilitated by later‐developing cortical structures involved in higher cognitive processing (such as the temporal and parietal regions and their connections to the medial temporal lobe; Ullman, 2016), and metacognition (such as the frontal lobe; Bradley, King, & Hernandez, 2013).

Overall, the changes described above are in line with Abutalebi and Green's model of bilingual language control. Specifically, late bilinguals had thicker cortex than early bilinguals in the left IPL and higher voxel concentration (i.e., density) in the right SMG of the IPL and bilateral frontal areas. These structural changes may be the result of learning to link novel phonemes to pre‐existing semantic information while utilizing top‐down control processes for word selection. Although Abutalebi and Green's model focuses on bilingualism at large and does not focus on AoA effects, the present results imply that this model may have separate implications for late and early bilinguals.

In fact, research has begun to show that if there is a relationship between dual language control and cognitive control (Bialystok, Craik, & Luk, 2012; see Paap, Johnson, & Sawi, 2015 for information on bilingual advantage debate), any effects may differ according to AoA. For instance, Tao, Marzecová, Taft, Asanowicz, and Wodniecka (2011) provide evidence for the case that late bilinguals have an advantage at inhibiting nonrelevant information, whereas early bilinguals have an enhanced monitoring system that evaluates whether there is a need to engage cognitive control. Others have found that early bilinguals respond faster than late bilinguals or monolinguals on incongruent Flanker trials (Luk, Bialystok, Craik, & Grady, 2011) and attentional network tests (Kapa & Colombo, 2013). In line with the sensorimotor hypothesis, late bilinguals may depend more on later‐developing cortical structures to facilitate the L2, leading to the structural changes seen here.

It is also important to also consider that the neuroanatomical correlates of AoA differed between the ANCOVA (where English proficiency was the covariate) and the ANOVA, as displayed in Figure 3. This finding demonstrates the point that inclusion/exclusion of particular variables in the field of bilingual research may influence replicability across studies. English proficiency is unique because, instead of being an explanatory variable, it is an outcome variable that is the result of a few main factors (AoA, language exposure, motivation to learn, and potential genetic factors; Vaughn & Hernandez, 2018). Given that, early AoA is a consistently strong predictor of having higher proficiency in both languages (Birdsong, 2018), attempts to separate these two variables may lead to results that are less informative. For instance, matching early and late bilinguals on proficiency would require picking only the early bilinguals with English proficiency that is lower than expected in order to match the late bilinguals who have English proficiency that is higher than expected. In both cases, these individuals would be outliers because earlier AoA is expected to lead to higher L2 proficiency (Granena & Long, 2013; Johnson & Newport, 1989).

In light of the complexity of the decision to include or exclude this variable as a covariate, we analyzed AoA effects both without (ANOVA) and with English proficiency (ANCOVA) as a covariate. As can be seen in Figure 3, significant regions did not overlap between these two analyses. In the ANCOVA, late bilinguals had greater GM density in the right IFG than early bilinguals; in the ANOVA, late bilinguals had greater density in the right STG, SMG, and left MFG. This result demonstrates that even when two studies examine the same neuroanatomical metric, inclusion of English proficiency can result in a separate set of significant clusters. For late versus early bilingual comparisons, volume results were only significant when English proficiency was a covariate. On the other hand, thickness results were only significant when there was no covariate. This implies that for thickness, regions where late bilinguals had thicker cortex than early bilinguals might be explained by proficiency. However, this does not mean that the ANOVA results are not meaningful. Rather, this points out the close connection between AoA and proficiency, and provides insight into the specific areas that may be changing to help explain why AoA is such a strong predictor of English proficiency in the first place.

Furthermore, AoA effects were found for volume with Freesurfer (not SPM), and these results were only significant when English proficiency was included as a covariate in the ANCOVA (and not in the ANOVA). This finding is interesting because volume was overall the least sensitive metric to AoA effects. The fact that late bilinguals had lesser volume than early bilinguals in the left temporal pole shows that including English proficiency as a covariate may remove variance that can, in some cases, obscure effects. Figure 3 displays these findings, which emphasizes an additional point, which is that the inclusion/exclusion of English proficiency as a covariate may be one influential variable on findings across studies.

### 5.3 Differences between bilinguals and monolinguals

The current study also considered structural differences between bilinguals and monolinguals. GM analyses revealed differences in a broad set of cortical regions that varied depending on the metric. Cortical thickness analyses showed that bilinguals had thicker cortex than monolinguals in a set of clusters concentrated near the occipital and temporal lobes, especially the bilateral LOC, STG, ITG, and right MTG and lingual gyrus. Bilinguals showed greater density than monolinguals in a broad set of bilateral temporal, parietal, and frontal regions (see Figure 1). Volume results were not as straightforward since the results differed between SPM12 and Freesurfer (likely influenced by SPM's voxel‐based and Freesurfer's surface‐based calculations). Results from the analyses using SPM12 showed that bilinguals had greater volume than monolinguals in the bilateral temporal lobes and lesser volume in the left IFG, MFG, and SFG. Results using Freesurfer showed that bilinguals had greater volume than monolinguals in the right cuneus and SPL, and lesser volume in the right MTG and DLPFC.

These findings demonstrate that the ability to speak two languages can be linked to differences in brain structure. Multiple factors may play into these adaptations, including the need for top‐down selection of words and inhibition of words in the nontarget language (Costa, Pannunzi, Deco, & Pickering, 2016; Hernandez et al., 2019). In the present case, density analyses were more sensitive to alterations that may occur within a fronto‐parietal control network, thickness analyses were more sensitive to alterations in brain areas associated with visual and semantic processing, and volume was more sensitive to either temporal (SPM) or a smaller set of regions in the middle temporal lobe and DLPFC (Freesurfer). Given that bilingualism is often treated as a monolithic variable, it is interesting that the regions seen here did not necessarily overlap with those in the late versus early bilingual comparisons (see Figure 1). These findings emphasize the point that the effects of language differ in relation to individual differences in language experience, and that different neuroanatomical metrics are sensitive to different sets of language‐related adaptations.

### 5.4 The role of software

The field of neuroimaging research is fortunate to have access to multiple software packages to conduct analyses that would have been previously not pragmatic or possible due to the complex computational analyses required to measure the thousands of voxels in one brain volume. Although the present study examined two popular software packages, researchers today have a plethora of options, including FSL (Functional MRI Brain Software Library; https://fsl.fmrib.ox.ac.uk), Brainsuite (https://brainsuite.org), and AFNI (Analysis of Functional NeuroImages; https://afni.nimh.nih.gov). Whitwell (2009) notes that preprocessing steps vary across studies and software packages, with some using different degrees of smoothing, registration, and default algorithms for segmentation. One group of researchers (Bowring, Maumet, & Nichols, 2019) tested comparability across AFNI, FSL, and SPM, and determined that even in cases where packages intended to use the same MNI space, activation detected by some programs AFNI and FSL continued to fall outside of SPM's analysis masks.

As mentioned in the Method, the programs chosen for the present study, SPM and Freesurfer, use different calculation methods and make different assumptions. In an examination of the consistency between FSL, Freesurfer, and SPM for measurement of GM atrophy, Popescu et al. (2016) found marked differences between packages, with a wide range of intraclass correlation coefficient values. Agreement between Freesurfer and SPM was lowest in the occipital lobes and insula, but the majority of disagreement between programs was with deep GM structures (e.g., thalamus, putamen, hippocampus). Popescu and colleagues note that a large part of these differences may be the influence of the different atlases used across programs. Given that both surface‐ and voxel‐based methods are valid for measuring the brain (Li et al., 2014), it is important for researchers to consider the retest reliability of analyses conducted with a the neuroimaging software of choice for the population of interest, the effects that software package has on the order of preprocessing steps, and the algorithms used to achieve these steps.

### 5.5 Replicability in neuroscience

In light of a replication crisis in neuroscience, we suggest that neuroscientists develop agreed‐upon metrics by which they will evaluate neuroimaging data in order to decrease variability that is caused solely by using differential analytical methods. The fact that the present study did not replicate many previous findings is not surprising, given the different sample populations and sample sizes across previous studies. For example, Mechelli et al. (2004) used 22 native Italian‐English bilinguals and found that later AoA related to decreased left IPL density, whereas we used 334 Spanish‐English bilinguals and English monolinguals in Houston, TX and did not find this effect (see Table 1 for full summary of previous studies). Button et al. (2013) recommend that researchers disclose methods, data, and results transparently, and work to increase power and replicate findings. For researchers studying language acquisition and bilingualism, this means that there should be discussion about the most accurate methods of parameterizing AoA (as well as other language background variables), and that researchers should develop consensus on which neuroanatomical metrics provide the most accurate means by which to evaluate the effects of language timing on the brain. Achieving these goals should result in an increase in cross‐study comparability in neuroscience.

In addition to multiple ways of defining AoA and use of different neuroanatomical metrics, a third methodological difference across previous studies is that some studies use region‐of‐interest (ROI) analyses that do not match across studies. ROI analyses can help investigate the role of one or a few regions (and are often necessary in order to obtain high enough power to conduct analyses; Poldrack, 2007), but this type of analysis removes the possibility of finding novel effects because regions are generally hypothesized based on previous findings. In addition to the regions that we hypothesized the whole‐brain analyses would reveal, this study also found that AoA was related to many regions that were not hypothesized, which have been listed in the Appendices. These nonhypothesized findings underscore the importance of obtaining enough power to conduct whole‐brain analyses. Therefore, inclusion of multiple measures of neuroanatomy (and measuring them using whole‐brain analyses) will be important for building our understanding of the neuroanatomical correlates of AoA, especially while we anticipate larger advances in neuroimaging methodologies that will help us better relate constructs such as volume to specific changes in cell biology.

### 5.6 Limitations, alternative explanations, and future directions

One limitation is that our sample was specific to participants in Houston, Texas, who may differ from other populations because they have exposure to a higher‐than‐average number of foreign languages (Emerson, Bratter, Howell, Jeanty, & Cline, 2013). Another limitation is that, because later AoA correlates with fewer years of language use, the present results showing cortical expansion in late bilinguals relative to early bilinguals may reflect fewer years of experience with the L2. Wenger et al. (2017) propose that cortical structure follows a U‐shaped curve, where increased use of a region at the beginning of skill acquisition leads to initial GM expansion (reflecting neural, glial, and/or dendritic growth), which renormalizes following skill mastery. GM could eventually return to baseline, given enough time (see Hernandez et al., 2019, for nonlinear language development). However, given the vast differences in developmental processes occurring at the time of L2 acquisition (e.g., early development of motor/speech pathways), it is unlikely that language processing in late and early bilinguals would exactly mirror one another.

In the present sample, age was not significantly different between bilingual (*M* = 23.41, *SD* = 4.76) and monolingual (*M* = 23.31, *SD* = 4.94) participants; *t*(325) = 0.18, *p =* .86). However, the age difference between early (*M* = 22.09, *SD* = 3.65) and late (*M* = 25.75, *SD* = 5.57) bilinguals was significantly different; *t*(187) = −5.45, *p* < .001. Although this difference reached significance, the majority of cortical (frontal, temporal, occipital, and parietal) changes are have been found to peak in adolescence and stabilize in adulthood around age 20 (Raznahan et al., 2011; Shaw et al., 2008; Wierenga, Langen, Oranje, & Durston, 2014). Given that the difference between early and late bilinguals was 3 years and remained within the 20s (as opposed to comparing adolescent and nonadolescent brains), it is not likely that age would explain results across the sample of participants examined in this study. However, future studies may want to examine the relationship between age and language development in the 20s.

This study operated under the assumption that greater use of an area leads to expansion of that area. Most areas of the human cortex have six layers of inputs and outputs (Johnson & de Haan, 2015), some of which communicate with other cortical areas, and others of which communicate with subcortical areas such as the thalamus (Guillery & Sherman, 2002), so one limitation is that the exact reason for cortical expansion may vary by circumstance. Current MRI technology cannot provide information on the structure of cortical layers or individual cells in vivo, which means that neuroanatomical studies should be cautious in making direct inferences about changes to function until researchers are able to elucidate the relationship between regional brain function and structural expansion versus compression. However, given that there is a strong relationship between function and structural changes (e.g., Boyke, Driemeyer, Gaser, Büchel, & May, 2008; May, 2011), research on bilingual brain structure should not be ignored. It is still clear from our results that the neural adaptations required to support bilingualism are different when the L2 is added late versus early in development.

Ideally, neuroimaging studies would always have an ample number of participants in every group to provide adequate power to examine the hypotheses of interest. Future studies should focus on including simultaneous bilinguals in their analyses, a group which we did not have the power to examine here. Munson and Hernandez (2019) demonstrated the importance of obtaining adequate statistical power by conducting repeated subsampling on an MRI brain dataset to examine the effect of power on consistency in GM volume results. The researchers found that for neuroimaging studies with subsamples below 40 per group and an alpha = .025, the probability of finding a false positive result was higher than finding a true positive. Repeated subsampling on an MRI brain dataset showed that as subsample size increased to 50, the probability of a true positive increased beyond that of a false positive, and that the probability of a true positive increased steadily as group size increased. Meanwhile, the probability of a false positive remained approximately the same. Although the present paper had group sizes (68 late bilinguals, 121 early bilinguals, and 145 monolinguals) that are overall larger than is the norm for many neuroimaging studies, we still did not have enough power to include 27 simultaneous bilinguals that were dropped from analyses due to the disparity in group sizes. Future research should continue to aim to increase sample sizes, and especially focus on recruiting simultaneous bilinguals.

The fact that differences were only found when bilinguals were grouped categorically by AoA (and no linear regression results were significant) implies that language development is best characterized by optimal windows of development rather than having a linear trajectory, although the possibility remains that linear changes still exist at the functional or subcortical level. Future researchers should continue to explore the possibility that language development does not occur in a linear fashion. This could be done by examining the progression of the relationship between brain structure and AoA throughout the course of development by conducting longitudinal studies that begin in childhood. Future studies may also want to more deeply examine the interaction between AoA and other background variables, such as genetics, SES, and language exposure. Future studies should also examine how subcortical and functional differences culminate in bilinguals according to language experience.

## 6 CONCLUSION

The present study aimed to create clarity regarding previous studies of the relationship between language organization in the brain and timing of second language acquisition. Discrepancy in the findings reported by previous studies is likely due to methodological differences across studies. To summarize the present findings, our results suggest that late bilinguals handle L2 acquisition via structural changes to a set of areas that are involved in cognitive control and language processing, but that the exact changes that occur depend on which measure of GM (density, volume, or thickness) is used. Relative to early bilinguals, late bilinguals showed thicker cortex in regions implicated in both speech articulation and language planning and greater density in regions related to language planning (but not articulation). Volume was overall the least sensitive to AoA‐related differences but showed differences in one semantic processing area. The fact that cortical thickness, volume, and density results were each unique demonstrates the importance of considering multiple indices of brain structure in regards to the neural bases of bilingualism and helps explain some discrepancy among previous findings. Finally, this study highlights the importance of obtaining enough power to conduct whole‐brain analyses in place of region‐of‐interest analyses because it found results in a variety of areas that were not initially hypothesized.

## CONFLICT OF INTEREST

The authors declare no competing financial interests.

## Supporting information


**Appendix A** Regions where cortical thickness was significantly related to age of acquisition in Freesurfer

**Appendix B** Regions where gray matter density was significantly related to age of acquisition in SPM

**Appendix C** Regions where gray matter volume was significantly related to age of acquisition in Freesurfer

**Appendix D** Regions where gray matter volume was significantly related to age of acquisition in SPM

**Data S1**. Outline of proficiency measures included in each study

## Data Availability

The data that support the findings of this study are available from the corresponding author upon reasonable request.
