# Supplementary material for: Age of acquisition impacts the brain differently depending on neuroanatomical metric
Source: Hum Brain Mapp. 2019 Oct 10;41(2):484–502. doi: 10.1002/hbm.24817 (PMC7267963; doi:10.1002/hbm.24817)
Supplement: Supplementary file 1 — Appendix A Regions where cortical thickness was significantly related to age of acquisition in Freesurfer [file HBM-41-484-s001.docx]

Appendix A

*Regions where cortical thickness was significantly related to age of acquisition in Freesurfer*

Contents:

A.1 ANOVA: Early vs. late bilingual contrast…………………..………..………..……… Page 1

A.2 ANOVA: Monolingual vs. bilingual contrast …………………..………..…………… Page 2

**A.1 ANOVA: Early vs. late bilingual contrast**

*Contrast: Early bilingual vs. Late bilingual. “Max” indicates *t* value, where positive/negative sign indicates direction of relationship (positive: Early > Late; negative: Early < Late). VtxMax – Vertix number at the maximum. Coordinates are in MNI305 space. NVtxs – number of voxels in cluster. Annot – annotation of the brain region where significant cluster fell according to Desikan-Killiany atlas, a gyral-based atlas built into Freesurfer package. Note that formatting of Appendices A and B1 differ from that of B2 and C due to differences in SPM and Freesurfer outputs.

Left hemisphere

# Max VtxMax Size(mm^2) X Y Z NVtxs Annot

1 -4.685 150721 76.49 -18.3 -60.9 57.3 181 superiorparietal

2 -4.435 14722 96.38 -9.0 -24.4 67.3 221 precentral

3 -4.409 135662 114.93 -41.3 -74.4 19.3 202 inferiorparietal

4 -4.397 100789 185.79 -58.6 -18.8 2.1 429 superiortemporal

5 -4.071 160277 38.45 -33.7 4.0 -12.3 118 insula

6 -3.798 40965 42.40 -36.9 -16.9 56.0 100 precentral

7 -3.459 81338 2.46 -28.5 -51.6 60.0 6 superiorparietal

**A.2 ANOVA: Monolingual vs. bilingual contrast**

*Contrast: Monolingual vs. bilingual. “Max” indicates *t* value, where positive/negative sign indicates direction of relationship (positive: Monolingual > Bilingual; negative: Monolingual < Bilingual).

Left hemisphere

# Max VtxMax Size(mm^2) X Y Z NVtxs Annot

1 -6.732 3173 1232.65 -35.4 -79.3 24.9 1915 inferiorparietal

2 -6.463 27587 578.85 -41.1 -80.4 7.9 901 lateraloccipital

3 -5.488 84147 214.03 -4.3 37.2 -19.7 381 medialorbitofrontal

4 -4.700 90225 110.41 -42.0 -10.2 -33.7 174 inferiortemporal

5 -4.580 120875 135.71 -53.1 -26.0 -24.8 232 inferiortemporal

6 -4.531 43751 438.21 -18.6 -56.2 18.0 919 precuneus

7 4.368 110805 120.18 -31.8 28.6 -4.7 261 lateralorbitofrontal

8 -4.280 82160 115.47 -3.5 -29.9 61.0 271 paracentral

9 3.398 50192 58.53 -23.7 -32.3 54.9 125 postcentral

10 -3.317 37265 98.39 -6.6 14.2 57.4 213 superiorfrontal

11 -3.252 126504 86.51 -9.1 -83.1 31.8 109 superiorparietal

12 -3.069 61107 23.60 -33.6 6.7 0.6 49 insula

13 -3.051 58908 18.56 -5.2 12.0 25.9 49 caudalanteriorcingulate

14 -2.983 118570 12.78 -41.5 -7.1 52.0 32 precentral

15 -2.962 135189 14.29 -63.3 -21.2 3.0 44 superiortemporal

16 -2.938 130129 20.15 -56.6 -43.0 12.2 39 bankssts

17 2.886 6899 9.77 -6.1 -86.0 20.3 11 cuneus

18 -2.882 118163 5.79 -23.2 -20.1 -19.4 18 parahippocampal

19 -2.840 75732 6.59 -29.1 -56.9 -10.8 11 fusiform

20 2.782 58461 2.58 -38.1 42.7 17.9 4 rostralmiddlefrontal

Right hemisphere

# Max VtxMax Size(mm^2) TalX TalY TalZ NVtxs Annot

1 -8.018 41021 212.12 3.7 -29.1 60.7 544 paracentral

2 -5.296 135947 1083.27 29.7 -85.8 16.7 1588 lateraloccipital

3 5.143 160995 53.55 17.8 -37.8 -0.9 182 parahippocampal

4 -4.264 953 117.60 37.1 6.2 -5.3 258 insula

5 -4.247 55921 37.87 25.0 -20.6 -18.0 126 parahippocampal

6 -4.241 18805 275.26 6.9 -64.0 6.0 403 lingual

7 -3.847 97284 68.46 63.7 -29.6 4.0 164 superiortemporal

8 -3.840 33637 47.66 22.5 -55.7 21.6 93 precuneus

9 -3.836 32160 86.60 53.5 -27.4 -22.6 133 inferiortemporal

10 -3.816 58587 43.58 4.6 -3.3 34.5 107 posteriorcingulate

11 -3.722 57931 42.47 64.1 -19.9 -11.5 80 middletemporal

12 -3.600 118363 36.33 32.5 -34.3 -18.1 80 fusiform

13 -3.539 15453 34.61 43.8 -24.3 9.2 93 transversetemporal

14 -3.480 130192 108.35 19.7 -75.0 43.4 182 superiorparietal

15 -3.452 25533 63.99 61.2 -7.6 -3.1 128 superiortemporal

16 -3.277 31378 50.27 26.8 -71.4 23.9 79 superiorparietal

17 -3.224 57884 133.85 40.4 -58.4 -11.4 222 fusiform

18 -3.211 49302 112.00 49.4 -51.1 39.1 219 inferiorparietal

19 -3.203 46282 24.01 44.5 -8.0 -34.3 42 inferiortemporal

20 -3.158 104469 62.09 23.8 -79.4 32.3 76 superiorparietal

21 -3.117 141916 32.52 6.1 -79.8 35.1 64 cuneus

22 -3.083 87383 15.53 45.3 -50.8 17.5 41 inferiorparietal

23 -3.075 148849 26.14 36.2 -82.2 3.8 33 lateraloccipital

24 -3.058 123521 12.10 11.0 18.8 36.6 29 superiorfrontal

25 -2.972 157214 24.74 27.1 -55.3 4.5 61 lingual

26 2.882 161814 8.54 22.1 -93.8 -6.5 10 lateraloccipital

27 -2.830 125847 2.78 7.3 -80.3 18.3 3 cuneus
