# Supplementary material for: Age of acquisition impacts the brain differently depending on neuroanatomical metric
Source: Hum Brain Mapp. 2019 Oct 10;41(2):484–502. doi: 10.1002/hbm.24817 (PMC7267963; doi:10.1002/hbm.24817)
Supplement: Supplementary file 2 — Appendix B Regions where gray matter density was significantly related to age of acquisition in SPM [file HBM-41-484-s003.docx]

Appendix B

*Regions where gray matter density was significantly related to age of acquisition in SPM*

Contents:

B.1 ANOVA: Early vs. late bilingual contrast…………………………………………… Page 1

B.2 ANCOVA: Early vs. late bilingual contrast, controlling English proficiency………. Page 12

B.3 ANOVA: Monolingual vs. bilingual contrast …………………..…………………… Page 14

B.4 ANCOVA: Monolingual vs. bilingual contrast, controlling English proficiency ...... Page 127

**B.1 ANOVA: Early vs. late bilingual contrast**

*Contrast: Early vs. late bilingual. “Peak intensity” indicates *t* value at cluster peak, where positive/negative sign indicates direction of relationship (positive: Early bilingual > Late bilingual; negative: Early bilingual < Late bilingual). Results rendered using xjview toolbox. Note that formatting of Appendices A and B1 differ from that of B2 and C due to differences in SPM and Freesurfer outputs.

***Early < Late (negative direction relationship)***

Type: T

df: 324

Threshold

-- p value = 0.00021105

-- intensity = 3.5627

-- cluster size = 10

Number of clusters found: 29

----------------------

Cluster 1

Number of voxels: 40

Peak MNI coordinate: 10.5 -10.5 -31.5

Peak MNI coordinate region: // undefined // undefined // undefined // undefined // undefined // undefined

Peak intensity: -5.0286

# voxels structure

40 --TOTAL # VOXELS--

6 Right Brainstem

6 Pons

----------------------

Cluster 2

Number of voxels: 14

Peak MNI coordinate: -28.5 -82.5 -42

Peak MNI coordinate region: // Left Cerebellum // Cerebellum Posterior Lobe // Pyramis // undefined // undefined // Cerebelum_Crus2_L (aal)

Peak intensity: -4.6145

# voxels structure

14 --TOTAL # VOXELS--

14 Cerebelum_Crus2_L (aal)

14 Left Cerebellum

14 Cerebellum Posterior Lobe

11 Pyramis

3 Tuber

----------------------

Cluster 3

Number of voxels: 12

Peak MNI coordinate: 46.5 -40.5 -37.5

Peak MNI coordinate region: // Right Cerebellum // Cerebellum Anterior Lobe // Culmen // undefined // undefined // Cerebelum_Crus2_R (aal)

Peak intensity: -4.6952

# voxels structure

12 --TOTAL # VOXELS--

10 Culmen

10 Right Cerebellum

10 Cerebellum Anterior Lobe

8 Cerebelum_Crus1_R (aal)

4 Cerebelum_Crus2_R (aal)

----------------------

Cluster 4

Number of voxels: 301

Peak MNI coordinate: 10.5 -16.5 -22.5

Peak MNI coordinate region: // Right Brainstem // Midbrain // undefined // undefined // undefined // undefined

Peak intensity: -5.7932

# voxels structure

301 --TOTAL # VOXELS--

179 Pons

151 Left Brainstem

149 Right Brainstem

121 Midbrain

8 Gray Matter

6 Substania Nigra

2 Mammillary Body

----------------------

Cluster 5

Number of voxels: 12

Peak MNI coordinate: -9 -12 -33

Peak MNI coordinate region: // undefined // undefined // undefined // undefined // undefined // undefined

Peak intensity: -4.8228

# voxels structure

12 --TOTAL # VOXELS--

----------------------

Cluster 6

Number of voxels: 12

Peak MNI coordinate: 18 -6 -24

Peak MNI coordinate region: // Right Cerebrum // Limbic Lobe // Uncus // White Matter // undefined // ParaHippocampal_R (aal)

Peak intensity: -4.1404

# voxels structure

12 --TOTAL # VOXELS--

12 Limbic Lobe

12 ParaHippocampal_R (aal)

12 Right Cerebrum

10 Parahippocampa Gyrus

8 Gray Matter

4 brodmann area 34

4 brodmann area 28

3 White Matter

2 Uncus

----------------------

Cluster 7

Number of voxels: 10

Peak MNI coordinate: 19.5 0 -21

Peak MNI coordinate region: // Right Cerebrum // Limbic Lobe // Parahippocampa Gyrus // Gray Matter // Amygdala // ParaHippocampal_R (aal)

Peak intensity: -4.3801

# voxels structure

10 --TOTAL # VOXELS--

10 Limbic Lobe

10 ParaHippocampal_R (aal)

10 Right Cerebrum

8 Gray Matter

8 Amygdala

7 Parahippocampa Gyrus

3 Uncus

2 White Matter

----------------------

Cluster 8

Number of voxels: 42

Peak MNI coordinate: 6 16.5 -18

Peak MNI coordinate region: // Right Cerebrum // Frontal Lobe // Medial Frontal Gyrus // White Matter // undefined // Olfactory_R (aal)

Peak intensity: -5.4826

# voxels structure

42 --TOTAL # VOXELS--

42 Right Cerebrum

40 Frontal Lobe

30 White Matter

22 Olfactory_R (aal)

20 Rectus_R (aal)

17 Subcallosal Gyrus

14 Rectal Gyrus

12 Gray Matter

9 Medial Frontal Gyrus

7 brodmann area 25

5 brodmann area 11

2 Anterior Cingulate

2 Limbic Lobe

----------------------

Cluster 9

Number of voxels: 21

Peak MNI coordinate: -13.5 12 -22.5

Peak MNI coordinate region: // undefined // undefined // Inferior Frontal Gyrus // undefined // undefined // Frontal_Sup_Orb_L (aal)

Peak intensity: -4.7709

# voxels structure

21 --TOTAL # VOXELS--

12 Frontal_Inf_Orb_L (aal)

9 Left Cerebrum

9 Frontal Lobe

8 Inferior Frontal Gyrus

6 Frontal_Sup_Orb_L (aal)

4 brodmann area 47

4 Gray Matter

1 Olfactory_L (aal)

----------------------

Cluster 10

Number of voxels: 11

Peak MNI coordinate: 15 16.5 -22.5

Peak MNI coordinate region: // Right Cerebrum // Frontal Lobe // Inferior Frontal Gyrus // Gray Matter // brodmann area 47 // Frontal_Sup_Orb_R (aal)

Peak intensity: -4.855

# voxels structure

11 --TOTAL # VOXELS--

10 Frontal Lobe

10 Frontal_Sup_Orb_R (aal)

10 Inferior Frontal Gyrus

10 Right Cerebrum

8 brodmann area 47

8 Gray Matter

----------------------

Cluster 11

Number of voxels: 39

Peak MNI coordinate: -6 16.5 -22.5

Peak MNI coordinate region: // Left Cerebrum // Frontal Lobe // Rectal Gyrus // Gray Matter // brodmann area 11 // Rectus_L (aal)

Peak intensity: -4.9899

# voxels structure

39 --TOTAL # VOXELS--

39 Frontal Lobe

39 Left Cerebrum

27 White Matter

24 Rectus_L (aal)

16 Medial Frontal Gyrus

15 Olfactory_L (aal)

12 Rectal Gyrus

12 Gray Matter

11 Subcallosal Gyrus

9 brodmann area 11

3 brodmann area 25

----------------------

Cluster 12

Number of voxels: 18

Peak MNI coordinate: -22.5 -27 -18

Peak MNI coordinate region: // Left Cerebrum // Limbic Lobe // Parahippocampa Gyrus // Gray Matter // brodmann area 35 // ParaHippocampal_L (aal)

Peak intensity: -4.4663

# voxels structure

18 --TOTAL # VOXELS--

18 Left Cerebrum

18 Limbic Lobe

18 ParaHippocampal_L (aal)

18 Parahippocampa Gyrus

15 Gray Matter

14 brodmann area 35

1 brodmann area 36

1 White Matter

----------------------

Cluster 13

Number of voxels: 20

Peak MNI coordinate: 22.5 -30 -12

Peak MNI coordinate region: // Right Cerebrum // Limbic Lobe // Parahippocampa Gyrus // White Matter // undefined // ParaHippocampal_R (aal)

Peak intensity: -4.7583

# voxels structure

20 --TOTAL # VOXELS--

20 Limbic Lobe

20 Parahippocampa Gyrus

20 Right Cerebrum

19 ParaHippocampal_R (aal)

10 White Matter

9 Gray Matter

8 brodmann area 35

1 brodmann area 36

1 Lingual_R (aal)

----------------------

Cluster 14

Number of voxels: 47

Peak MNI coordinate: -1.5 -1.5 -16.5

Peak MNI coordinate region: // undefined // undefined // undefined // undefined // undefined // undefined

Peak intensity: -5.0122

# voxels structure

47 --TOTAL # VOXELS--

6 White Matter

3 Gray Matter

3 Hypothalamus

----------------------

Cluster 15

Number of voxels: 103

Peak MNI coordinate: 9 -18 -15

Peak MNI coordinate region: // Right Brainstem // Midbrain // undefined // undefined // undefined // undefined

Peak intensity: -5.575

# voxels structure

103 --TOTAL # VOXELS--

103 Right Brainstem

103 Midbrain

5 Substania Nigra

5 Gray Matter

----------------------

Cluster 16

Number of voxels: 45

Peak MNI coordinate: -15 -21 -10.5

Peak MNI coordinate region: // Left Brainstem // Midbrain // undefined // undefined // undefined // undefined

Peak intensity: -4.9658

# voxels structure

45 --TOTAL # VOXELS--

44 Midbrain

44 Left Brainstem

3 Substania Nigra

3 Gray Matter

----------------------

Cluster 17

Number of voxels: 38

Peak MNI coordinate: 34.5 10.5 -13.5

Peak MNI coordinate region: // Right Cerebrum // Frontal Lobe // Inferior Frontal Gyrus // Gray Matter // brodmann area 13 // undefined

Peak intensity: -5.1103

# voxels structure

38 --TOTAL # VOXELS--

38 Right Cerebrum

28 Insula_R (aal)

26 Inferior Frontal Gyrus

22 Frontal Lobe

21 Gray Matter

18 brodmann area 13

12 Extra-Nuclear

11 Sub-lobar

7 White Matter

5 Temporal Lobe

3 brodmann area 47

----------------------

Cluster 18

Number of voxels: 18

Peak MNI coordinate: 21 52.5 -3

Peak MNI coordinate region: // Right Cerebrum // Frontal Lobe // Superior Frontal Gyrus // White Matter // undefined // undefined

Peak intensity: -5.0589

# voxels structure

18 --TOTAL # VOXELS--

18 Right Cerebrum

18 White Matter

18 Frontal Lobe

14 Superior Frontal Gyrus

5 Frontal_Sup_Orb_R (aal)

4 Medial Frontal Gyrus

3 Frontal_Sup_R (aal)

----------------------

Cluster 19

Number of voxels: 26

Peak MNI coordinate: 6 36 7.5

Peak MNI coordinate region: // Right Cerebrum // Limbic Lobe // Anterior Cingulate // White Matter // undefined // Cingulum_Ant_R (aal)

Peak intensity: -5.9459

# voxels structure

26 --TOTAL # VOXELS--

26 Right Cerebrum

25 Limbic Lobe

25 Anterior Cingulate

25 White Matter

25 Cingulum_Ant_R (aal)

1 Sub-Gyral

1 Frontal Lobe

1 brodmann area 24

1 Gray Matter

----------------------

Cluster 20

Number of voxels: 14

Peak MNI coordinate: -13.5 61.5 9

Peak MNI coordinate region: // Left Cerebrum // Frontal Lobe // Superior Frontal Gyrus // White Matter // undefined // Frontal_Sup_Medial_L (aal)

Peak intensity: -4.2758

# voxels structure

14 --TOTAL # VOXELS--

14 Frontal Lobe

14 Left Cerebrum

14 White Matter

13 Superior Frontal Gyrus

9 Frontal_Sup_Medial_L (aal)

5 Frontal_Sup_L (aal)

1 Medial Frontal Gyrus

----------------------

Cluster 21

Number of voxels: 12

Peak MNI coordinate: 63 -54 22.5

Peak MNI coordinate region: // Right Cerebrum // Temporal Lobe // Supramarginal Gyrus // Gray Matter // brodmann area 40 // Temporal_Sup_R (aal)

Peak intensity: -3.9061

# voxels structure

12 --TOTAL # VOXELS--

12 Right Cerebrum

12 Temporal Lobe

10 Temporal_Sup_R (aal)

9 Gray Matter

7 Supramarginal Gyrus

5 brodmann area 22

5 Superior Temporal Gyrus

4 brodmann area 40

1 White Matter

1 Temporal_Mid_R (aal)

----------------------

Cluster 22

Number of voxels: 13

Peak MNI coordinate: 51 -73.5 22.5

Peak MNI coordinate region: // Right Cerebrum // Temporal Lobe // Middle Temporal Gyrus // White Matter // undefined // Temporal_Mid_R (aal)

Peak intensity: -4.5011

# voxels structure

13 --TOTAL # VOXELS--

13 Middle Temporal Gyrus

13 Right Cerebrum

13 Temporal Lobe

10 White Matter

8 Temporal_Mid_R (aal)

5 Occipital_Mid_R (aal)

3 brodmann area 39

3 Gray Matter

----------------------

Cluster 23

Number of voxels: 14

Peak MNI coordinate: -24 24 30

Peak MNI coordinate region: // Left Cerebrum // Frontal Lobe // Sub-Gyral // White Matter // undefined // undefined

Peak intensity: -4.448

# voxels structure

14 --TOTAL # VOXELS--

14 Left Cerebrum

14 Frontal Lobe

12 White Matter

8 Frontal_Mid_L (aal)

8 Sub-Gyral

6 Middle Frontal Gyrus

2 brodmann area 9

2 Gray Matter

----------------------

Cluster 24

Number of voxels: 30

Peak MNI coordinate: 61.5 -54 36

Peak MNI coordinate region: // Right Cerebrum // Parietal Lobe // Supramarginal Gyrus // Gray Matter // brodmann area 40 // Angular_R (aal)

Peak intensity: -4.2621

# voxels structure

30 --TOTAL # VOXELS--

28 Right Cerebrum

28 Parietal Lobe

18 brodmann area 40

18 Gray Matter

16 Supramarginal Gyrus

15 Parietal_Inf_R (aal)

12 Inferior Parietal Lobule

10 Angular_R (aal)

3 SupraMarginal_R (aal)

3 White Matter

----------------------

Cluster 25

Number of voxels: 12

Peak MNI coordinate: -33 -49.5 34.5

Peak MNI coordinate region: // Left Cerebrum // Parietal Lobe // Sub-Gyral // White Matter // undefined // Angular_L (aal)

Peak intensity: -4.2259

# voxels structure

12 --TOTAL # VOXELS--

12 Parietal Lobe

12 White Matter

12 Left Cerebrum

8 Angular_L (aal)

6 Sub-Gyral

6 Supramarginal Gyrus

1 Parietal_Inf_L (aal)

----------------------

Cluster 26

Number of voxels: 10

Peak MNI coordinate: -9 19.5 36

Peak MNI coordinate region: // Left Cerebrum // Limbic Lobe // Cingulate Gyrus // White Matter // undefined // Cingulum_Mid_L (aal)

Peak intensity: -4.4224

# voxels structure

10 --TOTAL # VOXELS--

10 Cingulate Gyrus

10 Left Cerebrum

8 White Matter

8 Cingulum_Mid_L (aal)

7 Limbic Lobe

3 Frontal Lobe

2 brodmann area 32

2 Gray Matter

----------------------

Cluster 27

Number of voxels: 13

Peak MNI coordinate: -19.5 -18 42

Peak MNI coordinate region: // Left Cerebrum // Limbic Lobe // Cingulate Gyrus // Gray Matter // brodmann area 24 // undefined

Peak intensity: -4.9125

# voxels structure

13 --TOTAL # VOXELS--

13 Left Cerebrum

12 Sub-Gyral

11 White Matter

11 Frontal Lobe

2 Limbic Lobe

2 brodmann area 24

2 Gray Matter

1 Cingulate Gyrus

----------------------

Cluster 28

Number of voxels: 11

Peak MNI coordinate: -36 -1.5 42

Peak MNI coordinate region: // Left Cerebrum // Frontal Lobe // Middle Frontal Gyrus // Gray Matter // brodmann area 6 // Precentral_L (aal)

Peak intensity: -3.9869

# voxels structure

11 --TOTAL # VOXELS--

11 Frontal Lobe

11 Left Cerebrum

9 Precentral_L (aal)

6 White Matter

5 Gray Matter

5 Middle Frontal Gyrus

5 brodmann area 6

5 Precentral Gyrus

1 Sub-Gyral

----------------------

Cluster 29

Number of voxels: 19

Peak MNI coordinate: 48 1.5 46.5

Peak MNI coordinate region: // Right Cerebrum // Frontal Lobe // Middle Frontal Gyrus // White Matter // undefined // Precentral_R (aal)

Peak intensity: -4.5329

# voxels structure

19 --TOTAL # VOXELS--

19 Frontal Lobe

19 Precentral_R (aal)

19 Right Cerebrum

18 Middle Frontal Gyrus

9 brodmann area 6

9 Gray Matter

4 White Matter

1 Precentral Gyrus

>>

**B.2 ANCOVA: Early vs. late bilingual contrast, controlling for English proficiency**

*Contrast: Early vs. late bilingual. “Peak intensity” indicates *t* value at cluster peak, where positive/negative sign indicates direction of relationship (positive: Early bilingual > Late bilingual; negative: Early bilingual < Late bilingual). Results rendered using xjview toolbox.

***Early < Late (negative direction relationship)***

Type: T

df: 323

Threshold

-- p value = 3.6107e-05

-- intensity = 4.0209

-- cluster size = 10

Number of clusters found: 7

----------------------

Cluster 1

Number of voxels: 10

Peak MNI coordinate: 3 -19.5 -27

Peak MNI coordinate region: // Right Brainstem // Pons // undefined // undefined // undefined // undefined

Peak intensity: -4.6157

# voxels structure

10 --TOTAL # VOXELS--

10 Right Brainstem

10 Pons

----------------------

Cluster 2

Number of voxels: 16

Peak MNI coordinate: 6 16.5 -18

Peak MNI coordinate region: // Right Cerebrum // Frontal Lobe // Medial Frontal Gyrus // White Matter // undefined // Olfactory_R (aal)

Peak intensity: -5.492

# voxels structure

16 --TOTAL # VOXELS--

16 Frontal Lobe

16 Right Cerebrum

14 White Matter

10 Rectus_R (aal)

7 Rectal Gyrus

6 Olfactory_R (aal)

6 Medial Frontal Gyrus

3 Subcallosal Gyrus

2 brodmann area 11

2 Gray Matter

----------------------

Cluster 3

Number of voxels: 19

Peak MNI coordinate: -6 15 -19.5

Peak MNI coordinate region: // Left Cerebrum // Frontal Lobe // Medial Frontal Gyrus // White Matter // undefined // Rectus_L (aal)

Peak intensity: -4.7293

# voxels structure

19 --TOTAL # VOXELS--

19 Frontal Lobe

19 Left Cerebrum

14 White Matter

12 Rectus_L (aal)

10 Medial Frontal Gyrus

7 Rectal Gyrus

7 Olfactory_L (aal)

5 brodmann area 11

5 Gray Matter

2 Subcallosal Gyrus

----------------------

Cluster 4

Number of voxels: 27

Peak MNI coordinate: 9 -18 -15

Peak MNI coordinate region: // Right Brainstem // Midbrain // undefined // undefined // undefined // undefined

Peak intensity: -5.4187

# voxels structure

27 --TOTAL # VOXELS--

27 Right Brainstem

27 Midbrain

1 Substania Nigra

1 Gray Matter

----------------------

Cluster 5

Number of voxels: 10

Peak MNI coordinate: -6 -19.5 -15

Peak MNI coordinate region: // Left Brainstem // Midbrain // undefined // undefined // undefined // undefined

Peak intensity: -4.8558

# voxels structure

10 --TOTAL # VOXELS--

10 Midbrain

10 Left Brainstem

----------------------

Cluster 6

Number of voxels: 11

Peak MNI coordinate: 34.5 10.5 -13.5

Peak MNI coordinate region: // Right Cerebrum // Frontal Lobe // Inferior Frontal Gyrus // Gray Matter // brodmann area 13 // undefined

Peak intensity: -4.6493

# voxels structure

11 --TOTAL # VOXELS--

11 Right Cerebrum

8 Inferior Frontal Gyrus

7 Insula_R (aal)

6 brodmann area 13

6 Gray Matter

4 Frontal Lobe

4 Temporal Lobe

3 White Matter

3 Sub-lobar

3 Extra-Nuclear

----------------------

Cluster 7

Number of voxels: 13

Peak MNI coordinate: 6 36 7.5

Peak MNI coordinate region: // Right Cerebrum // Limbic Lobe // Anterior Cingulate // White Matter // undefined // Cingulum_Ant_R (aal)

Peak intensity: -5.5391

# voxels structure

13 --TOTAL # VOXELS--

13 Anterior Cingulate

13 Cingulum_Ant_R (aal)

13 Limbic Lobe

13 Right Cerebrum

12 White Matter

1 brodmann area 24

1 Gray Matter

>>

**B.3 ANOVA: Monolingual vs. bilingual contrast**

*Contrast: Monolingual vs. bilingual. “Peak intensity” indicates *t* value at cluster peak, where positive/negative sign indicates direction of relationship (positive: Monolingual > bilingual; negative: Monolingual < bilingual). Results rendered using xjview toolbox.

***Monolingual < Bilingual***

Type: T

df: 324

Threshold

-- p value = 0.0047019

-- intensity = 2.6126

-- cluster size = 10

Number of clusters found: 281

----------------------

Cluster 1

Number of voxels: 17

Peak MNI coordinate: -18 -58.5 -66

Peak MNI coordinate region: // undefined // undefined // undefined // undefined // undefined // undefined

Peak intensity: -3.5873

# voxels structure

17 --TOTAL # VOXELS--

----------------------

Cluster 2

Number of voxels: 58

Peak MNI coordinate: 28.5 -70.5 -60

Peak MNI coordinate region: // undefined // undefined // undefined // undefined // undefined // Cerebelum_8_R (aal)

Peak intensity: -3.8272

# voxels structure

58 --TOTAL # VOXELS--

9 Cerebelum_8_R (aal)

----------------------

Cluster 3

Number of voxels: 429

Peak MNI coordinate: -7.5 -79.5 -52.5

Peak MNI coordinate region: // undefined // undefined // undefined // undefined // undefined // undefined

Peak intensity: -4.86

# voxels structure

429 --TOTAL # VOXELS--

40 Left Cerebellum

40 Cerebellum Posterior Lobe

27 Pyramis

24 Cerebelum_Crus2_L (aal)

12 Inferior Semi-Lunar Lobule

1 Tuber

----------------------

Cluster 4

Number of voxels: 25

Peak MNI coordinate: -36 -54 -63

Peak MNI coordinate region: // undefined // undefined // undefined // undefined // undefined // undefined

Peak intensity: -4.0715

# voxels structure

25 --TOTAL # VOXELS--

----------------------

Cluster 5

Number of voxels: 12

Peak MNI coordinate: -18 -70.5 -60

Peak MNI coordinate region: // undefined // undefined // undefined // undefined // undefined // undefined

Peak intensity: -4.2149

# voxels structure

12 --TOTAL # VOXELS--

----------------------

Cluster 6

Number of voxels: 14

Peak MNI coordinate: 27 -45 -60

Peak MNI coordinate region: // undefined // undefined // undefined // undefined // undefined // undefined

Peak intensity: -4.0482

# voxels structure

14 --TOTAL # VOXELS--

2 Cerebelum_8_R (aal)

----------------------

Cluster 7

Number of voxels: 55

Peak MNI coordinate: -22.5 -43.5 -60

Peak MNI coordinate region: // undefined // undefined // undefined // undefined // undefined // undefined

Peak intensity: -4.2031

# voxels structure

55 --TOTAL # VOXELS--

32 Cerebelum_8_L (aal)

16 Cerebellar Tonsil

16 Left Cerebellum

16 Cerebellum Posterior Lobe

----------------------

Cluster 8

Number of voxels: 67

Peak MNI coordinate: -4.5 -43.5 -51

Peak MNI coordinate region: // Left Brainstem // Medulla // undefined // undefined // undefined // undefined

Peak intensity: -4.1734

# voxels structure

67 --TOTAL # VOXELS--

46 Medulla

46 Left Brainstem

20 Cerebelum_9_L (aal)

4 Left Cerebellum

4 Cerebellar Tonsil

4 Cerebellum Posterior Lobe

----------------------

Cluster 9

Number of voxels: 14

Peak MNI coordinate: 1.5 -33 -58.5

Peak MNI coordinate region: // undefined // undefined // undefined // undefined // undefined // undefined

Peak intensity: -3.976

# voxels structure

14 --TOTAL # VOXELS--

----------------------

Cluster 10

Number of voxels: 457

Peak MNI coordinate: 19.5 -90 -42

Peak MNI coordinate region: // undefined // undefined // undefined // undefined // undefined // undefined

Peak intensity: -5.7571

# voxels structure

457 --TOTAL # VOXELS--

186 Cerebelum_Crus2_R (aal)

95 Right Cerebellum

94 Cerebellum Posterior Lobe

52 Pyramis

25 Tuber

10 Uvula

7 Inferior Semi-Lunar Lobule

7 Cerebelum_7b_R (aal)

----------------------

Cluster 11

Number of voxels: 65

Peak MNI coordinate: 34.5 -37.5 -46.5

Peak MNI coordinate region: // Right Cerebellum // Cerebellum Posterior Lobe // Cerebellar Tonsil // undefined // undefined // Cerebelum_8_R (aal)

Peak intensity: -4.5083

# voxels structure

65 --TOTAL # VOXELS--

60 Cerebellum Posterior Lobe

60 Right Cerebellum

60 Cerebellar Tonsil

38 Cerebelum_8_R (aal)

5 Cerebelum_10_R (aal)

4 Cerebelum_7b_R (aal)

----------------------

Cluster 12

Number of voxels: 29

Peak MNI coordinate: -25.5 -61.5 -51

Peak MNI coordinate region: // Left Cerebellum // Cerebellum Posterior Lobe // Inferior Semi-Lunar Lobule // undefined // undefined // Cerebelum_8_L (aal)

Peak intensity: -4.2779

# voxels structure

29 --TOTAL # VOXELS--

29 Cerebelum_8_L (aal)

29 Left Cerebellum

29 Cerebellum Posterior Lobe

21 Inferior Semi-Lunar Lobule

8 Cerebellar Tonsil

----------------------

Cluster 13

Number of voxels: 23

Peak MNI coordinate: 13.5 -58.5 -52.5

Peak MNI coordinate region: // Right Cerebellum // Cerebellum Posterior Lobe // Cerebellar Tonsil // undefined // undefined // Cerebelum_9_R (aal)

Peak intensity: -3.7264

# voxels structure

23 --TOTAL # VOXELS--

23 Right Cerebellum

23 Cerebellum Posterior Lobe

19 Cerebelum_8_R (aal)

15 Cerebellar Tonsil

8 Inferior Semi-Lunar Lobule

4 Cerebelum_9_R (aal)

----------------------

Cluster 14

Number of voxels: 14

Peak MNI coordinate: -18 -51 -49.5

Peak MNI coordinate region: // Left Cerebellum // Cerebellum Posterior Lobe // Cerebellar Tonsil // undefined // undefined // Cerebelum_9_L (aal)

Peak intensity: -3.5969

# voxels structure

14 --TOTAL # VOXELS--

13 Cerebellum Posterior Lobe

13 Left Cerebellum

13 Cerebellar Tonsil

10 Cerebelum_9_L (aal)

4 Cerebelum_8_L (aal)

----------------------

Cluster 15

Number of voxels: 61

Peak MNI coordinate: -19.5 -46.5 -43.5

Peak MNI coordinate region: // Left Cerebellum // Cerebellum Posterior Lobe // Cerebellar Tonsil // undefined // undefined // Cerebelum_9_L (aal)

Peak intensity: -4.9012

# voxels structure

61 --TOTAL # VOXELS--

60 Cerebellum Posterior Lobe

60 Left Cerebellum

60 Cerebellar Tonsil

37 Cerebelum_8_L (aal)

16 Cerebelum_9_L (aal)

----------------------

Cluster 16

Number of voxels: 162

Peak MNI coordinate: 24 -54 -45

Peak MNI coordinate region: // Right Cerebellum // Cerebellum Posterior Lobe // Cerebellar Tonsil // undefined // undefined // Cerebelum_8_R (aal)

Peak intensity: -4.6105

# voxels structure

162 --TOTAL # VOXELS--

162 Right Cerebellum

162 Cerebellum Posterior Lobe

118 Cerebellar Tonsil

95 Cerebelum_8_R (aal)

27 Cerebelum_Crus1_R (aal)

24 Cerebelum_7b_R (aal)

15 Inferior Semi-Lunar Lobule

15 Pyramis

9 Tuber

5 Uvula

----------------------

Cluster 17

Number of voxels: 35941

Peak MNI coordinate: -46.5 18 -36

Peak MNI coordinate region: // undefined // undefined // undefined // undefined // undefined // Temporal_Pole_Mid_L (aal)

Peak intensity: -7.4613

# voxels structure

35941 --TOTAL # VOXELS--

20222 White Matter

15763 Right Cerebrum

15195 Left Cerebrum

13995 Frontal Lobe

8332 Gray Matter

8188 Sub-Gyral

6502 Sub-lobar

4547 Temporal Lobe

3580 Extra-Nuclear

2727 Limbic Lobe

2684 Inferior Frontal Gyrus

2542 Middle Frontal Gyrus

1859 Parietal Lobe

1853 Cingulate Gyrus

1773 Superior Temporal Gyrus

1208 Occipital Lobe

1149 Frontal_Mid_R (aal)

1049 Frontal_Mid_L (aal)

988 Superior Frontal Gyrus

985 Precentral Gyrus

944 Cerebro-Spinal Fluid

942 Lentiform Nucleus

926 Lateral Ventricle

924 Medial Frontal Gyrus

884 Corpus Callosum

839 Middle Temporal Gyrus

777 Frontal_Inf_Tri_L (aal)

776 Putamen

771 Midbrain

739 brodmann area 38

683 Frontal_Inf_Tri_R (aal)

653 Right Brainstem

640 Thalamus

635 Frontal_Inf_Orb_R (aal)

625 Left Brainstem

623 Temporal_Pole_Mid_R (aal)

614 Temporal_Pole_Sup_R (aal)

612 Precentral_R (aal)

592 Putamen_R (aal)

563 brodmann area 6

558 Temporal_Inf_R (aal)

542 Frontal_Inf_Orb_L (aal)

535 brodmann area 47

530 Frontal_Sup_L (aal)

526 brodmann area 46

514 brodmann area 10

514 Temporal_Sup_R (aal)

511 brodmann area 9

507 brodmann area 8

495 Pons

466 Anterior Cingulate

420 Precuneus

410 Temporal_Pole_Sup_L (aal)

395 Temporal_Mid_R (aal)

388 Putamen_L (aal)

375 Calcarine_R (aal)

363 Cuneus

350 Posterior Cingulate

338 Thalamus_L (aal)

330 Postcentral_R (aal)

323 Rectus_L (aal)

313 brodmann area 21

313 Cingulum_Mid_R (aal)

313 brodmann area 45

303 Postcentral Gyrus

297 Middle Occipital Gyrus

296 Thalamus_R (aal)

258 Cingulum_Mid_L (aal)

251 Temporal_Pole_Mid_L (aal)

246 Calcarine_L (aal)

241 Frontal_Sup_R (aal)

236 Occipital_Mid_L (aal)

229 Caudate_R (aal)

215 Pulvinar

215 brodmann area 24

210 Temporal_Mid_L (aal)

206 Supp_Motor_Area_L (aal)

198 Rectus_R (aal)

195 brodmann area 32

186 Cingulum_Ant_R (aal)

183 Inferior Parietal Lobule

181 Lingual Gyrus

181 Paracentral_Lobule_L (aal)

174 brodmann area 22

173 Frontal_Sup_Medial_L (aal)

171 Hippocampus_R (aal)

171 Caudate

167 Lingual_R (aal)

159 Inferior Temporal Gyrus

156 Insula

154 Frontal_Sup_Orb_L (aal)

152 brodmann area 30

151 Supp_Motor_Area_R (aal)

144 Cerebellum Anterior Lobe

130 brodmann area 11

128 Lingual_L (aal)

128 Frontal_Mid_Orb_R (aal)

126 Postcentral_L (aal)

126 Precentral_L (aal)

123 Pallidum_R (aal)

123 Lateral Globus Pallidus

121 Rectal Gyrus

120 Frontal_Sup_Orb_R (aal)

118 Precuneus_L (aal)

118 Occipital_Sup_R (aal)

112 Paracentral Lobule

110 brodmann area 20

109 Supramarginal Gyrus

108 Angular_R (aal)

107 Ventral Lateral Nucleus

106 Hippocampus_L (aal)

105 Parietal_Sup_L (aal)

104 Caudate Tail

103 Frontal_Inf_Oper_R (aal)

101 Cingulum_Post_L (aal)

101 Precuneus_R (aal)

99 Right Cerebellum

97 Pallidum_L (aal)

95 brodmann area 7

94 Occipital_Inf_L (aal)

94 Fusiform_R (aal)

94 Temporal_Inf_L (aal)

91 Parahippocampa Gyrus

88 Inter-Hemispheric

88 Occipital_Sup_L (aal)

87 Temporal_Sup_L (aal)

81 Transverse Temporal Gyrus

77 Frontal_Med_Orb_L (aal)

76 Fusiform Gyrus

74 brodmann area 19

74 Insula_L (aal)

69 brodmann area 18

68 brodmann area 3

68 Caudate_L (aal)

67 Claustrum

67 Parietal_Inf_L (aal)

65 Left Cerebellum

64 Culmen

56 Medial Dorsal Nucleus

55 Frontal_Mid_Orb_L (aal)

55 Subthalamic Nucleus

53 Substania Nigra

53 Cingulum_Ant_L (aal)

53 Cingulum_Post_R (aal)

53 Subcallosal Gyrus

52 Lateral Posterior Nucleus

49 Fusiform_L (aal)

49 brodmann area 31

47 Caudate Head

47 brodmann area 39

44 brodmann area 23

44 brodmann area 41

42 Medial Globus Pallidus

42 Insula_R (aal)

41 brodmann area 13

37 Inferior Occipital Gyrus

37 brodmann area 2

36 Cuneus_L (aal)

35 Cerebelum_6_R (aal)

33 Uncus

33 Rolandic_Oper_R (aal)

33 brodmann area 4

32 Orbital Gyrus

30 ParaHippocampal_R (aal)

30 brodmann area 25

29 Vermis_10 (aal)

29 Ventral Posterior Lateral Nucleus

27 brodmann area 42

26 Vermis_1_2 (aal)

25 brodmann area 5

23 Cuneus_R (aal)

23 Ventral Posterior Medial Nucleus

23 brodmann area 44

23 Hippocampus

21 Superior Parietal Lobule

20 Cerebelum_9_R (aal)

20 Caudate Body

17 Red Nucleus

17 SupraMarginal_R (aal)

16 Fourth Ventricle

16 Cerebelum_9_L (aal)

16 brodmann area 37

16 brodmann area 40

15 Frontal_Inf_Oper_L (aal)

15 Heschl_R (aal)

14 Cerebelum_4_5_R (aal)

13 Nodule

13 Medulla

12 brodmann area 43

11 Vermis_3 (aal)

10 Hypothalamus

10 Medial Geniculum Body

9 Ventral Anterior Nucleus

8 Occipital_Mid_R (aal)

8 SupraMarginal_L (aal)

7 ParaHippocampal_L (aal)

6 Heschl_L (aal)

6 Paracentral_Lobule_R (aal)

6 Mammillary Body

6 Amygdala_R (aal)

5 brodmann area 17

5 Amygdala_L (aal)

4 brodmann area 33

4 brodmann area 36

4 Cerebellar Tonsil

4 Cerebellum Posterior Lobe

4 Olfactory_R (aal)

4 Optic Tract

4 Cerebelum_3_L (aal)

4 Lateral Geniculum Body

4 brodmann area 1

4 Cerebelum_4_5_L (aal)

3 Olfactory_L (aal)

2 Anterior Commissure

2 brodmann area 27

2 brodmann area 29

1 Angular Gyrus

1 Angular_L (aal)

1 Frontal_Sup_Medial_R (aal)

----------------------

Cluster 18

Number of voxels: 193

Peak MNI coordinate: -9 -64.5 -30

Peak MNI coordinate region: // Left Cerebellum // Cerebellum Posterior Lobe // Declive // undefined // undefined // undefined

Peak intensity: -4.9443

# voxels structure

193 --TOTAL # VOXELS--

185 Left Cerebellum

114 Cerebellum Posterior Lobe

79 Cerebellum Anterior Lobe

52 Cerebelum_8_L (aal)

44 Pyramis

28 Cerebelum_7b_L (aal)

27 Inferior Semi-Lunar Lobule

24 Fastigium

20 Declive

20 Culmen

19 Uvula

19 Cerebelum_Crus2_L (aal)

11 Dentate

8 Nodule

8 Vermis_7 (aal)

8 Right Cerebellum

5 Declive of Vermis

3 Cerebelum_4_5_L (aal)

3 Vermis_8 (aal)

3 Vermis_6 (aal)

2 Cerebelum_9_L (aal)

2 Vermis_4_5 (aal)

1 Vermis_9 (aal)

1 Cerebelum_6_L (aal)

----------------------

Cluster 19

Number of voxels: 42

Peak MNI coordinate: -30 -63 -43.5

Peak MNI coordinate region: // Left Cerebellum // Cerebellum Posterior Lobe // Cerebellar Tonsil // undefined // undefined // Cerebelum_7b_L (aal)

Peak intensity: -3.8182

# voxels structure

42 --TOTAL # VOXELS--

42 Left Cerebellum

42 Cerebellum Posterior Lobe

23 Cerebellar Tonsil

19 Inferior Semi-Lunar Lobule

19 Cerebelum_7b_L (aal)

19 Cerebelum_8_L (aal)

4 Cerebelum_Crus2_L (aal)

----------------------

Cluster 20

Number of voxels: 41

Peak MNI coordinate: -31.5 -16.5 -39

Peak MNI coordinate region: // undefined // undefined // undefined // undefined // undefined // undefined

Peak intensity: -4.1022

# voxels structure

41 --TOTAL # VOXELS--

10 Fusiform_L (aal)

3 ParaHippocampal_L (aal)

1 Limbic Lobe

1 Gray Matter

1 Uncus

1 brodmann area 28

1 Left Cerebrum

----------------------

Cluster 21

Number of voxels: 26

Peak MNI coordinate: -48 -4.5 -46.5

Peak MNI coordinate region: // undefined // undefined // undefined // undefined // undefined // undefined

Peak intensity: -3.4781

# voxels structure

26 --TOTAL # VOXELS--

7 Temporal_Inf_L (aal)

5 Temporal Lobe

5 Inferior Temporal Gyrus

5 Left Cerebrum

3 brodmann area 20

3 Gray Matter

----------------------

Cluster 22

Number of voxels: 19

Peak MNI coordinate: -27 -67.5 -42

Peak MNI coordinate region: // Left Cerebellum // Cerebellum Posterior Lobe // Pyramis // undefined // undefined // Cerebelum_7b_L (aal)

Peak intensity: -3.8394

# voxels structure

19 --TOTAL # VOXELS--

19 Left Cerebellum

19 Cerebellum Posterior Lobe

15 Cerebelum_Crus2_L (aal)

12 Pyramis

6 Inferior Semi-Lunar Lobule

3 Cerebelum_7b_L (aal)

1 Cerebelum_8_L (aal)

1 Cerebellar Tonsil

----------------------

Cluster 23

Number of voxels: 17

Peak MNI coordinate: 15 -51 -43.5

Peak MNI coordinate region: // Right Cerebellum // Cerebellum Posterior Lobe // Cerebellar Tonsil // undefined // undefined // Cerebelum_9_R (aal)

Peak intensity: -4.854

# voxels structure

17 --TOTAL # VOXELS--

17 Cerebellum Posterior Lobe

17 Right Cerebellum

17 Cerebellar Tonsil

12 Cerebelum_9_R (aal)

3 Cerebelum_8_R (aal)

----------------------

Cluster 24

Number of voxels: 119

Peak MNI coordinate: -24 -81 -37.5

Peak MNI coordinate region: // Left Cerebellum // Cerebellum Posterior Lobe // Tuber // undefined // undefined // Cerebelum_Crus2_L (aal)

Peak intensity: -4.1309

# voxels structure

119 --TOTAL # VOXELS--

119 Left Cerebellum

116 Cerebellum Posterior Lobe

46 Pyramis

44 Cerebelum_Crus1_L (aal)

42 Cerebelum_Crus2_L (aal)

31 Tuber

25 Uvula

19 Cerebelum_8_L (aal)

14 Cerebellar Tonsil

6 Cerebelum_7b_L (aal)

3 Cerebellum Anterior Lobe

----------------------

Cluster 25

Number of voxels: 13

Peak MNI coordinate: -18 -39 -42

Peak MNI coordinate region: // undefined // undefined // undefined // undefined // undefined // Cerebelum_10_L (aal)

Peak intensity: -4.3236

# voxels structure

13 --TOTAL # VOXELS--

10 Cerebellum Posterior Lobe

10 Left Cerebellum

10 Cerebellar Tonsil

5 Cerebelum_10_L (aal)

2 Pons

2 Left Brainstem

----------------------

Cluster 26

Number of voxels: 18

Peak MNI coordinate: 46.5 -58.5 -42

Peak MNI coordinate region: // Right Cerebellum // Cerebellum Posterior Lobe // Cerebellar Tonsil // undefined // undefined // Cerebelum_Crus2_R (aal)

Peak intensity: -3.7047

# voxels structure

18 --TOTAL # VOXELS--

18 Cerebellum Posterior Lobe

18 Right Cerebellum

18 Cerebellar Tonsil

9 Cerebelum_Crus1_R (aal)

9 Cerebelum_Crus2_R (aal)

----------------------

Cluster 27

Number of voxels: 22

Peak MNI coordinate: 18 -37.5 -40.5

Peak MNI coordinate region: // Right Brainstem // Pons // undefined // undefined // undefined // undefined

Peak intensity: -3.8101

# voxels structure

22 --TOTAL # VOXELS--

10 Right Brainstem

10 Pons

9 Right Cerebellum

6 Cerebellar Tonsil

6 Cerebellum Posterior Lobe

5 Cerebelum_10_R (aal)

3 Cerebellum Anterior Lobe

1 Cerebelum_9_R (aal)

----------------------

Cluster 28

Number of voxels: 282

Peak MNI coordinate: -31.5 -27 -31.5

Peak MNI coordinate region: // undefined // undefined // undefined // undefined // undefined // Cerebelum_4_5_L (aal)

Peak intensity: -5.0957

# voxels structure

282 --TOTAL # VOXELS--

78 Left Cerebellum

68 Cerebellum Anterior Lobe

68 Culmen

63 Left Cerebrum

52 Temporal_Inf_L (aal)

49 Temporal Lobe

46 Cerebelum_4_5_L (aal)

46 Cerebelum_6_L (aal)

45 Fusiform Gyrus

33 Gray Matter

28 brodmann area 20

23 White Matter

13 Parahippocampa Gyrus

13 Fusiform_L (aal)

13 Limbic Lobe

10 Cerebellum Posterior Lobe

10 Cerebellar Tonsil

7 Cerebelum_10_L (aal)

5 brodmann area 36

4 Sub-Gyral

----------------------

Cluster 29

Number of voxels: 50

Peak MNI coordinate: 48 -9 -36

Peak MNI coordinate region: // Right Cerebrum // Temporal Lobe // Inferior Temporal Gyrus // Gray Matter // brodmann area 20 // Temporal_Inf_R (aal)

Peak intensity: -3.9278

# voxels structure

50 --TOTAL # VOXELS--

50 Temporal_Inf_R (aal)

48 Temporal Lobe

48 Right Cerebrum

34 Gray Matter

33 Inferior Temporal Gyrus

26 brodmann area 20

14 White Matter

12 Middle Temporal Gyrus

8 brodmann area 21

3 Fusiform Gyrus

----------------------

Cluster 30

Number of voxels: 10

Peak MNI coordinate: -3 -67.5 -40.5

Peak MNI coordinate region: // Left Cerebellum // Cerebellum Posterior Lobe // Uvula // undefined // undefined // Cerebelum_8_L (aal)

Peak intensity: -3.9868

# voxels structure

10 --TOTAL # VOXELS--

10 Cerebelum_8_L (aal)

10 Left Cerebellum

10 Uvula

10 Cerebellum Posterior Lobe

----------------------

Cluster 31

Number of voxels: 29

Peak MNI coordinate: -45 -61.5 -39

Peak MNI coordinate region: // Left Cerebellum // Cerebellum Posterior Lobe // Cerebellar Tonsil // undefined // undefined // Cerebelum_Crus2_L (aal)

Peak intensity: -3.6334

# voxels structure

29 --TOTAL # VOXELS--

29 Left Cerebellum

29 Cerebellum Posterior Lobe

18 Tuber

18 Cerebelum_Crus2_L (aal)

11 Cerebellar Tonsil

11 Cerebelum_Crus1_L (aal)

----------------------

Cluster 32

Number of voxels: 34

Peak MNI coordinate: 13.5 -75 -36

Peak MNI coordinate region: // Right Cerebellum // Cerebellum Posterior Lobe // Pyramis // undefined // undefined // Cerebelum_Crus2_R (aal)

Peak intensity: -4.6544

# voxels structure

34 --TOTAL # VOXELS--

34 Right Cerebellum

34 Cerebellum Posterior Lobe

30 Cerebelum_Crus2_R (aal)

26 Pyramis

8 Uvula

4 Cerebelum_8_R (aal)

----------------------

Cluster 33

Number of voxels: 19

Peak MNI coordinate: -16.5 -37.5 -34.5

Peak MNI coordinate region: // Left Brainstem // Pons // undefined // undefined // undefined // undefined

Peak intensity: -4.3297

# voxels structure

19 --TOTAL # VOXELS--

18 Pons

18 Left Brainstem

1 Cerebellum Anterior Lobe

1 Left Cerebellum

----------------------

Cluster 34

Number of voxels: 10

Peak MNI coordinate: 15 -30 -34.5

Peak MNI coordinate region: // Right Brainstem // Pons // undefined // undefined // undefined // undefined

Peak intensity: -3.7624

# voxels structure

10 --TOTAL # VOXELS--

10 Right Brainstem

10 Pons

----------------------

Cluster 35

Number of voxels: 66

Peak MNI coordinate: -25.5 4.5 -37.5

Peak MNI coordinate region: // Left Cerebrum // Temporal Lobe // Sub-Gyral // White Matter // undefined // Temporal_Pole_Mid_L (aal)

Peak intensity: -3.9423

# voxels structure

66 --TOTAL # VOXELS--

66 Left Cerebrum

65 White Matter

58 Temporal_Pole_Mid_L (aal)

54 Temporal Lobe

49 Superior Temporal Gyrus

12 Limbic Lobe

12 Uncus

6 Temporal_Pole_Sup_L (aal)

5 Sub-Gyral

2 Temporal_Inf_L (aal)

1 brodmann area 38

1 Gray Matter

----------------------

Cluster 36

Number of voxels: 14

Peak MNI coordinate: -24 18 -37.5

Peak MNI coordinate region: // Left Cerebrum // Temporal Lobe // Superior Temporal Gyrus // undefined // undefined // Temporal_Pole_Mid_L (aal)

Peak intensity: -3.8462

# voxels structure

14 --TOTAL # VOXELS--

12 Superior Temporal Gyrus

12 Temporal Lobe

12 Left Cerebrum

11 Temporal_Pole_Mid_L (aal)

10 brodmann area 38

10 Gray Matter

2 Temporal_Pole_Sup_L (aal)

----------------------

Cluster 37

Number of voxels: 32

Peak MNI coordinate: 15 -42 -33

Peak MNI coordinate region: // Right Cerebellum // Cerebellum Anterior Lobe // undefined // undefined // undefined // undefined

Peak intensity: -4.8527

# voxels structure

32 --TOTAL # VOXELS--

31 Right Cerebellum

31 Cerebellum Anterior Lobe

6 Culmen

2 Cerebelum_4_5_R (aal)

1 Right Brainstem

1 Dentate

1 Pons

----------------------

Cluster 38

Number of voxels: 10

Peak MNI coordinate: -37.5 -4.5 -34.5

Peak MNI coordinate region: // Left Cerebrum // Temporal Lobe // Middle Temporal Gyrus // White Matter // undefined // Temporal_Inf_L (aal)

Peak intensity: -3.4879

# voxels structure

10 --TOTAL # VOXELS--

10 Left Cerebrum

10 Temporal_Inf_L (aal)

10 White Matter

8 Temporal Lobe

6 Middle Temporal Gyrus

2 Uncus

2 Limbic Lobe

2 Sub-Gyral

----------------------

Cluster 39

Number of voxels: 22

Peak MNI coordinate: 9 -67.5 -31.5

Peak MNI coordinate region: // Right Cerebellum // Cerebellum Posterior Lobe // Pyramis // undefined // undefined // Cerebelum_8_R (aal)

Peak intensity: -3.9882

# voxels structure

22 --TOTAL # VOXELS--

22 Right Cerebellum

13 Cerebellum Posterior Lobe

13 Cerebelum_8_R (aal)

9 Cerebellum Anterior Lobe

8 Pyramis

7 Nodule

6 Declive

4 Vermis_8 (aal)

3 Vermis_7 (aal)

1 Uvula

1 Cerebelum_Crus2_R (aal)

----------------------

Cluster 40

Number of voxels: 40

Peak MNI coordinate: -15 -57 -28.5

Peak MNI coordinate region: // Left Cerebellum // Cerebellum Anterior Lobe // undefined // undefined // Dentate // undefined

Peak intensity: -3.9614

# voxels structure

40 --TOTAL # VOXELS--

40 Left Cerebellum

40 Cerebellum Anterior Lobe

34 Dentate

3 Cerebelum_6_L (aal)

1 Culmen

----------------------

Cluster 41

Number of voxels: 10

Peak MNI coordinate: 34.5 3 -34.5

Peak MNI coordinate region: // Right Cerebrum // Temporal Lobe // Sub-Gyral // White Matter // undefined // Temporal_Pole_Mid_R (aal)

Peak intensity: -3.1904

# voxels structure

10 --TOTAL # VOXELS--

10 Right Cerebrum

10 White Matter

9 Sub-Gyral

9 Temporal Lobe

5 Fusiform_R (aal)

5 Temporal_Pole_Mid_R (aal)

1 Limbic Lobe

1 Uncus

----------------------

Cluster 42

Number of voxels: 29

Peak MNI coordinate: 34.5 -73.5 -33

Peak MNI coordinate region: // Right Cerebellum // Cerebellum Posterior Lobe // Uvula // undefined // undefined // Cerebelum_Crus1_R (aal)

Peak intensity: -4.1809

# voxels structure

29 --TOTAL # VOXELS--

29 Cerebelum_Crus1_R (aal)

29 Right Cerebellum

29 Cerebellum Posterior Lobe

17 Uvula

12 Tuber

----------------------

Cluster 43

Number of voxels: 18

Peak MNI coordinate: -1.5 -66 -31.5

Peak MNI coordinate region: // Left Cerebellum // Cerebellum Anterior Lobe // Pyramis // undefined // undefined // Vermis_8 (aal)

Peak intensity: -3.5932

# voxels structure

18 --TOTAL # VOXELS--

15 Left Cerebellum

15 Cerebellum Posterior Lobe

12 Vermis_8 (aal)

10 Tuber of Vermis

7 Pyramis

4 Cerebelum_8_L (aal)

3 Right Cerebellum

3 Cerebellum Anterior Lobe

2 Cerebelum_Crus2_L (aal)

1 Declive of Vermis

----------------------

Cluster 44

Number of voxels: 23

Peak MNI coordinate: -28.5 -52.5 -30

Peak MNI coordinate region: // Left Cerebellum // Cerebellum Anterior Lobe // Culmen // undefined // undefined // Cerebelum_6_L (aal)

Peak intensity: -3.8121

# voxels structure

23 --TOTAL # VOXELS--

23 Cerebelum_6_L (aal)

23 Culmen

23 Left Cerebellum

23 Cerebellum Anterior Lobe

----------------------

Cluster 45

Number of voxels: 18

Peak MNI coordinate: -4.5 -12 -30

Peak MNI coordinate region: // Left Brainstem // Pons // undefined // undefined // undefined // undefined

Peak intensity: -3.4702

# voxels structure

18 --TOTAL # VOXELS--

13 Pons

13 Left Brainstem

----------------------

Cluster 46

Number of voxels: 34

Peak MNI coordinate: 19.5 1.5 -31.5

Peak MNI coordinate region: // Right Cerebrum // Limbic Lobe // Uncus // Gray Matter // brodmann area 28 // ParaHippocampal_R (aal)

Peak intensity: -4.5758

# voxels structure

34 --TOTAL # VOXELS--

34 Limbic Lobe

34 Right Cerebrum

33 Uncus

24 Gray Matter

21 ParaHippocampal_R (aal)

14 brodmann area 28

8 brodmann area 34

7 White Matter

1 Amygdala

1 brodmann area 36

1 Parahippocampa Gyrus

----------------------

Cluster 47

Number of voxels: 13

Peak MNI coordinate: -37.5 -78 -31.5

Peak MNI coordinate region: // Left Cerebellum // Cerebellum Posterior Lobe // Tuber // undefined // undefined // Cerebelum_Crus1_L (aal)

Peak intensity: -3.8983

# voxels structure

13 --TOTAL # VOXELS--

13 Cerebelum_Crus1_L (aal)

13 Left Cerebellum

13 Cerebellum Posterior Lobe

10 Tuber

2 Uvula

1 Declive

----------------------

Cluster 48

Number of voxels: 77

Peak MNI coordinate: 18 -4.5 -18

Peak MNI coordinate region: // Right Cerebrum // Limbic Lobe // Parahippocampa Gyrus // Gray Matter // Amygdala // ParaHippocampal_R (aal)

Peak intensity: -4.7066

# voxels structure

77 --TOTAL # VOXELS--

75 Right Cerebrum

74 Limbic Lobe

59 Parahippocampa Gyrus

59 ParaHippocampal_R (aal)

57 Gray Matter

30 brodmann area 34

14 Uncus

12 Amygdala

12 White Matter

11 brodmann area 28

10 Hippocampus_R (aal)

4 brodmann area 35

----------------------

Cluster 49

Number of voxels: 138

Peak MNI coordinate: -19.5 3 -30

Peak MNI coordinate region: // Left Cerebrum // Limbic Lobe // Uncus // Gray Matter // brodmann area 28 // ParaHippocampal_L (aal)

Peak intensity: -4.9535

# voxels structure

138 --TOTAL # VOXELS--

131 Left Cerebrum

129 Limbic Lobe

97 ParaHippocampal_L (aal)

86 Gray Matter

66 Uncus

63 Parahippocampa Gyrus

30 brodmann area 28

29 brodmann area 34

25 White Matter

22 Amygdala

22 Amygdala_L (aal)

13 Hippocampus_L (aal)

5 brodmann area 35

2 Sub-Gyral

2 Temporal Lobe

2 Temporal_Pole_Sup_L (aal)

----------------------

Cluster 50

Number of voxels: 24

Peak MNI coordinate: -43.5 -4.5 -25.5

Peak MNI coordinate region: // Left Cerebrum // Temporal Lobe // Sub-Gyral // White Matter // undefined // Temporal_Mid_L (aal)

Peak intensity: -4.0742

# voxels structure

24 --TOTAL # VOXELS--

24 Left Cerebrum

24 Temporal Lobe

23 Temporal_Mid_L (aal)

15 White Matter

12 Fusiform Gyrus

10 Middle Temporal Gyrus

7 Gray Matter

4 brodmann area 20

3 brodmann area 21

2 Sub-Gyral

1 Temporal_Inf_L (aal)

----------------------

Cluster 51

Number of voxels: 18

Peak MNI coordinate: -34.5 3 -27

Peak MNI coordinate region: // Left Cerebrum // Temporal Lobe // Sub-Gyral // White Matter // undefined // undefined

Peak intensity: -4.8463

# voxels structure

18 --TOTAL # VOXELS--

18 White Matter

18 Left Cerebrum

17 Temporal Lobe

11 Sub-Gyral

6 Superior Temporal Gyrus

1 Temporal_Pole_Sup_L (aal)

1 Uncus

1 Limbic Lobe

1 Temporal_Mid_L (aal)

----------------------

Cluster 52

Number of voxels: 11

Peak MNI coordinate: 1.5 -70.5 -28.5

Peak MNI coordinate region: // Right Cerebellum // Cerebellum Posterior Lobe // Declive of Vermis // undefined // undefined // Vermis_7 (aal)

Peak intensity: -3.7339

# voxels structure

11 --TOTAL # VOXELS--

11 Right Cerebellum

11 Vermis_7 (aal)

11 Cerebellum Posterior Lobe

9 Declive of Vermis

2 Declive

----------------------

Cluster 53

Number of voxels: 13

Peak MNI coordinate: 18 -55.5 -28.5

Peak MNI coordinate region: // Right Cerebellum // Cerebellum Anterior Lobe // undefined // undefined // Dentate // Cerebelum_6_R (aal)

Peak intensity: -4.3777

# voxels structure

13 --TOTAL # VOXELS--

13 Right Cerebellum

13 Dentate

12 Cerebellum Anterior Lobe

11 Cerebelum_6_R (aal)

1 Cerebellum Posterior Lobe

----------------------

Cluster 54

Number of voxels: 11

Peak MNI coordinate: -58.5 -33 -27

Peak MNI coordinate region: // undefined // Temporal Lobe // undefined // undefined // undefined // Temporal_Inf_L (aal)

Peak intensity: -3.6211

# voxels structure

11 --TOTAL # VOXELS--

10 Temporal_Inf_L (aal)

9 Temporal Lobe

7 Left Cerebrum

5 Inferior Temporal Gyrus

3 brodmann area 20

3 Gray Matter

2 Fusiform Gyrus

----------------------

Cluster 55

Number of voxels: 26

Peak MNI coordinate: -48 -16.5 -27

Peak MNI coordinate region: // Left Cerebrum // Temporal Lobe // Fusiform Gyrus // White Matter // undefined // Temporal_Inf_L (aal)

Peak intensity: -3.9436

# voxels structure

26 --TOTAL # VOXELS--

26 Left Cerebrum

26 Temporal Lobe

26 Temporal_Inf_L (aal)

23 White Matter

15 Inferior Temporal Gyrus

6 Sub-Gyral

5 Fusiform Gyrus

3 brodmann area 20

3 Gray Matter

----------------------

Cluster 56

Number of voxels: 26

Peak MNI coordinate: 40.5 6 -25.5

Peak MNI coordinate region: // Right Cerebrum // Temporal Lobe // Superior Temporal Gyrus // White Matter // undefined // undefined

Peak intensity: -3.7103

# voxels structure

26 --TOTAL # VOXELS--

26 White Matter

26 Right Cerebrum

21 Temporal Lobe

18 Superior Temporal Gyrus

13 Temporal_Pole_Sup_R (aal)

5 Limbic Lobe

5 Uncus

3 Amygdala_R (aal)

3 Sub-Gyral

----------------------

Cluster 57

Number of voxels: 12

Peak MNI coordinate: -13.5 -48 -27

Peak MNI coordinate region: // Left Cerebellum // Cerebellum Anterior Lobe // undefined // undefined // Dentate // undefined

Peak intensity: -3.7203

# voxels structure

12 --TOTAL # VOXELS--

12 Left Cerebellum

12 Cerebellum Anterior Lobe

6 Culmen

6 Dentate

5 Cerebelum_4_5_L (aal)

----------------------

Cluster 58

Number of voxels: 17

Peak MNI coordinate: 10.5 18 -25.5

Peak MNI coordinate region: // undefined // undefined // undefined // undefined // undefined // undefined

Peak intensity: -4.1794

# voxels structure

17 --TOTAL # VOXELS--

10 Right Cerebrum

10 Frontal Lobe

8 Gray Matter

5 Orbital Gyrus

5 brodmann area 11

5 Rectal Gyrus

4 Rectus_R (aal)

4 Frontal_Sup_Orb_R (aal)

3 brodmann area 47

----------------------

Cluster 59

Number of voxels: 11

Peak MNI coordinate: 10.5 -88.5 -22.5

Peak MNI coordinate region: // Right Cerebrum // Occipital Lobe // Lingual Gyrus // undefined // undefined // Cerebelum_Crus1_R (aal)

Peak intensity: -3.8185

# voxels structure

11 --TOTAL # VOXELS--

9 Cerebelum_Crus1_R (aal)

4 Occipital Lobe

4 Right Cerebrum

4 Lingual Gyrus

2 Cerebelum_Crus2_R (aal)

2 Right Cerebellum

2 Declive

2 Cerebellum Posterior Lobe

1 brodmann area 18

1 Gray Matter

----------------------

Cluster 60

Number of voxels: 10

Peak MNI coordinate: 10.5 -75 -24

Peak MNI coordinate region: // Right Cerebellum // Cerebellum Posterior Lobe // Declive // undefined // undefined // Cerebelum_Crus1_R (aal)

Peak intensity: -3.22

# voxels structure

10 --TOTAL # VOXELS--

10 Declive

10 Right Cerebellum

10 Cerebellum Posterior Lobe

6 Cerebelum_Crus1_R (aal)

2 Vermis_7 (aal)

2 Cerebelum_6_R (aal)

----------------------

Cluster 61

Number of voxels: 11

Peak MNI coordinate: 15 -40.5 -24

Peak MNI coordinate region: // Right Cerebellum // Cerebellum Anterior Lobe // Culmen // undefined // undefined // Cerebelum_4_5_R (aal)

Peak intensity: -3.794

# voxels structure

11 --TOTAL # VOXELS--

11 Culmen

11 Right Cerebellum

11 Cerebellum Anterior Lobe

8 Cerebelum_4_5_R (aal)

2 Cerebelum_3_R (aal)

----------------------

Cluster 62

Number of voxels: 16

Peak MNI coordinate: -13.5 -40.5 -24

Peak MNI coordinate region: // Left Cerebellum // Cerebellum Anterior Lobe // Culmen // undefined // undefined // Cerebelum_4_5_L (aal)

Peak intensity: -4.2274

# voxels structure

16 --TOTAL # VOXELS--

16 Culmen

16 Left Cerebellum

16 Cerebellum Anterior Lobe

15 Cerebelum_4_5_L (aal)

----------------------

Cluster 63

Number of voxels: 18

Peak MNI coordinate: 27 -18 -25.5

Peak MNI coordinate region: // Right Cerebrum // Limbic Lobe // Parahippocampa Gyrus // White Matter // undefined // ParaHippocampal_R (aal)

Peak intensity: -3.6499

# voxels structure

18 --TOTAL # VOXELS--

18 Parahippocampa Gyrus

18 Right Cerebrum

18 Limbic Lobe

16 White Matter

14 ParaHippocampal_R (aal)

2 Gray Matter

1 brodmann area 35

1 Hippocampus

----------------------

Cluster 64

Number of voxels: 19

Peak MNI coordinate: -34.5 -6 -21

Peak MNI coordinate region: // Left Cerebrum // Limbic Lobe // Parahippocampa Gyrus // White Matter // undefined // undefined

Peak intensity: -4.3131

# voxels structure

19 --TOTAL # VOXELS--

19 White Matter

19 Left Cerebrum

14 Limbic Lobe

14 Parahippocampa Gyrus

5 Sub-Gyral

5 Temporal Lobe

1 Hippocampus_L (aal)

----------------------

Cluster 65

Number of voxels: 21

Peak MNI coordinate: 34.5 -3 -22.5

Peak MNI coordinate region: // Right Cerebrum // Limbic Lobe // Parahippocampa Gyrus // White Matter // undefined // Hippocampus_R (aal)

Peak intensity: -3.9788

# voxels structure

21 --TOTAL # VOXELS--

21 White Matter

21 Right Cerebrum

11 Sub-Gyral

11 Temporal Lobe

10 Parahippocampa Gyrus

10 Limbic Lobe

5 Hippocampus_R (aal)

2 Amygdala_R (aal)

----------------------

Cluster 66

Number of voxels: 91

Peak MNI coordinate: 57 -1.5 -24

Peak MNI coordinate region: // Right Cerebrum // Temporal Lobe // Middle Temporal Gyrus // Gray Matter // brodmann area 21 // Temporal_Mid_R (aal)

Peak intensity: -4.279

# voxels structure

91 --TOTAL # VOXELS--

91 Right Cerebrum

91 Temporal Lobe

90 Temporal_Mid_R (aal)

75 Middle Temporal Gyrus

43 brodmann area 21

43 Gray Matter

38 White Matter

16 Inferior Temporal Gyrus

1 Temporal_Sup_R (aal)

----------------------

Cluster 67

Number of voxels: 27

Peak MNI coordinate: -19.5 31.5 -24

Peak MNI coordinate region: // Left Cerebrum // Frontal Lobe // Inferior Frontal Gyrus // White Matter // undefined // Frontal_Sup_Orb_L (aal)

Peak intensity: -3.7527

# voxels structure

27 --TOTAL # VOXELS--

27 Left Cerebrum

27 Frontal Lobe

19 Inferior Frontal Gyrus

13 Frontal_Mid_Orb_L (aal)

11 brodmann area 11

11 Gray Matter

9 White Matter

7 Superior Frontal Gyrus

5 Frontal_Sup_Orb_L (aal)

1 Orbital Gyrus

----------------------

Cluster 68

Number of voxels: 22

Peak MNI coordinate: 6 -70.5 -22.5

Peak MNI coordinate region: // Right Cerebellum // Cerebellum Posterior Lobe // Declive // undefined // undefined // Vermis_6 (aal)

Peak intensity: -4.5865

# voxels structure

22 --TOTAL # VOXELS--

22 Cerebellum Posterior Lobe

19 Vermis_6 (aal)

17 Right Cerebellum

14 Declive

8 Declive of Vermis

5 Left Cerebellum

1 Cerebelum_6_L (aal)

1 Cerebelum_6_R (aal)

1 Cerebelum_Crus1_L (aal)

----------------------

Cluster 69

Number of voxels: 14

Peak MNI coordinate: -21 -24 -19.5

Peak MNI coordinate region: // Left Cerebrum // Limbic Lobe // Parahippocampa Gyrus // Gray Matter // brodmann area 35 // ParaHippocampal_L (aal)

Peak intensity: -3.8407

# voxels structure

14 --TOTAL # VOXELS--

14 ParaHippocampal_L (aal)

12 Left Cerebrum

12 Parahippocampa Gyrus

12 Limbic Lobe

10 brodmann area 35

10 Gray Matter

----------------------

Cluster 70

Number of voxels: 16

Peak MNI coordinate: 48 -6 -24

Peak MNI coordinate region: // Right Cerebrum // Temporal Lobe // Inferior Temporal Gyrus // Gray Matter // brodmann area 20 // Temporal_Mid_R (aal)

Peak intensity: -3.4513

# voxels structure

16 --TOTAL # VOXELS--

16 Temporal Lobe

16 Right Cerebrum

11 White Matter

11 Sub-Gyral

6 Temporal_Mid_R (aal)

5 Gray Matter

3 brodmann area 20

3 Inferior Temporal Gyrus

2 brodmann area 21

2 Middle Temporal Gyrus

----------------------

Cluster 71

Number of voxels: 10

Peak MNI coordinate: -33 -33 -21

Peak MNI coordinate region: // Left Cerebrum // Temporal Lobe // Fusiform Gyrus // Gray Matter // brodmann area 20 // Fusiform_L (aal)

Peak intensity: -4.002

# voxels structure

10 --TOTAL # VOXELS--

10 Fusiform_L (aal)

10 Left Cerebrum

9 Gray Matter

9 Fusiform Gyrus

9 Temporal Lobe

9 brodmann area 20

1 Parahippocampa Gyrus

1 Limbic Lobe

----------------------

Cluster 72

Number of voxels: 15

Peak MNI coordinate: 16.5 6 -19.5

Peak MNI coordinate region: // Right Cerebrum // Frontal Lobe // undefined // undefined // undefined // undefined

Peak intensity: -3.7231

# voxels structure

15 --TOTAL # VOXELS--

14 Right Cerebrum

10 Frontal Lobe

4 Limbic Lobe

3 ParaHippocampal_R (aal)

2 Olfactory_R (aal)

2 Uncus

2 Parahippocampa Gyrus

1 Inferior Frontal Gyrus

1 Gray Matter

1 brodmann area 34

1 Medial Frontal Gyrus

----------------------

Cluster 73

Number of voxels: 47

Peak MNI coordinate: 48 -64.5 -18

Peak MNI coordinate region: // Right Cerebrum // Temporal Lobe // Fusiform Gyrus // White Matter // undefined // Fusiform_R (aal)

Peak intensity: -4.3587

# voxels structure

47 --TOTAL # VOXELS--

47 Right Cerebrum

28 Fusiform Gyrus

27 White Matter

27 Occipital Lobe

20 Temporal Lobe

19 Occipital_Inf_R (aal)

16 Gray Matter

15 Temporal_Inf_R (aal)

11 brodmann area 37

11 Middle Occipital Gyrus

10 Fusiform_R (aal)

8 Sub-Gyral

5 brodmann area 19

3 Cerebelum_Crus1_R (aal)

----------------------

Cluster 74

Number of voxels: 16

Peak MNI coordinate: 1.5 -58.5 -21

Peak MNI coordinate region: // Right Cerebellum // Cerebellum Anterior Lobe // Culmen // undefined // undefined // Vermis_6 (aal)

Peak intensity: -3.1788

# voxels structure

16 --TOTAL # VOXELS--

16 Vermis_6 (aal)

13 Right Cerebellum

10 Declive

10 Cerebellum Posterior Lobe

6 Cerebellum Anterior Lobe

5 Culmen

3 Left Cerebellum

1 Culmen of Vermis

----------------------

Cluster 75

Number of voxels: 31

Peak MNI coordinate: -52.5 -43.5 -18

Peak MNI coordinate region: // Left Cerebrum // Temporal Lobe // Middle Temporal Gyrus // undefined // undefined // Temporal_Inf_L (aal)

Peak intensity: -4.2061

# voxels structure

31 --TOTAL # VOXELS--

31 Left Cerebrum

31 Temporal Lobe

31 Temporal_Inf_L (aal)

21 Gray Matter

17 Middle Temporal Gyrus

11 brodmann area 37

10 brodmann area 20

6 Inferior Temporal Gyrus

5 Fusiform Gyrus

3 White Matter

3 Sub-Gyral

----------------------

Cluster 76

Number of voxels: 12

Peak MNI coordinate: 55.5 -36 -21

Peak MNI coordinate region: // Right Cerebrum // Temporal Lobe // Inferior Temporal Gyrus // White Matter // undefined // Temporal_Inf_R (aal)

Peak intensity: -3.1682

# voxels structure

12 --TOTAL # VOXELS--

12 Temporal Lobe

12 White Matter

12 Right Cerebrum

11 Temporal_Inf_R (aal)

7 Inferior Temporal Gyrus

5 Middle Temporal Gyrus

----------------------

Cluster 77

Number of voxels: 73

Peak MNI coordinate: -1.5 -90 -4.5

Peak MNI coordinate region: // Left Cerebrum // Occipital Lobe // Lingual Gyrus // Gray Matter // brodmann area 18 // Calcarine_L (aal)

Peak intensity: -4.5539

# voxels structure

73 --TOTAL # VOXELS--

73 Occipital Lobe

72 Left Cerebrum

66 Lingual Gyrus

48 Calcarine_L (aal)

46 Gray Matter

33 brodmann area 18

22 Lingual_L (aal)

22 White Matter

13 brodmann area 17

5 Inferior Occipital Gyrus

1 Inter-Hemispheric

1 Cerebelum_6_L (aal)

1 Cuneus

----------------------

Cluster 78

Number of voxels: 11

Peak MNI coordinate: -42 -49.5 -16.5

Peak MNI coordinate region: // Left Cerebrum // Temporal Lobe // Fusiform Gyrus // White Matter // undefined // Temporal_Inf_L (aal)

Peak intensity: -4.2015

# voxels structure

11 --TOTAL # VOXELS--

11 Fusiform Gyrus

11 Left Cerebrum

11 Temporal Lobe

11 White Matter

8 Temporal_Inf_L (aal)

3 Fusiform_L (aal)

----------------------

Cluster 79

Number of voxels: 33

Peak MNI coordinate: -45 -27 -10.5

Peak MNI coordinate region: // Left Cerebrum // Temporal Lobe // Sub-Gyral // White Matter // undefined // undefined

Peak intensity: -4.1572

# voxels structure

33 --TOTAL # VOXELS--

33 Sub-Gyral

33 Temporal Lobe

33 White Matter

33 Left Cerebrum

2 Temporal_Inf_L (aal)

2 Temporal_Mid_L (aal)

----------------------

Cluster 80

Number of voxels: 20

Peak MNI coordinate: -13.5 13.5 -18

Peak MNI coordinate region: // Left Cerebrum // Frontal Lobe // Medial Frontal Gyrus // Gray Matter // brodmann area 25 // Frontal_Sup_Orb_L (aal)

Peak intensity: -4.418

# voxels structure

20 --TOTAL # VOXELS--

20 Frontal Lobe

20 Left Cerebrum

13 Medial Frontal Gyrus

11 Gray Matter

9 Rectus_L (aal)

8 brodmann area 25

8 White Matter

7 Frontal_Sup_Orb_L (aal)

5 Subcallosal Gyrus

4 Olfactory_L (aal)

3 brodmann area 47

2 Inferior Frontal Gyrus

----------------------

Cluster 81

Number of voxels: 12

Peak MNI coordinate: -27 -73.5 -15

Peak MNI coordinate region: // Left Cerebrum // Occipital Lobe // Lingual Gyrus // White Matter // undefined // Fusiform_L (aal)

Peak intensity: -3.9669

# voxels structure

12 --TOTAL # VOXELS--

12 Fusiform_L (aal)

10 Occipital Lobe

10 Left Cerebrum

9 Lingual Gyrus

6 White Matter

3 brodmann area 18

3 Gray Matter

2 Cerebellum Posterior Lobe

2 Declive

2 Left Cerebellum

1 Fusiform Gyrus

----------------------

Cluster 82

Number of voxels: 10

Peak MNI coordinate: 28.5 -28.5 -16.5

Peak MNI coordinate region: // Right Cerebrum // Limbic Lobe // Parahippocampa Gyrus // White Matter // undefined // ParaHippocampal_R (aal)

Peak intensity: -4.121

# voxels structure

10 --TOTAL # VOXELS--

10 Limbic Lobe

10 ParaHippocampal_R (aal)

10 Parahippocampa Gyrus

10 Right Cerebrum

10 White Matter

----------------------

Cluster 83

Number of voxels: 16

Peak MNI coordinate: 7.5 61.5 -16.5

Peak MNI coordinate region: // Right Cerebrum // Frontal Lobe // Medial Frontal Gyrus // Gray Matter // brodmann area 11 // Rectus_R (aal)

Peak intensity: -3.4938

# voxels structure

16 --TOTAL # VOXELS--

16 Frontal Lobe

16 Medial Frontal Gyrus

16 Right Cerebrum

13 Gray Matter

13 brodmann area 11

7 Rectus_R (aal)

6 Frontal_Med_Orb_R (aal)

3 White Matter

3 Frontal_Sup_Orb_R (aal)

----------------------

Cluster 84

Number of voxels: 12

Peak MNI coordinate: 4.5 -76.5 -13.5

Peak MNI coordinate region: // undefined // undefined // undefined // undefined // undefined // Vermis_6 (aal)

Peak intensity: -3.4896

# voxels structure

12 --TOTAL # VOXELS--

11 Vermis_6 (aal)

6 Right Cerebellum

6 Cerebellum Posterior Lobe

6 Declive

1 Cerebelum_6_R (aal)

----------------------

Cluster 85

Number of voxels: 44

Peak MNI coordinate: -45 -75 -13.5

Peak MNI coordinate region: // Left Cerebrum // Occipital Lobe // Middle Occipital Gyrus // White Matter // undefined // Occipital_Inf_L (aal)

Peak intensity: -3.9665

# voxels structure

44 --TOTAL # VOXELS--

44 Left Cerebrum

43 White Matter

42 Middle Occipital Gyrus

42 Occipital Lobe

38 Occipital_Inf_L (aal)

6 Fusiform_L (aal)

2 Inferior Temporal Gyrus

2 Temporal Lobe

1 brodmann area 19

1 Gray Matter

----------------------

Cluster 86

Number of voxels: 15

Peak MNI coordinate: -12 -78 -15

Peak MNI coordinate region: // Left Cerebrum // Occipital Lobe // Lingual Gyrus // undefined // undefined // Cerebelum_6_L (aal)

Peak intensity: -3.6366

# voxels structure

15 --TOTAL # VOXELS--

15 Lingual Gyrus

15 Occipital Lobe

15 Left Cerebrum

10 Cerebelum_6_L (aal)

5 Lingual_L (aal)

2 brodmann area 18

2 Gray Matter

1 White Matter

----------------------

Cluster 87

Number of voxels: 10

Peak MNI coordinate: 60 -51 -13.5

Peak MNI coordinate region: // Right Cerebrum // Temporal Lobe // Middle Temporal Gyrus // Gray Matter // brodmann area 37 // Temporal_Inf_R (aal)

Peak intensity: -3.2598

# voxels structure

10 --TOTAL # VOXELS--

10 Right Cerebrum

10 Temporal Lobe

10 Temporal_Inf_R (aal)

7 Inferior Temporal Gyrus

6 Gray Matter

4 White Matter

3 brodmann area 20

3 brodmann area 37

3 Middle Temporal Gyrus

----------------------

Cluster 88

Number of voxels: 35

Peak MNI coordinate: 36 1.5 -7.5

Peak MNI coordinate region: // Right Cerebrum // Sub-lobar // Extra-Nuclear // White Matter // undefined // undefined

Peak intensity: -4.2929

# voxels structure

35 --TOTAL # VOXELS--

35 Right Cerebrum

34 White Matter

29 Sub-lobar

21 Extra-Nuclear

9 Putamen_R (aal)

7 Insula

6 Temporal Lobe

5 Sub-Gyral

1 Gray Matter

1 Claustrum

1 Superior Temporal Gyrus

----------------------

Cluster 89

Number of voxels: 69

Peak MNI coordinate: 3 67.5 -6

Peak MNI coordinate region: // Inter-Hemispheric // undefined // undefined // undefined // undefined // Frontal_Med_Orb_R (aal)

Peak intensity: -3.9989

# voxels structure

69 --TOTAL # VOXELS--

19 Inter-Hemispheric

19 Frontal_Med_Orb_R (aal)

18 Frontal_Med_Orb_L (aal)

7 Right Cerebrum

3 Superior Frontal Gyrus

3 Frontal Lobe

1 Frontal_Sup_Medial_L (aal)

1 brodmann area 11

1 Gray Matter

----------------------

Cluster 90

Number of voxels: 15

Peak MNI coordinate: -30 -91.5 -13.5

Peak MNI coordinate region: // Left Cerebrum // Occipital Lobe // Inferior Occipital Gyrus // White Matter // undefined // Lingual_L (aal)

Peak intensity: -3.7311

# voxels structure

15 --TOTAL # VOXELS--

15 Inferior Occipital Gyrus

15 Left Cerebrum

15 Occipital Lobe

13 White Matter

7 Lingual_L (aal)

7 Occipital_Inf_L (aal)

2 brodmann area 18

2 Gray Matter

1 Fusiform_L (aal)

----------------------

Cluster 91

Number of voxels: 10

Peak MNI coordinate: 34.5 -87 -13.5

Peak MNI coordinate region: // Right Cerebrum // Occipital Lobe // Inferior Occipital Gyrus // Gray Matter // brodmann area 18 // Occipital_Inf_R (aal)

Peak intensity: -3.713

# voxels structure

10 --TOTAL # VOXELS--

10 Inferior Occipital Gyrus

10 Occipital Lobe

10 Occipital_Inf_R (aal)

10 Right Cerebrum

9 Gray Matter

9 brodmann area 18

1 White Matter

----------------------

Cluster 92

Number of voxels: 10

Peak MNI coordinate: -21 -85.5 -12

Peak MNI coordinate region: // Left Cerebrum // Occipital Lobe // Lingual Gyrus // White Matter // undefined // Fusiform_L (aal)

Peak intensity: -3.7419

# voxels structure

10 --TOTAL # VOXELS--

10 Left Cerebrum

10 Lingual Gyrus

10 Occipital Lobe

10 White Matter

6 Lingual_L (aal)

4 Fusiform_L (aal)

----------------------

Cluster 93

Number of voxels: 27

Peak MNI coordinate: 30 -15 -10.5

Peak MNI coordinate region: // Right Cerebrum // Sub-lobar // Lateral Ventricle // Cerebro-Spinal Fluid // undefined // undefined

Peak intensity: -4.8849

# voxels structure

27 --TOTAL # VOXELS--

27 Right Cerebrum

17 White Matter

17 Sub-lobar

9 Lateral Ventricle

9 Temporal Lobe

9 Cerebro-Spinal Fluid

9 Sub-Gyral

7 Extra-Nuclear

6 Hippocampus_R (aal)

1 Lentiform Nucleus

1 Limbic Lobe

1 Parahippocampa Gyrus

1 Putamen

1 Gray Matter

----------------------

Cluster 94

Number of voxels: 17

Peak MNI coordinate: -19.5 7.5 -13.5

Peak MNI coordinate region: // Left Cerebrum // Frontal Lobe // Subcallosal Gyrus // White Matter // undefined // Olfactory_L (aal)

Peak intensity: -3.7044

# voxels structure

17 --TOTAL # VOXELS--

17 Left Cerebrum

10 Subcallosal Gyrus

10 Frontal Lobe

9 Gray Matter

9 Olfactory_L (aal)

8 White Matter

7 Sub-lobar

6 Lentiform Nucleus

6 Putamen

4 Putamen_L (aal)

3 brodmann area 34

1 Extra-Nuclear

----------------------

Cluster 95

Number of voxels: 30

Peak MNI coordinate: 19.5 7.5 -12

Peak MNI coordinate region: // Right Cerebrum // Sub-lobar // Lentiform Nucleus // Gray Matter // Putamen // undefined

Peak intensity: -3.805

# voxels structure

30 --TOTAL # VOXELS--

30 Right Cerebrum

23 White Matter

20 Sub-lobar

13 Extra-Nuclear

13 Putamen_R (aal)

10 Frontal Lobe

10 Subcallosal Gyrus

7 Olfactory_R (aal)

7 Putamen

7 Gray Matter

7 Lentiform Nucleus

----------------------

Cluster 96

Number of voxels: 15

Peak MNI coordinate: -7.5 12 -13.5

Peak MNI coordinate region: // Left Cerebrum // Frontal Lobe // Subcallosal Gyrus // White Matter // undefined // Olfactory_L (aal)

Peak intensity: -3.7786

# voxels structure

15 --TOTAL # VOXELS--

15 Left Cerebrum

14 White Matter

10 Frontal Lobe

10 Subcallosal Gyrus

8 Caudate_L (aal)

5 Olfactory_L (aal)

4 Anterior Cingulate

4 Limbic Lobe

2 Rectus_L (aal)

1 Sub-lobar

1 Gray Matter

----------------------

Cluster 97

Number of voxels: 13

Peak MNI coordinate: 24 48 -13.5

Peak MNI coordinate region: // Right Cerebrum // Frontal Lobe // Middle Frontal Gyrus // White Matter // undefined // Frontal_Mid_Orb_R (aal)

Peak intensity: -4.0675

# voxels structure

13 --TOTAL # VOXELS--

13 Frontal Lobe

13 Right Cerebrum

9 White Matter

7 Middle Frontal Gyrus

7 Frontal_Mid_Orb_R (aal)

6 Frontal_Sup_Orb_R (aal)

6 Superior Frontal Gyrus

4 brodmann area 11

4 Gray Matter

----------------------

Cluster 98

Number of voxels: 13

Peak MNI coordinate: -16.5 -96 -10.5

Peak MNI coordinate region: // Left Cerebrum // Occipital Lobe // Lingual Gyrus // White Matter // undefined // Occipital_Inf_L (aal)

Peak intensity: -3.5035

# voxels structure

13 --TOTAL # VOXELS--

13 Left Cerebrum

13 Lingual Gyrus

13 Occipital Lobe

12 White Matter

8 Occipital_Inf_L (aal)

5 Lingual_L (aal)

1 brodmann area 17

1 Gray Matter

----------------------

Cluster 99

Number of voxels: 27

Peak MNI coordinate: 15 -72 -9

Peak MNI coordinate region: // Right Cerebrum // Occipital Lobe // Lingual Gyrus // White Matter // undefined // Lingual_R (aal)

Peak intensity: -4.222

# voxels structure

27 --TOTAL # VOXELS--

27 Lingual Gyrus

27 Lingual_R (aal)

27 Occipital Lobe

27 Right Cerebrum

13 Gray Matter

12 White Matter

10 brodmann area 18

----------------------

Cluster 100

Number of voxels: 28

Peak MNI coordinate: 55.5 -70.5 -9

Peak MNI coordinate region: // Right Cerebrum // Occipital Lobe // Middle Occipital Gyrus // White Matter // undefined // Temporal_Inf_R (aal)

Peak intensity: -3.3279

# voxels structure

28 --TOTAL # VOXELS--

28 Right Cerebrum

25 Temporal_Inf_R (aal)

19 Occipital Lobe

18 Gray Matter

16 Middle Occipital Gyrus

9 brodmann area 37

9 Temporal Lobe

7 Middle Temporal Gyrus

7 brodmann area 19

6 White Matter

5 Inferior Temporal Gyrus

1 Occipital_Inf_R (aal)

----------------------

Cluster 101

Number of voxels: 22

Peak MNI coordinate: 3 58.5 -10.5

Peak MNI coordinate region: // Right Cerebrum // Frontal Lobe // Medial Frontal Gyrus // Gray Matter // brodmann area 11 // Frontal_Med_Orb_R (aal)

Peak intensity: -5.6373

# voxels structure

22 --TOTAL # VOXELS--

22 Frontal Lobe

22 Frontal_Med_Orb_R (aal)

22 Medial Frontal Gyrus

22 Right Cerebrum

17 Gray Matter

17 brodmann area 11

5 White Matter

----------------------

Cluster 102

Number of voxels: 14

Peak MNI coordinate: -21 -66 -10.5

Peak MNI coordinate region: // Left Cerebrum // Occipital Lobe // Lingual Gyrus // White Matter // undefined // Lingual_L (aal)

Peak intensity: -3.6492

# voxels structure

14 --TOTAL # VOXELS--

14 Left Cerebrum

14 Lingual_L (aal)

14 Occipital Lobe

10 Lingual Gyrus

10 White Matter

4 Fusiform Gyrus

4 brodmann area 19

4 Gray Matter

----------------------

Cluster 103

Number of voxels: 11

Peak MNI coordinate: -61.5 -25.5 -10.5

Peak MNI coordinate region: // Left Cerebrum // Temporal Lobe // Middle Temporal Gyrus // Gray Matter // brodmann area 21 // Temporal_Mid_L (aal)

Peak intensity: -2.9769

# voxels structure

11 --TOTAL # VOXELS--

11 Left Cerebrum

11 Middle Temporal Gyrus

11 Temporal Lobe

11 Temporal_Mid_L (aal)

6 White Matter

5 brodmann area 21

5 Gray Matter

----------------------

Cluster 104

Number of voxels: 12

Peak MNI coordinate: -18 -10.5 -10.5

Peak MNI coordinate region: // Left Cerebrum // Sub-lobar // Extra-Nuclear // White Matter // undefined // undefined

Peak intensity: -5.3293

# voxels structure

12 --TOTAL # VOXELS--

12 Left Cerebrum

12 Sub-lobar

12 White Matter

12 Extra-Nuclear

5 Hippocampus_L (aal)

----------------------

Cluster 105

Number of voxels: 75

Peak MNI coordinate: -43.5 37.5 -9

Peak MNI coordinate region: // Left Cerebrum // Frontal Lobe // Middle Frontal Gyrus // White Matter // undefined // Frontal_Inf_Orb_L (aal)

Peak intensity: -4.6397

# voxels structure

75 --TOTAL # VOXELS--

75 Frontal Lobe

75 Left Cerebrum

68 White Matter

47 Frontal_Inf_Orb_L (aal)

36 Middle Frontal Gyrus

24 Inferior Frontal Gyrus

23 Frontal_Inf_Tri_L (aal)

15 Sub-Gyral

7 brodmann area 47

7 Gray Matter

3 Frontal_Mid_L (aal)

----------------------

Cluster 106

Number of voxels: 14

Peak MNI coordinate: 48 -75 -7.5

Peak MNI coordinate region: // Right Cerebrum // Occipital Lobe // Middle Occipital Gyrus // White Matter // undefined // Occipital_Inf_R (aal)

Peak intensity: -3.2119

# voxels structure

14 --TOTAL # VOXELS--

14 Middle Occipital Gyrus

14 Occipital Lobe

14 Right Cerebrum

12 White Matter

9 Temporal_Inf_R (aal)

5 Occipital_Inf_R (aal)

2 brodmann area 19

2 Gray Matter

----------------------

Cluster 107

Number of voxels: 14

Peak MNI coordinate: -13.5 -103.5 -4.5

Peak MNI coordinate region: // Left Cerebrum // Occipital Lobe // Cuneus // Gray Matter // brodmann area 18 // Calcarine_L (aal)

Peak intensity: -3.5993

# voxels structure

14 --TOTAL # VOXELS--

14 Left Cerebrum

14 Occipital Lobe

13 Cuneus

11 Calcarine_L (aal)

8 Gray Matter

6 White Matter

3 brodmann area 17

3 brodmann area 18

3 Occipital_Inf_L (aal)

1 Lingual Gyrus

----------------------

Cluster 108

Number of voxels: 16

Peak MNI coordinate: 21 69 -6

Peak MNI coordinate region: // undefined // undefined // undefined // undefined // undefined // undefined

Peak intensity: -3.4093

# voxels structure

16 --TOTAL # VOXELS--

2 Frontal_Sup_Orb_R (aal)

2 Gray Matter

2 Right Cerebrum

2 Superior Frontal Gyrus

2 brodmann area 10

2 Frontal Lobe

----------------------

Cluster 109

Number of voxels: 18

Peak MNI coordinate: 28.5 -84 -1.5

Peak MNI coordinate region: // Right Cerebrum // Occipital Lobe // Sub-Gyral // White Matter // undefined // Fusiform_R (aal)

Peak intensity: -3.6786

# voxels structure

18 --TOTAL # VOXELS--

18 Right Cerebrum

18 Occipital Lobe

16 White Matter

11 Middle Occipital Gyrus

8 Fusiform_R (aal)

6 Sub-Gyral

5 Occipital_Inf_R (aal)

2 brodmann area 18

2 Gray Matter

1 Inferior Occipital Gyrus

----------------------

Cluster 110

Number of voxels: 20

Peak MNI coordinate: -3 -70.5 -6

Peak MNI coordinate region: // undefined // undefined // undefined // undefined // undefined // Vermis_6 (aal)

Peak intensity: -3.7494

# voxels structure

20 --TOTAL # VOXELS--

12 Lingual Gyrus

9 Occipital Lobe

9 Left Cerebrum

8 Lingual_L (aal)

6 Vermis_6 (aal)

3 Inter-Hemispheric

2 brodmann area 19

2 Gray Matter

1 Cerebelum_6_L (aal)

----------------------

Cluster 111

Number of voxels: 37

Peak MNI coordinate: 51 -67.5 -6

Peak MNI coordinate region: // Right Cerebrum // Temporal Lobe // Inferior Temporal Gyrus // White Matter // undefined // Temporal_Inf_R (aal)

Peak intensity: -4.3701

# voxels structure

37 --TOTAL # VOXELS--

37 Right Cerebrum

37 Temporal_Inf_R (aal)

28 Inferior Temporal Gyrus

20 Temporal Lobe

19 White Matter

17 Occipital Lobe

16 Gray Matter

13 brodmann area 37

6 Middle Temporal Gyrus

3 Middle Occipital Gyrus

----------------------

Cluster 112

Number of voxels: 27

Peak MNI coordinate: 18 54 -3

Peak MNI coordinate region: // Right Cerebrum // Frontal Lobe // Medial Frontal Gyrus // White Matter // undefined // Frontal_Sup_Orb_R (aal)

Peak intensity: -4.1137

# voxels structure

27 --TOTAL # VOXELS--

27 Right Cerebrum

27 White Matter

27 Frontal Lobe

20 Medial Frontal Gyrus

19 Frontal_Med_Orb_R (aal)

6 Sub-Gyral

5 Frontal_Sup_Orb_R (aal)

1 Frontal_Sup_Medial_R (aal)

1 Superior Frontal Gyrus

----------------------

Cluster 113

Number of voxels: 184

Peak MNI coordinate: -51 -31.5 7.5

Peak MNI coordinate region: // Left Cerebrum // Temporal Lobe // Superior Temporal Gyrus // White Matter // undefined // Temporal_Mid_L (aal)

Peak intensity: -4.2028

# voxels structure

184 --TOTAL # VOXELS--

184 Left Cerebrum

184 Temporal Lobe

166 White Matter

166 Superior Temporal Gyrus

92 Temporal_Sup_L (aal)

81 Temporal_Mid_L (aal)

18 Gray Matter

10 brodmann area 22

10 Middle Temporal Gyrus

8 Sub-Gyral

5 brodmann area 21

2 brodmann area 41

----------------------

Cluster 114

Number of voxels: 16

Peak MNI coordinate: -30 -21 -3

Peak MNI coordinate region: // Left Cerebrum // Sub-lobar // Lentiform Nucleus // Gray Matter // Putamen // undefined

Peak intensity: -4.0168

# voxels structure

16 --TOTAL # VOXELS--

16 Sub-lobar

16 Left Cerebrum

11 Putamen

11 Gray Matter

11 Lentiform Nucleus

5 White Matter

5 Extra-Nuclear

----------------------

Cluster 115

Number of voxels: 24

Peak MNI coordinate: -54 0 -4.5

Peak MNI coordinate region: // Left Cerebrum // Temporal Lobe // Superior Temporal Gyrus // White Matter // undefined // Temporal_Sup_L (aal)

Peak intensity: -4.0372

# voxels structure

24 --TOTAL # VOXELS--

24 Left Cerebrum

24 Superior Temporal Gyrus

24 Temporal Lobe

24 Temporal_Sup_L (aal)

23 White Matter

1 brodmann area 22

1 Gray Matter

----------------------

Cluster 116

Number of voxels: 25

Peak MNI coordinate: -6 16.5 -4.5

Peak MNI coordinate region: // Left Cerebrum // Sub-lobar // Caudate // Gray Matter // Caudate Head // Caudate_L (aal)

Peak intensity: -4.4084

# voxels structure

25 --TOTAL # VOXELS--

25 Left Cerebrum

17 Caudate_L (aal)

16 Gray Matter

16 Anterior Cingulate

16 Limbic Lobe

9 Sub-lobar

7 Olfactory_L (aal)

7 Caudate

7 brodmann area 25

7 Caudate Head

6 White Matter

2 Extra-Nuclear

2 brodmann area 24

----------------------

Cluster 117

Number of voxels: 14

Peak MNI coordinate: -4.5 -33 -1.5

Peak MNI coordinate region: // Left Brainstem // Midbrain // undefined // undefined // undefined // undefined

Peak intensity: -3.4946

# voxels structure

14 --TOTAL # VOXELS--

14 Midbrain

14 Left Brainstem

----------------------

Cluster 118

Number of voxels: 10

Peak MNI coordinate: 34.5 -13.5 -1.5

Peak MNI coordinate region: // Right Cerebrum // Sub-lobar // Extra-Nuclear // White Matter // undefined // Putamen_R (aal)

Peak intensity: -3.2166

# voxels structure

10 --TOTAL # VOXELS--

10 Sub-lobar

10 Right Cerebrum

6 White Matter

5 Extra-Nuclear

5 Putamen_R (aal)

4 Gray Matter

4 Claustrum

1 Insula_R (aal)

1 Insula

----------------------

Cluster 119

Number of voxels: 40

Peak MNI coordinate: 51 -40.5 0

Peak MNI coordinate region: // Right Cerebrum // Temporal Lobe // Middle Temporal Gyrus // White Matter // undefined // Temporal_Mid_R (aal)

Peak intensity: -3.6884

# voxels structure

40 --TOTAL # VOXELS--

40 Right Cerebrum

40 Temporal Lobe

39 Middle Temporal Gyrus

37 Temporal_Mid_R (aal)

33 White Matter

6 brodmann area 22

6 Gray Matter

1 Sub-Gyral

----------------------

Cluster 120

Number of voxels: 11

Peak MNI coordinate: -33 -18 -1.5

Peak MNI coordinate region: // Left Cerebrum // Sub-lobar // Extra-Nuclear // White Matter // undefined // undefined

Peak intensity: -4.0226

# voxels structure

11 --TOTAL # VOXELS--

11 Left Cerebrum

11 Sub-lobar

11 White Matter

11 Extra-Nuclear

----------------------

Cluster 121

Number of voxels: 137

Peak MNI coordinate: -4.5 -102 3

Peak MNI coordinate region: // Left Cerebrum // Occipital Lobe // Cuneus // Gray Matter // brodmann area 18 // Calcarine_L (aal)

Peak intensity: -4.9231

# voxels structure

137 --TOTAL # VOXELS--

134 Occipital Lobe

132 Left Cerebrum

125 Cuneus

66 White Matter

59 brodmann area 18

59 Gray Matter

56 Calcarine_L (aal)

54 Occipital_Sup_L (aal)

21 Occipital_Mid_L (aal)

9 Middle Occipital Gyrus

5 Cuneus_L (aal)

2 Inter-Hemispheric

----------------------

Cluster 122

Number of voxels: 27

Peak MNI coordinate: 36 -70.5 0

Peak MNI coordinate region: // Right Cerebrum // Occipital Lobe // Middle Occipital Gyrus // White Matter // undefined // undefined

Peak intensity: -3.3722

# voxels structure

27 --TOTAL # VOXELS--

27 White Matter

27 Right Cerebrum

20 Occipital Lobe

14 Middle Occipital Gyrus

13 Sub-Gyral

7 Temporal Lobe

----------------------

Cluster 123

Number of voxels: 10

Peak MNI coordinate: -69 -37.5 1.5

Peak MNI coordinate region: // undefined // undefined // undefined // undefined // undefined // Temporal_Mid_L (aal)

Peak intensity: -3.3185

# voxels structure

10 --TOTAL # VOXELS--

10 Temporal_Mid_L (aal)

1 Temporal Lobe

1 Left Cerebrum

1 Middle Temporal Gyrus

----------------------

Cluster 124

Number of voxels: 11

Peak MNI coordinate: -46.5 -15 1.5

Peak MNI coordinate region: // Left Cerebrum // Temporal Lobe // Insula // undefined // undefined // Temporal_Sup_L (aal)

Peak intensity: -3.4698

# voxels structure

11 --TOTAL # VOXELS--

11 Left Cerebrum

10 Temporal_Sup_L (aal)

10 Temporal Lobe

6 Superior Temporal Gyrus

5 Insula

5 Gray Matter

4 brodmann area 22

1 Sub-lobar

1 Heschl_L (aal)

----------------------

Cluster 125

Number of voxels: 42

Peak MNI coordinate: -16.5 15 6

Peak MNI coordinate region: // Left Cerebrum // Sub-lobar // Caudate // Gray Matter // Caudate Head // Caudate_L (aal)

Peak intensity: -4.3397

# voxels structure

42 --TOTAL # VOXELS--

42 Left Cerebrum

42 Sub-lobar

29 Caudate_L (aal)

27 White Matter

27 Extra-Nuclear

15 Caudate

15 Gray Matter

13 Caudate Head

2 Caudate Body

1 Putamen_L (aal)

----------------------

Cluster 126

Number of voxels: 14

Peak MNI coordinate: 18 -100.5 3

Peak MNI coordinate region: // Right Cerebrum // Occipital Lobe // Cuneus // Gray Matter // brodmann area 18 // Calcarine_R (aal)

Peak intensity: -3.8946

# voxels structure

14 --TOTAL # VOXELS--

14 Occipital Lobe

14 Right Cerebrum

13 Cuneus

10 Gray Matter

10 brodmann area 18

9 Calcarine_R (aal)

4 White Matter

4 Cuneus_R (aal)

1 Middle Occipital Gyrus

1 Occipital_Sup_R (aal)

----------------------

Cluster 127

Number of voxels: 15

Peak MNI coordinate: -6 -79.5 4.5

Peak MNI coordinate region: // Left Cerebrum // Occipital Lobe // Cuneus // White Matter // undefined // Calcarine_L (aal)

Peak intensity: -3.1358

# voxels structure

15 --TOTAL # VOXELS--

15 Cuneus

15 Left Cerebrum

15 Occipital Lobe

14 White Matter

12 Calcarine_L (aal)

3 Lingual_L (aal)

1 brodmann area 17

1 Gray Matter

----------------------

Cluster 128

Number of voxels: 10

Peak MNI coordinate: 6 -7.5 3

Peak MNI coordinate region: // Right Cerebrum // Sub-lobar // Thalamus // Gray Matter // undefined // Thalamus_R (aal)

Peak intensity: -4.3213

# voxels structure

10 --TOTAL # VOXELS--

10 Sub-lobar

10 Right Cerebrum

8 Thalamus

8 Gray Matter

5 Thalamus_R (aal)

2 White Matter

2 Extra-Nuclear

1 Medial Dorsal Nucleus

----------------------

Cluster 129

Number of voxels: 18

Peak MNI coordinate: 57 16.5 6

Peak MNI coordinate region: // Right Cerebrum // Frontal Lobe // Inferior Frontal Gyrus // undefined // undefined // Frontal_Inf_Oper_R (aal)

Peak intensity: -4.3342

# voxels structure

18 --TOTAL # VOXELS--

18 Right Cerebrum

17 Frontal Lobe

16 Inferior Frontal Gyrus

15 Frontal_Inf_Oper_R (aal)

11 Gray Matter

9 brodmann area 45

3 Frontal_Inf_Tri_R (aal)

3 White Matter

1 brodmann area 44

1 Frontal-Temporal Space

1 Precentral Gyrus

----------------------

Cluster 130

Number of voxels: 13

Peak MNI coordinate: 28.5 19.5 1.5

Peak MNI coordinate region: // Right Cerebrum // Sub-lobar // Claustrum // Gray Matter // undefined // Putamen_R (aal)

Peak intensity: -3.258

# voxels structure

13 --TOTAL # VOXELS--

13 Sub-lobar

13 Right Cerebrum

9 White Matter

9 Insula

5 Insula_R (aal)

4 Gray Matter

2 Claustrum

2 Putamen_R (aal)

2 Extra-Nuclear

1 brodmann area 13

----------------------

Cluster 131

Number of voxels: 83

Peak MNI coordinate: 10.5 -100.5 9

Peak MNI coordinate region: // Right Cerebrum // Occipital Lobe // Middle Occipital Gyrus // Gray Matter // brodmann area 18 // undefined

Peak intensity: -4.5817

# voxels structure

83 --TOTAL # VOXELS--

83 Occipital Lobe

83 Right Cerebrum

47 Middle Occipital Gyrus

46 Cuneus_R (aal)

43 brodmann area 18

43 Gray Matter

40 White Matter

36 Cuneus

11 Calcarine_R (aal)

9 Occipital_Sup_R (aal)

----------------------

Cluster 132

Number of voxels: 52

Peak MNI coordinate: -43.5 -73.5 7.5

Peak MNI coordinate region: // Left Cerebrum // Temporal Lobe // Middle Temporal Gyrus // Gray Matter // brodmann area 39 // Occipital_Mid_L (aal)

Peak intensity: -4.5501

# voxels structure

52 --TOTAL # VOXELS--

52 Left Cerebrum

50 Occipital_Mid_L (aal)

31 Gray Matter

28 Temporal Lobe

24 Occipital Lobe

24 Middle Occipital Gyrus

24 Middle Temporal Gyrus

20 White Matter

16 brodmann area 19

15 brodmann area 39

4 Sub-Gyral

2 Temporal_Mid_L (aal)

----------------------

Cluster 133

Number of voxels: 13

Peak MNI coordinate: 42 -75 3

Peak MNI coordinate region: // Right Cerebrum // Occipital Lobe // Middle Occipital Gyrus // Gray Matter // brodmann area 19 // Occipital_Mid_R (aal)

Peak intensity: -3.5194

# voxels structure

13 --TOTAL # VOXELS--

13 Middle Occipital Gyrus

13 Occipital Lobe

13 Right Cerebrum

12 Occipital_Mid_R (aal)

6 brodmann area 19

6 Gray Matter

5 White Matter

----------------------

Cluster 134

Number of voxels: 10

Peak MNI coordinate: 13.5 -48 9

Peak MNI coordinate region: // Right Cerebrum // Sub-lobar // Extra-Nuclear // White Matter // Corpus Callosum // Calcarine_R (aal)

Peak intensity: -3.5848

# voxels structure

10 --TOTAL # VOXELS--

10 Right Cerebrum

7 Extra-Nuclear

7 Sub-lobar

7 White Matter

5 Calcarine_R (aal)

4 Corpus Callosum

3 Precuneus_R (aal)

3 Gray Matter

3 Limbic Lobe

3 Posterior Cingulate

3 brodmann area 29

2 Lingual_R (aal)

----------------------

Cluster 135

Number of voxels: 13

Peak MNI coordinate: 7.5 -15 4.5

Peak MNI coordinate region: // Right Cerebrum // Sub-lobar // Thalamus // Gray Matter // undefined // Thalamus_R (aal)

Peak intensity: -4.7381

# voxels structure

13 --TOTAL # VOXELS--

13 Gray Matter

13 Right Cerebrum

13 Sub-lobar

13 Thalamus

13 Thalamus_R (aal)

7 Medial Dorsal Nucleus

2 Ventral Lateral Nucleus

----------------------

Cluster 136

Number of voxels: 96

Peak MNI coordinate: -66 -21 19.5

Peak MNI coordinate region: // Left Cerebrum // Parietal Lobe // Postcentral Gyrus // Gray Matter // brodmann area 40 // SupraMarginal_L (aal)

Peak intensity: -4.3354

# voxels structure

96 --TOTAL # VOXELS--

96 Left Cerebrum

66 Gray Matter

48 Parietal Lobe

47 Temporal Lobe

46 Postcentral Gyrus

37 Postcentral_L (aal)

28 Superior Temporal Gyrus

21 Temporal_Sup_L (aal)

20 brodmann area 40

19 brodmann area 43

19 brodmann area 42

16 Transverse Temporal Gyrus

14 SupraMarginal_L (aal)

7 White Matter

4 Precentral Gyrus

4 brodmann area 22

3 brodmann area 2

3 Rolandic_Oper_L (aal)

2 Inferior Parietal Lobule

1 Frontal Lobe

1 brodmann area 1

----------------------

Cluster 137

Number of voxels: 23

Peak MNI coordinate: 37.5 52.5 4.5

Peak MNI coordinate region: // Right Cerebrum // Frontal Lobe // Middle Frontal Gyrus // undefined // undefined // Frontal_Mid_R (aal)

Peak intensity: -3.8067

# voxels structure

23 --TOTAL # VOXELS--

23 Frontal_Mid_R (aal)

23 Right Cerebrum

23 Frontal Lobe

15 Gray Matter

12 Inferior Frontal Gyrus

11 Middle Frontal Gyrus

3 brodmann area 10

3 White Matter

----------------------

Cluster 138

Number of voxels: 66

Peak MNI coordinate: 28.5 66 15

Peak MNI coordinate region: // Right Cerebrum // Frontal Lobe // Superior Frontal Gyrus // undefined // undefined // Frontal_Sup_R (aal)

Peak intensity: -3.7839

# voxels structure

66 --TOTAL # VOXELS--

63 Frontal Lobe

63 Right Cerebrum

57 Superior Frontal Gyrus

53 Frontal_Sup_R (aal)

52 Gray Matter

50 brodmann area 10

8 Frontal_Sup_Medial_R (aal)

6 Middle Frontal Gyrus

----------------------

Cluster 139

Number of voxels: 10

Peak MNI coordinate: -19.5 -102 6

Peak MNI coordinate region: // Left Cerebrum // Occipital Lobe // Cuneus // Gray Matter // brodmann area 18 // Occipital_Mid_L (aal)

Peak intensity: -4.706

# voxels structure

10 --TOTAL # VOXELS--

10 Left Cerebrum

10 Occipital Lobe

9 Gray Matter

9 brodmann area 18

8 Occipital_Mid_L (aal)

6 Middle Occipital Gyrus

4 Cuneus

2 Occipital_Sup_L (aal)

1 White Matter

----------------------

Cluster 140

Number of voxels: 16

Peak MNI coordinate: 13.5 -91.5 6

Peak MNI coordinate region: // Right Cerebrum // Occipital Lobe // Cuneus // White Matter // undefined // Calcarine_R (aal)

Peak intensity: -4.3192

# voxels structure

16 --TOTAL # VOXELS--

16 Cuneus

16 Occipital Lobe

16 Right Cerebrum

16 White Matter

13 Calcarine_R (aal)

3 Cuneus_R (aal)

----------------------

Cluster 141

Number of voxels: 21

Peak MNI coordinate: 67.5 -31.5 6

Peak MNI coordinate region: // Right Cerebrum // Temporal Lobe // Superior Temporal Gyrus // undefined // undefined // Temporal_Sup_R (aal)

Peak intensity: -4.2464

# voxels structure

21 --TOTAL # VOXELS--

21 Right Cerebrum

21 Temporal Lobe

21 Temporal_Sup_R (aal)

18 Superior Temporal Gyrus

16 Gray Matter

8 brodmann area 42

8 brodmann area 22

3 Middle Temporal Gyrus

----------------------

Cluster 142

Number of voxels: 164

Peak MNI coordinate: 13.5 55.5 7.5

Peak MNI coordinate region: // Right Cerebrum // Frontal Lobe // Medial Frontal Gyrus // White Matter // undefined // Frontal_Sup_Medial_R (aal)

Peak intensity: -5.037

# voxels structure

164 --TOTAL # VOXELS--

163 Frontal Lobe

163 Right Cerebrum

154 Medial Frontal Gyrus

142 White Matter

123 Frontal_Sup_Medial_R (aal)

41 Frontal_Sup_R (aal)

19 brodmann area 10

19 Gray Matter

6 Superior Frontal Gyrus

1 Inter-Hemispheric

----------------------

Cluster 143

Number of voxels: 14

Peak MNI coordinate: -24 -94.5 7.5

Peak MNI coordinate region: // Left Cerebrum // Occipital Lobe // Middle Occipital Gyrus // White Matter // undefined // Occipital_Mid_L (aal)

Peak intensity: -3.6167

# voxels structure

14 --TOTAL # VOXELS--

14 Left Cerebrum

14 Occipital Lobe

14 Occipital_Mid_L (aal)

13 White Matter

10 Middle Occipital Gyrus

4 Cuneus

1 brodmann area 18

1 Gray Matter

----------------------

Cluster 144

Number of voxels: 283

Peak MNI coordinate: -66 -3 18

Peak MNI coordinate region: // Left Cerebrum // undefined // undefined // undefined // undefined // Postcentral_L (aal)

Peak intensity: -4.614

# voxels structure

283 --TOTAL # VOXELS--

268 Left Cerebrum

201 Frontal Lobe

186 Precentral Gyrus

130 Gray Matter

98 Postcentral_L (aal)

71 brodmann area 6

55 Frontal_Inf_Oper_L (aal)

47 Parietal Lobe

37 Postcentral Gyrus

34 Rolandic_Oper_L (aal)

31 Inferior Frontal Gyrus

30 brodmann area 44

30 Precentral_L (aal)

30 White Matter

15 Temporal Lobe

11 brodmann area 4

11 Superior Temporal Gyrus

9 brodmann area 22

4 brodmann area 9

4 Transverse Temporal Gyrus

2 brodmann area 45

2 brodmann area 42

1 brodmann area 1

----------------------

Cluster 145

Number of voxels: 35

Peak MNI coordinate: -6 -54 12

Peak MNI coordinate region: // Left Cerebrum // Limbic Lobe // Posterior Cingulate // Gray Matter // brodmann area 30 // Precuneus_L (aal)

Peak intensity: -4.2965

# voxels structure

35 --TOTAL # VOXELS--

35 Left Cerebrum

35 Limbic Lobe

35 Precuneus_L (aal)

34 Posterior Cingulate

19 White Matter

16 Gray Matter

6 brodmann area 30

5 brodmann area 23

5 brodmann area 29

1 Sub-Gyral

1 Corpus Callosum

----------------------

Cluster 146

Number of voxels: 12

Peak MNI coordinate: 0 -45 10.5

Peak MNI coordinate region: // Inter-Hemispheric // undefined // undefined // White Matter // Corpus Callosum // undefined

Peak intensity: -4.3709

# voxels structure

12 --TOTAL # VOXELS--

12 White Matter

12 Corpus Callosum

6 Right Cerebrum

6 Extra-Nuclear

6 Inter-Hemispheric

3 Sub-lobar

3 Limbic Lobe

3 Cingulum_Post_R (aal)

----------------------

Cluster 147

Number of voxels: 15

Peak MNI coordinate: 24 -18 9

Peak MNI coordinate region: // Right Cerebrum // Sub-lobar // Extra-Nuclear // White Matter // undefined // undefined

Peak intensity: -4.0462

# voxels structure

15 --TOTAL # VOXELS--

15 Right Cerebrum

15 Sub-lobar

15 White Matter

15 Extra-Nuclear

1 Thalamus_R (aal)

----------------------

Cluster 148

Number of voxels: 18

Peak MNI coordinate: -1.5 22.5 12

Peak MNI coordinate region: // Left Cerebrum // Sub-lobar // Extra-Nuclear // White Matter // Corpus Callosum // undefined

Peak intensity: -4.3943

# voxels structure

18 --TOTAL # VOXELS--

18 White Matter

18 Corpus Callosum

12 Extra-Nuclear

12 Sub-lobar

7 Left Cerebrum

6 Inter-Hemispheric

5 Right Cerebrum

2 Cingulum_Ant_L (aal)

----------------------

Cluster 149

Number of voxels: 12

Peak MNI coordinate: 57 -48 10.5

Peak MNI coordinate region: // Right Cerebrum // Temporal Lobe // Superior Temporal Gyrus // Gray Matter // brodmann area 22 // Temporal_Mid_R (aal)

Peak intensity: -4.3571

# voxels structure

12 --TOTAL # VOXELS--

12 Right Cerebrum

12 Superior Temporal Gyrus

12 Temporal Lobe

11 Temporal_Mid_R (aal)

7 Gray Matter

7 brodmann area 22

5 White Matter

1 Temporal_Sup_R (aal)

----------------------

Cluster 150

Number of voxels: 71

Peak MNI coordinate: 4.5 -58.5 22.5

Peak MNI coordinate region: // Right Cerebrum // Limbic Lobe // Posterior Cingulate // Gray Matter // brodmann area 31 // Precuneus_R (aal)

Peak intensity: -4.8184

# voxels structure

71 --TOTAL # VOXELS--

71 Precuneus_R (aal)

71 Right Cerebrum

65 Limbic Lobe

55 Posterior Cingulate

42 Gray Matter

29 White Matter

22 brodmann area 31

10 Cingulate Gyrus

9 brodmann area 23

7 brodmann area 30

6 Precuneus

6 Parietal Lobe

4 brodmann area 29

----------------------

Cluster 151

Number of voxels: 23

Peak MNI coordinate: 12 64.5 10.5

Peak MNI coordinate region: // Right Cerebrum // Frontal Lobe // Superior Frontal Gyrus // White Matter // undefined // Frontal_Sup_Medial_R (aal)

Peak intensity: -4.1331

# voxels structure

23 --TOTAL # VOXELS--

23 Frontal_Sup_Medial_R (aal)

22 Right Cerebrum

22 Frontal Lobe

20 Superior Frontal Gyrus

15 White Matter

4 brodmann area 10

4 Gray Matter

2 Medial Frontal Gyrus

----------------------

Cluster 152

Number of voxels: 17

Peak MNI coordinate: 21 69 12

Peak MNI coordinate region: // Right Cerebrum // Frontal Lobe // Middle Frontal Gyrus // undefined // undefined // undefined

Peak intensity: -3.7841

# voxels structure

17 --TOTAL # VOXELS--

17 Frontal Lobe

17 Right Cerebrum

12 Frontal_Sup_R (aal)

12 brodmann area 10

12 Gray Matter

11 Superior Frontal Gyrus

6 Middle Frontal Gyrus

----------------------

Cluster 153

Number of voxels: 17

Peak MNI coordinate: -43.5 -82.5 21

Peak MNI coordinate region: // Left Cerebrum // Temporal Lobe // Middle Temporal Gyrus // Gray Matter // brodmann area 39 // Occipital_Mid_L (aal)

Peak intensity: -4.3365

# voxels structure

17 --TOTAL # VOXELS--

17 Left Cerebrum

16 Occipital_Mid_L (aal)

11 Gray Matter

10 Middle Temporal Gyrus

10 Temporal Lobe

9 brodmann area 19

7 Occipital Lobe

7 Middle Occipital Gyrus

6 White Matter

2 brodmann area 39

----------------------

Cluster 154

Number of voxels: 23

Peak MNI coordinate: -34.5 -81 15

Peak MNI coordinate region: // Left Cerebrum // Occipital Lobe // Middle Temporal Gyrus // White Matter // undefined // Occipital_Mid_L (aal)

Peak intensity: -3.9062

# voxels structure

23 --TOTAL # VOXELS--

23 Left Cerebrum

23 Occipital_Mid_L (aal)

22 White Matter

14 Middle Temporal Gyrus

12 Occipital Lobe

11 Temporal Lobe

9 Middle Occipital Gyrus

1 brodmann area 19

1 Gray Matter

----------------------

Cluster 155

Number of voxels: 41

Peak MNI coordinate: -57 -69 16.5

Peak MNI coordinate region: // Left Cerebrum // Temporal Lobe // Middle Temporal Gyrus // Gray Matter // brodmann area 39 // undefined

Peak intensity: -4.1696

# voxels structure

41 --TOTAL # VOXELS--

41 Left Cerebrum

38 Middle Temporal Gyrus

29 Temporal Lobe

25 Gray Matter

18 brodmann area 39

12 Occipital Lobe

10 White Matter

7 Occipital_Mid_L (aal)

7 brodmann area 19

6 Temporal_Mid_L (aal)

3 Superior Temporal Gyrus

----------------------

Cluster 156

Number of voxels: 19

Peak MNI coordinate: 10.5 -43.5 15

Peak MNI coordinate region: // Right Cerebrum // Sub-lobar // Extra-Nuclear // White Matter // Corpus Callosum // Cingulum_Post_R (aal)

Peak intensity: -3.6711

# voxels structure

19 --TOTAL # VOXELS--

19 Right Cerebrum

19 Sub-lobar

17 Corpus Callosum

17 Extra-Nuclear

17 White Matter

11 Cingulum_Post_R (aal)

5 Precuneus_R (aal)

2 Lateral Ventricle

2 Cerebro-Spinal Fluid

----------------------

Cluster 157

Number of voxels: 13

Peak MNI coordinate: 45 -6 13.5

Peak MNI coordinate region: // Right Cerebrum // Sub-lobar // Insula // White Matter // undefined // Rolandic_Oper_R (aal)

Peak intensity: -4.5241

# voxels structure

13 --TOTAL # VOXELS--

13 Right Cerebrum

13 Rolandic_Oper_R (aal)

13 Sub-lobar

11 Insula

9 White Matter

3 brodmann area 13

3 Gray Matter

2 Extra-Nuclear

----------------------

Cluster 158

Number of voxels: 48

Peak MNI coordinate: -15 7.5 10.5

Peak MNI coordinate region: // Left Cerebrum // Sub-lobar // Extra-Nuclear // White Matter // undefined // undefined

Peak intensity: -3.9843

# voxels structure

48 --TOTAL # VOXELS--

48 Left Cerebrum

48 Sub-lobar

37 Caudate_L (aal)

29 White Matter

29 Extra-Nuclear

19 Gray Matter

18 Caudate Body

18 Caudate

1 Putamen

1 Lentiform Nucleus

----------------------

Cluster 159

Number of voxels: 18

Peak MNI coordinate: 3 49.5 12

Peak MNI coordinate region: // Right Cerebrum // Limbic Lobe // Anterior Cingulate // undefined // undefined // Cingulum_Ant_R (aal)

Peak intensity: -4.3012

# voxels structure

18 --TOTAL # VOXELS--

18 Cingulum_Ant_R (aal)

18 Right Cerebrum

15 Frontal Lobe

14 Medial Frontal Gyrus

10 Gray Matter

9 brodmann area 10

5 White Matter

3 Limbic Lobe

3 Anterior Cingulate

1 brodmann area 32

----------------------

Cluster 160

Number of voxels: 10

Peak MNI coordinate: -30 -94.5 13.5

Peak MNI coordinate region: // Left Cerebrum // Occipital Lobe // Middle Occipital Gyrus // White Matter // undefined // Occipital_Mid_L (aal)

Peak intensity: -3.5545

# voxels structure

10 --TOTAL # VOXELS--

10 Left Cerebrum

10 Middle Occipital Gyrus

10 Occipital Lobe

10 Occipital_Mid_L (aal)

8 White Matter

2 brodmann area 19

2 Gray Matter

----------------------

Cluster 161

Number of voxels: 12

Peak MNI coordinate: -66 -42 15

Peak MNI coordinate region: // Left Cerebrum // Temporal Lobe // Superior Temporal Gyrus // Gray Matter // brodmann area 22 // Temporal_Sup_L (aal)

Peak intensity: -3.2463

# voxels structure

12 --TOTAL # VOXELS--

12 Gray Matter

12 Left Cerebrum

12 Superior Temporal Gyrus

12 Temporal Lobe

12 Temporal_Sup_L (aal)

12 brodmann area 22

----------------------

Cluster 162

Number of voxels: 11

Peak MNI coordinate: -18 -24 15

Peak MNI coordinate region: // Left Cerebrum // Sub-lobar // Thalamus // Gray Matter // Pulvinar // undefined

Peak intensity: -5.007

# voxels structure

11 --TOTAL # VOXELS--

11 Sub-lobar

11 Left Cerebrum

8 Thalamus

8 Gray Matter

7 Pulvinar

3 White Matter

3 Extra-Nuclear

2 Thalamus_L (aal)

1 Lateral Posterior Nucleus

----------------------

Cluster 163

Number of voxels: 11

Peak MNI coordinate: -16.5 -88.5 15

Peak MNI coordinate region: // Left Cerebrum // Occipital Lobe // Cuneus // White Matter // undefined // Occipital_Sup_L (aal)

Peak intensity: -3.4305

# voxels structure

11 --TOTAL # VOXELS--

11 Left Cerebrum

11 Occipital Lobe

7 Occipital_Sup_L (aal)

7 Cuneus

6 Gray Matter

5 White Matter

4 Middle Occipital Gyrus

3 brodmann area 18

3 Cuneus_L (aal)

1 Occipital_Mid_L (aal)

----------------------

Cluster 164

Number of voxels: 20

Peak MNI coordinate: -42 16.5 15

Peak MNI coordinate region: // Left Cerebrum // Frontal Lobe // Sub-Gyral // White Matter // undefined // Frontal_Inf_Oper_L (aal)

Peak intensity: -3.7912

# voxels structure

20 --TOTAL # VOXELS--

20 Left Cerebrum

19 Frontal Lobe

19 White Matter

18 Frontal_Inf_Oper_L (aal)

17 Sub-Gyral

2 Frontal_Inf_Tri_L (aal)

2 Inferior Frontal Gyrus

1 Sub-lobar

1 Gray Matter

1 brodmann area 45

1 Extra-Nuclear

----------------------

Cluster 165

Number of voxels: 19

Peak MNI coordinate: 58.5 -57 15

Peak MNI coordinate region: // Right Cerebrum // Temporal Lobe // Superior Temporal Gyrus // White Matter // undefined // Temporal_Mid_R (aal)

Peak intensity: -3.9094

# voxels structure

19 --TOTAL # VOXELS--

19 Right Cerebrum

19 Superior Temporal Gyrus

19 Temporal Lobe

19 Temporal_Mid_R (aal)

19 White Matter

----------------------

Cluster 166

Number of voxels: 12

Peak MNI coordinate: -16.5 -18 16.5

Peak MNI coordinate region: // Left Cerebrum // Sub-lobar // Thalamus // Gray Matter // Ventral Lateral Nucleus // Thalamus_L (aal)

Peak intensity: -3.9935

# voxels structure

12 --TOTAL # VOXELS--

12 Left Cerebrum

12 Sub-lobar

10 Gray Matter

10 Thalamus

10 Ventral Lateral Nucleus

8 Thalamus_L (aal)

2 White Matter

2 Extra-Nuclear

----------------------

Cluster 167

Number of voxels: 13

Peak MNI coordinate: -45 -9 16.5

Peak MNI coordinate region: // Left Cerebrum // Parietal Lobe // Sub-Gyral // White Matter // undefined // Rolandic_Oper_L (aal)

Peak intensity: -3.5395

# voxels structure

13 --TOTAL # VOXELS--

13 Left Cerebrum

13 White Matter

12 Parietal Lobe

11 Rolandic_Oper_L (aal)

10 Sub-Gyral

2 Insula

2 Postcentral_L (aal)

1 Postcentral Gyrus

1 Sub-lobar

----------------------

Cluster 168

Number of voxels: 32

Peak MNI coordinate: -45 -1.5 19.5

Peak MNI coordinate region: // Left Cerebrum // Frontal Lobe // Sub-Gyral // White Matter // undefined // Precentral_L (aal)

Peak intensity: -5.3495

# voxels structure

32 --TOTAL # VOXELS--

32 Left Cerebrum

32 Sub-Gyral

32 White Matter

32 Frontal Lobe

14 Precentral_L (aal)

9 Rolandic_Oper_L (aal)

----------------------

Cluster 169

Number of voxels: 10

Peak MNI coordinate: 48 13.5 16.5

Peak MNI coordinate region: // Right Cerebrum // Frontal Lobe // Sub-Gyral // White Matter // undefined // Frontal_Inf_Oper_R (aal)

Peak intensity: -3.5607

# voxels structure

10 --TOTAL # VOXELS--

10 Frontal Lobe

10 Right Cerebrum

9 Sub-Gyral

9 White Matter

9 Frontal_Inf_Oper_R (aal)

1 Gray Matter

1 brodmann area 44

1 Inferior Frontal Gyrus

----------------------

Cluster 170

Number of voxels: 10

Peak MNI coordinate: -1.5 -69 16.5

Peak MNI coordinate region: // Left Cerebrum // Occipital Lobe // Precuneus // Gray Matter // brodmann area 31 // Calcarine_L (aal)

Peak intensity: -3.9856

# voxels structure

10 --TOTAL # VOXELS--

10 Left Cerebrum

10 Precuneus

9 Gray Matter

9 brodmann area 31

8 Calcarine_L (aal)

5 Occipital Lobe

5 Parietal Lobe

2 Cuneus_L (aal)

1 White Matter

----------------------

Cluster 171

Number of voxels: 27

Peak MNI coordinate: -13.5 -69 18

Peak MNI coordinate region: // Left Cerebrum // Occipital Lobe // Cuneus // undefined // undefined // Calcarine_L (aal)

Peak intensity: -4.1948

# voxels structure

27 --TOTAL # VOXELS--

27 Left Cerebrum

24 Precuneus

19 Occipital Lobe

18 Cuneus_L (aal)

12 brodmann area 31

12 Gray Matter

9 Calcarine_L (aal)

8 Parietal Lobe

8 White Matter

3 Cuneus

----------------------

Cluster 172

Number of voxels: 13

Peak MNI coordinate: -45 -42 21

Peak MNI coordinate region: // Left Cerebrum // Parietal Lobe // Inferior Parietal Lobule // White Matter // undefined // Temporal_Sup_L (aal)

Peak intensity: -3.3598

# voxels structure

13 --TOTAL # VOXELS--

13 Temporal_Sup_L (aal)

13 Left Cerebrum

7 Insula

7 Sub-lobar

6 White Matter

5 Inferior Parietal Lobule

5 Parietal Lobe

3 brodmann area 13

3 Gray Matter

1 Superior Temporal Gyrus

1 Temporal Lobe

----------------------

Cluster 173

Number of voxels: 19

Peak MNI coordinate: -55.5 -37.5 18

Peak MNI coordinate region: // Left Cerebrum // Sub-lobar // Insula // Gray Matter // brodmann area 13 // Temporal_Sup_L (aal)

Peak intensity: -4.0143

# voxels structure

19 --TOTAL # VOXELS--

19 Left Cerebrum

19 Temporal_Sup_L (aal)

12 Insula

12 Sub-lobar

9 White Matter

7 Temporal Lobe

7 Superior Temporal Gyrus

6 brodmann area 13

6 Gray Matter

----------------------

Cluster 174

Number of voxels: 43

Peak MNI coordinate: 64.5 -31.5 18

Peak MNI coordinate region: // Right Cerebrum // Temporal Lobe // Superior Temporal Gyrus // Gray Matter // brodmann area 42 // Temporal_Sup_R (aal)

Peak intensity: -3.6537

# voxels structure

43 --TOTAL # VOXELS--

43 Right Cerebrum

38 Parietal Lobe

27 SupraMarginal_R (aal)

24 White Matter

24 Inferior Parietal Lobule

17 Gray Matter

16 Temporal_Sup_R (aal)

14 Postcentral Gyrus

13 brodmann area 40

5 Superior Temporal Gyrus

5 Temporal Lobe

4 brodmann area 42

----------------------

Cluster 175

Number of voxels: 20

Peak MNI coordinate: 51 -22.5 16.5

Peak MNI coordinate region: // Right Cerebrum // Sub-lobar // Insula // Gray Matter // brodmann area 40 // Rolandic_Oper_R (aal)

Peak intensity: -3.698

# voxels structure

20 --TOTAL # VOXELS--

20 Right Cerebrum

17 Rolandic_Oper_R (aal)

15 Parietal Lobe

15 White Matter

14 Postcentral Gyrus

5 Sub-lobar

5 Gray Matter

5 brodmann area 40

5 Insula

3 SupraMarginal_R (aal)

1 Sub-Gyral

----------------------

Cluster 176

Number of voxels: 13

Peak MNI coordinate: 4.5 22.5 16.5

Peak MNI coordinate region: // Right Cerebrum // Sub-lobar // Extra-Nuclear // White Matter // Corpus Callosum // undefined

Peak intensity: -3.3862

# voxels structure

13 --TOTAL # VOXELS--

13 White Matter

13 Corpus Callosum

10 Extra-Nuclear

10 Sub-lobar

8 Right Cerebrum

4 Left Cerebrum

3 Cingulum_Ant_L (aal)

1 Inter-Hemispheric

1 Cingulum_Ant_R (aal)

----------------------

Cluster 177

Number of voxels: 10

Peak MNI coordinate: 7.5 -87 21

Peak MNI coordinate region: // Right Cerebrum // Occipital Lobe // Cuneus // White Matter // undefined // Cuneus_R (aal)

Peak intensity: -3.6806

# voxels structure

10 --TOTAL # VOXELS--

10 Cuneus

10 Cuneus_R (aal)

10 Occipital Lobe

10 Right Cerebrum

8 White Matter

2 brodmann area 18

2 Gray Matter

----------------------

Cluster 178

Number of voxels: 14

Peak MNI coordinate: 3 -66 19.5

Peak MNI coordinate region: // Right Cerebrum // Parietal Lobe // Precuneus // Gray Matter // brodmann area 31 // Cuneus_R (aal)

Peak intensity: -3.6765

# voxels structure

14 --TOTAL # VOXELS--

14 Gray Matter

14 Precuneus

14 Right Cerebrum

11 Parietal Lobe

11 brodmann area 31

9 Cuneus_R (aal)

3 Occipital Lobe

3 brodmann area 23

3 Precuneus_R (aal)

2 Calcarine_R (aal)

----------------------

Cluster 179

Number of voxels: 58

Peak MNI coordinate: 9 -46.5 30

Peak MNI coordinate region: // Right Cerebrum // Limbic Lobe // Cingulate Gyrus // Gray Matter // brodmann area 31 // Cingulum_Post_R (aal)

Peak intensity: -4.8812

# voxels structure

58 --TOTAL # VOXELS--

58 Right Cerebrum

46 Limbic Lobe

42 Cingulate Gyrus

35 Cingulum_Post_R (aal)

28 brodmann area 31

28 Gray Matter

26 White Matter

13 Cingulum_Mid_R (aal)

10 Sub-lobar

9 Extra-Nuclear

7 Posterior Cingulate

4 Precuneus_R (aal)

2 Parietal Lobe

1 Corpus Callosum

----------------------

Cluster 180

Number of voxels: 155

Peak MNI coordinate: 63 -39 30

Peak MNI coordinate region: // Right Cerebrum // Parietal Lobe // Inferior Parietal Lobule // Gray Matter // brodmann area 40 // SupraMarginal_R (aal)

Peak intensity: -4.5921

# voxels structure

155 --TOTAL # VOXELS--

155 Right Cerebrum

152 Parietal Lobe

148 Inferior Parietal Lobule

133 SupraMarginal_R (aal)

100 White Matter

55 Gray Matter

54 brodmann area 40

6 Rolandic_Oper_R (aal)

3 Insula

3 Sub-lobar

2 Supramarginal Gyrus

2 Sub-Gyral

1 Temporal_Sup_R (aal)

1 brodmann area 13

----------------------

Cluster 181

Number of voxels: 28

Peak MNI coordinate: -61.5 -7.5 27

Peak MNI coordinate region: // Left Cerebrum // Frontal Lobe // Precentral Gyrus // White Matter // undefined // Postcentral_L (aal)

Peak intensity: -4.8802

# voxels structure

28 --TOTAL # VOXELS--

28 Left Cerebrum

28 Postcentral_L (aal)

16 Parietal Lobe

15 Postcentral Gyrus

14 Gray Matter

13 Precentral Gyrus

12 White Matter

12 Frontal Lobe

10 brodmann area 43

4 brodmann area 4

----------------------

Cluster 182

Number of voxels: 26

Peak MNI coordinate: 18 -66 19.5

Peak MNI coordinate region: // Right Cerebrum // Temporal Lobe // Precuneus // White Matter // undefined // Cuneus_R (aal)

Peak intensity: -3.9578

# voxels structure

26 --TOTAL # VOXELS--

26 Precuneus

26 Right Cerebrum

21 White Matter

19 Cuneus_R (aal)

13 Parietal Lobe

11 Occipital Lobe

7 Precuneus_R (aal)

5 brodmann area 31

5 Gray Matter

2 Temporal Lobe

----------------------

Cluster 183

Number of voxels: 32

Peak MNI coordinate: -3 -69 30

Peak MNI coordinate region: // Left Cerebrum // Occipital Lobe // Cuneus // Gray Matter // brodmann area 7 // Cuneus_L (aal)

Peak intensity: -3.9501

# voxels structure

32 --TOTAL # VOXELS--

32 Left Cerebrum

25 Precuneus

21 Gray Matter

20 Cuneus_L (aal)

17 Occipital Lobe

15 Parietal Lobe

13 brodmann area 31

9 White Matter

8 Calcarine_L (aal)

7 brodmann area 7

7 Cuneus

4 Precuneus_L (aal)

1 brodmann area 23

----------------------

Cluster 184

Number of voxels: 14

Peak MNI coordinate: 21 -57 21

Peak MNI coordinate region: // Right Cerebrum // Parietal Lobe // Sub-Gyral // White Matter // undefined // Precuneus_R (aal)

Peak intensity: -3.5907

# voxels structure

14 --TOTAL # VOXELS--

14 Right Cerebrum

14 White Matter

13 Precuneus_R (aal)

13 Sub-Gyral

11 Parietal Lobe

2 Occipital Lobe

1 Sub-lobar

1 Extra-Nuclear

1 Cuneus_R (aal)

----------------------

Cluster 185

Number of voxels: 40

Peak MNI coordinate: 28.5 -79.5 24

Peak MNI coordinate region: // Right Cerebrum // Occipital Lobe // Sub-Gyral // White Matter // undefined // Occipital_Sup_R (aal)

Peak intensity: -3.8769

# voxels structure

40 --TOTAL # VOXELS--

40 Right Cerebrum

35 White Matter

32 Occipital Lobe

31 Occipital_Sup_R (aal)

22 Cuneus

18 Sub-Gyral

9 Occipital_Mid_R (aal)

8 Temporal Lobe

5 brodmann area 19

5 Gray Matter

----------------------

Cluster 186

Number of voxels: 16

Peak MNI coordinate: 10.5 -78 22.5

Peak MNI coordinate region: // Right Cerebrum // Occipital Lobe // Cuneus // Gray Matter // brodmann area 18 // Cuneus_R (aal)

Peak intensity: -3.7801

# voxels structure

16 --TOTAL # VOXELS--

16 Cuneus_R (aal)

16 Right Cerebrum

15 Occipital Lobe

15 Cuneus

11 Gray Matter

11 brodmann area 18

2 White Matter

1 Parietal Lobe

1 Precuneus

----------------------

Cluster 187

Number of voxels: 10

Peak MNI coordinate: -46.5 -63 21

Peak MNI coordinate region: // Left Cerebrum // Temporal Lobe // Middle Temporal Gyrus // White Matter // undefined // Temporal_Mid_L (aal)

Peak intensity: -3.7012

# voxels structure

10 --TOTAL # VOXELS--

10 Left Cerebrum

10 Middle Temporal Gyrus

10 Temporal Lobe

10 Temporal_Mid_L (aal)

6 Gray Matter

6 brodmann area 39

4 White Matter

----------------------

Cluster 188

Number of voxels: 106

Peak MNI coordinate: 7.5 46.5 37.5

Peak MNI coordinate region: // Right Cerebrum // Frontal Lobe // Medial Frontal Gyrus // White Matter // undefined // Frontal_Sup_Medial_R (aal)

Peak intensity: -4.3447

# voxels structure

106 --TOTAL # VOXELS--

106 Frontal Lobe

106 Right Cerebrum

101 Frontal_Sup_Medial_R (aal)

68 White Matter

55 Medial Frontal Gyrus

51 Superior Frontal Gyrus

37 Gray Matter

24 brodmann area 9

11 brodmann area 8

5 Frontal_Sup_R (aal)

2 brodmann area 6

----------------------

Cluster 189

Number of voxels: 13

Peak MNI coordinate: -36 -87 24

Peak MNI coordinate region: // Left Cerebrum // Occipital Lobe // Superior Occipital Gyrus // Gray Matter // brodmann area 19 // Occipital_Mid_L (aal)

Peak intensity: -3.7939

# voxels structure

13 --TOTAL # VOXELS--

13 Left Cerebrum

13 Occipital_Mid_L (aal)

12 Occipital Lobe

12 Superior Occipital Gyrus

8 Gray Matter

8 brodmann area 19

3 White Matter

1 Temporal Lobe

1 Middle Temporal Gyrus

----------------------

Cluster 190

Number of voxels: 33

Peak MNI coordinate: -3 -79.5 27

Peak MNI coordinate region: // Left Cerebrum // Occipital Lobe // Cuneus // Gray Matter // brodmann area 19 // Cuneus_L (aal)

Peak intensity: -5.2782

# voxels structure

33 --TOTAL # VOXELS--

33 Cuneus_L (aal)

33 Left Cerebrum

33 Occipital Lobe

26 Cuneus

20 Gray Matter

11 brodmann area 18

6 Precuneus

5 brodmann area 31

4 brodmann area 19

1 White Matter

----------------------

Cluster 191

Number of voxels: 31

Peak MNI coordinate: -6 -58.5 25.5

Peak MNI coordinate region: // Left Cerebrum // Limbic Lobe // Cingulate Gyrus // Gray Matter // brodmann area 31 // Precuneus_L (aal)

Peak intensity: -4.0032

# voxels structure

31 --TOTAL # VOXELS--

30 Limbic Lobe

27 Left Cerebrum

22 Gray Matter

22 Precuneus_L (aal)

20 brodmann area 31

19 Cingulate Gyrus

11 Posterior Cingulate

6 Cingulum_Post_L (aal)

4 Right Cerebrum

3 Precuneus_R (aal)

2 White Matter

2 brodmann area 23

1 Precuneus

1 Occipital Lobe

----------------------

Cluster 192

Number of voxels: 16

Peak MNI coordinate: 4.5 -72 25.5

Peak MNI coordinate region: // Right Cerebrum // Occipital Lobe // Precuneus // Gray Matter // brodmann area 31 // Cuneus_R (aal)

Peak intensity: -4.3424

# voxels structure

16 --TOTAL # VOXELS--

16 Occipital Lobe

16 Right Cerebrum

15 Precuneus

14 Gray Matter

14 Cuneus_R (aal)

13 brodmann area 31

2 White Matter

2 Cuneus_L (aal)

1 brodmann area 7

1 Cuneus

----------------------

Cluster 193

Number of voxels: 17

Peak MNI coordinate: -3 -46.5 27

Peak MNI coordinate region: // Left Cerebrum // Limbic Lobe // Cingulate Gyrus // White Matter // undefined // Cingulum_Post_L (aal)

Peak intensity: -3.8187

# voxels structure

17 --TOTAL # VOXELS--

17 Cingulum_Post_L (aal)

17 Left Cerebrum

17 Limbic Lobe

16 Cingulate Gyrus

15 White Matter

2 brodmann area 31

2 Gray Matter

1 Posterior Cingulate

----------------------

Cluster 194

Number of voxels: 14

Peak MNI coordinate: 52.5 -10.5 27

Peak MNI coordinate region: // Right Cerebrum // Frontal Lobe // Precentral Gyrus // White Matter // undefined // Postcentral_R (aal)

Peak intensity: -3.948

# voxels structure

14 --TOTAL # VOXELS--

14 Right Cerebrum

14 White Matter

14 Frontal Lobe

13 Precentral Gyrus

12 Postcentral_R (aal)

1 Postcentral Gyrus

----------------------

Cluster 195

Number of voxels: 14

Peak MNI coordinate: -12 46.5 25.5

Peak MNI coordinate region: // Left Cerebrum // Frontal Lobe // Medial Frontal Gyrus // White Matter // undefined // Frontal_Sup_L (aal)

Peak intensity: -3.4984

# voxels structure

14 --TOTAL # VOXELS--

14 Frontal Lobe

14 Left Cerebrum

13 White Matter

12 Medial Frontal Gyrus

8 Frontal_Sup_Medial_L (aal)

6 Frontal_Sup_L (aal)

2 Superior Frontal Gyrus

1 brodmann area 9

1 Gray Matter

----------------------

Cluster 196

Number of voxels: 16

Peak MNI coordinate: -45 -78 30

Peak MNI coordinate region: // Left Cerebrum // Temporal Lobe // Angular Gyrus // Gray Matter // brodmann area 39 // Angular_L (aal)

Peak intensity: -3.9884

# voxels structure

16 --TOTAL # VOXELS--

16 Left Cerebrum

16 Temporal Lobe

14 Angular Gyrus

13 Occipital_Mid_L (aal)

10 Gray Matter

10 brodmann area 39

3 White Matter

2 Middle Temporal Gyrus

2 Angular_L (aal)

----------------------

Cluster 197

Number of voxels: 12

Peak MNI coordinate: -51 -40.5 28.5

Peak MNI coordinate region: // Left Cerebrum // Parietal Lobe // Inferior Parietal Lobule // White Matter // undefined // SupraMarginal_L (aal)

Peak intensity: -3.7656

# voxels structure

12 --TOTAL # VOXELS--

12 Inferior Parietal Lobule

12 Left Cerebrum

12 Parietal Lobe

12 SupraMarginal_L (aal)

12 White Matter

----------------------

Cluster 198

Number of voxels: 15

Peak MNI coordinate: -48 -31.5 30

Peak MNI coordinate region: // Left Cerebrum // Parietal Lobe // Inferior Parietal Lobule // White Matter // undefined // SupraMarginal_L (aal)

Peak intensity: -3.7225

# voxels structure

15 --TOTAL # VOXELS--

15 Inferior Parietal Lobule

15 Left Cerebrum

15 Parietal Lobe

15 SupraMarginal_L (aal)

13 White Matter

2 brodmann area 40

2 Gray Matter

----------------------

Cluster 199

Number of voxels: 11

Peak MNI coordinate: 34.5 -24 31.5

Peak MNI coordinate region: // Right Cerebrum // Frontal Lobe // Sub-Gyral // White Matter // undefined // undefined

Peak intensity: -3.3649

# voxels structure

11 --TOTAL # VOXELS--

11 Right Cerebrum

11 White Matter

11 Frontal Lobe

7 Sub-Gyral

4 Postcentral Gyrus

----------------------

Cluster 200

Number of voxels: 10

Peak MNI coordinate: -24 -84 30

Peak MNI coordinate region: // Left Cerebrum // Occipital Lobe // Cuneus // Gray Matter // brodmann area 7 // Occipital_Sup_L (aal)

Peak intensity: -3.4272

# voxels structure

10 --TOTAL # VOXELS--

10 Cuneus

10 Left Cerebrum

10 Occipital Lobe

8 Occipital_Sup_L (aal)

7 Gray Matter

4 brodmann area 19

3 White Matter

3 brodmann area 7

2 Occipital_Mid_L (aal)

----------------------

Cluster 201

Number of voxels: 19

Peak MNI coordinate: -52.5 -66 34.5

Peak MNI coordinate region: // Left Cerebrum // Parietal Lobe // Angular Gyrus // White Matter // undefined // Angular_L (aal)

Peak intensity: -3.7502

# voxels structure

19 --TOTAL # VOXELS--

15 Left Cerebrum

14 Angular Gyrus

14 Angular_L (aal)

13 Parietal Lobe

9 brodmann area 39

9 Gray Matter

6 White Matter

2 Temporal Lobe

2 Occipital_Mid_L (aal)

1 Middle Temporal Gyrus

----------------------

Cluster 202

Number of voxels: 14

Peak MNI coordinate: 12 4.5 28.5

Peak MNI coordinate region: // Right Cerebrum // Limbic Lobe // Cingulate Gyrus // White Matter // undefined // undefined

Peak intensity: -3.8362

# voxels structure

14 --TOTAL # VOXELS--

14 Limbic Lobe

14 Right Cerebrum

14 White Matter

14 Cingulate Gyrus

----------------------

Cluster 203

Number of voxels: 29

Peak MNI coordinate: -6 51 30

Peak MNI coordinate region: // Left Cerebrum // Frontal Lobe // Superior Frontal Gyrus // Gray Matter // brodmann area 9 // Frontal_Sup_Medial_L (aal)

Peak intensity: -4.9083

# voxels structure

29 --TOTAL # VOXELS--

29 Frontal Lobe

29 Frontal_Sup_Medial_L (aal)

29 Left Cerebrum

25 Superior Frontal Gyrus

24 Gray Matter

24 brodmann area 9

5 White Matter

4 Medial Frontal Gyrus

----------------------

Cluster 204

Number of voxels: 10

Peak MNI coordinate: -57 9 34.5

Peak MNI coordinate region: // Left Cerebrum // Frontal Lobe // Inferior Frontal Gyrus // Gray Matter // brodmann area 9 // Precentral_L (aal)

Peak intensity: -3.5593

# voxels structure

10 --TOTAL # VOXELS--

10 Frontal Lobe

10 Inferior Frontal Gyrus

10 Left Cerebrum

10 Precentral_L (aal)

9 Gray Matter

9 brodmann area 9

1 White Matter

----------------------

Cluster 205

Number of voxels: 31

Peak MNI coordinate: 4.5 31.5 31.5

Peak MNI coordinate region: // Right Cerebrum // Limbic Lobe // Cingulate Gyrus // Gray Matter // brodmann area 32 // Cingulum_Mid_R (aal)

Peak intensity: -3.5008

# voxels structure

31 --TOTAL # VOXELS--

31 Right Cerebrum

26 Cingulum_Mid_R (aal)

23 Gray Matter

17 Limbic Lobe

17 Cingulate Gyrus

16 brodmann area 32

14 Medial Frontal Gyrus

14 Frontal Lobe

7 brodmann area 9

6 White Matter

----------------------

Cluster 206

Number of voxels: 15

Peak MNI coordinate: -43.5 -73.5 33

Peak MNI coordinate region: // Left Cerebrum // Parietal Lobe // Precuneus // Gray Matter // brodmann area 39 // Occipital_Mid_L (aal)

Peak intensity: -3.9265

# voxels structure

15 --TOTAL # VOXELS--

15 Left Cerebrum

14 Parietal Lobe

13 Angular Gyrus

12 Gray Matter

12 brodmann area 39

5 Occipital_Mid_L (aal)

5 Angular_L (aal)

3 White Matter

2 Precuneus

1 Temporal Lobe

----------------------

Cluster 207

Number of voxels: 14

Peak MNI coordinate: 49.5 -67.5 37.5

Peak MNI coordinate region: // Right Cerebrum // Parietal Lobe // Inferior Parietal Lobule // Gray Matter // brodmann area 39 // Angular_R (aal)

Peak intensity: -3.4681

# voxels structure

14 --TOTAL # VOXELS--

14 Angular_R (aal)

14 Parietal Lobe

14 Right Cerebrum

9 Angular Gyrus

7 Gray Matter

7 White Matter

7 brodmann area 39

5 Inferior Parietal Lobule

----------------------

Cluster 208

Number of voxels: 27

Peak MNI coordinate: 64.5 -31.5 31.5

Peak MNI coordinate region: // Right Cerebrum // Parietal Lobe // Inferior Parietal Lobule // White Matter // undefined // SupraMarginal_R (aal)

Peak intensity: -4.812

# voxels structure

27 --TOTAL # VOXELS--

27 SupraMarginal_R (aal)

24 Right Cerebrum

24 Inferior Parietal Lobule

24 Parietal Lobe

12 White Matter

8 brodmann area 40

8 Gray Matter

----------------------

Cluster 209

Number of voxels: 44

Peak MNI coordinate: 42 6 33

Peak MNI coordinate region: // Right Cerebrum // Frontal Lobe // Inferior Frontal Gyrus // White Matter // undefined // Precentral_R (aal)

Peak intensity: -4.8018

# voxels structure

44 --TOTAL # VOXELS--

44 Frontal Lobe

44 Right Cerebrum

43 White Matter

35 Inferior Frontal Gyrus

22 Precentral_R (aal)

20 Frontal_Inf_Oper_R (aal)

9 Middle Frontal Gyrus

2 Frontal_Mid_R (aal)

1 brodmann area 9

1 Gray Matter

----------------------

Cluster 210

Number of voxels: 12

Peak MNI coordinate: -48 21 36

Peak MNI coordinate region: // Left Cerebrum // Frontal Lobe // Middle Frontal Gyrus // White Matter // undefined // Frontal_Mid_L (aal)

Peak intensity: -3.3355

# voxels structure

12 --TOTAL # VOXELS--

12 Frontal Lobe

12 Left Cerebrum

12 Middle Frontal Gyrus

12 White Matter

11 Frontal_Mid_L (aal)

1 Frontal_Inf_Oper_L (aal)

----------------------

Cluster 211

Number of voxels: 11

Peak MNI coordinate: 27 -87 33

Peak MNI coordinate region: // Right Cerebrum // Occipital Lobe // Cuneus // Gray Matter // brodmann area 19 // Occipital_Sup_R (aal)

Peak intensity: -3.3984

# voxels structure

11 --TOTAL # VOXELS--

11 Occipital_Sup_R (aal)

10 Right Cerebrum

7 brodmann area 19

7 Gray Matter

6 Precuneus

6 Parietal Lobe

4 Cuneus

4 Occipital Lobe

----------------------

Cluster 212

Number of voxels: 38

Peak MNI coordinate: -15 -73.5 37.5

Peak MNI coordinate region: // Left Cerebrum // Parietal Lobe // Precuneus // White Matter // undefined // Cuneus_L (aal)

Peak intensity: -3.7121

# voxels structure

38 --TOTAL # VOXELS--

38 Left Cerebrum

38 Parietal Lobe

38 Precuneus

32 White Matter

22 Occipital_Sup_L (aal)

13 Cuneus_L (aal)

6 Gray Matter

4 brodmann area 7

2 Precuneus_L (aal)

2 brodmann area 19

1 Parietal_Sup_L (aal)

----------------------

Cluster 213

Number of voxels: 178

Peak MNI coordinate: -49.5 -61.5 40.5

Peak MNI coordinate region: // Left Cerebrum // Parietal Lobe // Inferior Parietal Lobule // Gray Matter // brodmann area 40 // Angular_L (aal)

Peak intensity: -5.1529

# voxels structure

178 --TOTAL # VOXELS--

167 Parietal Lobe

167 Left Cerebrum

148 Angular_L (aal)

120 Inferior Parietal Lobule

115 White Matter

52 Gray Matter

37 brodmann area 40

36 Angular Gyrus

28 Parietal_Inf_L (aal)

9 Supramarginal Gyrus

8 brodmann area 7

7 brodmann area 39

2 Superior Parietal Lobule

----------------------

Cluster 214

Number of voxels: 30

Peak MNI coordinate: 6 -27 34.5

Peak MNI coordinate region: // Right Cerebrum // Limbic Lobe // Cingulate Gyrus // White Matter // undefined // Cingulum_Mid_R (aal)

Peak intensity: -4.379

# voxels structure

30 --TOTAL # VOXELS--

30 Cingulate Gyrus

30 Cingulum_Mid_R (aal)

30 Limbic Lobe

30 Right Cerebrum

16 Gray Matter

14 White Matter

13 brodmann area 23

2 brodmann area 31

1 brodmann area 24

----------------------

Cluster 215

Number of voxels: 472

Peak MNI coordinate: -28.5 -45 70.5

Peak MNI coordinate region: // Left Cerebrum // Parietal Lobe // Postcentral Gyrus // Gray Matter // brodmann area 5 // Parietal_Sup_L (aal)

Peak intensity: -4.5719

# voxels structure

472 --TOTAL # VOXELS--

340 Parietal_Sup_L (aal)

129 Left Cerebrum

129 Parietal Lobe

83 Precuneus

73 White Matter

64 Precuneus_L (aal)

47 Gray Matter

29 brodmann area 7

28 Postcentral Gyrus

18 brodmann area 5

15 Superior Parietal Lobule

6 Postcentral_L (aal)

5 Occipital_Sup_L (aal)

3 Parietal_Inf_L (aal)

2 Occipital_Mid_L (aal)

----------------------

Cluster 216

Number of voxels: 11

Peak MNI coordinate: 51 -48 37.5

Peak MNI coordinate region: // Right Cerebrum // Parietal Lobe // Supramarginal Gyrus // White Matter // undefined // Parietal_Inf_R (aal)

Peak intensity: -3.5704

# voxels structure

11 --TOTAL # VOXELS--

11 Parietal Lobe

11 Right Cerebrum

11 White Matter

8 Parietal_Inf_R (aal)

8 Supramarginal Gyrus

3 SupraMarginal_R (aal)

3 Inferior Parietal Lobule

----------------------

Cluster 217

Number of voxels: 11

Peak MNI coordinate: 21 -48 37.5

Peak MNI coordinate region: // Right Cerebrum // Parietal Lobe // Sub-Gyral // White Matter // undefined // undefined

Peak intensity: -4.2618

# voxels structure

11 --TOTAL # VOXELS--

11 Right Cerebrum

10 White Matter

9 Sub-Gyral

7 Parietal Lobe

3 Frontal Lobe

1 Gray Matter

1 Limbic Lobe

1 Cingulate Gyrus

1 brodmann area 31

1 Precuneus

----------------------

Cluster 218

Number of voxels: 20

Peak MNI coordinate: 1.5 -43.5 39

Peak MNI coordinate region: // Right Cerebrum // Limbic Lobe // Cingulate Gyrus // undefined // undefined // Precuneus_R (aal)

Peak intensity: -3.7275

# voxels structure

20 --TOTAL # VOXELS--

18 Limbic Lobe

18 Cingulate Gyrus

13 Right Cerebrum

8 Cingulum_Mid_R (aal)

5 Precuneus_R (aal)

5 Left Cerebrum

5 Precuneus_L (aal)

3 brodmann area 31

3 Gray Matter

2 Inter-Hemispheric

2 Cingulum_Mid_L (aal)

----------------------

Cluster 219

Number of voxels: 104

Peak MNI coordinate: 60 -24 36

Peak MNI coordinate region: // Right Cerebrum // Parietal Lobe // Postcentral Gyrus // White Matter // undefined // SupraMarginal_R (aal)

Peak intensity: -4.5858

# voxels structure

104 --TOTAL # VOXELS--

91 Right Cerebrum

73 Parietal Lobe

72 Postcentral Gyrus

71 SupraMarginal_R (aal)

53 Gray Matter

33 Postcentral_R (aal)

19 brodmann area 2

18 Frontal Lobe

18 White Matter

17 Precentral Gyrus

13 brodmann area 3

11 brodmann area 1

9 brodmann area 4

1 brodmann area 6

----------------------

Cluster 220

Number of voxels: 47

Peak MNI coordinate: 52.5 -15 36

Peak MNI coordinate region: // Right Cerebrum // Frontal Lobe // Precentral Gyrus // White Matter // undefined // Postcentral_R (aal)

Peak intensity: -3.9793

# voxels structure

47 --TOTAL # VOXELS--

47 Frontal Lobe

47 Precentral Gyrus

47 Right Cerebrum

42 White Matter

32 Postcentral_R (aal)

15 Precentral_R (aal)

4 Gray Matter

3 brodmann area 6

1 brodmann area 4

----------------------

Cluster 221

Number of voxels: 242

Peak MNI coordinate: -55.5 -6 51

Peak MNI coordinate region: // undefined // undefined // undefined // undefined // undefined // undefined

Peak intensity: -4.5815

# voxels structure

242 --TOTAL # VOXELS--

93 Left Cerebrum

80 Frontal Lobe

75 Precentral Gyrus

52 Gray Matter

50 Postcentral_L (aal)

49 brodmann area 6

16 White Matter

14 Postcentral Gyrus

13 Parietal Lobe

11 Precentral_L (aal)

4 Middle Frontal Gyrus

2 brodmann area 1

1 brodmann area 3

----------------------

Cluster 222

Number of voxels: 21

Peak MNI coordinate: 28.5 -49.5 37.5

Peak MNI coordinate region: // Right Cerebrum // Parietal Lobe // Sub-Gyral // White Matter // undefined // undefined

Peak intensity: -3.3259

# voxels structure

21 --TOTAL # VOXELS--

21 Right Cerebrum

21 Parietal Lobe

18 White Matter

16 Sub-Gyral

8 Angular_R (aal)

3 Gray Matter

3 brodmann area 7

3 Precuneus

2 Inferior Parietal Lobule

----------------------

Cluster 223

Number of voxels: 53

Peak MNI coordinate: 10.5 25.5 37.5

Peak MNI coordinate region: // Right Cerebrum // Frontal Lobe // Medial Frontal Gyrus // Gray Matter // brodmann area 6 // Cingulum_Mid_R (aal)

Peak intensity: -3.8571

# voxels structure

53 --TOTAL # VOXELS--

53 Right Cerebrum

52 Frontal Lobe

46 Medial Frontal Gyrus

26 Frontal_Sup_R (aal)

24 Gray Matter

24 Cingulum_Mid_R (aal)

23 White Matter

19 brodmann area 6

7 Cingulate Gyrus

5 brodmann area 9

1 Frontal_Sup_Medial_R (aal)

1 Limbic Lobe

----------------------

Cluster 224

Number of voxels: 21

Peak MNI coordinate: -54 -45 39

Peak MNI coordinate region: // Left Cerebrum // Parietal Lobe // Inferior Parietal Lobule // White Matter // undefined // Parietal_Inf_L (aal)

Peak intensity: -3.5785

# voxels structure

21 --TOTAL # VOXELS--

21 Left Cerebrum

21 Parietal Lobe

21 Parietal_Inf_L (aal)

20 Inferior Parietal Lobule

15 Gray Matter

15 brodmann area 40

6 White Matter

1 Supramarginal Gyrus

----------------------

Cluster 225

Number of voxels: 13

Peak MNI coordinate: -66 -25.5 39

Peak MNI coordinate region: // undefined // undefined // undefined // undefined // undefined // undefined

Peak intensity: -3.2753

# voxels structure

13 --TOTAL # VOXELS--

2 SupraMarginal_L (aal)

----------------------

Cluster 226

Number of voxels: 13

Peak MNI coordinate: 33 -58.5 40.5

Peak MNI coordinate region: // Right Cerebrum // Parietal Lobe // Sub-Gyral // White Matter // undefined // Angular_R (aal)

Peak intensity: -3.014

# voxels structure

13 --TOTAL # VOXELS--

13 Angular_R (aal)

13 Parietal Lobe

13 Right Cerebrum

13 White Matter

11 Sub-Gyral

2 Inferior Parietal Lobule

----------------------

Cluster 227

Number of voxels: 14

Peak MNI coordinate: 12 -52.5 40.5

Peak MNI coordinate region: // Right Cerebrum // Parietal Lobe // Precuneus // Gray Matter // brodmann area 7 // Precuneus_R (aal)

Peak intensity: -3.7133

# voxels structure

14 --TOTAL # VOXELS--

14 Precuneus_R (aal)

14 Right Cerebrum

12 Gray Matter

11 Parietal Lobe

10 Precuneus

9 brodmann area 7

4 Cingulate Gyrus

3 brodmann area 31

3 Limbic Lobe

1 White Matter

----------------------

Cluster 228

Number of voxels: 29

Peak MNI coordinate: 33 7.5 42

Peak MNI coordinate region: // Right Cerebrum // Frontal Lobe // Middle Frontal Gyrus // White Matter // undefined // undefined

Peak intensity: -3.3985

# voxels structure

29 --TOTAL # VOXELS--

29 Right Cerebrum

29 White Matter

29 Frontal Lobe

19 Middle Frontal Gyrus

15 Frontal_Mid_R (aal)

10 Sub-Gyral

2 Frontal_Sup_R (aal)

----------------------

Cluster 229

Number of voxels: 21

Peak MNI coordinate: 58.5 -43.5 45

Peak MNI coordinate region: // Right Cerebrum // Parietal Lobe // Inferior Parietal Lobule // Gray Matter // brodmann area 40 // Parietal_Inf_R (aal)

Peak intensity: -3.7678

# voxels structure

21 --TOTAL # VOXELS--

21 Inferior Parietal Lobule

21 Parietal Lobe

21 Right Cerebrum

17 Gray Matter

17 brodmann area 40

13 SupraMarginal_R (aal)

8 Parietal_Inf_R (aal)

----------------------

Cluster 230

Number of voxels: 136

Peak MNI coordinate: 45 -42 51

Peak MNI coordinate region: // Right Cerebrum // Parietal Lobe // Inferior Parietal Lobule // White Matter // undefined // Parietal_Inf_R (aal)

Peak intensity: -5.477

# voxels structure

136 --TOTAL # VOXELS--

136 Inferior Parietal Lobule

136 Parietal Lobe

136 Right Cerebrum

110 Parietal_Inf_R (aal)

75 White Matter

61 brodmann area 40

61 Gray Matter

15 Parietal_Sup_R (aal)

11 SupraMarginal_R (aal)

----------------------

Cluster 231

Number of voxels: 10

Peak MNI coordinate: -55.5 -31.5 42

Peak MNI coordinate region: // Left Cerebrum // Parietal Lobe // Postcentral Gyrus // undefined // undefined // Parietal_Inf_L (aal)

Peak intensity: -3.0198

# voxels structure

10 --TOTAL # VOXELS--

10 Left Cerebrum

10 Parietal Lobe

10 Parietal_Inf_L (aal)

5 Gray Matter

5 Postcentral Gyrus

5 Inferior Parietal Lobule

3 brodmann area 2

2 brodmann area 40

----------------------

Cluster 232

Number of voxels: 13

Peak MNI coordinate: 48 7.5 45

Peak MNI coordinate region: // Right Cerebrum // Frontal Lobe // Middle Frontal Gyrus // Gray Matter // brodmann area 8 // Precentral_R (aal)

Peak intensity: -2.997

# voxels structure

13 --TOTAL # VOXELS--

13 Frontal Lobe

13 Middle Frontal Gyrus

13 Right Cerebrum

12 Precentral_R (aal)

8 Gray Matter

8 brodmann area 8

5 White Matter

1 Frontal_Mid_R (aal)

----------------------

Cluster 233

Number of voxels: 14

Peak MNI coordinate: -45 13.5 48

Peak MNI coordinate region: // Left Cerebrum // Frontal Lobe // Middle Frontal Gyrus // White Matter // undefined // Frontal_Mid_L (aal)

Peak intensity: -2.8955

# voxels structure

14 --TOTAL # VOXELS--

14 Frontal Lobe

14 Left Cerebrum

14 Middle Frontal Gyrus

12 White Matter

8 Precentral_L (aal)

6 Frontal_Mid_L (aal)

2 brodmann area 8

2 Gray Matter

----------------------

Cluster 234

Number of voxels: 45

Peak MNI coordinate: 25.5 -72 48

Peak MNI coordinate region: // Right Cerebrum // Parietal Lobe // Precuneus // undefined // undefined // Parietal_Sup_R (aal)

Peak intensity: -4.4484

# voxels structure

45 --TOTAL # VOXELS--

41 Parietal Lobe

41 Right Cerebrum

40 Parietal_Sup_R (aal)

30 brodmann area 7

30 Gray Matter

22 Superior Parietal Lobule

19 Precuneus

5 Occipital_Sup_R (aal)

2 White Matter

----------------------

Cluster 235

Number of voxels: 13

Peak MNI coordinate: 18 -64.5 45

Peak MNI coordinate region: // Right Cerebrum // Parietal Lobe // Precuneus // White Matter // undefined // Precuneus_R (aal)

Peak intensity: -3.8065

# voxels structure

13 --TOTAL # VOXELS--

13 Parietal Lobe

13 Precuneus

13 Precuneus_R (aal)

13 Right Cerebrum

13 White Matter

----------------------

Cluster 236

Number of voxels: 14

Peak MNI coordinate: 51 -58.5 46.5

Peak MNI coordinate region: // Right Cerebrum // Parietal Lobe // Inferior Parietal Lobule // Gray Matter // brodmann area 40 // Parietal_Inf_R (aal)

Peak intensity: -4.0765

# voxels structure

14 --TOTAL # VOXELS--

13 Parietal_Inf_R (aal)

12 Inferior Parietal Lobule

12 Parietal Lobe

12 Gray Matter

12 Right Cerebrum

12 brodmann area 40

1 Angular_R (aal)

----------------------

Cluster 237

Number of voxels: 33

Peak MNI coordinate: 4.5 -60 48

Peak MNI coordinate region: // Right Cerebrum // Parietal Lobe // Precuneus // Gray Matter // brodmann area 7 // Precuneus_R (aal)

Peak intensity: -4.4106

# voxels structure

33 --TOTAL # VOXELS--

33 Parietal Lobe

33 Precuneus

33 Precuneus_R (aal)

33 Right Cerebrum

25 Gray Matter

25 brodmann area 7

8 White Matter

----------------------

Cluster 238

Number of voxels: 19

Peak MNI coordinate: -61.5 -33 48

Peak MNI coordinate region: // undefined // undefined // undefined // undefined // undefined // undefined

Peak intensity: -3.7944

# voxels structure

19 --TOTAL # VOXELS--

----------------------

Cluster 239

Number of voxels: 307

Peak MNI coordinate: 9 -33 64.5

Peak MNI coordinate region: // Right Cerebrum // Frontal Lobe // Paracentral Lobule // White Matter // undefined // Paracentral_Lobule_R (aal)

Peak intensity: -4.666

# voxels structure

307 --TOTAL # VOXELS--

307 Right Cerebrum

217 Paracentral_Lobule_R (aal)

209 White Matter

203 Frontal Lobe

200 Paracentral Lobule

104 Parietal Lobe

94 Gray Matter

64 Sub-Gyral

46 brodmann area 4

41 Postcentral Gyrus

32 Postcentral_R (aal)

22 brodmann area 6

16 brodmann area 5

9 brodmann area 3

6 Cingulum_Mid_R (aal)

2 Medial Frontal Gyrus

1 brodmann area 7

1 Supp_Motor_Area_R (aal)

----------------------

Cluster 240

Number of voxels: 37

Peak MNI coordinate: -48 -9 52.5

Peak MNI coordinate region: // Left Cerebrum // Frontal Lobe // Precentral Gyrus // Gray Matter // brodmann area 4 // Postcentral_L (aal)

Peak intensity: -3.8983

# voxels structure

37 --TOTAL # VOXELS--

37 Frontal Lobe

37 Left Cerebrum

37 Precentral Gyrus

33 Postcentral_L (aal)

24 White Matter

13 Gray Matter

8 brodmann area 6

5 brodmann area 4

4 Precentral_L (aal)

----------------------

Cluster 241

Number of voxels: 409

Peak MNI coordinate: 16.5 -49.5 54

Peak MNI coordinate region: // Right Cerebrum // Parietal Lobe // Precuneus // White Matter // undefined // Parietal_Sup_R (aal)

Peak intensity: -4.5919

# voxels structure

409 --TOTAL # VOXELS--

257 Right Cerebrum

250 Parietal Lobe

212 Parietal_Sup_R (aal)

152 White Matter

100 Precuneus

100 Gray Matter

81 Sub-Gyral

68 brodmann area 7

62 Precuneus_R (aal)

44 Postcentral_R (aal)

29 Inferior Parietal Lobule

24 Superior Parietal Lobule

22 brodmann area 40

13 Postcentral Gyrus

12 Parietal_Inf_R (aal)

10 Paracentral Lobule

10 brodmann area 5

7 Frontal Lobe

----------------------

Cluster 242

Number of voxels: 11

Peak MNI coordinate: -36 -25.5 49.5

Peak MNI coordinate region: // Left Cerebrum // Parietal Lobe // Postcentral Gyrus // White Matter // undefined // Postcentral_L (aal)

Peak intensity: -3.0609

# voxels structure

11 --TOTAL # VOXELS--

11 Left Cerebrum

11 Postcentral_L (aal)

10 Postcentral Gyrus

10 Parietal Lobe

6 White Matter

5 Gray Matter

3 brodmann area 2

1 Precentral Gyrus

1 brodmann area 3

1 brodmann area 4

1 Frontal Lobe

----------------------

Cluster 243

Number of voxels: 31

Peak MNI coordinate: 52.5 -15 51

Peak MNI coordinate region: // Right Cerebrum // Parietal Lobe // Postcentral Gyrus // Gray Matter // brodmann area 3 // Postcentral_R (aal)

Peak intensity: -4.2584

# voxels structure

31 --TOTAL # VOXELS--

31 Right Cerebrum

26 Postcentral Gyrus

20 Gray Matter

19 Parietal Lobe

19 Postcentral_R (aal)

16 brodmann area 3

12 Precentral_R (aal)

12 Frontal Lobe

5 Precentral Gyrus

4 brodmann area 4

----------------------

Cluster 244

Number of voxels: 10

Peak MNI coordinate: -15 -67.5 51

Peak MNI coordinate region: // Left Cerebrum // Parietal Lobe // Precuneus // White Matter // undefined // Parietal_Sup_L (aal)

Peak intensity: -3.1969

# voxels structure

10 --TOTAL # VOXELS--

10 Left Cerebrum

10 Parietal Lobe

10 Precuneus

10 White Matter

9 Parietal_Sup_L (aal)

1 Precuneus_L (aal)

----------------------

Cluster 245

Number of voxels: 14

Peak MNI coordinate: -54 -42 52.5

Peak MNI coordinate region: // Left Cerebrum // Parietal Lobe // Inferior Parietal Lobule // Gray Matter // brodmann area 40 // Parietal_Inf_L (aal)

Peak intensity: -3.6742

# voxels structure

14 --TOTAL # VOXELS--

14 Inferior Parietal Lobule

14 Left Cerebrum

14 Parietal Lobe

14 Parietal_Inf_L (aal)

8 Gray Matter

8 brodmann area 40

6 White Matter

----------------------

Cluster 246

Number of voxels: 17

Peak MNI coordinate: 49.5 -27 51

Peak MNI coordinate region: // Right Cerebrum // Parietal Lobe // Postcentral Gyrus // White Matter // undefined // Postcentral_R (aal)

Peak intensity: -3.3533

# voxels structure

17 --TOTAL # VOXELS--

17 Parietal Lobe

17 Postcentral Gyrus

17 Postcentral_R (aal)

17 Right Cerebrum

11 White Matter

6 Gray Matter

4 brodmann area 2

2 brodmann area 1

----------------------

Cluster 247

Number of voxels: 30

Peak MNI coordinate: -43.5 -28.5 52.5

Peak MNI coordinate region: // Left Cerebrum // Parietal Lobe // Postcentral Gyrus // White Matter // undefined // Postcentral_L (aal)

Peak intensity: -4.2817

# voxels structure

30 --TOTAL # VOXELS--

30 Left Cerebrum

30 Parietal Lobe

30 Postcentral Gyrus

30 Postcentral_L (aal)

25 White Matter

5 Gray Matter

4 brodmann area 2

1 brodmann area 1

----------------------

Cluster 248

Number of voxels: 88

Peak MNI coordinate: -34.5 -16.5 61.5

Peak MNI coordinate region: // Left Cerebrum // Frontal Lobe // Precentral Gyrus // White Matter // undefined // Precentral_L (aal)

Peak intensity: -4.9906

# voxels structure

88 --TOTAL # VOXELS--

86 Precentral_L (aal)

80 Precentral Gyrus

80 Frontal Lobe

80 Left Cerebrum

63 White Matter

17 Gray Matter

16 brodmann area 4

1 brodmann area 6

----------------------

Cluster 249

Number of voxels: 76

Peak MNI coordinate: 4.5 22.5 54

Peak MNI coordinate region: // Right Cerebrum // Frontal Lobe // Superior Frontal Gyrus // Gray Matter // brodmann area 8 // Supp_Motor_Area_R (aal)

Peak intensity: -4.2707

# voxels structure

76 --TOTAL # VOXELS--

76 Frontal Lobe

76 Right Cerebrum

70 White Matter

64 Superior Frontal Gyrus

38 Supp_Motor_Area_R (aal)

35 Frontal_Sup_Medial_R (aal)

12 Medial Frontal Gyrus

6 brodmann area 8

6 Gray Matter

3 Frontal_Sup_R (aal)

----------------------

Cluster 250

Number of voxels: 10

Peak MNI coordinate: -46.5 -49.5 51

Peak MNI coordinate region: // Left Cerebrum // Parietal Lobe // Inferior Parietal Lobule // Gray Matter // brodmann area 40 // Parietal_Inf_L (aal)

Peak intensity: -3.0525

# voxels structure

10 --TOTAL # VOXELS--

10 Parietal_Inf_L (aal)

8 Parietal Lobe

8 Inferior Parietal Lobule

8 Left Cerebrum

6 brodmann area 40

6 Gray Matter

2 White Matter

----------------------

Cluster 251

Number of voxels: 19

Peak MNI coordinate: -30 -28.5 54

Peak MNI coordinate region: // Left Cerebrum // Frontal Lobe // Precentral Gyrus // White Matter // undefined // Precentral_L (aal)

Peak intensity: -3.8837

# voxels structure

19 --TOTAL # VOXELS--

19 Frontal Lobe

19 Left Cerebrum

19 Precentral Gyrus

17 White Matter

10 Precentral_L (aal)

7 Postcentral_L (aal)

2 brodmann area 4

2 Gray Matter

----------------------

Cluster 252

Number of voxels: 290

Peak MNI coordinate: 48 -18 58.5

Peak MNI coordinate region: // Right Cerebrum // Parietal Lobe // Postcentral Gyrus // Gray Matter // brodmann area 3 // Postcentral_R (aal)

Peak intensity: -5.2143

# voxels structure

290 --TOTAL # VOXELS--

175 Right Cerebrum

147 Postcentral_R (aal)

115 Parietal Lobe

110 Postcentral Gyrus

87 Gray Matter

70 Precentral_R (aal)

65 Precentral Gyrus

60 Frontal Lobe

58 brodmann area 3

47 White Matter

21 brodmann area 4

5 brodmann area 1

2 brodmann area 2

2 Frontal_Mid_R (aal)

1 brodmann area 6

----------------------

Cluster 253

Number of voxels: 38

Peak MNI coordinate: 15 -72 61.5

Peak MNI coordinate region: // undefined // undefined // undefined // undefined // undefined // Parietal_Sup_R (aal)

Peak intensity: -4.7856

# voxels structure

38 --TOTAL # VOXELS--

22 Parietal_Sup_R (aal)

16 Precuneus_R (aal)

13 Right Cerebrum

13 Superior Parietal Lobule

13 Parietal Lobe

12 brodmann area 7

12 Gray Matter

----------------------

Cluster 254

Number of voxels: 11

Peak MNI coordinate: -7.5 -51 55.5

Peak MNI coordinate region: // Left Cerebrum // Parietal Lobe // Precuneus // White Matter // undefined // Precuneus_L (aal)

Peak intensity: -3.6999

# voxels structure

11 --TOTAL # VOXELS--

11 Left Cerebrum

11 Parietal Lobe

11 Precuneus

11 Precuneus_L (aal)

6 White Matter

5 brodmann area 7

5 Gray Matter

----------------------

Cluster 255

Number of voxels: 12

Peak MNI coordinate: 40.5 -22.5 54

Peak MNI coordinate region: // Right Cerebrum // Parietal Lobe // Postcentral Gyrus // Gray Matter // brodmann area 3 // Precentral_R (aal)

Peak intensity: -3.6044

# voxels structure

12 --TOTAL # VOXELS--

12 Precentral_R (aal)

12 Right Cerebrum

10 Gray Matter

7 Frontal Lobe

7 Precentral Gyrus

5 Postcentral Gyrus

5 Parietal Lobe

5 brodmann area 3

5 brodmann area 4

----------------------

Cluster 256

Number of voxels: 68

Peak MNI coordinate: 24 -31.5 58.5

Peak MNI coordinate region: // Right Cerebrum // Parietal Lobe // Postcentral Gyrus // Gray Matter // brodmann area 3 // Postcentral_R (aal)

Peak intensity: -4.4759

# voxels structure

68 --TOTAL # VOXELS--

68 Right Cerebrum

61 Postcentral Gyrus

61 Parietal Lobe

45 Gray Matter

44 Postcentral_R (aal)

33 brodmann area 3

18 Precentral_R (aal)

13 White Matter

7 Precentral Gyrus

7 brodmann area 4

7 Frontal Lobe

3 brodmann area 1

2 brodmann area 2

----------------------

Cluster 257

Number of voxels: 31

Peak MNI coordinate: 34.5 -16.5 63

Peak MNI coordinate region: // Right Cerebrum // Frontal Lobe // Precentral Gyrus // White Matter // undefined // Precentral_R (aal)

Peak intensity: -3.4224

# voxels structure

31 --TOTAL # VOXELS--

31 Frontal Lobe

31 Precentral Gyrus

31 Precentral_R (aal)

31 Right Cerebrum

25 White Matter

6 brodmann area 6

6 Gray Matter

----------------------

Cluster 258

Number of voxels: 10

Peak MNI coordinate: 28.5 -3 60

Peak MNI coordinate region: // Right Cerebrum // Frontal Lobe // Sub-Gyral // White Matter // undefined // Frontal_Sup_R (aal)

Peak intensity: -3.5057

# voxels structure

10 --TOTAL # VOXELS--

10 Frontal_Sup_R (aal)

10 Right Cerebrum

10 Frontal Lobe

7 White Matter

7 Sub-Gyral

3 Middle Frontal Gyrus

1 brodmann area 6

1 Gray Matter

----------------------

Cluster 259

Number of voxels: 22

Peak MNI coordinate: -45 -25.5 61.5

Peak MNI coordinate region: // undefined // undefined // undefined // undefined // undefined // Postcentral_L (aal)

Peak intensity: -5.3557

# voxels structure

22 --TOTAL # VOXELS--

22 Postcentral_L (aal)

11 Postcentral Gyrus

11 Left Cerebrum

11 Parietal Lobe

7 brodmann area 3

7 Gray Matter

4 White Matter

----------------------

Cluster 260

Number of voxels: 10

Peak MNI coordinate: 40.5 4.5 63

Peak MNI coordinate region: // undefined // undefined // undefined // undefined // undefined // Frontal_Mid_R (aal)

Peak intensity: -3.4051

# voxels structure

10 --TOTAL # VOXELS--

8 Frontal_Mid_R (aal)

7 Gray Matter

7 Middle Frontal Gyrus

7 Right Cerebrum

7 brodmann area 6

7 Frontal Lobe

----------------------

Cluster 261

Number of voxels: 14

Peak MNI coordinate: -6 19.5 63

Peak MNI coordinate region: // Left Cerebrum // Frontal Lobe // Superior Frontal Gyrus // Gray Matter // brodmann area 6 // Supp_Motor_Area_L (aal)

Peak intensity: -3.2764

# voxels structure

14 --TOTAL # VOXELS--

14 Supp_Motor_Area_L (aal)

13 Superior Frontal Gyrus

13 Frontal Lobe

13 Left Cerebrum

9 White Matter

4 brodmann area 6

4 Gray Matter

----------------------

Cluster 262

Number of voxels: 12

Peak MNI coordinate: -30 -4.5 66

Peak MNI coordinate region: // Left Cerebrum // Frontal Lobe // Middle Frontal Gyrus // undefined // undefined // Frontal_Sup_L (aal)

Peak intensity: -2.9311

# voxels structure

12 --TOTAL # VOXELS--

12 Left Cerebrum

12 Middle Frontal Gyrus

12 Frontal Lobe

6 Frontal_Sup_L (aal)

3 Gray Matter

3 Precentral_L (aal)

3 White Matter

3 brodmann area 6

3 Frontal_Mid_L (aal)

----------------------

Cluster 263

Number of voxels: 14

Peak MNI coordinate: -40.5 -4.5 61.5

Peak MNI coordinate region: // Left Cerebrum // Frontal Lobe // Middle Frontal Gyrus // Gray Matter // brodmann area 6 // Precentral_L (aal)

Peak intensity: -3.8677

# voxels structure

14 --TOTAL # VOXELS--

14 Precentral_L (aal)

11 Frontal Lobe

11 Left Cerebrum

10 Middle Frontal Gyrus

6 brodmann area 6

6 Gray Matter

1 Precentral Gyrus

----------------------

Cluster 264

Number of voxels: 13

Peak MNI coordinate: 9 31.5 61.5

Peak MNI coordinate region: // Right Cerebrum // Frontal Lobe // Superior Frontal Gyrus // Gray Matter // brodmann area 6 // Frontal_Sup_Medial_R (aal)

Peak intensity: -4.6057

# voxels structure

13 --TOTAL # VOXELS--

12 Frontal Lobe

12 Frontal_Sup_Medial_R (aal)

12 Right Cerebrum

12 Superior Frontal Gyrus

10 Gray Matter

10 brodmann area 6

2 White Matter

----------------------

Cluster 265

Number of voxels: 23

Peak MNI coordinate: -22.5 0 72

Peak MNI coordinate region: // undefined // undefined // undefined // undefined // undefined // Frontal_Sup_L (aal)

Peak intensity: -3.868

# voxels structure

23 --TOTAL # VOXELS--

23 Frontal_Sup_L (aal)

21 Left Cerebrum

21 Frontal Lobe

11 Superior Frontal Gyrus

10 Middle Frontal Gyrus

7 White Matter

5 brodmann area 6

5 Gray Matter

----------------------

Cluster 266

Number of voxels: 21

Peak MNI coordinate: 7.5 1.5 64.5

Peak MNI coordinate region: // Right Cerebrum // Frontal Lobe // Medial Frontal Gyrus // undefined // undefined // Supp_Motor_Area_R (aal)

Peak intensity: -3.9205

# voxels structure

21 --TOTAL # VOXELS--

21 Supp_Motor_Area_R (aal)

15 Frontal Lobe

15 Right Cerebrum

10 Superior Frontal Gyrus

8 brodmann area 6

8 Gray Matter

6 Inter-Hemispheric

5 Medial Frontal Gyrus

----------------------

Cluster 267

Number of voxels: 65

Peak MNI coordinate: 25.5 15 64.5

Peak MNI coordinate region: // undefined // undefined // undefined // undefined // undefined // Frontal_Sup_R (aal)

Peak intensity: -4.2125

# voxels structure

65 --TOTAL # VOXELS--

42 Frontal_Sup_R (aal)

26 Right Cerebrum

26 Frontal Lobe

16 brodmann area 6

16 Gray Matter

14 Middle Frontal Gyrus

12 Superior Frontal Gyrus

5 White Matter

----------------------

Cluster 268

Number of voxels: 31

Peak MNI coordinate: -6 21 67.5

Peak MNI coordinate region: // undefined // undefined // undefined // undefined // undefined // Supp_Motor_Area_L (aal)

Peak intensity: -4.8885

# voxels structure

31 --TOTAL # VOXELS--

24 Supp_Motor_Area_L (aal)

7 Left Cerebrum

7 Superior Frontal Gyrus

7 Frontal Lobe

7 brodmann area 6

7 Gray Matter

----------------------

Cluster 269

Number of voxels: 21

Peak MNI coordinate: -4.5 -57 66

Peak MNI coordinate region: // Inter-Hemispheric // undefined // undefined // undefined // undefined // Precuneus_L (aal)

Peak intensity: -3.5082

# voxels structure

21 --TOTAL # VOXELS--

21 Precuneus_L (aal)

16 Left Cerebrum

16 Parietal Lobe

14 Postcentral Gyrus

7 brodmann area 7

7 Gray Matter

5 Inter-Hemispheric

3 White Matter

2 Precuneus

----------------------

Cluster 270

Number of voxels: 17

Peak MNI coordinate: 34.5 -48 67.5

Peak MNI coordinate region: // undefined // undefined // undefined // undefined // undefined // Postcentral_R (aal)

Peak intensity: -3.5696

# voxels structure

17 --TOTAL # VOXELS--

9 Postcentral_R (aal)

7 Parietal_Sup_R (aal)

----------------------

Cluster 271

Number of voxels: 16

Peak MNI coordinate: 33 -42 67.5

Peak MNI coordinate region: // undefined // undefined // undefined // undefined // undefined // Postcentral_R (aal)

Peak intensity: -3.6598

# voxels structure

16 --TOTAL # VOXELS--

16 Postcentral_R (aal)

3 Parietal Lobe

3 Right Cerebrum

3 Postcentral Gyrus

2 brodmann area 5

2 Gray Matter

----------------------

Cluster 272

Number of voxels: 48

Peak MNI coordinate: 16.5 -40.5 75

Peak MNI coordinate region: // Right Cerebrum // Parietal Lobe // Postcentral Gyrus // White Matter // undefined // Postcentral_R (aal)

Peak intensity: -4.1481

# voxels structure

48 --TOTAL # VOXELS--

48 Postcentral_R (aal)

40 Parietal Lobe

40 Right Cerebrum

40 Postcentral Gyrus

24 White Matter

11 Gray Matter

7 brodmann area 3

4 brodmann area 7

----------------------

Cluster 273

Number of voxels: 14

Peak MNI coordinate: 27 -46.5 70.5

Peak MNI coordinate region: // Right Cerebrum // Parietal Lobe // Postcentral Gyrus // Gray Matter // brodmann area 5 // Postcentral_R (aal)

Peak intensity: -3.209

# voxels structure

14 --TOTAL # VOXELS--

12 Postcentral_R (aal)

10 Parietal Lobe

10 Right Cerebrum

10 Postcentral Gyrus

9 brodmann area 5

9 Gray Matter

2 Parietal_Sup_R (aal)

----------------------

Cluster 274

Number of voxels: 31

Peak MNI coordinate: 12 16.5 69

Peak MNI coordinate region: // undefined // undefined // undefined // undefined // undefined // Supp_Motor_Area_R (aal)

Peak intensity: -4.0316

# voxels structure

31 --TOTAL # VOXELS--

17 Supp_Motor_Area_R (aal)

8 Frontal_Sup_R (aal)

5 Right Cerebrum

5 Superior Frontal Gyrus

5 Frontal Lobe

5 brodmann area 6

5 Gray Matter

----------------------

Cluster 275

Number of voxels: 16

Peak MNI coordinate: -16.5 -45 76.5

Peak MNI coordinate region: // undefined // undefined // undefined // undefined // undefined // Parietal_Sup_L (aal)

Peak intensity: -3.7828

# voxels structure

16 --TOTAL # VOXELS--

12 Parietal_Sup_L (aal)

4 Left Cerebrum

4 Postcentral Gyrus

4 Parietal Lobe

3 brodmann area 7

3 Gray Matter

2 Postcentral_L (aal)

2 Precuneus_L (aal)

1 White Matter

----------------------

Cluster 276

Number of voxels: 27

Peak MNI coordinate: 16.5 -22.5 75

Peak MNI coordinate region: // Right Cerebrum // Frontal Lobe // Precentral Gyrus // Gray Matter // brodmann area 6 // Precentral_R (aal)

Peak intensity: -4.2125

# voxels structure

27 --TOTAL # VOXELS--

27 Frontal Lobe

27 Precentral Gyrus

27 Right Cerebrum

24 Precentral_R (aal)

14 brodmann area 6

14 Gray Matter

13 White Matter

----------------------

Cluster 277

Number of voxels: 24

Peak MNI coordinate: -30 -18 73.5

Peak MNI coordinate region: // undefined // undefined // undefined // undefined // undefined // Precentral_L (aal)

Peak intensity: -4.0918

# voxels structure

24 --TOTAL # VOXELS--

24 Precentral_L (aal)

3 Precentral Gyrus

3 Frontal Lobe

3 Left Cerebrum

2 brodmann area 6

2 Gray Matter

----------------------

Cluster 278

Number of voxels: 43

Peak MNI coordinate: -13.5 -12 79.5

Peak MNI coordinate region: // Left Cerebrum // Frontal Lobe // Superior Frontal Gyrus // undefined // undefined // Precentral_L (aal)

Peak intensity: -4.8979

# voxels structure

43 --TOTAL # VOXELS--

33 Left Cerebrum

33 Frontal Lobe

23 Superior Frontal Gyrus

22 Precentral_L (aal)

16 brodmann area 6

16 Gray Matter

10 Frontal_Sup_L (aal)

10 Precentral Gyrus

8 Paracentral_Lobule_L (aal)

----------------------

Cluster 279

Number of voxels: 50

Peak MNI coordinate: -7.5 10.5 72

Peak MNI coordinate region: // undefined // undefined // undefined // undefined // undefined // Supp_Motor_Area_L (aal)

Peak intensity: -4.1984

# voxels structure

50 --TOTAL # VOXELS--

48 Supp_Motor_Area_L (aal)

37 Superior Frontal Gyrus

37 Frontal Lobe

37 Left Cerebrum

21 brodmann area 6

21 Gray Matter

16 White Matter

----------------------

Cluster 280

Number of voxels: 11

Peak MNI coordinate: -19.5 -43.5 79.5

Peak MNI coordinate region: // undefined // undefined // undefined // undefined // undefined // Postcentral_L (aal)

Peak intensity: -3.7988

# voxels structure

11 --TOTAL # VOXELS--

8 Postcentral_L (aal)

3 Parietal_Sup_L (aal)

----------------------

Cluster 281

Number of voxels: 11

Peak MNI coordinate: 15 -36 82.5

Peak MNI coordinate region: // Right Cerebrum // Parietal Lobe // Postcentral Gyrus // White Matter // undefined // Postcentral_R (aal)

Peak intensity: -3.896

# voxels structure

11 --TOTAL # VOXELS--

11 Postcentral_R (aal)

8 Parietal Lobe

8 Right Cerebrum

8 Postcentral Gyrus

6 White Matter

2 brodmann area 7

2 Gray Matter

>>

xx **spmDens-anova-nosim-noprof-onlyPos-afterFDR-MonoBi**

**Clusters >= 10**

/Users/uhroot/Desktop/Hannah/Density/DensityANOVA/nosim/spmT_0001.nii,1

Type: T

df: 324

Threshold

-- p value = 1.1836e-05

-- intensity = 4.2894

-- cluster size = 10

Number of clusters found: 3

----------------------

Cluster 1

Number of voxels: 14

Peak MNI coordinate: 24 -36 -48

Peak MNI coordinate region: // Right Cerebellum // Cerebellum Posterior Lobe // Cerebellar Tonsil // undefined // undefined // undefined

Peak intensity: 7.0322

# voxels structure

14 --TOTAL # VOXELS--

12 Cerebellum Posterior Lobe

12 Right Cerebellum

12 Cerebellar Tonsil

8 Cerebelum_10_R (aal)

1 Cerebelum_8_R (aal)

----------------------

Cluster 2

Number of voxels: 10

Peak MNI coordinate: 39 -19.5 -15

Peak MNI coordinate region: // Right Cerebrum // Temporal Lobe // Sub-Gyral // White Matter // undefined // Hippocampus_R (aal)

Peak intensity: 5.2506

# voxels structure

10 --TOTAL # VOXELS--

10 Hippocampus_R (aal)

10 Right Cerebrum

8 Temporal Lobe

8 White Matter

8 Sub-Gyral

2 Cerebro-Spinal Fluid

2 Lateral Ventricle

2 Sub-lobar

----------------------

Cluster 3

Number of voxels: 10

Peak MNI coordinate: -18 -64.5 18

Peak MNI coordinate region: // Left Cerebrum // Temporal Lobe // Sub-Gyral // White Matter // undefined // Calcarine_L (aal)

Peak intensity: 6.0404

# voxels structure

10 --TOTAL # VOXELS--

10 White Matter

10 Left Cerebrum

9 Sub-Gyral

8 Temporal Lobe

6 Calcarine_L (aal)

2 Occipital Lobe

1 Precuneus

>>

**B.4 ANCOVA: Monolingual vs. bilingual contrast, controlling for English proficiency**

*Contrast: Monolingual vs. bilingual. “Peak intensity” indicates *t* value at cluster peak, where positive/negative sign indicates direction of relationship (positive: Monolingual > bilingual; Negative: Monolingual < bilingual). Results rendered using xjview toolbox.

***Monolingual < Bilingual***

Type: T

df: 323

Threshold

-- p value = 0.0012263

-- intensity = 3.0532

-- cluster size = 10

Number of clusters found: 175

----------------------

Cluster 1

Number of voxels: 11

Peak MNI coordinate: -4.5 -48 -57

Peak MNI coordinate region: // undefined // undefined // undefined // undefined // undefined // undefined

Peak intensity: -3.421

# voxels structure

11 --TOTAL # VOXELS--

8 Cerebelum_9_L (aal)

5 Medulla

5 Left Brainstem

1 Left Cerebellum

1 Cerebellar Tonsil

1 Cerebellum Posterior Lobe

----------------------

Cluster 2

Number of voxels: 34

Peak MNI coordinate: -21 -78 -55.5

Peak MNI coordinate region: // undefined // undefined // undefined // undefined // undefined // undefined

Peak intensity: -4.1804

# voxels structure

34 --TOTAL # VOXELS--

6 Inferior Semi-Lunar Lobule

6 Left Cerebellum

6 Cerebellum Posterior Lobe

----------------------

Cluster 3

Number of voxels: 240

Peak MNI coordinate: 18 -88.5 -43.5

Peak MNI coordinate region: // undefined // undefined // undefined // undefined // undefined // undefined

Peak intensity: -6.3623

# voxels structure

240 --TOTAL # VOXELS--

86 Cerebelum_Crus2_R (aal)

75 Right Cerebellum

75 Cerebellum Posterior Lobe

46 Pyramis

24 Tuber

4 Inferior Semi-Lunar Lobule

3 Cerebelum_7b_R (aal)

1 Uvula

----------------------

Cluster 4

Number of voxels: 77

Peak MNI coordinate: -10.5 -82.5 -51

Peak MNI coordinate region: // undefined // undefined // undefined // undefined // undefined // undefined

Peak intensity: -4.5411

# voxels structure

77 --TOTAL # VOXELS--

----------------------

Cluster 5

Number of voxels: 41

Peak MNI coordinate: 34.5 -37.5 -46.5

Peak MNI coordinate region: // Right Cerebellum // Cerebellum Posterior Lobe // Cerebellar Tonsil // undefined // undefined // Cerebelum_8_R (aal)

Peak intensity: -4.4215

# voxels structure

41 --TOTAL # VOXELS--

41 Cerebellum Posterior Lobe

41 Right Cerebellum

41 Cerebellar Tonsil

24 Cerebelum_8_R (aal)

----------------------

Cluster 6

Number of voxels: 2772

Peak MNI coordinate: 42 22.5 -37.5

Peak MNI coordinate region: // undefined // undefined // undefined // undefined // undefined // Temporal_Pole_Mid_R (aal)

Peak intensity: -6.6015

# voxels structure

2772 --TOTAL # VOXELS--

2002 Right Cerebrum

1494 Frontal Lobe

1260 Gray Matter

742 Middle Frontal Gyrus

715 Frontal_Mid_R (aal)

605 Inferior Frontal Gyrus

474 Temporal Lobe

395 Frontal_Inf_Tri_R (aal)

359 White Matter

331 Superior Temporal Gyrus

326 Temporal_Pole_Mid_R (aal)

303 Temporal_Pole_Sup_R (aal)

264 Frontal_Inf_Orb_R (aal)

250 brodmann area 46

215 brodmann area 38

203 brodmann area 9

136 brodmann area 8

132 Middle Temporal Gyrus

126 Superior Frontal Gyrus

122 brodmann area 10

116 Temporal_Mid_R (aal)

115 brodmann area 45

96 brodmann area 47

91 Temporal_Sup_R (aal)

69 brodmann area 21

68 Precentral_R (aal)

43 Frontal_Inf_Oper_R (aal)

36 Frontal_Sup_R (aal)

29 Temporal_Inf_R (aal)

26 brodmann area 22

11 Fusiform_R (aal)

9 brodmann area 6

9 Frontal_Sup_Orb_R (aal)

6 Rectal Gyrus

6 Frontal_Mid_Orb_R (aal)

5 brodmann area 44

5 Sub-Gyral

4 Inferior Temporal Gyrus

3 brodmann area 42

3 Precentral Gyrus

3 ParaHippocampal_R (aal)

2 Rolandic_Oper_R (aal)

1 Subcallosal Gyrus

1 brodmann area 11

----------------------

Cluster 7

Number of voxels: 12

Peak MNI coordinate: -24 -33 -48

Peak MNI coordinate region: // Left Cerebellum // Cerebellum Posterior Lobe // Cerebellar Tonsil // undefined // undefined // Cerebelum_10_L (aal)

Peak intensity: -4.0934

# voxels structure

12 --TOTAL # VOXELS--

11 Cerebelum_10_L (aal)

8 Cerebellar Tonsil

8 Left Cerebellum

8 Cerebellum Posterior Lobe

----------------------

Cluster 8

Number of voxels: 34

Peak MNI coordinate: -30 12 -46.5

Peak MNI coordinate region: // undefined // undefined // undefined // undefined // undefined // undefined

Peak intensity: -4.5798

# voxels structure

34 --TOTAL # VOXELS--

25 Superior Temporal Gyrus

25 Temporal Lobe

25 Left Cerebrum

22 brodmann area 38

22 Gray Matter

3 White Matter

----------------------

Cluster 9

Number of voxels: 39

Peak MNI coordinate: -22.5 -88.5 -45

Peak MNI coordinate region: // Left Cerebellum // Cerebellum Posterior Lobe // Pyramis // undefined // undefined // undefined

Peak intensity: -4.0351

# voxels structure

39 --TOTAL # VOXELS--

27 Left Cerebellum

27 Cerebellum Posterior Lobe

26 Pyramis

1 Tuber

----------------------

Cluster 10

Number of voxels: 2008

Peak MNI coordinate: -46.5 18 -36

Peak MNI coordinate region: // undefined // undefined // undefined // undefined // undefined // Temporal_Pole_Mid_L (aal)

Peak intensity: -7.3377

# voxels structure

2008 --TOTAL # VOXELS--

1579 Left Cerebrum

1114 Frontal Lobe

950 Gray Matter

585 Middle Frontal Gyrus

479 Frontal_Mid_L (aal)

453 Inferior Frontal Gyrus

433 Temporal Lobe

410 Frontal_Inf_Tri_L (aal)

403 White Matter

257 Superior Temporal Gyrus

193 Temporal_Pole_Sup_L (aal)

183 brodmann area 38

180 brodmann area 46

168 Middle Temporal Gyrus

152 Temporal_Pole_Mid_L (aal)

137 brodmann area 9

126 Temporal_Mid_L (aal)

118 Frontal_Inf_Orb_L (aal)

110 brodmann area 45

98 brodmann area 8

94 brodmann area 21

75 Superior Frontal Gyrus

69 brodmann area 10

37 brodmann area 47

31 Temporal_Sup_L (aal)

30 brodmann area 22

21 Frontal_Sup_L (aal)

10 Inferior Temporal Gyrus

6 Frontal_Inf_Oper_L (aal)

5 Temporal_Inf_L (aal)

3 brodmann area 44

2 Frontal_Sup_Orb_L (aal)

----------------------

Cluster 11

Number of voxels: 10

Peak MNI coordinate: -9 -64.5 -42

Peak MNI coordinate region: // Left Cerebellum // Cerebellum Posterior Lobe // Uvula // undefined // undefined // Cerebelum_8_L (aal)

Peak intensity: -4.582

# voxels structure

10 --TOTAL # VOXELS--

10 Cerebelum_8_L (aal)

10 Left Cerebellum

10 Cerebellum Posterior Lobe

9 Uvula

1 Inferior Semi-Lunar Lobule

----------------------

Cluster 12

Number of voxels: 78

Peak MNI coordinate: -31.5 -27 -31.5

Peak MNI coordinate region: // undefined // undefined // undefined // undefined // undefined // Cerebelum_4_5_L (aal)

Peak intensity: -4.7667

# voxels structure

78 --TOTAL # VOXELS--

44 Left Cerebellum

33 Cerebellum Anterior Lobe

33 Culmen

27 Cerebelum_4_5_L (aal)

19 Cerebelum_6_L (aal)

11 Cerebellum Posterior Lobe

11 Cerebellar Tonsil

9 Cerebelum_10_L (aal)

5 Fusiform_L (aal)

----------------------

Cluster 13

Number of voxels: 15

Peak MNI coordinate: -24 -81 -37.5

Peak MNI coordinate region: // Left Cerebellum // Cerebellum Posterior Lobe // Tuber // undefined // undefined // Cerebelum_Crus2_L (aal)

Peak intensity: -4.1414

# voxels structure

15 --TOTAL # VOXELS--

15 Cerebelum_Crus2_L (aal)

15 Left Cerebellum

15 Cerebellum Posterior Lobe

10 Tuber

5 Pyramis

----------------------

Cluster 14

Number of voxels: 11

Peak MNI coordinate: -3 -45 -39

Peak MNI coordinate region: // undefined // undefined // undefined // undefined // undefined // Cerebelum_9_L (aal)

Peak intensity: -4.2582

# voxels structure

11 --TOTAL # VOXELS--

10 Cerebelum_9_L (aal)

4 Left Cerebellum

4 Cerebellum Anterior Lobe

3 Nodule

1 Vermis_10 (aal)

----------------------

Cluster 15

Number of voxels: 17

Peak MNI coordinate: -48 -18 -39

Peak MNI coordinate region: // undefined // undefined // undefined // undefined // undefined // undefined

Peak intensity: -4.8784

# voxels structure

17 --TOTAL # VOXELS--

----------------------

Cluster 16

Number of voxels: 41

Peak MNI coordinate: -25.5 9 -37.5

Peak MNI coordinate region: // Left Cerebrum // Temporal Lobe // Superior Temporal Gyrus // White Matter // undefined // Temporal_Pole_Mid_L (aal)

Peak intensity: -4.708

# voxels structure

41 --TOTAL # VOXELS--

41 Left Cerebrum

41 Temporal_Pole_Mid_L (aal)

40 Temporal Lobe

34 Superior Temporal Gyrus

27 White Matter

14 brodmann area 38

14 Gray Matter

6 Sub-Gyral

1 Uncus

1 Limbic Lobe

----------------------

Cluster 17

Number of voxels: 18

Peak MNI coordinate: 33 16.5 -39

Peak MNI coordinate region: // Right Cerebrum // Temporal Lobe // Superior Temporal Gyrus // White Matter // undefined // Temporal_Pole_Mid_R (aal)

Peak intensity: -4.154

# voxels structure

18 --TOTAL # VOXELS--

18 Right Cerebrum

17 Superior Temporal Gyrus

17 Temporal Lobe

17 Temporal_Pole_Mid_R (aal)

14 White Matter

4 brodmann area 38

4 Gray Matter

1 Limbic Lobe

1 Temporal_Pole_Sup_R (aal)

1 Uncus

----------------------

Cluster 18

Number of voxels: 10

Peak MNI coordinate: 7.5 -64.5 -37.5

Peak MNI coordinate region: // Right Cerebellum // Cerebellum Posterior Lobe // Uvula // undefined // undefined // Cerebelum_8_R (aal)

Peak intensity: -3.5613

# voxels structure

10 --TOTAL # VOXELS--

10 Right Cerebellum

7 Vermis_8 (aal)

6 Cerebellum Posterior Lobe

6 Uvula

4 Cerebellum Anterior Lobe

4 Nodule

3 Cerebelum_8_R (aal)

----------------------

Cluster 19

Number of voxels: 29

Peak MNI coordinate: 4.5 -40.5 -27

Peak MNI coordinate region: // Right Cerebellum // Cerebellum Anterior Lobe // Culmen // undefined // undefined // undefined

Peak intensity: -4.2234

# voxels structure

29 --TOTAL # VOXELS--

26 Right Cerebellum

25 Cerebellum Anterior Lobe

11 Culmen

5 Vermis_10 (aal)

3 Right Brainstem

3 Pons

1 Sub-lobar

1 Fourth Ventricle

1 Cerebro-Spinal Fluid

----------------------

Cluster 20

Number of voxels: 12

Peak MNI coordinate: 54 -21 -34.5

Peak MNI coordinate region: // undefined // undefined // undefined // undefined // undefined // undefined

Peak intensity: -4.5879

# voxels structure

12 --TOTAL # VOXELS--

2 Inferior Temporal Gyrus

2 Right Cerebrum

2 Temporal Lobe

2 brodmann area 20

2 Gray Matter

1 Temporal_Inf_R (aal)

----------------------

Cluster 21

Number of voxels: 19

Peak MNI coordinate: -3 -78 -28.5

Peak MNI coordinate region: // Left Cerebellum // Cerebellum Posterior Lobe // Declive // undefined // undefined // Cerebelum_Crus2_L (aal)

Peak intensity: -4.9638

# voxels structure

19 --TOTAL # VOXELS--

19 Left Cerebellum

18 Cerebellum Posterior Lobe

13 Cerebelum_Crus2_L (aal)

11 Declive

6 Vermis_7 (aal)

6 Pyramis

1 Declive of Vermis

----------------------

Cluster 22

Number of voxels: 10

Peak MNI coordinate: -52.5 -22.5 -33

Peak MNI coordinate region: // undefined // undefined // undefined // undefined // undefined // undefined

Peak intensity: -4.6436

# voxels structure

10 --TOTAL # VOXELS--

4 Left Cerebrum

4 Temporal Lobe

4 Fusiform Gyrus

3 Temporal_Inf_L (aal)

3 brodmann area 20

3 Gray Matter

----------------------

Cluster 23

Number of voxels: 26

Peak MNI coordinate: 19.5 1.5 -31.5

Peak MNI coordinate region: // Right Cerebrum // Limbic Lobe // Uncus // Gray Matter // brodmann area 28 // ParaHippocampal_R (aal)

Peak intensity: -4.6871

# voxels structure

26 --TOTAL # VOXELS--

26 Limbic Lobe

26 Right Cerebrum

26 Uncus

20 Gray Matter

14 brodmann area 28

14 ParaHippocampal_R (aal)

4 White Matter

3 brodmann area 36

3 brodmann area 34

----------------------

Cluster 24

Number of voxels: 11

Peak MNI coordinate: 39 -28.5 -30

Peak MNI coordinate region: // undefined // undefined // undefined // undefined // undefined // Fusiform_R (aal)

Peak intensity: -3.8156

# voxels structure

11 --TOTAL # VOXELS--

10 Fusiform_R (aal)

3 Limbic Lobe

3 Parahippocampa Gyrus

3 Right Cerebrum

3 brodmann area 36

3 Gray Matter

1 Cerebelum_6_R (aal)

----------------------

Cluster 25

Number of voxels: 23

Peak MNI coordinate: 66 -9 -22.5

Peak MNI coordinate region: // Right Cerebrum // Temporal Lobe // Inferior Temporal Gyrus // Gray Matter // brodmann area 21 // Temporal_Mid_R (aal)

Peak intensity: -4.5393

# voxels structure

23 --TOTAL # VOXELS--

22 Temporal_Mid_R (aal)

2 Right Cerebrum

2 Temporal Lobe

2 Gray Matter

2 brodmann area 21

2 Inferior Temporal Gyrus

1 Temporal_Inf_R (aal)

----------------------

Cluster 26

Number of voxels: 10

Peak MNI coordinate: -19.5 3 -30

Peak MNI coordinate region: // Left Cerebrum // Limbic Lobe // Uncus // Gray Matter // brodmann area 28 // ParaHippocampal_L (aal)

Peak intensity: -4.9741

# voxels structure

10 --TOTAL # VOXELS--

10 Left Cerebrum

10 Limbic Lobe

10 Uncus

8 ParaHippocampal_L (aal)

7 Gray Matter

6 brodmann area 28

3 White Matter

2 Temporal_Pole_Sup_L (aal)

1 brodmann area 38

----------------------

Cluster 27

Number of voxels: 23

Peak MNI coordinate: -13.5 -7.5 -25.5

Peak MNI coordinate region: // Left Cerebrum // Limbic Lobe // Parahippocampa Gyrus // undefined // undefined // ParaHippocampal_L (aal)

Peak intensity: -4.5291

# voxels structure

23 --TOTAL # VOXELS--

21 Left Cerebrum

21 Limbic Lobe

17 Gray Matter

17 ParaHippocampal_L (aal)

15 Uncus

7 Amygdala

6 brodmann area 34

6 Parahippocampa Gyrus

5 Amygdala_L (aal)

4 brodmann area 28

1 White Matter

1 Hippocampus_L (aal)

----------------------

Cluster 28

Number of voxels: 27

Peak MNI coordinate: -6 13.5 -24

Peak MNI coordinate region: // Left Cerebrum // Frontal Lobe // Rectal Gyrus // Gray Matter // brodmann area 11 // Rectus_L (aal)

Peak intensity: -4.9133

# voxels structure

27 --TOTAL # VOXELS--

15 Left Cerebrum

15 Frontal Lobe

11 Rectal Gyrus

10 Gray Matter

9 brodmann area 11

8 Frontal_Sup_Orb_L (aal)

8 Rectus_L (aal)

4 Orbital Gyrus

1 brodmann area 47

1 White Matter

----------------------

Cluster 29

Number of voxels: 16

Peak MNI coordinate: 12 18 -25.5

Peak MNI coordinate region: // undefined // undefined // undefined // undefined // undefined // Frontal_Sup_Orb_R (aal)

Peak intensity: -4.4716

# voxels structure

16 --TOTAL # VOXELS--

10 Frontal_Sup_Orb_R (aal)

9 Right Cerebrum

9 Frontal Lobe

7 Gray Matter

5 Orbital Gyrus

4 brodmann area 47

3 brodmann area 11

3 Rectus_R (aal)

3 Rectal Gyrus

----------------------

Cluster 30

Number of voxels: 10

Peak MNI coordinate: -27 -69 -24

Peak MNI coordinate region: // Left Cerebellum // Cerebellum Posterior Lobe // Declive // undefined // undefined // Cerebelum_6_L (aal)

Peak intensity: -3.6901

# voxels structure

10 --TOTAL # VOXELS--

10 Cerebelum_6_L (aal)

10 Declive

10 Left Cerebellum

10 Cerebellum Posterior Lobe

----------------------

Cluster 31

Number of voxels: 11

Peak MNI coordinate: 64.5 -25.5 -24

Peak MNI coordinate region: // undefined // undefined // undefined // undefined // undefined // Temporal_Inf_R (aal)

Peak intensity: -4.686

# voxels structure

11 --TOTAL # VOXELS--

11 Temporal_Inf_R (aal)

3 Temporal Lobe

3 Inferior Temporal Gyrus

3 Right Cerebrum

2 brodmann area 20

2 Gray Matter

1 White Matter

----------------------

Cluster 32

Number of voxels: 28

Peak MNI coordinate: -4.5 25.5 -22.5

Peak MNI coordinate region: // Left Cerebrum // Frontal Lobe // Rectal Gyrus // Gray Matter // brodmann area 11 // Rectus_L (aal)

Peak intensity: -4.7831

# voxels structure

28 --TOTAL # VOXELS--

28 Rectus_L (aal)

27 Left Cerebrum

27 Rectal Gyrus

27 Frontal Lobe

15 White Matter

12 brodmann area 11

12 Gray Matter

1 Inter-Hemispheric

----------------------

Cluster 33

Number of voxels: 10

Peak MNI coordinate: -1.5 -70.5 -22.5

Peak MNI coordinate region: // Left Cerebellum // Cerebellum Posterior Lobe // Declive of Vermis // undefined // undefined // Vermis_6 (aal)

Peak intensity: -4.7196

# voxels structure

10 --TOTAL # VOXELS--

10 Cerebellum Posterior Lobe

7 Vermis_6 (aal)

7 Declive of Vermis

5 Left Cerebellum

5 Right Cerebellum

3 Declive

3 Cerebelum_6_L (aal)

----------------------

Cluster 34

Number of voxels: 14

Peak MNI coordinate: 22.5 34.5 -22.5

Peak MNI coordinate region: // Right Cerebrum // Frontal Lobe // Inferior Frontal Gyrus // White Matter // undefined // Frontal_Sup_Orb_R (aal)

Peak intensity: -4.0311

# voxels structure

14 --TOTAL # VOXELS--

14 Frontal Lobe

14 Right Cerebrum

11 Inferior Frontal Gyrus

8 Frontal_Mid_Orb_R (aal)

6 White Matter

6 Frontal_Sup_Orb_R (aal)

4 brodmann area 11

4 Gray Matter

3 Superior Frontal Gyrus

----------------------

Cluster 35

Number of voxels: 11

Peak MNI coordinate: 61.5 -45 -22.5

Peak MNI coordinate region: // undefined // undefined // undefined // undefined // undefined // Temporal_Inf_R (aal)

Peak intensity: -4.0832

# voxels structure

11 --TOTAL # VOXELS--

11 Temporal_Inf_R (aal)

2 Temporal Lobe

2 Inferior Temporal Gyrus

2 Right Cerebrum

----------------------

Cluster 36

Number of voxels: 63

Peak MNI coordinate: -39 22.5 -15

Peak MNI coordinate region: // Left Cerebrum // Frontal Lobe // Inferior Frontal Gyrus // White Matter // undefined // Frontal_Inf_Orb_L (aal)

Peak intensity: -5.4332

# voxels structure

63 --TOTAL # VOXELS--

60 Left Cerebrum

60 Frontal Lobe

56 Frontal_Inf_Orb_L (aal)

54 Inferior Frontal Gyrus

47 White Matter

12 brodmann area 47

12 Gray Matter

6 Sub-Gyral

6 Insula_L (aal)

1 Frontal_Sup_Orb_L (aal)

----------------------

Cluster 37

Number of voxels: 13

Peak MNI coordinate: 6 31.5 -22.5

Peak MNI coordinate region: // Right Cerebrum // Frontal Lobe // Rectal Gyrus // White Matter // undefined // Rectus_R (aal)

Peak intensity: -3.9251

# voxels structure

13 --TOTAL # VOXELS--

13 Frontal Lobe

13 Rectus_R (aal)

13 Right Cerebrum

12 White Matter

11 Rectal Gyrus

2 Medial Frontal Gyrus

1 brodmann area 11

1 Gray Matter

----------------------

Cluster 38

Number of voxels: 10

Peak MNI coordinate: -22.5 37.5 -21

Peak MNI coordinate region: // Left Cerebrum // Frontal Lobe // Inferior Frontal Gyrus // undefined // undefined // Frontal_Mid_Orb_L (aal)

Peak intensity: -3.7808

# voxels structure

10 --TOTAL # VOXELS--

10 Left Cerebrum

10 Frontal Lobe

8 Inferior Frontal Gyrus

6 Frontal_Mid_Orb_L (aal)

4 brodmann area 11

4 Gray Matter

2 Frontal_Sup_Orb_L (aal)

2 Superior Frontal Gyrus

1 White Matter

----------------------

Cluster 39

Number of voxels: 10

Peak MNI coordinate: -4.5 40.5 -21

Peak MNI coordinate region: // Left Cerebrum // Frontal Lobe // Orbital Gyrus // Gray Matter // brodmann area 11 // Rectus_L (aal)

Peak intensity: -3.7974

# voxels structure

10 --TOTAL # VOXELS--

10 Frontal Lobe

10 Left Cerebrum

10 Rectus_L (aal)

7 Orbital Gyrus

7 White Matter

3 Gray Matter

3 brodmann area 11

3 Sub-Gyral

----------------------

Cluster 40

Number of voxels: 101

Peak MNI coordinate: 12 25.5 -13.5

Peak MNI coordinate region: // Right Cerebrum // Frontal Lobe // Medial Frontal Gyrus // White Matter // undefined // Rectus_R (aal)

Peak intensity: -6.1177

# voxels structure

101 --TOTAL # VOXELS--

101 Frontal Lobe

101 Right Cerebrum

87 Rectus_R (aal)

63 White Matter

38 Gray Matter

35 Medial Frontal Gyrus

30 brodmann area 47

24 Sub-Gyral

24 Inferior Frontal Gyrus

18 Subcallosal Gyrus

12 Frontal_Sup_Orb_R (aal)

7 brodmann area 25

2 Olfactory_R (aal)

1 brodmann area 11

----------------------

Cluster 41

Number of voxels: 20

Peak MNI coordinate: 69 -31.5 -15

Peak MNI coordinate region: // undefined // undefined // undefined // undefined // undefined // Temporal_Mid_R (aal)

Peak intensity: -4.7844

# voxels structure

20 --TOTAL # VOXELS--

14 Temporal_Mid_R (aal)

6 Temporal_Inf_R (aal)

----------------------

Cluster 42

Number of voxels: 47

Peak MNI coordinate: -46.5 46.5 -16.5

Peak MNI coordinate region: // undefined // undefined // undefined // undefined // undefined // undefined

Peak intensity: -4.4235

# voxels structure

47 --TOTAL # VOXELS--

6 Left Cerebrum

6 Frontal_Inf_Orb_L (aal)

1 Middle Frontal Gyrus

1 Frontal Lobe

----------------------

Cluster 43

Number of voxels: 11

Peak MNI coordinate: -9 48 -18

Peak MNI coordinate region: // Left Cerebrum // Frontal Lobe // Medial Frontal Gyrus // White Matter // undefined // Rectus_L (aal)

Peak intensity: -4.0552

# voxels structure

11 --TOTAL # VOXELS--

11 Frontal Lobe

11 Left Cerebrum

11 White Matter

10 Medial Frontal Gyrus

10 Rectus_L (aal)

1 Frontal_Sup_Orb_L (aal)

1 Sub-Gyral

----------------------

Cluster 44

Number of voxels: 19

Peak MNI coordinate: 4.5 -18 -16.5

Peak MNI coordinate region: // Right Brainstem // Midbrain // undefined // undefined // undefined // undefined

Peak intensity: -4.2107

# voxels structure

19 --TOTAL # VOXELS--

19 Right Brainstem

19 Midbrain

4 Substania Nigra

4 Gray Matter

----------------------

Cluster 45

Number of voxels: 13

Peak MNI coordinate: 37.5 24 -16.5

Peak MNI coordinate region: // Right Cerebrum // Frontal Lobe // Inferior Frontal Gyrus // Gray Matter // brodmann area 47 // Frontal_Inf_Orb_R (aal)

Peak intensity: -3.7358

# voxels structure

13 --TOTAL # VOXELS--

13 Frontal Lobe

13 Inferior Frontal Gyrus

13 Right Cerebrum

11 White Matter

9 Frontal_Inf_Orb_R (aal)

4 Insula_R (aal)

2 brodmann area 47

2 Gray Matter

----------------------

Cluster 46

Number of voxels: 15

Peak MNI coordinate: -10.5 28.5 -16.5

Peak MNI coordinate region: // Left Cerebrum // Frontal Lobe // Medial Frontal Gyrus // White Matter // undefined // Rectus_L (aal)

Peak intensity: -3.9761

# voxels structure

15 --TOTAL # VOXELS--

15 Frontal Lobe

15 Left Cerebrum

15 Rectus_L (aal)

11 White Matter

9 Medial Frontal Gyrus

4 Gray Matter

3 Sub-Gyral

2 brodmann area 25

2 brodmann area 47

2 Inferior Frontal Gyrus

1 Subcallosal Gyrus

----------------------

Cluster 47

Number of voxels: 10

Peak MNI coordinate: 7.5 -1.5 -15

Peak MNI coordinate region: // undefined // undefined // undefined // undefined // undefined // undefined

Peak intensity: -4.9226

# voxels structure

10 --TOTAL # VOXELS--

1 White Matter

1 Optic Tract

----------------------

Cluster 48

Number of voxels: 13

Peak MNI coordinate: -1.5 -87 -12

Peak MNI coordinate region: // Left Cerebrum // Occipital Lobe // Lingual Gyrus // Gray Matter // brodmann area 18 // Calcarine_L (aal)

Peak intensity: -3.9055

# voxels structure

13 --TOTAL # VOXELS--

13 Left Cerebrum

13 Lingual Gyrus

13 Occipital Lobe

11 Calcarine_L (aal)

9 Gray Matter

9 brodmann area 18

4 White Matter

2 Lingual_L (aal)

----------------------

Cluster 49

Number of voxels: 15

Peak MNI coordinate: -46.5 -73.5 -13.5

Peak MNI coordinate region: // Left Cerebrum // Occipital Lobe // Middle Occipital Gyrus // White Matter // undefined // Occipital_Inf_L (aal)

Peak intensity: -3.8257

# voxels structure

15 --TOTAL # VOXELS--

15 Left Cerebrum

15 Middle Occipital Gyrus

15 Occipital Lobe

15 Occipital_Inf_L (aal)

15 White Matter

----------------------

Cluster 50

Number of voxels: 18

Peak MNI coordinate: 51 43.5 -13.5

Peak MNI coordinate region: // Right Cerebrum // Frontal Lobe // Inferior Frontal Gyrus // Gray Matter // brodmann area 47 // Frontal_Inf_Orb_R (aal)

Peak intensity: -3.4697

# voxels structure

18 --TOTAL # VOXELS--

17 Right Cerebrum

17 Frontal Lobe

16 Frontal_Inf_Orb_R (aal)

13 Inferior Frontal Gyrus

6 brodmann area 47

6 Gray Matter

4 Middle Frontal Gyrus

1 Frontal_Mid_Orb_R (aal)

----------------------

Cluster 51

Number of voxels: 41

Peak MNI coordinate: -7.5 -12 -7.5

Peak MNI coordinate region: // Left Brainstem // Midbrain // undefined // Gray Matter // Subthalamic Nucleus // undefined

Peak intensity: -4.8146

# voxels structure

41 --TOTAL # VOXELS--

40 Midbrain

40 Left Brainstem

16 Gray Matter

15 Subthalamic Nucleus

1 Sub-lobar

1 Substania Nigra

1 Left Cerebrum

1 White Matter

1 Extra-Nuclear

----------------------

Cluster 52

Number of voxels: 10

Peak MNI coordinate: 28.5 -16.5 -9

Peak MNI coordinate region: // Right Cerebrum // Sub-lobar // Lateral Ventricle // Cerebro-Spinal Fluid // undefined // undefined

Peak intensity: -3.9776

# voxels structure

10 --TOTAL # VOXELS--

10 Right Cerebrum

7 White Matter

7 Sub-lobar

4 Extra-Nuclear

3 Hippocampus_R (aal)

3 Lateral Ventricle

3 Cerebro-Spinal Fluid

2 Sub-Gyral

2 Temporal Lobe

1 Limbic Lobe

1 Parahippocampa Gyrus

----------------------

Cluster 53

Number of voxels: 13

Peak MNI coordinate: -43.5 37.5 -10.5

Peak MNI coordinate region: // Left Cerebrum // Frontal Lobe // Middle Frontal Gyrus // White Matter // undefined // Frontal_Inf_Orb_L (aal)

Peak intensity: -4.8165

# voxels structure

13 --TOTAL # VOXELS--

13 Frontal Lobe

13 Frontal_Inf_Orb_L (aal)

13 Left Cerebrum

7 Inferior Frontal Gyrus

7 White Matter

6 Gray Matter

6 brodmann area 47

6 Middle Frontal Gyrus

----------------------

Cluster 54

Number of voxels: 11

Peak MNI coordinate: 57 -67.5 -9

Peak MNI coordinate region: // Right Cerebrum // Occipital Lobe // Middle Occipital Gyrus // Gray Matter // brodmann area 19 // Temporal_Inf_R (aal)

Peak intensity: -3.6952

# voxels structure

11 --TOTAL # VOXELS--

11 Right Cerebrum

10 Temporal_Inf_R (aal)

9 Gray Matter

8 Occipital Lobe

6 Middle Occipital Gyrus

6 brodmann area 19

5 Inferior Temporal Gyrus

3 brodmann area 37

3 Temporal Lobe

----------------------

Cluster 55

Number of voxels: 25

Peak MNI coordinate: 9 -10.5 -6

Peak MNI coordinate region: // Right Cerebrum // Sub-lobar // Extra-Nuclear // White Matter // undefined // undefined

Peak intensity: -4.7117

# voxels structure

25 --TOTAL # VOXELS--

17 Right Brainstem

17 Midbrain

8 Gray Matter

8 Right Cerebrum

8 Sub-lobar

8 Subthalamic Nucleus

8 White Matter

8 Extra-Nuclear

----------------------

Cluster 56

Number of voxels: 11

Peak MNI coordinate: 45 -49.5 -7.5

Peak MNI coordinate region: // Right Cerebrum // Temporal Lobe // Sub-Gyral // White Matter // undefined // Temporal_Inf_R (aal)

Peak intensity: -3.9618

# voxels structure

11 --TOTAL # VOXELS--

11 Sub-Gyral

11 Temporal Lobe

11 White Matter

11 Right Cerebrum

5 Temporal_Inf_R (aal)

----------------------

Cluster 57

Number of voxels: 15

Peak MNI coordinate: -7.5 1.5 -7.5

Peak MNI coordinate region: // Left Cerebrum // Sub-lobar // Extra-Nuclear // White Matter // undefined // undefined

Peak intensity: -4.6408

# voxels structure

15 --TOTAL # VOXELS--

15 Sub-lobar

15 Left Cerebrum

12 White Matter

12 Extra-Nuclear

3 Gray Matter

1 Lentiform Nucleus

1 Medial Globus Pallidus

1 Pallidum_L (aal)

----------------------

Cluster 58

Number of voxels: 24

Peak MNI coordinate: 3 67.5 -6

Peak MNI coordinate region: // Inter-Hemispheric // undefined // undefined // undefined // undefined // Frontal_Med_Orb_R (aal)

Peak intensity: -4.1217

# voxels structure

24 --TOTAL # VOXELS--

11 Inter-Hemispheric

7 Frontal_Med_Orb_R (aal)

----------------------

Cluster 59

Number of voxels: 23

Peak MNI coordinate: 52.5 -67.5 -6

Peak MNI coordinate region: // Right Cerebrum // Temporal Lobe // Inferior Temporal Gyrus // White Matter // undefined // Temporal_Inf_R (aal)

Peak intensity: -4.1634

# voxels structure

23 --TOTAL # VOXELS--

23 Right Cerebrum

23 Temporal_Inf_R (aal)

15 Inferior Temporal Gyrus

15 Temporal Lobe

11 Gray Matter

10 White Matter

8 Occipital Lobe

6 Middle Temporal Gyrus

5 brodmann area 37

2 Middle Occipital Gyrus

1 brodmann area 19

----------------------

Cluster 60

Number of voxels: 10

Peak MNI coordinate: 24 -46.5 -6

Peak MNI coordinate region: // Right Cerebrum // Limbic Lobe // Parahippocampa Gyrus // White Matter // undefined // Lingual_R (aal)

Peak intensity: -3.839

# voxels structure

10 --TOTAL # VOXELS--

10 Lingual_R (aal)

10 Right Cerebrum

10 White Matter

6 Occipital Lobe

6 Sub-Gyral

4 Limbic Lobe

4 Parahippocampa Gyrus

----------------------

Cluster 61

Number of voxels: 17

Peak MNI coordinate: 58.5 1.5 -6

Peak MNI coordinate region: // Right Cerebrum // Temporal Lobe // Superior Temporal Gyrus // Gray Matter // brodmann area 22 // Temporal_Pole_Sup_R (aal)

Peak intensity: -4.7519

# voxels structure

17 --TOTAL # VOXELS--

17 Right Cerebrum

17 Temporal Lobe

15 Superior Temporal Gyrus

10 Gray Matter

10 Temporal_Pole_Sup_R (aal)

8 brodmann area 22

7 Temporal_Sup_R (aal)

6 White Matter

2 Middle Temporal Gyrus

2 brodmann area 21

----------------------

Cluster 62

Number of voxels: 156

Peak MNI coordinate: 27 0 3

Peak MNI coordinate region: // Right Cerebrum // Sub-lobar // Lentiform Nucleus // Gray Matter // Putamen // Putamen_R (aal)

Peak intensity: -4.9924

# voxels structure

156 --TOTAL # VOXELS--

156 Right Cerebrum

156 Sub-lobar

154 Putamen_R (aal)

96 Gray Matter

94 Putamen

94 Lentiform Nucleus

60 White Matter

60 Extra-Nuclear

2 Claustrum

1 Pallidum_R (aal)

----------------------

Cluster 63

Number of voxels: 14

Peak MNI coordinate: 6 -33 -1.5

Peak MNI coordinate region: // Right Brainstem // Midbrain // undefined // undefined // undefined // undefined

Peak intensity: -4.5327

# voxels structure

14 --TOTAL # VOXELS--

14 Right Brainstem

13 Midbrain

2 Vermis_3 (aal)

----------------------

Cluster 64

Number of voxels: 12

Peak MNI coordinate: -30 -13.5 -1.5

Peak MNI coordinate region: // Left Cerebrum // Sub-lobar // Lentiform Nucleus // Gray Matter // Putamen // Putamen_L (aal)

Peak intensity: -4.8031

# voxels structure

12 --TOTAL # VOXELS--

12 Left Cerebrum

12 Putamen_L (aal)

12 Sub-lobar

8 Putamen

8 Gray Matter

8 Lentiform Nucleus

4 Extra-Nuclear

4 White Matter

----------------------

Cluster 65

Number of voxels: 67

Peak MNI coordinate: -25.5 67.5 10.5

Peak MNI coordinate region: // Left Cerebrum // Frontal Lobe // Middle Frontal Gyrus // Gray Matter // brodmann area 10 // undefined

Peak intensity: -4.2691

# voxels structure

67 --TOTAL # VOXELS--

57 Left Cerebrum

57 Frontal Lobe

50 Gray Matter

49 Superior Frontal Gyrus

48 brodmann area 10

37 Frontal_Sup_L (aal)

7 Middle Frontal Gyrus

3 Frontal_Sup_Orb_L (aal)

2 Frontal_Sup_Medial_L (aal)

----------------------

Cluster 66

Number of voxels: 330

Peak MNI coordinate: 28.5 -54 10.5

Peak MNI coordinate region: // Right Cerebrum // Sub-lobar // Lateral Ventricle // Cerebro-Spinal Fluid // undefined // Calcarine_R (aal)

Peak intensity: -5.4324

# voxels structure

330 --TOTAL # VOXELS--

330 Right Cerebrum

283 Sub-lobar

153 White Matter

148 Cerebro-Spinal Fluid

148 Lateral Ventricle

111 Extra-Nuclear

59 Hippocampus_R (aal)

47 Sub-Gyral

47 Temporal Lobe

43 Precuneus_R (aal)

29 Gray Matter

26 Calcarine_R (aal)

13 Thalamus

11 Caudate Tail

11 Caudate

8 Pulvinar

5 Hippocampus

5 Corpus Callosum

----------------------

Cluster 67

Number of voxels: 11

Peak MNI coordinate: -40.5 58.5 0

Peak MNI coordinate region: // Left Cerebrum // Frontal Lobe // Middle Frontal Gyrus // Gray Matter // brodmann area 10 // Frontal_Mid_L (aal)

Peak intensity: -3.8809

# voxels structure

11 --TOTAL # VOXELS--

11 Frontal Lobe

11 Gray Matter

11 Left Cerebrum

10 Middle Frontal Gyrus

10 brodmann area 10

3 Frontal_Mid_Orb_L (aal)

2 Frontal_Mid_L (aal)

1 Superior Frontal Gyrus

----------------------

Cluster 68

Number of voxels: 62

Peak MNI coordinate: -4.5 -102 3

Peak MNI coordinate region: // Left Cerebrum // Occipital Lobe // Cuneus // Gray Matter // brodmann area 18 // Calcarine_L (aal)

Peak intensity: -5.4313

# voxels structure

62 --TOTAL # VOXELS--

62 Occipital Lobe

59 Left Cerebrum

55 Cuneus

50 Gray Matter

50 brodmann area 18

30 Calcarine_L (aal)

18 Occipital_Sup_L (aal)

10 Cuneus_L (aal)

7 Middle Occipital Gyrus

5 White Matter

3 Inter-Hemispheric

2 Occipital_Mid_L (aal)

----------------------

Cluster 69

Number of voxels: 33

Peak MNI coordinate: -6 -58.5 4.5

Peak MNI coordinate region: // Left Cerebrum // Limbic Lobe // Posterior Cingulate // White Matter // undefined // Calcarine_L (aal)

Peak intensity: -4.5731

# voxels structure

33 --TOTAL # VOXELS--

31 Left Cerebrum

21 Limbic Lobe

21 Posterior Cingulate

21 Calcarine_L (aal)

17 White Matter

12 Lingual_L (aal)

12 Gray Matter

10 Occipital Lobe

9 Lingual Gyrus

6 brodmann area 30

5 brodmann area 18

2 Inter-Hemispheric

1 brodmann area 19

----------------------

Cluster 70

Number of voxels: 41

Peak MNI coordinate: 16.5 -34.5 4.5

Peak MNI coordinate region: // Right Cerebrum // Sub-lobar // Thalamus // Gray Matter // Pulvinar // Thalamus_R (aal)

Peak intensity: -5.6593

# voxels structure

41 --TOTAL # VOXELS--

41 Right Cerebrum

38 Sub-lobar

26 Thalamus

26 Gray Matter

23 Pulvinar

19 Hippocampus_R (aal)

15 White Matter

12 Extra-Nuclear

12 Thalamus_R (aal)

3 Parahippocampa Gyrus

3 Limbic Lobe

----------------------

Cluster 71

Number of voxels: 26

Peak MNI coordinate: 49.5 -12 1.5

Peak MNI coordinate region: // Right Cerebrum // Temporal Lobe // Superior Temporal Gyrus // White Matter // undefined // Temporal_Sup_R (aal)

Peak intensity: -4.5319

# voxels structure

26 --TOTAL # VOXELS--

26 Right Cerebrum

23 Superior Temporal Gyrus

23 Temporal Lobe

23 Temporal_Sup_R (aal)

17 White Matter

6 brodmann area 22

6 Gray Matter

3 Insula

3 Heschl_R (aal)

3 Sub-lobar

----------------------

Cluster 72

Number of voxels: 291

Peak MNI coordinate: -22.5 -48 10.5

Peak MNI coordinate region: // Left Cerebrum // Sub-lobar // Lateral Ventricle // Cerebro-Spinal Fluid // undefined // Precuneus_L (aal)

Peak intensity: -5.3643

# voxels structure

291 --TOTAL # VOXELS--

291 Left Cerebrum

217 Sub-lobar

152 Lateral Ventricle

152 Cerebro-Spinal Fluid

104 White Matter

91 Calcarine_L (aal)

65 Extra-Nuclear

44 Posterior Cingulate

44 Limbic Lobe

35 Gray Matter

32 brodmann area 30

25 Precuneus_L (aal)

17 Cuneus

17 Occipital Lobe

13 Temporal Lobe

13 Sub-Gyral

2 brodmann area 17

1 Lingual_L (aal)

1 brodmann area 23

----------------------

Cluster 73

Number of voxels: 31

Peak MNI coordinate: -15 -34.5 4.5

Peak MNI coordinate region: // Left Cerebrum // Sub-lobar // Thalamus // Gray Matter // Pulvinar // Thalamus_L (aal)

Peak intensity: -5.1074

# voxels structure

31 --TOTAL # VOXELS--

31 Left Cerebrum

26 Sub-lobar

19 White Matter

14 Extra-Nuclear

12 Gray Matter

12 Thalamus

11 Pulvinar

11 Thalamus_L (aal)

9 Hippocampus_L (aal)

4 Limbic Lobe

4 Parahippocampa Gyrus

3 Corpus Callosum

1 Sub-Gyral

1 Temporal Lobe

----------------------

Cluster 74

Number of voxels: 21

Peak MNI coordinate: -16.5 15 6

Peak MNI coordinate region: // Left Cerebrum // Sub-lobar // Caudate // Gray Matter // Caudate Head // Caudate_L (aal)

Peak intensity: -4.8075

# voxels structure

21 --TOTAL # VOXELS--

21 Left Cerebrum

21 Sub-lobar

15 Caudate_L (aal)

11 Gray Matter

11 Caudate

10 White Matter

10 Extra-Nuclear

9 Caudate Head

2 Caudate Body

----------------------

Cluster 75

Number of voxels: 88

Peak MNI coordinate: 18 19.5 3

Peak MNI coordinate region: // Right Cerebrum // Sub-lobar // Extra-Nuclear // White Matter // undefined // Caudate_R (aal)

Peak intensity: -5.7754

# voxels structure

88 --TOTAL # VOXELS--

88 Right Cerebrum

88 Sub-lobar

87 Caudate_R (aal)

70 White Matter

70 Extra-Nuclear

18 Caudate

18 Gray Matter

9 Caudate Head

9 Caudate Body

----------------------

Cluster 76

Number of voxels: 17

Peak MNI coordinate: -3 30 0

Peak MNI coordinate region: // Left Cerebrum // Limbic Lobe // Anterior Cingulate // Gray Matter // brodmann area 24 // Cingulum_Ant_L (aal)

Peak intensity: -4.0418

# voxels structure

17 --TOTAL # VOXELS--

17 Left Cerebrum

11 White Matter

8 Sub-lobar

8 Extra-Nuclear

8 Corpus Callosum

6 Gray Matter

6 Anterior Cingulate

6 brodmann area 24

6 Limbic Lobe

3 Frontal Lobe

3 Sub-Gyral

2 Cingulum_Ant_L (aal)

----------------------

Cluster 77

Number of voxels: 10

Peak MNI coordinate: -27 -13.5 3

Peak MNI coordinate region: // Left Cerebrum // Sub-lobar // Lentiform Nucleus // Gray Matter // Putamen // Putamen_L (aal)

Peak intensity: -4.0831

# voxels structure

10 --TOTAL # VOXELS--

10 Left Cerebrum

10 Sub-lobar

9 Lentiform Nucleus

9 Putamen

9 Putamen_L (aal)

9 Gray Matter

1 White Matter

1 Extra-Nuclear

----------------------

Cluster 78

Number of voxels: 24

Peak MNI coordinate: -28.5 -7.5 6

Peak MNI coordinate region: // Left Cerebrum // Sub-lobar // Lentiform Nucleus // Gray Matter // Putamen // Putamen_L (aal)

Peak intensity: -4.2252

# voxels structure

24 --TOTAL # VOXELS--

24 Left Cerebrum

24 Putamen_L (aal)

24 Sub-lobar

23 Putamen

23 Gray Matter

23 Lentiform Nucleus

1 Extra-Nuclear

1 White Matter

----------------------

Cluster 79

Number of voxels: 10

Peak MNI coordinate: -36 -3 6

Peak MNI coordinate region: // Left Cerebrum // Sub-lobar // Extra-Nuclear // White Matter // undefined // Insula_L (aal)

Peak intensity: -4.7739

# voxels structure

10 --TOTAL # VOXELS--

10 Sub-lobar

10 White Matter

10 Left Cerebrum

8 Insula_L (aal)

5 Insula

5 Extra-Nuclear

----------------------

Cluster 80

Number of voxels: 19

Peak MNI coordinate: -22.5 7.5 6

Peak MNI coordinate region: // Left Cerebrum // Sub-lobar // Lentiform Nucleus // Gray Matter // Putamen // Putamen_L (aal)

Peak intensity: -4.4734

# voxels structure

19 --TOTAL # VOXELS--

19 Gray Matter

19 Left Cerebrum

19 Lentiform Nucleus

19 Putamen

19 Putamen_L (aal)

19 Sub-lobar

----------------------

Cluster 81

Number of voxels: 15

Peak MNI coordinate: 21 69 4.5

Peak MNI coordinate region: // Right Cerebrum // Frontal Lobe // Superior Frontal Gyrus // Gray Matter // brodmann area 10 // Frontal_Sup_R (aal)

Peak intensity: -3.5417

# voxels structure

15 --TOTAL # VOXELS--

15 Frontal Lobe

15 Frontal_Sup_R (aal)

15 Gray Matter

15 Right Cerebrum

15 Superior Frontal Gyrus

15 brodmann area 10

----------------------

Cluster 82

Number of voxels: 11

Peak MNI coordinate: 13.5 -91.5 6

Peak MNI coordinate region: // Right Cerebrum // Occipital Lobe // Cuneus // White Matter // undefined // Calcarine_R (aal)

Peak intensity: -4.0264

# voxels structure

11 --TOTAL # VOXELS--

11 Cuneus

11 Occipital Lobe

11 Right Cerebrum

11 White Matter

8 Calcarine_R (aal)

3 Cuneus_R (aal)

----------------------

Cluster 83

Number of voxels: 12

Peak MNI coordinate: 4.5 -63 6

Peak MNI coordinate region: // Right Cerebrum // Occipital Lobe // Cuneus // undefined // undefined // Lingual_R (aal)

Peak intensity: -4.2152

# voxels structure

12 --TOTAL # VOXELS--

12 Right Cerebrum

10 Lingual_R (aal)

7 Limbic Lobe

7 Posterior Cingulate

5 Occipital Lobe

5 Cuneus

3 brodmann area 30

3 Gray Matter

2 Calcarine_R (aal)

2 White Matter

----------------------

Cluster 84

Number of voxels: 69

Peak MNI coordinate: -22.5 -37.5 9

Peak MNI coordinate region: // Left Cerebrum // Sub-lobar // Lateral Ventricle // Cerebro-Spinal Fluid // undefined // undefined

Peak intensity: -4.467

# voxels structure

69 --TOTAL # VOXELS--

69 Left Cerebrum

68 Sub-lobar

36 White Matter

35 Extra-Nuclear

27 Cerebro-Spinal Fluid

27 Lateral Ventricle

23 Hippocampus_L (aal)

6 Gray Matter

4 Thalamus

3 Pulvinar

2 Caudate Tail

2 Caudate

1 Parahippocampa Gyrus

1 Corpus Callosum

1 Limbic Lobe

----------------------

Cluster 85

Number of voxels: 26

Peak MNI coordinate: 40.5 36 6

Peak MNI coordinate region: // Right Cerebrum // Frontal Lobe // Inferior Frontal Gyrus // White Matter // undefined // Frontal_Inf_Tri_R (aal)

Peak intensity: -4.8692

# voxels structure

26 --TOTAL # VOXELS--

26 Right Cerebrum

26 White Matter

26 Frontal Lobe

19 Inferior Frontal Gyrus

19 Frontal_Inf_Tri_R (aal)

7 Sub-Gyral

3 Frontal_Mid_R (aal)

----------------------

Cluster 86

Number of voxels: 16

Peak MNI coordinate: -45 -34.5 7.5

Peak MNI coordinate region: // Left Cerebrum // Temporal Lobe // Superior Temporal Gyrus // White Matter // undefined // Temporal_Sup_L (aal)

Peak intensity: -3.6934

# voxels structure

16 --TOTAL # VOXELS--

16 Superior Temporal Gyrus

16 Temporal Lobe

16 White Matter

16 Left Cerebrum

15 Temporal_Sup_L (aal)

----------------------

Cluster 87

Number of voxels: 16

Peak MNI coordinate: -51 -28.5 7.5

Peak MNI coordinate region: // Left Cerebrum // Temporal Lobe // Superior Temporal Gyrus // White Matter // undefined // Temporal_Sup_L (aal)

Peak intensity: -3.8771

# voxels structure

16 --TOTAL # VOXELS--

16 Left Cerebrum

16 Superior Temporal Gyrus

16 Temporal Lobe

16 White Matter

13 Temporal_Sup_L (aal)

3 Temporal_Mid_L (aal)

----------------------

Cluster 88

Number of voxels: 19

Peak MNI coordinate: -15 -12 7.5

Peak MNI coordinate region: // Left Cerebrum // Sub-lobar // Thalamus // Gray Matter // Ventral Lateral Nucleus // Thalamus_L (aal)

Peak intensity: -5.2769

# voxels structure

19 --TOTAL # VOXELS--

19 Gray Matter

19 Left Cerebrum

19 Sub-lobar

19 Thalamus

19 Thalamus_L (aal)

12 Ventral Lateral Nucleus

2 Ventral Posterior Lateral Nucleus

----------------------

Cluster 89

Number of voxels: 37

Peak MNI coordinate: -36 36 7.5

Peak MNI coordinate region: // Left Cerebrum // Frontal Lobe // Sub-Gyral // White Matter // undefined // Frontal_Inf_Tri_L (aal)

Peak intensity: -4.7515

# voxels structure

37 --TOTAL # VOXELS--

37 Frontal Lobe

37 Frontal_Inf_Tri_L (aal)

37 Left Cerebrum

37 White Matter

25 Inferior Frontal Gyrus

12 Sub-Gyral

----------------------

Cluster 90

Number of voxels: 12

Peak MNI coordinate: 16.5 -58.5 9

Peak MNI coordinate region: // Right Cerebrum // Limbic Lobe // Posterior Cingulate // Gray Matter // brodmann area 30 // Calcarine_R (aal)

Peak intensity: -4.0395

# voxels structure

12 --TOTAL # VOXELS--

12 Calcarine_R (aal)

12 Limbic Lobe

12 Posterior Cingulate

12 Right Cerebrum

10 Gray Matter

10 brodmann area 30

2 White Matter

----------------------

Cluster 91

Number of voxels: 14

Peak MNI coordinate: -4.5 -31.5 9

Peak MNI coordinate region: // Left Cerebrum // Sub-lobar // Extra-Nuclear // White Matter // Corpus Callosum // undefined

Peak intensity: -3.675

# voxels structure

14 --TOTAL # VOXELS--

14 White Matter

13 Extra-Nuclear

13 Sub-lobar

11 Corpus Callosum

8 Left Cerebrum

5 Right Cerebrum

1 Inter-Hemispheric

----------------------

Cluster 92

Number of voxels: 13

Peak MNI coordinate: -4.5 43.5 9

Peak MNI coordinate region: // Left Cerebrum // Limbic Lobe // Anterior Cingulate // White Matter // undefined // Cingulum_Ant_L (aal)

Peak intensity: -4.322

# voxels structure

13 --TOTAL # VOXELS--

13 Anterior Cingulate

13 Cingulum_Ant_L (aal)

13 Left Cerebrum

13 Limbic Lobe

7 White Matter

6 brodmann area 32

6 Gray Matter

----------------------

Cluster 93

Number of voxels: 13

Peak MNI coordinate: 13.5 55.5 7.5

Peak MNI coordinate region: // Right Cerebrum // Frontal Lobe // Medial Frontal Gyrus // White Matter // undefined // Frontal_Sup_Medial_R (aal)

Peak intensity: -3.8211

# voxels structure

13 --TOTAL # VOXELS--

13 Frontal Lobe

13 Medial Frontal Gyrus

13 Right Cerebrum

13 White Matter

10 Frontal_Sup_Medial_R (aal)

3 Frontal_Sup_R (aal)

----------------------

Cluster 94

Number of voxels: 11

Peak MNI coordinate: 12 -100.5 15

Peak MNI coordinate region: // Right Cerebrum // Occipital Lobe // Cuneus // Gray Matter // brodmann area 18 // Occipital_Sup_R (aal)

Peak intensity: -3.7854

# voxels structure

11 --TOTAL # VOXELS--

11 Occipital Lobe

11 Right Cerebrum

9 Gray Matter

9 brodmann area 18

7 Middle Occipital Gyrus

4 Cuneus

3 Occipital_Sup_R (aal)

3 Cuneus_R (aal)

2 White Matter

----------------------

Cluster 95

Number of voxels: 18

Peak MNI coordinate: 27 -78 10.5

Peak MNI coordinate region: // Right Cerebrum // Occipital Lobe // Middle Occipital Gyrus // White Matter // undefined // undefined

Peak intensity: -3.716

# voxels structure

18 --TOTAL # VOXELS--

18 White Matter

18 Right Cerebrum

13 Occipital Lobe

11 Middle Occipital Gyrus

6 Calcarine_R (aal)

4 Posterior Cingulate

4 Limbic Lobe

2 Cuneus

1 Extra-Nuclear

1 Sub-lobar

----------------------

Cluster 96

Number of voxels: 13

Peak MNI coordinate: -6 -52.5 12

Peak MNI coordinate region: // Left Cerebrum // Limbic Lobe // Posterior Cingulate // White Matter // undefined // Precuneus_L (aal)

Peak intensity: -4.0126

# voxels structure

13 --TOTAL # VOXELS--

13 Left Cerebrum

13 Limbic Lobe

13 Posterior Cingulate

13 Precuneus_L (aal)

7 White Matter

6 Gray Matter

4 brodmann area 30

2 brodmann area 29

----------------------

Cluster 97

Number of voxels: 12

Peak MNI coordinate: 19.5 -21 10.5

Peak MNI coordinate region: // Right Cerebrum // Sub-lobar // Thalamus // Gray Matter // Lateral Posterior Nucleus // Thalamus_R (aal)

Peak intensity: -4.8365

# voxels structure

12 --TOTAL # VOXELS--

12 Right Cerebrum

12 Sub-lobar

11 Gray Matter

11 Thalamus

11 Thalamus_R (aal)

6 Lateral Posterior Nucleus

1 Pulvinar

1 White Matter

1 Extra-Nuclear

----------------------

Cluster 98

Number of voxels: 10

Peak MNI coordinate: -66 -18 15

Peak MNI coordinate region: // Left Cerebrum // Parietal Lobe // Postcentral Gyrus // Gray Matter // brodmann area 43 // Postcentral_L (aal)

Peak intensity: -3.8727

# voxels structure

10 --TOTAL # VOXELS--

10 Left Cerebrum

8 Gray Matter

7 Parietal Lobe

7 Postcentral Gyrus

6 Postcentral_L (aal)

4 brodmann area 43

3 brodmann area 40

3 Temporal Lobe

3 Transverse Temporal Gyrus

1 brodmann area 42

1 SupraMarginal_L (aal)

1 Temporal_Sup_L (aal)

----------------------

Cluster 99

Number of voxels: 16

Peak MNI coordinate: 45 -6 13.5

Peak MNI coordinate region: // Right Cerebrum // Sub-lobar // Insula // White Matter // undefined // Rolandic_Oper_R (aal)

Peak intensity: -4.3719

# voxels structure

16 --TOTAL # VOXELS--

16 Right Cerebrum

16 Rolandic_Oper_R (aal)

16 Sub-lobar

15 Insula

9 White Matter

6 brodmann area 13

6 Gray Matter

1 Extra-Nuclear

----------------------

Cluster 100

Number of voxels: 13

Peak MNI coordinate: -40.5 55.5 10.5

Peak MNI coordinate region: // Left Cerebrum // Frontal Lobe // Middle Frontal Gyrus // White Matter // undefined // Frontal_Mid_L (aal)

Peak intensity: -3.6568

# voxels structure

13 --TOTAL # VOXELS--

13 Frontal Lobe

13 Frontal_Mid_L (aal)

13 Left Cerebrum

13 Middle Frontal Gyrus

11 White Matter

2 brodmann area 10

2 Gray Matter

----------------------

Cluster 101

Number of voxels: 11

Peak MNI coordinate: -57 -69 16.5

Peak MNI coordinate region: // Left Cerebrum // Temporal Lobe // Middle Temporal Gyrus // Gray Matter // brodmann area 39 // undefined

Peak intensity: -4.1012

# voxels structure

11 --TOTAL # VOXELS--

11 Gray Matter

11 Left Cerebrum

11 Middle Temporal Gyrus

8 brodmann area 39

8 Temporal Lobe

3 Occipital Lobe

3 brodmann area 19

----------------------

Cluster 102

Number of voxels: 19

Peak MNI coordinate: -16.5 9 13.5

Peak MNI coordinate region: // Left Cerebrum // Sub-lobar // Caudate // Gray Matter // Caudate Body // Caudate_L (aal)

Peak intensity: -4.2859

# voxels structure

19 --TOTAL # VOXELS--

19 Left Cerebrum

19 Sub-lobar

17 Caudate_L (aal)

10 White Matter

10 Extra-Nuclear

9 Caudate Body

9 Gray Matter

9 Caudate

----------------------

Cluster 103

Number of voxels: 14

Peak MNI coordinate: 28.5 66 15

Peak MNI coordinate region: // Right Cerebrum // Frontal Lobe // Superior Frontal Gyrus // undefined // undefined // Frontal_Sup_R (aal)

Peak intensity: -3.8209

# voxels structure

14 --TOTAL # VOXELS--

13 Frontal_Sup_R (aal)

13 Right Cerebrum

13 Frontal Lobe

9 Superior Frontal Gyrus

6 brodmann area 10

6 Gray Matter

4 Middle Frontal Gyrus

----------------------

Cluster 104

Number of voxels: 28

Peak MNI coordinate: -31.5 31.5 13.5

Peak MNI coordinate region: // Left Cerebrum // Frontal Lobe // Sub-Gyral // White Matter // undefined // Frontal_Mid_L (aal)

Peak intensity: -4.244

# voxels structure

28 --TOTAL # VOXELS--

28 Left Cerebrum

28 White Matter

28 Frontal Lobe

22 Frontal_Inf_Tri_L (aal)

14 Inferior Frontal Gyrus

14 Sub-Gyral

3 Frontal_Mid_L (aal)

----------------------

Cluster 105

Number of voxels: 20

Peak MNI coordinate: 43.5 27 16.5

Peak MNI coordinate region: // Right Cerebrum // Frontal Lobe // Inferior Frontal Gyrus // White Matter // undefined // Frontal_Inf_Tri_R (aal)

Peak intensity: -3.9053

# voxels structure

20 --TOTAL # VOXELS--

20 Right Cerebrum

20 White Matter

20 Frontal Lobe

11 Inferior Frontal Gyrus

11 Frontal_Inf_Tri_R (aal)

9 Sub-Gyral

----------------------

Cluster 106

Number of voxels: 14

Peak MNI coordinate: -43.5 43.5 16.5

Peak MNI coordinate region: // Left Cerebrum // Frontal Lobe // Middle Frontal Gyrus // Gray Matter // brodmann area 10 // Frontal_Mid_L (aal)

Peak intensity: -4.6878

# voxels structure

14 --TOTAL # VOXELS--

14 Frontal Lobe

14 Frontal_Mid_L (aal)

14 Left Cerebrum

13 Middle Frontal Gyrus

12 Gray Matter

8 brodmann area 10

4 brodmann area 46

2 White Matter

1 Inferior Frontal Gyrus

----------------------

Cluster 107

Number of voxels: 114

Peak MNI coordinate: 66 -1.5 31.5

Peak MNI coordinate region: // Right Cerebrum // Frontal Lobe // Precentral Gyrus // undefined // undefined // Postcentral_R (aal)

Peak intensity: -4.6982

# voxels structure

114 --TOTAL # VOXELS--

112 Precentral Gyrus

112 Right Cerebrum

111 Frontal Lobe

88 Postcentral_R (aal)

84 Gray Matter

78 brodmann area 6

9 Precentral_R (aal)

6 brodmann area 4

1 Parietal Lobe

----------------------

Cluster 108

Number of voxels: 113

Peak MNI coordinate: -21 -52.5 25.5

Peak MNI coordinate region: // Left Cerebrum // Frontal Lobe // Sub-Gyral // White Matter // undefined // undefined

Peak intensity: -4.9767

# voxels structure

113 --TOTAL # VOXELS--

113 White Matter

113 Left Cerebrum

102 Sub-Gyral

89 Parietal Lobe

13 Frontal Lobe

9 Cuneus_L (aal)

7 Extra-Nuclear

7 Sub-lobar

4 Precuneus_L (aal)

2 Precuneus

2 Temporal Lobe

2 Limbic Lobe

2 Cingulate Gyrus

1 Cingulum_Post_L (aal)

----------------------

Cluster 109

Number of voxels: 33

Peak MNI coordinate: 33 -46.5 21

Peak MNI coordinate region: // Right Cerebrum // Parietal Lobe // Sub-Gyral // White Matter // undefined // undefined

Peak intensity: -3.872

# voxels structure

33 --TOTAL # VOXELS--

33 White Matter

33 Right Cerebrum

26 Sub-Gyral

19 Parietal Lobe

7 Frontal Lobe

7 Sub-lobar

4 Extra-Nuclear

3 Insula

----------------------

Cluster 110

Number of voxels: 13

Peak MNI coordinate: -45 -1.5 19.5

Peak MNI coordinate region: // Left Cerebrum // Frontal Lobe // Sub-Gyral // White Matter // undefined // Precentral_L (aal)

Peak intensity: -4.2741

# voxels structure

13 --TOTAL # VOXELS--

13 Left Cerebrum

13 Sub-Gyral

13 White Matter

13 Frontal Lobe

8 Precentral_L (aal)

3 Rolandic_Oper_L (aal)

----------------------

Cluster 111

Number of voxels: 11

Peak MNI coordinate: -1.5 -67.5 22.5

Peak MNI coordinate region: // Left Cerebrum // Parietal Lobe // Precuneus // Gray Matter // brodmann area 31 // Calcarine_L (aal)

Peak intensity: -3.7032

# voxels structure

11 --TOTAL # VOXELS--

11 Left Cerebrum

11 Precuneus

10 Parietal Lobe

8 Gray Matter

7 brodmann area 31

6 Calcarine_L (aal)

5 Cuneus_L (aal)

3 White Matter

1 Occipital Lobe

1 brodmann area 23

----------------------

Cluster 112

Number of voxels: 13

Peak MNI coordinate: -61.5 3 22.5

Peak MNI coordinate region: // Left Cerebrum // Frontal Lobe // Precentral Gyrus // undefined // undefined // Precentral_L (aal)

Peak intensity: -4.2446

# voxels structure

13 --TOTAL # VOXELS--

13 Left Cerebrum

13 Frontal Lobe

10 Precentral_L (aal)

8 Precentral Gyrus

5 Inferior Frontal Gyrus

3 Postcentral_L (aal)

3 brodmann area 6

3 Gray Matter

2 White Matter

----------------------

Cluster 113

Number of voxels: 13

Peak MNI coordinate: 57 3 24

Peak MNI coordinate region: // Right Cerebrum // Frontal Lobe // Inferior Frontal Gyrus // Gray Matter // brodmann area 9 // Precentral_R (aal)

Peak intensity: -5.007

# voxels structure

13 --TOTAL # VOXELS--

13 Frontal Lobe

13 Inferior Frontal Gyrus

13 Precentral_R (aal)

13 Right Cerebrum

12 Gray Matter

8 brodmann area 9

4 brodmann area 44

1 White Matter

----------------------

Cluster 114

Number of voxels: 10

Peak MNI coordinate: -3 -79.5 27

Peak MNI coordinate region: // Left Cerebrum // Occipital Lobe // Cuneus // Gray Matter // brodmann area 19 // Cuneus_L (aal)

Peak intensity: -4.0819

# voxels structure

10 --TOTAL # VOXELS--

10 Cuneus

10 Cuneus_L (aal)

10 Left Cerebrum

10 Occipital Lobe

6 Gray Matter

3 brodmann area 19

3 brodmann area 18

----------------------

Cluster 115

Number of voxels: 13

Peak MNI coordinate: 34.5 54 25.5

Peak MNI coordinate region: // Right Cerebrum // Frontal Lobe // Middle Frontal Gyrus // White Matter // undefined // Frontal_Mid_R (aal)

Peak intensity: -4.1988

# voxels structure

13 --TOTAL # VOXELS--

13 Frontal Lobe

13 Frontal_Mid_R (aal)

13 Right Cerebrum

10 Middle Frontal Gyrus

7 Gray Matter

7 brodmann area 10

6 White Matter

3 Superior Frontal Gyrus

----------------------

Cluster 116

Number of voxels: 11

Peak MNI coordinate: 54 -36 27

Peak MNI coordinate region: // Right Cerebrum // Parietal Lobe // Inferior Parietal Lobule // White Matter // undefined // SupraMarginal_R (aal)

Peak intensity: -3.9468

# voxels structure

11 --TOTAL # VOXELS--

11 Inferior Parietal Lobule

11 Parietal Lobe

11 Right Cerebrum

11 SupraMarginal_R (aal)

10 White Matter

1 brodmann area 40

1 Gray Matter

----------------------

Cluster 117

Number of voxels: 14

Peak MNI coordinate: 0 -25.5 25.5

Peak MNI coordinate region: // Inter-Hemispheric // undefined // undefined // White Matter // Corpus Callosum // undefined

Peak intensity: -3.9195

# voxels structure

14 --TOTAL # VOXELS--

13 White Matter

12 Right Cerebrum

7 Cingulate Gyrus

7 Corpus Callosum

7 Limbic Lobe

5 Extra-Nuclear

5 Sub-lobar

2 Inter-Hemispheric

1 brodmann area 23

1 Gray Matter

----------------------

Cluster 118

Number of voxels: 18

Peak MNI coordinate: 13.5 22.5 30

Peak MNI coordinate region: // Right Cerebrum // Limbic Lobe // Cingulate Gyrus // White Matter // undefined // Cingulum_Mid_R (aal)

Peak intensity: -3.8614

# voxels structure

18 --TOTAL # VOXELS--

18 Right Cerebrum

18 White Matter

18 Limbic Lobe

11 Cingulate Gyrus

9 Cingulum_Mid_R (aal)

7 Anterior Cingulate

3 Cingulum_Ant_R (aal)

----------------------

Cluster 119

Number of voxels: 16

Peak MNI coordinate: 27 42 28.5

Peak MNI coordinate region: // Right Cerebrum // Frontal Lobe // Middle Frontal Gyrus // White Matter // undefined // Frontal_Mid_R (aal)

Peak intensity: -3.8557

# voxels structure

16 --TOTAL # VOXELS--

16 Frontal Lobe

16 Frontal_Mid_R (aal)

16 Right Cerebrum

14 White Matter

7 Sub-Gyral

5 Superior Frontal Gyrus

4 Middle Frontal Gyrus

2 brodmann area 10

2 Gray Matter

----------------------

Cluster 120

Number of voxels: 14

Peak MNI coordinate: -18 -85.5 34.5

Peak MNI coordinate region: // Left Cerebrum // Occipital Lobe // Cuneus // undefined // undefined // Occipital_Sup_L (aal)

Peak intensity: -3.8439

# voxels structure

14 --TOTAL # VOXELS--

14 Left Cerebrum

13 Cuneus

13 Occipital Lobe

12 Occipital_Sup_L (aal)

9 Gray Matter

7 brodmann area 7

2 Cuneus_L (aal)

2 brodmann area 19

1 Precuneus

1 Parietal Lobe

----------------------

Cluster 121

Number of voxels: 64

Peak MNI coordinate: 3 -10.5 28.5

Peak MNI coordinate region: // Right Cerebrum // Limbic Lobe // Cingulate Gyrus // Gray Matter // brodmann area 24 // undefined

Peak intensity: -4.6415

# voxels structure

64 --TOTAL # VOXELS--

63 Limbic Lobe

63 Cingulate Gyrus

49 Right Cerebrum

31 Gray Matter

27 White Matter

23 brodmann area 24

18 Cingulum_Mid_R (aal)

14 Left Cerebrum

8 brodmann area 23

2 Cingulum_Ant_R (aal)

1 Inter-Hemispheric

----------------------

Cluster 122

Number of voxels: 22

Peak MNI coordinate: -31.5 -67.5 33

Peak MNI coordinate region: // Left Cerebrum // Parietal Lobe // Precuneus // White Matter // undefined // Occipital_Mid_L (aal)

Peak intensity: -4.14

# voxels structure

22 --TOTAL # VOXELS--

22 Left Cerebrum

22 Occipital_Mid_L (aal)

22 Parietal Lobe

20 Precuneus

20 White Matter

2 Gray Matter

2 Sub-Gyral

1 brodmann area 39

1 brodmann area 19

----------------------

Cluster 123

Number of voxels: 16

Peak MNI coordinate: 42 6 33

Peak MNI coordinate region: // Right Cerebrum // Frontal Lobe // Inferior Frontal Gyrus // White Matter // undefined // Precentral_R (aal)

Peak intensity: -4.111

# voxels structure

16 --TOTAL # VOXELS--

16 Frontal Lobe

16 Right Cerebrum

16 White Matter

15 Inferior Frontal Gyrus

10 Precentral_R (aal)

6 Frontal_Inf_Oper_R (aal)

1 Middle Frontal Gyrus

----------------------

Cluster 124

Number of voxels: 10

Peak MNI coordinate: 6 -27 34.5

Peak MNI coordinate region: // Right Cerebrum // Limbic Lobe // Cingulate Gyrus // White Matter // undefined // Cingulum_Mid_R (aal)

Peak intensity: -3.9505

# voxels structure

10 --TOTAL # VOXELS--

10 Cingulate Gyrus

10 Cingulum_Mid_R (aal)

10 Limbic Lobe

10 Right Cerebrum

6 White Matter

4 Gray Matter

3 brodmann area 23

1 brodmann area 31

----------------------

Cluster 125

Number of voxels: 20

Peak MNI coordinate: 25.5 22.5 37.5

Peak MNI coordinate region: // Right Cerebrum // Frontal Lobe // Sub-Gyral // White Matter // undefined // Frontal_Mid_R (aal)

Peak intensity: -3.6557

# voxels structure

20 --TOTAL # VOXELS--

20 Frontal Lobe

20 Right Cerebrum

20 White Matter

19 Sub-Gyral

17 Frontal_Mid_R (aal)

3 Frontal_Sup_R (aal)

1 Middle Frontal Gyrus

----------------------

Cluster 126

Number of voxels: 21

Peak MNI coordinate: -49.5 -61.5 40.5

Peak MNI coordinate region: // Left Cerebrum // Parietal Lobe // Inferior Parietal Lobule // Gray Matter // brodmann area 40 // Angular_L (aal)

Peak intensity: -4.9045

# voxels structure

21 --TOTAL # VOXELS--

21 Inferior Parietal Lobule

21 Left Cerebrum

21 Parietal Lobe

13 Gray Matter

12 Angular_L (aal)

10 brodmann area 40

8 Parietal_Inf_L (aal)

8 White Matter

3 brodmann area 39

----------------------

Cluster 127

Number of voxels: 10

Peak MNI coordinate: -6 -21 40.5

Peak MNI coordinate region: // Left Cerebrum // Limbic Lobe // Cingulate Gyrus // undefined // undefined // Cingulum_Mid_L (aal)

Peak intensity: -4.7564

# voxels structure

10 --TOTAL # VOXELS--

10 Cingulum_Mid_L (aal)

10 Left Cerebrum

10 Limbic Lobe

10 Cingulate Gyrus

1 brodmann area 24

1 Gray Matter

----------------------

Cluster 128

Number of voxels: 16

Peak MNI coordinate: -61.5 -10.5 43.5

Peak MNI coordinate region: // Left Cerebrum // Frontal Lobe // Precentral Gyrus // Gray Matter // brodmann area 6 // undefined

Peak intensity: -3.6893

# voxels structure

16 --TOTAL # VOXELS--

15 Precentral Gyrus

14 Frontal Lobe

14 Left Cerebrum

7 Gray Matter

7 brodmann area 6

7 Postcentral_L (aal)

5 White Matter

----------------------

Cluster 129

Number of voxels: 60

Peak MNI coordinate: -24 12 42

Peak MNI coordinate region: // Left Cerebrum // Frontal Lobe // Sub-Gyral // White Matter // undefined // Frontal_Mid_L (aal)

Peak intensity: -4.3879

# voxels structure

60 --TOTAL # VOXELS--

60 Left Cerebrum

60 Frontal Lobe

57 White Matter

54 Sub-Gyral

23 Frontal_Mid_L (aal)

16 Frontal_Sup_L (aal)

6 Middle Frontal Gyrus

3 brodmann area 8

3 Gray Matter

----------------------

Cluster 130

Number of voxels: 19

Peak MNI coordinate: -55.5 -6 51

Peak MNI coordinate region: // undefined // undefined // undefined // undefined // undefined // undefined

Peak intensity: -4.208

# voxels structure

19 --TOTAL # VOXELS--

17 Left Cerebrum

17 Frontal Lobe

12 Precentral Gyrus

12 brodmann area 6

12 Gray Matter

7 Precentral_L (aal)

5 Middle Frontal Gyrus

1 White Matter

----------------------

Cluster 131

Number of voxels: 77

Peak MNI coordinate: 54 3 51

Peak MNI coordinate region: // undefined // undefined // undefined // undefined // undefined // undefined

Peak intensity: -4.9424

# voxels structure

77 --TOTAL # VOXELS--

64 Right Cerebrum

64 Frontal Lobe

41 Middle Frontal Gyrus

38 Precentral_R (aal)

28 Frontal_Mid_R (aal)

28 Gray Matter

27 brodmann area 6

22 Precentral Gyrus

2 White Matter

1 brodmann area 8

1 Inferior Frontal Gyrus

----------------------

Cluster 132

Number of voxels: 87

Peak MNI coordinate: -18 -75 55.5

Peak MNI coordinate region: // undefined // undefined // undefined // undefined // undefined // Parietal_Sup_L (aal)

Peak intensity: -4.957

# voxels structure

87 --TOTAL # VOXELS--

42 Parietal Lobe

42 Left Cerebrum

41 Parietal_Sup_L (aal)

38 Precuneus_L (aal)

38 Precuneus

23 White Matter

16 brodmann area 7

16 Gray Matter

4 Superior Parietal Lobule

----------------------

Cluster 133

Number of voxels: 11

Peak MNI coordinate: 25.5 -72 48

Peak MNI coordinate region: // Right Cerebrum // Parietal Lobe // Precuneus // undefined // undefined // Parietal_Sup_R (aal)

Peak intensity: -4.2208

# voxels structure

11 --TOTAL # VOXELS--

11 Parietal Lobe

11 Right Cerebrum

10 Parietal_Sup_R (aal)

9 Precuneus

8 Gray Matter

8 brodmann area 7

2 Superior Parietal Lobule

1 Occipital_Sup_R (aal)

----------------------

Cluster 134

Number of voxels: 46

Peak MNI coordinate: -54 -19.5 58.5

Peak MNI coordinate region: // undefined // undefined // undefined // undefined // undefined // Postcentral_L (aal)

Peak intensity: -4.5667

# voxels structure

46 --TOTAL # VOXELS--

8 Parietal Lobe

8 Left Cerebrum

7 Postcentral Gyrus

6 Postcentral_L (aal)

1 brodmann area 3

1 Gray Matter

----------------------

Cluster 135

Number of voxels: 21

Peak MNI coordinate: -21 -63 49.5

Peak MNI coordinate region: // Left Cerebrum // Parietal Lobe // Precuneus // Gray Matter // brodmann area 7 // Parietal_Sup_L (aal)

Peak intensity: -3.7991

# voxels structure

21 --TOTAL # VOXELS--

21 Left Cerebrum

21 Parietal Lobe

21 Parietal_Sup_L (aal)

17 Precuneus

12 Gray Matter

12 brodmann area 7

9 White Matter

4 Superior Parietal Lobule

----------------------

Cluster 136

Number of voxels: 39

Peak MNI coordinate: 45 -42 51

Peak MNI coordinate region: // Right Cerebrum // Parietal Lobe // Inferior Parietal Lobule // White Matter // undefined // Parietal_Inf_R (aal)

Peak intensity: -5.1884

# voxels structure

39 --TOTAL # VOXELS--

39 Inferior Parietal Lobule

39 Parietal Lobe

39 Right Cerebrum

28 Parietal_Inf_R (aal)

21 Gray Matter

21 brodmann area 40

18 White Matter

11 Parietal_Sup_R (aal)

----------------------

Cluster 137

Number of voxels: 12

Peak MNI coordinate: 12 -33 49.5

Peak MNI coordinate region: // Right Cerebrum // Frontal Lobe // Paracentral Lobule // White Matter // undefined // Paracentral_Lobule_R (aal)

Peak intensity: -3.5799

# voxels structure

12 --TOTAL # VOXELS--

12 White Matter

12 Right Cerebrum

11 Frontal Lobe

10 Paracentral_Lobule_R (aal)

6 Paracentral Lobule

6 Sub-Gyral

1 Parietal Lobe

----------------------

Cluster 138

Number of voxels: 68

Peak MNI coordinate: 18 -21 60

Peak MNI coordinate region: // Right Cerebrum // Frontal Lobe // Sub-Gyral // White Matter // undefined // undefined

Peak intensity: -5.1769

# voxels structure

68 --TOTAL # VOXELS--

68 Right Cerebrum

68 Frontal Lobe

67 White Matter

59 Sub-Gyral

27 Precentral_R (aal)

6 Middle Frontal Gyrus

2 Precentral Gyrus

1 Medial Frontal Gyrus

1 brodmann area 6

1 Gray Matter

----------------------

Cluster 139

Number of voxels: 16

Peak MNI coordinate: -12 6 52.5

Peak MNI coordinate region: // Left Cerebrum // Limbic Lobe // Cingulate Gyrus // Gray Matter // brodmann area 24 // Frontal_Sup_L (aal)

Peak intensity: -3.6994

# voxels structure

16 --TOTAL # VOXELS--

16 Left Cerebrum

14 Frontal Lobe

13 Medial Frontal Gyrus

9 Gray Matter

8 Supp_Motor_Area_L (aal)

7 White Matter

6 Frontal_Sup_L (aal)

5 brodmann area 6

2 brodmann area 24

2 brodmann area 32

2 Limbic Lobe

2 Cingulate Gyrus

1 Superior Frontal Gyrus

----------------------

Cluster 140

Number of voxels: 68

Peak MNI coordinate: -28.5 21 61.5

Peak MNI coordinate region: // Left Cerebrum // Frontal Lobe // Middle Frontal Gyrus // undefined // undefined // Frontal_Mid_L (aal)

Peak intensity: -4.1295

# voxels structure

68 --TOTAL # VOXELS--

47 Frontal_Mid_L (aal)

36 Left Cerebrum

36 Frontal Lobe

28 Superior Frontal Gyrus

15 Gray Matter

14 brodmann area 8

8 Middle Frontal Gyrus

3 White Matter

1 Frontal_Sup_L (aal)

1 brodmann area 6

----------------------

Cluster 141

Number of voxels: 13

Peak MNI coordinate: -22.5 -36 55.5

Peak MNI coordinate region: // Left Cerebrum // Parietal Lobe // Postcentral Gyrus // White Matter // undefined // Postcentral_L (aal)

Peak intensity: -3.9291

# voxels structure

13 --TOTAL # VOXELS--

13 Left Cerebrum

13 Parietal Lobe

12 Postcentral Gyrus

11 Postcentral_L (aal)

7 brodmann area 3

7 Gray Matter

6 White Matter

1 Sub-Gyral

----------------------

Cluster 142

Number of voxels: 29

Peak MNI coordinate: 54 -15 58.5

Peak MNI coordinate region: // undefined // undefined // undefined // undefined // undefined // Postcentral_R (aal)

Peak intensity: -4.5334

# voxels structure

29 --TOTAL # VOXELS--

6 Right Cerebrum

6 Parietal Lobe

5 Postcentral Gyrus

3 Postcentral_R (aal)

3 Precentral_R (aal)

1 Precentral Gyrus

1 brodmann area 3

1 Gray Matter

----------------------

Cluster 143

Number of voxels: 35

Peak MNI coordinate: 24 -31.5 58.5

Peak MNI coordinate region: // Right Cerebrum // Parietal Lobe // Postcentral Gyrus // Gray Matter // brodmann area 3 // Postcentral_R (aal)

Peak intensity: -4.5123

# voxels structure

35 --TOTAL # VOXELS--

35 Right Cerebrum

32 Postcentral Gyrus

32 Parietal Lobe

24 Gray Matter

23 Postcentral_R (aal)

21 brodmann area 3

7 Precentral_R (aal)

5 White Matter

3 Precentral Gyrus

3 brodmann area 4

3 Frontal Lobe

----------------------

Cluster 144

Number of voxels: 54

Peak MNI coordinate: 34.5 -28.5 57

Peak MNI coordinate region: // Right Cerebrum // Frontal Lobe // Precentral Gyrus // White Matter // undefined // Precentral_R (aal)

Peak intensity: -4.7252

# voxels structure

54 --TOTAL # VOXELS--

47 Right Cerebrum

36 Frontal Lobe

36 Precentral Gyrus

28 Postcentral_R (aal)

25 Gray Matter

24 Precentral_R (aal)

18 brodmann area 4

15 White Matter

11 Postcentral Gyrus

11 Parietal Lobe

7 brodmann area 3

----------------------

Cluster 145

Number of voxels: 17

Peak MNI coordinate: -37.5 -64.5 57

Peak MNI coordinate region: // undefined // undefined // undefined // undefined // undefined // Parietal_Sup_L (aal)

Peak intensity: -3.8886

# voxels structure

17 --TOTAL # VOXELS--

17 Parietal_Sup_L (aal)

----------------------

Cluster 146

Number of voxels: 28

Peak MNI coordinate: -12 -33 61.5

Peak MNI coordinate region: // Left Cerebrum // Frontal Lobe // Sub-Gyral // White Matter // undefined // Paracentral_Lobule_L (aal)

Peak intensity: -4.5671

# voxels structure

28 --TOTAL # VOXELS--

28 Left Cerebrum

28 White Matter

28 Frontal Lobe

23 Sub-Gyral

19 Paracentral_Lobule_L (aal)

5 Paracentral Lobule

----------------------

Cluster 147

Number of voxels: 14

Peak MNI coordinate: 15 -72 61.5

Peak MNI coordinate region: // undefined // undefined // undefined // undefined // undefined // Parietal_Sup_R (aal)

Peak intensity: -4.6968

# voxels structure

14 --TOTAL # VOXELS--

8 Parietal_Sup_R (aal)

6 Precuneus_R (aal)

2 Right Cerebrum

2 Superior Parietal Lobule

2 Parietal Lobe

1 brodmann area 7

1 Gray Matter

----------------------

Cluster 148

Number of voxels: 134

Peak MNI coordinate: 7.5 -34.5 63

Peak MNI coordinate region: // Right Cerebrum // Frontal Lobe // Paracentral Lobule // White Matter // undefined // Paracentral_Lobule_R (aal)

Peak intensity: -4.8706

# voxels structure

134 --TOTAL # VOXELS--

134 Right Cerebrum

114 Paracentral_Lobule_R (aal)

97 Paracentral Lobule

93 White Matter

74 Parietal Lobe

60 Frontal Lobe

40 Gray Matter

36 Postcentral Gyrus

20 brodmann area 4

20 Postcentral_R (aal)

11 brodmann area 5

7 brodmann area 6

1 Sub-Gyral

1 brodmann area 7

1 brodmann area 3

----------------------

Cluster 149

Number of voxels: 16

Peak MNI coordinate: -33 -15 61.5

Peak MNI coordinate region: // Left Cerebrum // Frontal Lobe // Precentral Gyrus // White Matter // undefined // Precentral_L (aal)

Peak intensity: -4.8133

# voxels structure

16 --TOTAL # VOXELS--

16 Frontal Lobe

16 Left Cerebrum

16 Precentral Gyrus

16 Precentral_L (aal)

15 White Matter

1 brodmann area 6

1 Gray Matter

----------------------

Cluster 150

Number of voxels: 13

Peak MNI coordinate: -6 18 63

Peak MNI coordinate region: // Left Cerebrum // Frontal Lobe // Superior Frontal Gyrus // Gray Matter // brodmann area 6 // Supp_Motor_Area_L (aal)

Peak intensity: -3.9119

# voxels structure

13 --TOTAL # VOXELS--

13 Frontal Lobe

13 Left Cerebrum

13 Superior Frontal Gyrus

13 Supp_Motor_Area_L (aal)

8 White Matter

3 brodmann area 6

3 Gray Matter

----------------------

Cluster 151

Number of voxels: 10

Peak MNI coordinate: -15 9 63

Peak MNI coordinate region: // Left Cerebrum // Frontal Lobe // Middle Frontal Gyrus // Gray Matter // brodmann area 6 // Frontal_Sup_L (aal)

Peak intensity: -4.0904

# voxels structure

10 --TOTAL # VOXELS--

10 Frontal Lobe

10 Left Cerebrum

9 Frontal_Sup_L (aal)

8 Middle Frontal Gyrus

6 White Matter

4 brodmann area 6

4 Gray Matter

2 Superior Frontal Gyrus

1 Supp_Motor_Area_L (aal)

----------------------

Cluster 152

Number of voxels: 14

Peak MNI coordinate: -15 -69 64.5

Peak MNI coordinate region: // undefined // undefined // undefined // undefined // undefined // undefined

Peak intensity: -4.0912

# voxels structure

14 --TOTAL # VOXELS--

7 Parietal_Sup_L (aal)

1 Left Cerebrum

1 Parietal Lobe

----------------------

Cluster 153

Number of voxels: 162

Peak MNI coordinate: -15 -55.5 75

Peak MNI coordinate region: // undefined // undefined // undefined // undefined // undefined // Parietal_Sup_L (aal)

Peak intensity: -4.8377

# voxels structure

162 --TOTAL # VOXELS--

116 Parietal_Sup_L (aal)

23 Precuneus_L (aal)

18 Postcentral_L (aal)

1 Left Cerebrum

1 Parietal Lobe

1 Superior Parietal Lobule

1 brodmann area 7

1 Gray Matter

----------------------

Cluster 154

Number of voxels: 12

Peak MNI coordinate: 39 -13.5 67.5

Peak MNI coordinate region: // undefined // undefined // undefined // undefined // undefined // Precentral_R (aal)

Peak intensity: -4.0299

# voxels structure

12 --TOTAL # VOXELS--

12 Precentral_R (aal)

4 Frontal Lobe

4 Right Cerebrum

4 White Matter

4 Precentral Gyrus

----------------------

Cluster 155

Number of voxels: 10

Peak MNI coordinate: -7.5 -3 67.5

Peak MNI coordinate region: // Left Cerebrum // Frontal Lobe // Superior Frontal Gyrus // Gray Matter // brodmann area 6 // Supp_Motor_Area_L (aal)

Peak intensity: -3.6807

# voxels structure

10 --TOTAL # VOXELS--

10 Frontal Lobe

10 Left Cerebrum

10 Supp_Motor_Area_L (aal)

6 Superior Frontal Gyrus

5 Gray Matter

5 White Matter

5 brodmann area 6

4 Medial Frontal Gyrus

----------------------

Cluster 156

Number of voxels: 17

Peak MNI coordinate: -24 -1.5 66

Peak MNI coordinate region: // Left Cerebrum // Frontal Lobe // Middle Frontal Gyrus // Gray Matter // brodmann area 6 // Frontal_Sup_L (aal)

Peak intensity: -4.4929

# voxels structure

17 --TOTAL # VOXELS--

17 Frontal_Sup_L (aal)

16 Left Cerebrum

16 Frontal Lobe

9 Superior Frontal Gyrus

7 Middle Frontal Gyrus

4 White Matter

4 brodmann area 6

4 Gray Matter

----------------------

Cluster 157

Number of voxels: 15

Peak MNI coordinate: -4.5 19.5 66

Peak MNI coordinate region: // Left Cerebrum // Frontal Lobe // Superior Frontal Gyrus // Gray Matter // brodmann area 6 // Supp_Motor_Area_L (aal)

Peak intensity: -4.0361

# voxels structure

15 --TOTAL # VOXELS--

11 Supp_Motor_Area_L (aal)

6 Left Cerebrum

6 Superior Frontal Gyrus

6 Frontal Lobe

6 brodmann area 6

6 Gray Matter

----------------------

Cluster 158

Number of voxels: 18

Peak MNI coordinate: -28.5 -30 70.5

Peak MNI coordinate region: // Left Cerebrum // Frontal Lobe // Precentral Gyrus // Gray Matter // brodmann area 4 // Postcentral_L (aal)

Peak intensity: -4.3503

# voxels structure

18 --TOTAL # VOXELS--

18 Left Cerebrum

18 Postcentral_L (aal)

11 Postcentral Gyrus

11 Gray Matter

11 Parietal Lobe

7 Precentral Gyrus

7 brodmann area 4

7 Frontal Lobe

4 brodmann area 3

----------------------

Cluster 159

Number of voxels: 21

Peak MNI coordinate: -16.5 19.5 67.5

Peak MNI coordinate region: // undefined // undefined // undefined // undefined // undefined // undefined

Peak intensity: -4.1451

# voxels structure

21 --TOTAL # VOXELS--

9 Frontal_Sup_L (aal)

6 Gray Matter

6 Left Cerebrum

6 Superior Frontal Gyrus

6 brodmann area 6

6 Frontal Lobe

----------------------

Cluster 160

Number of voxels: 40

Peak MNI coordinate: 12 -64.5 69

Peak MNI coordinate region: // undefined // undefined // undefined // undefined // undefined // Precuneus_R (aal)

Peak intensity: -4.6171

# voxels structure

40 --TOTAL # VOXELS--

36 Parietal_Sup_R (aal)

4 Precuneus_R (aal)

----------------------

Cluster 161

Number of voxels: 10

Peak MNI coordinate: 3 -55.5 69

Peak MNI coordinate region: // Inter-Hemispheric // undefined // undefined // undefined // undefined // Precuneus_R (aal)

Peak intensity: -3.9382

# voxels structure

10 --TOTAL # VOXELS--

10 Precuneus_R (aal)

8 Inter-Hemispheric

----------------------

Cluster 162

Number of voxels: 18

Peak MNI coordinate: -3 -55.5 69

Peak MNI coordinate region: // Left Cerebrum // Parietal Lobe // Postcentral Gyrus // undefined // undefined // Precuneus_L (aal)

Peak intensity: -4.4675

# voxels structure

18 --TOTAL # VOXELS--

16 Precuneus_L (aal)

12 Postcentral Gyrus

12 Left Cerebrum

12 Parietal Lobe

4 Inter-Hemispheric

3 brodmann area 7

3 Gray Matter

----------------------

Cluster 163

Number of voxels: 40

Peak MNI coordinate: 19.5 -21 72

Peak MNI coordinate region: // Right Cerebrum // Frontal Lobe // Precentral Gyrus // Gray Matter // brodmann area 6 // Precentral_R (aal)

Peak intensity: -4.6023

# voxels structure

40 --TOTAL # VOXELS--

40 Frontal Lobe

40 Precentral Gyrus

40 Precentral_R (aal)

40 Right Cerebrum

25 Gray Matter

25 brodmann area 6

14 White Matter

----------------------

Cluster 164

Number of voxels: 21

Peak MNI coordinate: 22.5 -31.5 72

Peak MNI coordinate region: // Right Cerebrum // Parietal Lobe // Postcentral Gyrus // Gray Matter // brodmann area 3 // Postcentral_R (aal)

Peak intensity: -4.3939

# voxels structure

21 --TOTAL # VOXELS--

21 Parietal Lobe

21 Postcentral Gyrus

21 Right Cerebrum

14 Postcentral_R (aal)

12 Gray Matter

11 brodmann area 3

7 Precentral_R (aal)

7 White Matter

1 brodmann area 1

----------------------

Cluster 165

Number of voxels: 48

Peak MNI coordinate: 22.5 -54 75

Peak MNI coordinate region: // undefined // undefined // undefined // undefined // undefined // Parietal_Sup_R (aal)

Peak intensity: -4.4514

# voxels structure

48 --TOTAL # VOXELS--

40 Parietal_Sup_R (aal)

8 Postcentral_R (aal)

----------------------

Cluster 166

Number of voxels: 36

Peak MNI coordinate: -6 -25.5 76.5

Peak MNI coordinate region: // Left Cerebrum // Frontal Lobe // Precentral Gyrus // White Matter // undefined // Paracentral_Lobule_L (aal)

Peak intensity: -4.2222

# voxels structure

36 --TOTAL # VOXELS--

36 Frontal Lobe

36 Left Cerebrum

36 Paracentral_Lobule_L (aal)

33 Precentral Gyrus

29 White Matter

7 Gray Matter

4 brodmann area 4

3 brodmann area 6

2 Medial Frontal Gyrus

1 Paracentral Lobule

----------------------

Cluster 167

Number of voxels: 25

Peak MNI coordinate: -7.5 -42 73.5

Peak MNI coordinate region: // Left Cerebrum // Parietal Lobe // Postcentral Gyrus // White Matter // undefined // Precuneus_L (aal)

Peak intensity: -4.2649

# voxels structure

25 --TOTAL # VOXELS--

25 Left Cerebrum

25 Parietal Lobe

25 Postcentral Gyrus

20 Precuneus_L (aal)

15 White Matter

9 Gray Matter

7 brodmann area 5

5 Paracentral_Lobule_L (aal)

2 brodmann area 7

----------------------

Cluster 168

Number of voxels: 16

Peak MNI coordinate: -13.5 -12 79.5

Peak MNI coordinate region: // Left Cerebrum // Frontal Lobe // Superior Frontal Gyrus // undefined // undefined // Precentral_L (aal)

Peak intensity: -4.3412

# voxels structure

16 --TOTAL # VOXELS--

14 Left Cerebrum

14 Frontal Lobe

12 Precentral_L (aal)

9 Superior Frontal Gyrus

5 Precentral Gyrus

4 brodmann area 6

4 Gray Matter

3 Paracentral_Lobule_L (aal)

----------------------

Cluster 169

Number of voxels: 15

Peak MNI coordinate: 12 -1.5 75

Peak MNI coordinate region: // Right Cerebrum // Frontal Lobe // Superior Frontal Gyrus // White Matter // undefined // Supp_Motor_Area_R (aal)

Peak intensity: -4.0526

# voxels structure

15 --TOTAL # VOXELS--

15 Frontal Lobe

15 Right Cerebrum

15 Superior Frontal Gyrus

14 White Matter

10 Supp_Motor_Area_R (aal)

5 Frontal_Sup_R (aal)

1 brodmann area 6

1 Gray Matter

----------------------

Cluster 170

Number of voxels: 13

Peak MNI coordinate: -4.5 -9 79.5

Peak MNI coordinate region: // Left Cerebrum // Frontal Lobe // Superior Frontal Gyrus // White Matter // undefined // Paracentral_Lobule_L (aal)

Peak intensity: -4.0315

# voxels structure

13 --TOTAL # VOXELS--

13 Frontal Lobe

13 Left Cerebrum

13 Superior Frontal Gyrus

12 Supp_Motor_Area_L (aal)

10 White Matter

3 brodmann area 6

3 Gray Matter

1 Paracentral_Lobule_L (aal)

----------------------

Cluster 171

Number of voxels: 11

Peak MNI coordinate: -16.5 -48 76.5

Peak MNI coordinate region: // undefined // undefined // undefined // undefined // undefined // Parietal_Sup_L (aal)

Peak intensity: -3.9766

# voxels structure

11 --TOTAL # VOXELS--

8 Parietal_Sup_L (aal)

3 Precuneus_L (aal)

----------------------

Cluster 172

Number of voxels: 14

Peak MNI coordinate: -9 -37.5 75

Peak MNI coordinate region: // Left Cerebrum // Parietal Lobe // Postcentral Gyrus // White Matter // undefined // Paracentral_Lobule_L (aal)

Peak intensity: -3.8567

# voxels structure

14 --TOTAL # VOXELS--

14 Left Cerebrum

14 Paracentral_Lobule_L (aal)

14 Parietal Lobe

14 Postcentral Gyrus

13 White Matter

1 brodmann area 3

1 Gray Matter

----------------------

Cluster 173

Number of voxels: 10

Peak MNI coordinate: -19.5 -21 79.5

Peak MNI coordinate region: // undefined // undefined // undefined // undefined // undefined // Precentral_L (aal)

Peak intensity: -3.7807

# voxels structure

10 --TOTAL # VOXELS--

6 Precentral_L (aal)

2 Paracentral_Lobule_L (aal)

2 Postcentral_L (aal)

----------------------

Cluster 174

Number of voxels: 15

Peak MNI coordinate: 16.5 -37.5 82.5

Peak MNI coordinate region: // Right Cerebrum // Parietal Lobe // Postcentral Gyrus // Gray Matter // brodmann area 7 // Postcentral_R (aal)

Peak intensity: -4.2683

# voxels structure

15 --TOTAL # VOXELS--

13 Postcentral_R (aal)

12 Right Cerebrum

12 Parietal Lobe

11 White Matter

11 Postcentral Gyrus

1 Gray Matter

1 brodmann area 7

1 Paracentral Lobule

----------------------

Cluster 175

Number of voxels: 10

Peak MNI coordinate: -12 -37.5 82.5

Peak MNI coordinate region: // undefined // undefined // undefined // undefined // undefined // Postcentral_L (aal)

Peak intensity: -4.5377

# voxels structure

10 --TOTAL # VOXELS--

5 Postcentral_L (aal)

5 Paracentral_Lobule_L (aal)

>>
