# Supplementary material for: Age of acquisition impacts the brain differently depending on neuroanatomical metric
Source: Hum Brain Mapp. 2019 Oct 10;41(2):484–502. doi: 10.1002/hbm.24817 (PMC7267963; doi:10.1002/hbm.24817)
Supplement: Supplementary file 3 — Appendix C Regions where gray matter volume was significantly related to age of acquisition in Freesurfer [file HBM-41-484-s004.docx]

Appendix C

*Regions where gray matter volume was significantly related to age of acquisition in Freesurfer*

Contents:

C.1 ANOVA: Monolingual vs. bilingual contrast………………………………………… Page 1

C.2 ANCOVA: Early vs. late bilingual contrast, controlling English proficiency…………Page 2

**C.1 ANOVA: Monolingual vs. bilingual contrast**

*Contrast: Monolingual vs. bilingual. “Max” indicates *t* value, where positive/negative sign indicates direction of relationship (positive: Monolingual > Bilingual; negative: Monolingual < Bilingual). VtxMax – Vertix number at the maximum. Coordinates are in MNI305 space. NVtxs – number of voxels in cluster. Annot – annotation of the brain region where significant cluster fell according to Desikan-Killiany atlas, a gyral-based atlas built into Freesurfer package. Note that formatting of Appendices A and B1 differ from that of B2 and C due to differences in SPM and Freesurfer outputs.

Right hemisphere

# Max VtxMax Size(mm^2) X Y Z NVtxs Annot

1 -6.904 17007 154.87 22.0 -54.8 20.4 309 precuneus

2 4.532 103037 74.91 45.3 -38.8 0.4 220 bankssts

3 -4.397 59739 48.12 35.6 -43.7 57.6 118 superiorparietal

4 4.394 19808 64.68 37.8 32.4 26.9 104 rostralmiddlefrontal

5 -4.159 3848 25.89 27.6 17.7 -17.8 70 lateralorbitofrontal

6 4.138 36060 24.88 44.7 -28.1 -5.0 77 middletemporal

7 -3.997 141951 29.98 4.2 -75.3 27.9 53 cuneus

8 3.910 107186 27.37 46.2 -14.7 19.1 78 postcentral

9 3.515 42026 0.93 44.0 17.2 6.7 2 parsopercularis

**C.2 ANCOVA: Early vs. late bilingual contrast, controlling for proficiency**

*Contrast: Early bilingual vs. Late bilingual controlling for English proficiency. “Max” indicates *t* value, where positive/negative sign indicates direction of relationship (positive: Early > Late; negative: Early < Late).

Left Hemisphere

# ClusterNo Max VtxMax Size(mm^2) TalX TalY TalZ NVtxs Annot

1 6.265 161348 139.16 -56.9 -4.5 -22.0 196 middletemporal
