# Supplementary material for: Age of acquisition impacts the brain differently depending on neuroanatomical metric
Source: Hum Brain Mapp. 2019 Oct 10;41(2):484–502. doi: 10.1002/hbm.24817 (PMC7267963; doi:10.1002/hbm.24817)
Supplement: Supplementary file 4 — Appendix D Regions where gray matter volume was significantly related to age of acquisition in SPM [file HBM-41-484-s005.docx]

Appendix D

*Regions where gray matter volume was significantly related to age of acquisition in SPM*

Contents:

D.1 ANOVA: Monolingual vs. bilingual contrast………………………………………… Page 1

D.2 ANCOVA: Early vs. late bilingual contrast, controlling English proficiency……… Page 29

**D.1 ANOVA: Monolingual vs. bilingual contrast**

*Contrast: Monolingual vs. bilingual. “Peak intensity” indicates *t* value at cluster peak, where positive/negative sign indicates direction of relationship (positive: Monolingual > Bilingual; negative: Monolingual < Bilingual). Results rendered using xjview toolbox. Note that formatting of Appendices A and B1 differ from that of B2 and C due to differences in SPM and Freesurfer outputs.

***Monolingual < Bilingual (negative direction relationship)***

Type: T

df: 324

Threshold

-- p value = 0.0010615

-- intensity = 3.0974

-- cluster size = 10

Number of clusters found: 41

----------------------

Cluster 1

Number of voxels: 23

Peak MNI coordinate: -18 -60 -54

Peak MNI coordinate region: // Left Cerebellum // Cerebellum Posterior Lobe // Inferior Semi-Lunar Lobule // undefined // undefined // Cerebelum_8_L (aal)

Peak intensity: -3.9829

# voxels structure

23 --TOTAL # VOXELS--

23 Cerebelum_8_L (aal)

21 Left Cerebellum

21 Cerebellum Posterior Lobe

14 Inferior Semi-Lunar Lobule

7 Cerebellar Tonsil

----------------------

Cluster 2

Number of voxels: 21

Peak MNI coordinate: -15 -70.5 -49.5

Peak MNI coordinate region: // Left Cerebellum // Cerebellum Posterior Lobe // Inferior Semi-Lunar Lobule // undefined // undefined // Cerebelum_8_L (aal)

Peak intensity: -4.3921

# voxels structure

21 --TOTAL # VOXELS--

21 Cerebelum_8_L (aal)

21 Inferior Semi-Lunar Lobule

21 Left Cerebellum

21 Cerebellum Posterior Lobe

----------------------

Cluster 3

Number of voxels: 30

Peak MNI coordinate: -34.5 4.5 -46.5

Peak MNI coordinate region: // Left Cerebrum // Temporal Lobe // Middle Temporal Gyrus // Gray Matter // brodmann area 38 // undefined

Peak intensity: -3.6788

# voxels structure

30 --TOTAL # VOXELS--

17 Temporal Lobe

17 Left Cerebrum

9 Gray Matter

8 Inferior Temporal Gyrus

8 Middle Temporal Gyrus

6 brodmann area 38

4 White Matter

3 brodmann area 20

1 Superior Temporal Gyrus

----------------------

Cluster 4

Number of voxels: 2224

Peak MNI coordinate: 42 22.5 -36

Peak MNI coordinate region: // undefined // undefined // undefined // undefined // undefined // Temporal_Pole_Mid_R (aal)

Peak intensity: -6.2922

# voxels structure

2224 --TOTAL # VOXELS--

1493 Right Cerebrum

1486 Temporal Lobe

857 Gray Matter

755 Temporal_Inf_R (aal)

571 Middle Temporal Gyrus

543 Temporal_Pole_Mid_R (aal)

521 Temporal_Mid_R (aal)

416 Superior Temporal Gyrus

357 Inferior Temporal Gyrus

336 brodmann area 38

314 White Matter

292 brodmann area 21

266 Temporal_Pole_Sup_R (aal)

229 brodmann area 20

126 Fusiform Gyrus

32 Fusiform_R (aal)

9 Temporal_Sup_R (aal)

5 Sub-Gyral

3 Limbic Lobe

3 Frontal Lobe

2 Uncus

----------------------

Cluster 5

Number of voxels: 30

Peak MNI coordinate: 30 7.5 -48

Peak MNI coordinate region: // Right Cerebrum // Temporal Lobe // Superior Temporal Gyrus // White Matter // undefined // undefined

Peak intensity: -5.2084

# voxels structure

30 --TOTAL # VOXELS--

28 Right Cerebrum

27 Temporal Lobe

27 Superior Temporal Gyrus

18 brodmann area 38

18 Gray Matter

10 Temporal_Inf_R (aal)

10 White Matter

8 Fusiform_R (aal)

1 Uncus

1 Limbic Lobe

----------------------

Cluster 6

Number of voxels: 15

Peak MNI coordinate: 43.5 -9 -43.5

Peak MNI coordinate region: // undefined // undefined // undefined // undefined // undefined // Temporal_Inf_R (aal)

Peak intensity: -4.1693

# voxels structure

15 --TOTAL # VOXELS--

15 Temporal_Inf_R (aal)

----------------------

Cluster 7

Number of voxels: 16

Peak MNI coordinate: -30 13.5 -45

Peak MNI coordinate region: // undefined // undefined // undefined // undefined // undefined // undefined

Peak intensity: -4.31

# voxels structure

16 --TOTAL # VOXELS--

10 Superior Temporal Gyrus

10 Temporal Lobe

10 Left Cerebrum

9 brodmann area 38

9 Gray Matter

4 Temporal_Pole_Mid_L (aal)

1 Fusiform_L (aal)

1 White Matter

----------------------

Cluster 8

Number of voxels: 699

Peak MNI coordinate: -45 21 -25.5

Peak MNI coordinate region: // Left Cerebrum // Temporal Lobe // Superior Temporal Gyrus // Gray Matter // brodmann area 38 // Temporal_Pole_Sup_L (aal)

Peak intensity: -6.4318

# voxels structure

699 --TOTAL # VOXELS--

550 Left Cerebrum

549 Temporal Lobe

474 Superior Temporal Gyrus

381 Gray Matter

335 brodmann area 38

332 Temporal_Pole_Sup_L (aal)

285 Temporal_Pole_Mid_L (aal)

128 White Matter

72 Middle Temporal Gyrus

46 brodmann area 21

18 Temporal_Inf_L (aal)

10 Temporal_Mid_L (aal)

----------------------

Cluster 9

Number of voxels: 100

Peak MNI coordinate: -54 -7.5 -37.5

Peak MNI coordinate region: // Left Cerebrum // Temporal Lobe // Inferior Temporal Gyrus // Gray Matter // brodmann area 20 // Temporal_Inf_L (aal)

Peak intensity: -4.7406

# voxels structure

100 --TOTAL # VOXELS--

98 Temporal_Inf_L (aal)

86 Left Cerebrum

86 Temporal Lobe

75 Inferior Temporal Gyrus

52 brodmann area 20

52 Gray Matter

32 White Matter

10 Fusiform Gyrus

----------------------

Cluster 10

Number of voxels: 32

Peak MNI coordinate: -28.5 -7.5 -39

Peak MNI coordinate region: // Left Cerebrum // Limbic Lobe // Uncus // undefined // undefined // Fusiform_L (aal)

Peak intensity: -3.7131

# voxels structure

32 --TOTAL # VOXELS--

32 Fusiform_L (aal)

28 Limbic Lobe

28 Uncus

28 Left Cerebrum

14 Gray Matter

10 White Matter

5 brodmann area 28

5 brodmann area 20

4 brodmann area 36

----------------------

Cluster 11

Number of voxels: 14

Peak MNI coordinate: 18 -87 -31.5

Peak MNI coordinate region: // Right Cerebellum // Cerebellum Posterior Lobe // Uvula // undefined // undefined // Cerebelum_Crus2_R (aal)

Peak intensity: -4.3045

# voxels structure

14 --TOTAL # VOXELS--

14 Cerebelum_Crus2_R (aal)

11 Right Cerebellum

11 Cerebellum Posterior Lobe

9 Uvula

2 Declive

----------------------

Cluster 12

Number of voxels: 30

Peak MNI coordinate: 30 -21 -31.5

Peak MNI coordinate region: // Right Cerebrum // Limbic Lobe // Parahippocampa Gyrus // Gray Matter // brodmann area 36 // undefined

Peak intensity: -4.4027

# voxels structure

30 --TOTAL # VOXELS--

21 Parahippocampa Gyrus

21 Right Cerebrum

21 Limbic Lobe

14 Fusiform_R (aal)

9 Gray Matter

8 brodmann area 36

8 White Matter

7 ParaHippocampal_R (aal)

1 Cerebelum_4_5_R (aal)

1 brodmann area 35

----------------------

Cluster 13

Number of voxels: 104

Peak MNI coordinate: -63 -6 -25.5

Peak MNI coordinate region: // undefined // undefined // undefined // undefined // undefined // Temporal_Mid_L (aal)

Peak intensity: -4.626

# voxels structure

104 --TOTAL # VOXELS--

95 Temporal_Mid_L (aal)

81 Left Cerebrum

81 Temporal Lobe

50 Middle Temporal Gyrus

38 Gray Matter

37 brodmann area 21

31 Inferior Temporal Gyrus

12 White Matter

6 Temporal_Inf_L (aal)

1 Temporal_Pole_Mid_L (aal)

1 brodmann area 20

----------------------

Cluster 14

Number of voxels: 325

Peak MNI coordinate: 24 -4.5 -21

Peak MNI coordinate region: // Right Cerebrum // Limbic Lobe // Parahippocampa Gyrus // Gray Matter // Amygdala // Hippocampus_R (aal)

Peak intensity: -4.8044

# voxels structure

325 --TOTAL # VOXELS--

325 Right Cerebrum

304 Limbic Lobe

236 Parahippocampa Gyrus

228 Gray Matter

200 Amygdala

162 Hippocampus_R (aal)

88 Amygdala_R (aal)

86 White Matter

68 Uncus

66 ParaHippocampal_R (aal)

21 Sub-lobar

15 brodmann area 34

13 Hippocampus

11 Lateral Ventricle

11 Cerebro-Spinal Fluid

6 Temporal_Pole_Sup_R (aal)

5 Extra-Nuclear

----------------------

Cluster 15

Number of voxels: 230

Peak MNI coordinate: -37.5 18 -18

Peak MNI coordinate region: // Left Cerebrum // Frontal Lobe // Inferior Frontal Gyrus // Gray Matter // brodmann area 47 // Temporal_Pole_Sup_L (aal)

Peak intensity: -6.3183

# voxels structure

230 --TOTAL # VOXELS--

151 Left Cerebrum

143 Frontal_Inf_Orb_L (aal)

128 Frontal Lobe

124 Inferior Frontal Gyrus

93 Gray Matter

92 brodmann area 47

46 Temporal_Pole_Sup_L (aal)

21 Temporal Lobe

20 White Matter

18 Superior Temporal Gyrus

14 Olfactory_L (aal)

12 Insula_L (aal)

4 Subcallosal Gyrus

2 Frontal_Sup_Orb_L (aal)

1 brodmann area 38

----------------------

Cluster 16

Number of voxels: 230

Peak MNI coordinate: -24 -3 -22.5

Peak MNI coordinate region: // Left Cerebrum // Limbic Lobe // Parahippocampa Gyrus // Gray Matter // Amygdala // Amygdala_L (aal)

Peak intensity: -4.6602

# voxels structure

230 --TOTAL # VOXELS--

230 Left Cerebrum

173 Limbic Lobe

153 Parahippocampa Gyrus

150 Gray Matter

146 Amygdala

113 Amygdala_L (aal)

105 Hippocampus_L (aal)

56 Sub-lobar

40 Lateral Ventricle

40 White Matter

40 Cerebro-Spinal Fluid

20 Uncus

9 Extra-Nuclear

3 brodmann area 34

2 ParaHippocampal_L (aal)

2 Temporal_Pole_Sup_L (aal)

1 Temporal Lobe

1 Sub-Gyral

1 Hippocampus

----------------------

Cluster 17

Number of voxels: 159

Peak MNI coordinate: 21 13.5 -25.5

Peak MNI coordinate region: // Right Cerebrum // Frontal Lobe // Inferior Frontal Gyrus // undefined // undefined // Frontal_Inf_Orb_R (aal)

Peak intensity: -5.6739

# voxels structure

159 --TOTAL # VOXELS--

119 Right Cerebrum

115 Frontal Lobe

109 Inferior Frontal Gyrus

106 Frontal_Inf_Orb_R (aal)

50 brodmann area 47

50 Gray Matter

49 White Matter

26 Insula_R (aal)

6 Frontal_Sup_Orb_R (aal)

6 Rectal Gyrus

4 Temporal Lobe

4 Superior Temporal Gyrus

3 ParaHippocampal_R (aal)

1 Olfactory_R (aal)

----------------------

Cluster 18

Number of voxels: 106

Peak MNI coordinate: -22.5 10.5 0

Peak MNI coordinate region: // Left Cerebrum // Sub-lobar // Lentiform Nucleus // Gray Matter // Putamen // Putamen_L (aal)

Peak intensity: -4.2637

# voxels structure

106 --TOTAL # VOXELS--

106 Left Cerebrum

106 Putamen_L (aal)

106 Sub-lobar

94 Putamen

94 Gray Matter

94 Lentiform Nucleus

12 Extra-Nuclear

12 White Matter

----------------------

Cluster 19

Number of voxels: 11

Peak MNI coordinate: 10.5 -70.5 -4.5

Peak MNI coordinate region: // Right Cerebrum // Occipital Lobe // Lingual Gyrus // White Matter // undefined // Lingual_R (aal)

Peak intensity: -4.6633

# voxels structure

11 --TOTAL # VOXELS--

11 Lingual_R (aal)

11 Occipital Lobe

11 Right Cerebrum

10 Lingual Gyrus

7 Gray Matter

5 brodmann area 18

3 White Matter

----------------------

Cluster 20

Number of voxels: 10

Peak MNI coordinate: -40.5 -6 -6

Peak MNI coordinate region: // Left Cerebrum // Sub-lobar // Insula // White Matter // undefined // Insula_L (aal)

Peak intensity: -3.8204

# voxels structure

10 --TOTAL # VOXELS--

10 Insula_L (aal)

10 Left Cerebrum

10 White Matter

6 Insula

6 Sub-lobar

4 Sub-Gyral

4 Temporal Lobe

----------------------

Cluster 21

Number of voxels: 38

Peak MNI coordinate: 18 15 -1.5

Peak MNI coordinate region: // Right Cerebrum // Sub-lobar // Lentiform Nucleus // Gray Matter // Putamen // Putamen_R (aal)

Peak intensity: -4.0178

# voxels structure

38 --TOTAL # VOXELS--

38 Putamen_R (aal)

38 Right Cerebrum

38 Sub-lobar

35 Gray Matter

35 Lentiform Nucleus

35 Putamen

3 Extra-Nuclear

3 White Matter

----------------------

Cluster 22

Number of voxels: 81

Peak MNI coordinate: -7.5 -55.5 0

Peak MNI coordinate region: // Left Cerebrum // Occipital Lobe // undefined // undefined // undefined // Lingual_L (aal)

Peak intensity: -5.3102

# voxels structure

81 --TOTAL # VOXELS--

69 Lingual_L (aal)

60 Left Cerebrum

52 Occipital Lobe

32 Lingual Gyrus

24 Gray Matter

11 brodmann area 18

11 Inter-Hemispheric

10 Cerebellum Anterior Lobe

10 Culmen

10 Left Cerebellum

8 Limbic Lobe

8 Posterior Cingulate

7 brodmann area 19

7 Calcarine_L (aal)

6 brodmann area 30

5 Cuneus

2 White Matter

----------------------

Cluster 23

Number of voxels: 105

Peak MNI coordinate: 13.5 24 0

Peak MNI coordinate region: // Right Cerebrum // Sub-lobar // Extra-Nuclear // White Matter // undefined // Caudate_R (aal)

Peak intensity: -4.581

# voxels structure

105 --TOTAL # VOXELS--

105 Right Cerebrum

101 Caudate_R (aal)

74 White Matter

74 Sub-lobar

43 Extra-Nuclear

31 Caudate

31 Caudate Head

30 Gray Matter

25 Sub-Gyral

25 Frontal Lobe

6 Limbic Lobe

6 Anterior Cingulate

1 Putamen_R (aal)

1 Cerebro-Spinal Fluid

----------------------

Cluster 24

Number of voxels: 24

Peak MNI coordinate: -3 -21 1.5

Peak MNI coordinate region: // Left Cerebrum // Sub-lobar // Thalamus // Gray Matter // undefined // Thalamus_L (aal)

Peak intensity: -5.7781

# voxels structure

24 --TOTAL # VOXELS--

24 Left Cerebrum

24 Sub-lobar

24 Thalamus_L (aal)

19 Thalamus

19 Gray Matter

5 Extra-Nuclear

5 White Matter

5 Medial Dorsal Nucleus

----------------------

Cluster 25

Number of voxels: 36

Peak MNI coordinate: -15 -33 7.5

Peak MNI coordinate region: // Left Cerebrum // Sub-lobar // Thalamus // Gray Matter // Pulvinar // Hippocampus_L (aal)

Peak intensity: -4.0499

# voxels structure

36 --TOTAL # VOXELS--

36 Left Cerebrum

36 Sub-lobar

28 Gray Matter

28 Thalamus

26 Pulvinar

26 Thalamus_L (aal)

8 White Matter

8 Extra-Nuclear

3 Hippocampus_L (aal)

----------------------

Cluster 26

Number of voxels: 35

Peak MNI coordinate: 13.5 -31.5 9

Peak MNI coordinate region: // Right Cerebrum // Sub-lobar // Thalamus // Gray Matter // Pulvinar // Hippocampus_R (aal)

Peak intensity: -4.4244

# voxels structure

35 --TOTAL # VOXELS--

35 Right Cerebrum

35 Sub-lobar

31 Gray Matter

31 Thalamus

30 Pulvinar

26 Thalamus_R (aal)

4 Extra-Nuclear

4 White Matter

4 Hippocampus_R (aal)

----------------------

Cluster 27

Number of voxels: 453

Peak MNI coordinate: 19.5 -61.5 18

Peak MNI coordinate region: // Right Cerebrum // Temporal Lobe // Sub-Gyral // White Matter // undefined // Calcarine_R (aal)

Peak intensity: -6.6593

# voxels structure

453 --TOTAL # VOXELS--

453 Right Cerebrum

344 White Matter

171 Cuneus_R (aal)

167 Sub-Gyral

163 Calcarine_R (aal)

156 Precuneus

136 Temporal Lobe

130 Occipital Lobe

100 Gray Matter

98 Precuneus_R (aal)

89 Parietal Lobe

83 Posterior Cingulate

83 Limbic Lobe

56 brodmann area 31

32 Cuneus

26 brodmann area 7

15 Sub-lobar

15 Extra-Nuclear

9 brodmann area 18

5 brodmann area 30

5 Lingual_R (aal)

4 brodmann area 23

----------------------

Cluster 28

Number of voxels: 17

Peak MNI coordinate: 3 -75 15

Peak MNI coordinate region: // Right Cerebrum // Occipital Lobe // Cuneus // Gray Matter // brodmann area 18 // Calcarine_R (aal)

Peak intensity: -4.2719

# voxels structure

17 --TOTAL # VOXELS--

17 Cuneus

17 Occipital Lobe

17 Right Cerebrum

15 Gray Matter

15 brodmann area 18

9 Calcarine_R (aal)

8 Calcarine_L (aal)

----------------------

Cluster 29

Number of voxels: 266

Peak MNI coordinate: -16.5 -64.5 16.5

Peak MNI coordinate region: // Left Cerebrum // Occipital Lobe // Sub-Gyral // White Matter // undefined // Calcarine_L (aal)

Peak intensity: -6.9065

# voxels structure

266 --TOTAL # VOXELS--

266 Left Cerebrum

215 White Matter

187 Precuneus

135 Cuneus_L (aal)

113 Occipital Lobe

101 Parietal Lobe

82 Calcarine_L (aal)

58 Sub-Gyral

48 Gray Matter

41 brodmann area 31

37 Temporal Lobe

26 Occipital_Sup_L (aal)

13 Limbic Lobe

13 Posterior Cingulate

9 Precuneus_L (aal)

6 Cuneus

4 brodmann area 7

2 Extra-Nuclear

2 Sub-lobar

2 brodmann area 18

1 brodmann area 23

----------------------

Cluster 30

Number of voxels: 12

Peak MNI coordinate: -63 -21 25.5

Peak MNI coordinate region: // Left Cerebrum // Parietal Lobe // Postcentral Gyrus // White Matter // undefined // SupraMarginal_L (aal)

Peak intensity: -4.0515

# voxels structure

12 --TOTAL # VOXELS--

12 Left Cerebrum

12 Parietal Lobe

12 Postcentral Gyrus

7 SupraMarginal_L (aal)

6 Gray Matter

6 White Matter

5 Postcentral_L (aal)

4 brodmann area 2

2 brodmann area 40

----------------------

Cluster 31

Number of voxels: 47

Peak MNI coordinate: -13.5 -76.5 22.5

Peak MNI coordinate region: // Left Cerebrum // Occipital Lobe // Cuneus // Gray Matter // brodmann area 18 // Occipital_Sup_L (aal)

Peak intensity: -5.0823

# voxels structure

47 --TOTAL # VOXELS--

47 Left Cerebrum

38 Cuneus_L (aal)

35 Occipital Lobe

33 Gray Matter

26 Precuneus

21 Cuneus

19 brodmann area 7

12 Parietal Lobe

10 brodmann area 31

9 Occipital_Sup_L (aal)

4 brodmann area 18

3 White Matter

----------------------

Cluster 32

Number of voxels: 14

Peak MNI coordinate: 4.5 13.5 49.5

Peak MNI coordinate region: // Right Cerebrum // Frontal Lobe // Medial Frontal Gyrus // Gray Matter // brodmann area 6 // Supp_Motor_Area_R (aal)

Peak intensity: -3.8656

# voxels structure

14 --TOTAL # VOXELS--

14 Right Cerebrum

13 Supp_Motor_Area_R (aal)

11 Gray Matter

8 Cingulate Gyrus

8 Limbic Lobe

8 brodmann area 32

6 Medial Frontal Gyrus

6 Frontal Lobe

3 brodmann area 6

1 White Matter

1 Cingulum_Mid_R (aal)

----------------------

Cluster 33

Number of voxels: 12

Peak MNI coordinate: -15 -79.5 48

Peak MNI coordinate region: // undefined // undefined // undefined // undefined // undefined // Parietal_Sup_L (aal)

Peak intensity: -3.6665

# voxels structure

12 --TOTAL # VOXELS--

10 Parietal_Sup_L (aal)

8 Left Cerebrum

8 Precuneus

8 Parietal Lobe

4 White Matter

4 brodmann area 7

4 Gray Matter

2 Precuneus_L (aal)

----------------------

Cluster 34

Number of voxels: 11

Peak MNI coordinate: 31.5 36 48

Peak MNI coordinate region: // Right Cerebrum // Frontal Lobe // Middle Frontal Gyrus // Gray Matter // brodmann area 8 // Frontal_Mid_R (aal)

Peak intensity: -3.9276

# voxels structure

11 --TOTAL # VOXELS--

11 Frontal Lobe

11 Frontal_Mid_R (aal)

11 Middle Frontal Gyrus

11 Right Cerebrum

9 Gray Matter

9 brodmann area 8

2 White Matter

----------------------

Cluster 35

Number of voxels: 13

Peak MNI coordinate: 21 -75 51

Peak MNI coordinate region: // Right Cerebrum // Parietal Lobe // Precuneus // Gray Matter // brodmann area 7 // Parietal_Sup_R (aal)

Peak intensity: -3.9717

# voxels structure

13 --TOTAL # VOXELS--

13 Parietal Lobe

13 Parietal_Sup_R (aal)

13 Precuneus

13 Right Cerebrum

12 Gray Matter

12 brodmann area 7

1 White Matter

----------------------

Cluster 36

Number of voxels: 62

Peak MNI coordinate: 4.5 -22.5 58.5

Peak MNI coordinate region: // Right Cerebrum // Frontal Lobe // Medial Frontal Gyrus // Gray Matter // brodmann area 6 // Supp_Motor_Area_R (aal)

Peak intensity: -5.1081

# voxels structure

62 --TOTAL # VOXELS--

62 Frontal Lobe

62 Right Cerebrum

52 Medial Frontal Gyrus

52 Gray Matter

52 brodmann area 6

50 Supp_Motor_Area_R (aal)

11 Paracentral_Lobule_R (aal)

10 Paracentral Lobule

1 Cingulum_Mid_R (aal)

----------------------

Cluster 37

Number of voxels: 12

Peak MNI coordinate: 3 -3 55.5

Peak MNI coordinate region: // Right Cerebrum // Frontal Lobe // Medial Frontal Gyrus // Gray Matter // brodmann area 6 // Supp_Motor_Area_R (aal)

Peak intensity: -3.9354

# voxels structure

12 --TOTAL # VOXELS--

12 Frontal Lobe

12 Gray Matter

12 Medial Frontal Gyrus

12 Right Cerebrum

12 Supp_Motor_Area_R (aal)

12 brodmann area 6

----------------------

Cluster 38

Number of voxels: 10

Peak MNI coordinate: -3 -15 55.5

Peak MNI coordinate region: // Left Cerebrum // Frontal Lobe // Medial Frontal Gyrus // Gray Matter // brodmann area 6 // Supp_Motor_Area_L (aal)

Peak intensity: -3.7365

# voxels structure

10 --TOTAL # VOXELS--

10 Frontal Lobe

10 Gray Matter

10 Left Cerebrum

10 Medial Frontal Gyrus

10 Supp_Motor_Area_L (aal)

10 brodmann area 6

----------------------

Cluster 39

Number of voxels: 18

Peak MNI coordinate: 4.5 -9 64.5

Peak MNI coordinate region: // Right Cerebrum // Frontal Lobe // Medial Frontal Gyrus // Gray Matter // brodmann area 6 // Supp_Motor_Area_R (aal)

Peak intensity: -4.1997

# voxels structure

18 --TOTAL # VOXELS--

18 Frontal Lobe

18 Medial Frontal Gyrus

18 Right Cerebrum

18 Supp_Motor_Area_R (aal)

14 Gray Matter

14 brodmann area 6

1 White Matter

----------------------

Cluster 40

Number of voxels: 10

Peak MNI coordinate: 22.5 -10.5 61.5

Peak MNI coordinate region: // Right Cerebrum // Frontal Lobe // Sub-Gyral // White Matter // undefined // Frontal_Sup_R (aal)

Peak intensity: -4.9079

# voxels structure

10 --TOTAL # VOXELS--

10 Frontal Lobe

10 Frontal_Sup_R (aal)

10 Right Cerebrum

9 White Matter

8 Sub-Gyral

2 Middle Frontal Gyrus

1 brodmann area 6

1 Gray Matter

----------------------

Cluster 41

Number of voxels: 10

Peak MNI coordinate: -3 -1.5 61.5

Peak MNI coordinate region: // Left Cerebrum // Frontal Lobe // Medial Frontal Gyrus // Gray Matter // brodmann area 6 // Supp_Motor_Area_L (aal)

Peak intensity: -4.1622

# voxels structure

10 --TOTAL # VOXELS--

10 Frontal Lobe

10 Gray Matter

10 Left Cerebrum

10 Medial Frontal Gyrus

10 Supp_Motor_Area_L (aal)

10 brodmann area 6

>>

***Monolingual > Bilingual (positive direction relationship)***

Type: T

df: 324

Threshold

-- p value = 0.00029593

-- intensity = 3.4696

-- cluster size = 10

Number of clusters found: 29

----------------------

Cluster 1

Number of voxels: 15

Peak MNI coordinate: 39 -64.5 -54

Peak MNI coordinate region: // Right Cerebellum // Cerebellum Posterior Lobe // Inferior Semi-Lunar Lobule // undefined // undefined // Cerebelum_8_R (aal)

Peak intensity: 4.5318

# voxels structure

15 --TOTAL # VOXELS--

14 Cerebelum_7b_R (aal)

9 Inferior Semi-Lunar Lobule

9 Right Cerebellum

9 Cerebellum Posterior Lobe

1 Cerebelum_8_R (aal)

----------------------

Cluster 2

Number of voxels: 10

Peak MNI coordinate: 37.5 -49.5 -51

Peak MNI coordinate region: // Right Cerebellum // Cerebellum Posterior Lobe // Cerebellar Tonsil // undefined // undefined // Cerebelum_8_R (aal)

Peak intensity: 4.6345

# voxels structure

10 --TOTAL # VOXELS--

10 Cerebellum Posterior Lobe

10 Cerebelum_8_R (aal)

10 Right Cerebellum

10 Cerebellar Tonsil

----------------------

Cluster 3

Number of voxels: 42

Peak MNI coordinate: 46.5 -57 -42

Peak MNI coordinate region: // Right Cerebellum // Cerebellum Posterior Lobe // Cerebellar Tonsil // undefined // undefined // Cerebelum_Crus2_R (aal)

Peak intensity: 5.2604

# voxels structure

42 --TOTAL # VOXELS--

42 Right Cerebellum

42 Cerebellum Posterior Lobe

30 Cerebellar Tonsil

24 Cerebelum_Crus2_R (aal)

17 Cerebelum_7b_R (aal)

12 Inferior Semi-Lunar Lobule

1 Cerebelum_Crus1_R (aal)

----------------------

Cluster 4

Number of voxels: 45

Peak MNI coordinate: 21 -36 -43.5

Peak MNI coordinate region: // Right Cerebellum // Cerebellum Posterior Lobe // Cerebellar Tonsil // undefined // undefined // Cerebelum_10_R (aal)

Peak intensity: 6.258

# voxels structure

45 --TOTAL # VOXELS--

27 Cerebellum Posterior Lobe

27 Cerebelum_10_R (aal)

27 Right Cerebellum

27 Cerebellar Tonsil

3 Cerebelum_8_R (aal)

3 Cerebelum_9_R (aal)

----------------------

Cluster 5

Number of voxels: 22

Peak MNI coordinate: 40.5 -45 -33

Peak MNI coordinate region: // Right Cerebellum // Cerebellum Anterior Lobe // Culmen // undefined // undefined // Cerebelum_Crus1_R (aal)

Peak intensity: 4.3774

# voxels structure

22 --TOTAL # VOXELS--

22 Culmen

22 Right Cerebellum

22 Cerebellum Anterior Lobe

16 Cerebelum_Crus1_R (aal)

4 Cerebelum_6_R (aal)

2 Cerebelum_Crus2_R (aal)

----------------------

Cluster 6

Number of voxels: 30

Peak MNI coordinate: -30 -57 -27

Peak MNI coordinate region: // Left Cerebellum // Cerebellum Anterior Lobe // Culmen // undefined // undefined // Cerebelum_6_L (aal)

Peak intensity: 4.8759

# voxels structure

30 --TOTAL # VOXELS--

30 Cerebelum_6_L (aal)

30 Culmen

30 Left Cerebellum

30 Cerebellum Anterior Lobe

----------------------

Cluster 7

Number of voxels: 11

Peak MNI coordinate: 31.5 -48 -31.5

Peak MNI coordinate region: // Right Cerebellum // Cerebellum Anterior Lobe // Culmen // undefined // undefined // Cerebelum_6_R (aal)

Peak intensity: 5.5221

# voxels structure

11 --TOTAL # VOXELS--

11 Cerebelum_6_R (aal)

11 Culmen

11 Right Cerebellum

11 Cerebellum Anterior Lobe

----------------------

Cluster 8

Number of voxels: 20

Peak MNI coordinate: -12 45 -27

Peak MNI coordinate region: // Left Cerebrum // Frontal Lobe // Orbital Gyrus // Gray Matter // brodmann area 11 // undefined

Peak intensity: 5.3214

# voxels structure

20 --TOTAL # VOXELS--

20 Left Cerebrum

20 Frontal Lobe

14 brodmann area 11

14 Gray Matter

13 Orbital Gyrus

6 Rectal Gyrus

4 White Matter

1 Superior Frontal Gyrus

----------------------

Cluster 9

Number of voxels: 29

Peak MNI coordinate: 18 -37.5 -25.5

Peak MNI coordinate region: // Right Cerebellum // Cerebellum Anterior Lobe // Culmen // undefined // undefined // Cerebelum_4_5_R (aal)

Peak intensity: 5.102

# voxels structure

29 --TOTAL # VOXELS--

29 Culmen

29 Right Cerebellum

29 Cerebellum Anterior Lobe

20 Cerebelum_4_5_R (aal)

8 Cerebelum_3_R (aal)

----------------------

Cluster 10

Number of voxels: 32

Peak MNI coordinate: -13.5 -39 -22.5

Peak MNI coordinate region: // Left Cerebellum // Cerebellum Anterior Lobe // Culmen // undefined // undefined // Cerebelum_4_5_L (aal)

Peak intensity: 4.9398

# voxels structure

32 --TOTAL # VOXELS--

32 Cerebelum_4_5_L (aal)

32 Left Cerebellum

32 Cerebellum Anterior Lobe

31 Culmen

1 Cerebellar Lingual

----------------------

Cluster 11

Number of voxels: 16

Peak MNI coordinate: -9 42 -24

Peak MNI coordinate region: // Left Cerebrum // Frontal Lobe // Sub-Gyral // White Matter // undefined // Frontal_Sup_Orb_L (aal)

Peak intensity: 4.5842

# voxels structure

16 --TOTAL # VOXELS--

16 Frontal Lobe

16 Left Cerebrum

14 White Matter

11 Sub-Gyral

9 Frontal_Sup_Orb_L (aal)

7 Rectus_L (aal)

5 Rectal Gyrus

2 brodmann area 11

2 Gray Matter

----------------------

Cluster 12

Number of voxels: 21

Peak MNI coordinate: 18 -24 -19.5

Peak MNI coordinate region: // undefined // undefined // undefined // undefined // undefined // undefined

Peak intensity: 4.9484

# voxels structure

21 --TOTAL # VOXELS--

13 ParaHippocampal_R (aal)

11 Right Cerebrum

11 Limbic Lobe

9 Gray Matter

9 brodmann area 35

9 Parahippocampa Gyrus

----------------------

Cluster 13

Number of voxels: 14

Peak MNI coordinate: -6 42 -21

Peak MNI coordinate region: // Left Cerebrum // Frontal Lobe // Orbital Gyrus // White Matter // undefined // Rectus_L (aal)

Peak intensity: 4.7617

# voxels structure

14 --TOTAL # VOXELS--

14 Frontal Lobe

14 Left Cerebrum

14 Rectus_L (aal)

11 White Matter

9 Orbital Gyrus

4 Sub-Gyral

3 brodmann area 11

3 Gray Matter

1 Rectal Gyrus

----------------------

Cluster 14

Number of voxels: 17

Peak MNI coordinate: -40.5 22.5 -13.5

Peak MNI coordinate region: // Left Cerebrum // Frontal Lobe // Inferior Frontal Gyrus // White Matter // undefined // Frontal_Inf_Orb_L (aal)

Peak intensity: 5.5994

# voxels structure

17 --TOTAL # VOXELS--

17 Frontal Lobe

17 Frontal_Inf_Orb_L (aal)

17 Inferior Frontal Gyrus

17 Left Cerebrum

15 White Matter

2 brodmann area 47

2 Gray Matter

----------------------

Cluster 15

Number of voxels: 20

Peak MNI coordinate: -21 -43.5 -6

Peak MNI coordinate region: // Left Cerebrum // Limbic Lobe // Parahippocampa Gyrus // White Matter // undefined // Lingual_L (aal)

Peak intensity: 4.6367

# voxels structure

20 --TOTAL # VOXELS--

19 Left Cerebrum

18 Parahippocampa Gyrus

18 Limbic Lobe

15 White Matter

9 Lingual_L (aal)

8 ParaHippocampal_L (aal)

3 Gray Matter

3 brodmann area 36

3 Fusiform_L (aal)

1 Sub-Gyral

1 Occipital Lobe

----------------------

Cluster 16

Number of voxels: 94

Peak MNI coordinate: 51 -10.5 0

Peak MNI coordinate region: // Right Cerebrum // Temporal Lobe // Superior Temporal Gyrus // White Matter // undefined // Temporal_Sup_R (aal)

Peak intensity: 5.0757

# voxels structure

94 --TOTAL # VOXELS--

94 Right Cerebrum

65 Temporal_Sup_R (aal)

54 Temporal Lobe

52 Superior Temporal Gyrus

50 Gray Matter

40 Sub-lobar

37 Insula

32 White Matter

26 brodmann area 22

23 brodmann area 13

10 Heschl_R (aal)

4 Insula_R (aal)

4 Sub-Gyral

1 Claustrum

----------------------

Cluster 17

Number of voxels: 13

Peak MNI coordinate: 4.5 -7.5 6

Peak MNI coordinate region: // Right Cerebrum // Sub-lobar // Thalamus // Gray Matter // undefined // Thalamus_R (aal)

Peak intensity: 4.9465

# voxels structure

13 --TOTAL # VOXELS--

13 Gray Matter

13 Right Cerebrum

13 Sub-lobar

13 Thalamus

10 Thalamus_R (aal)

3 Ventral Anterior Nucleus

2 Medial Dorsal Nucleus

----------------------

Cluster 18

Number of voxels: 17

Peak MNI coordinate: -12 7.5 7.5

Peak MNI coordinate region: // Left Cerebrum // Sub-lobar // Caudate // Gray Matter // Caudate Body // Caudate_L (aal)

Peak intensity: 4.7541

# voxels structure

17 --TOTAL # VOXELS--

17 Left Cerebrum

17 Sub-lobar

14 Caudate_L (aal)

10 White Matter

10 Extra-Nuclear

7 Caudate

7 Gray Matter

4 Caudate Head

3 Caudate Body

----------------------

Cluster 19

Number of voxels: 19

Peak MNI coordinate: 16.5 10.5 9

Peak MNI coordinate region: // Right Cerebrum // Sub-lobar // Extra-Nuclear // White Matter // undefined // Caudate_R (aal)

Peak intensity: 4.3738

# voxels structure

19 --TOTAL # VOXELS--

19 Sub-lobar

19 Right Cerebrum

16 White Matter

16 Extra-Nuclear

14 Caudate_R (aal)

3 Caudate Body

3 Gray Matter

3 Caudate

----------------------

Cluster 20

Number of voxels: 10

Peak MNI coordinate: 19.5 -22.5 15

Peak MNI coordinate region: // Right Cerebrum // Sub-lobar // Thalamus // Gray Matter // Lateral Posterior Nucleus // Thalamus_R (aal)

Peak intensity: 4.4232

# voxels structure

10 --TOTAL # VOXELS--

10 Right Cerebrum

10 Sub-lobar

7 Thalamus

7 Thalamus_R (aal)

7 Gray Matter

3 White Matter

3 Extra-Nuclear

2 Pulvinar

1 Lateral Posterior Nucleus

----------------------

Cluster 21

Number of voxels: 12

Peak MNI coordinate: 25.5 54 13.5

Peak MNI coordinate region: // Right Cerebrum // Frontal Lobe // Middle Frontal Gyrus // White Matter // undefined // Frontal_Sup_R (aal)

Peak intensity: 4.6179

# voxels structure

12 --TOTAL # VOXELS--

12 Frontal Lobe

12 Frontal_Sup_R (aal)

12 Right Cerebrum

12 White Matter

7 Middle Frontal Gyrus

5 Superior Frontal Gyrus

----------------------

Cluster 22

Number of voxels: 21

Peak MNI coordinate: 7.5 -9 15

Peak MNI coordinate region: // Right Cerebrum // Sub-lobar // Thalamus // Gray Matter // undefined // Thalamus_R (aal)

Peak intensity: 4.879

# voxels structure

21 --TOTAL # VOXELS--

21 Sub-lobar

21 Right Cerebrum

12 Thalamus_R (aal)

10 White Matter

10 Extra-Nuclear

6 Thalamus

6 Gray Matter

5 Cerebro-Spinal Fluid

5 Lateral Ventricle

1 Medial Dorsal Nucleus

1 Ventral Anterior Nucleus

----------------------

Cluster 23

Number of voxels: 11

Peak MNI coordinate: 18 3 16.5

Peak MNI coordinate region: // Right Cerebrum // Sub-lobar // Extra-Nuclear // White Matter // undefined // Caudate_R (aal)

Peak intensity: 4.8819

# voxels structure

11 --TOTAL # VOXELS--

11 Caudate_R (aal)

11 Extra-Nuclear

11 Right Cerebrum

11 Sub-lobar

11 White Matter

----------------------

Cluster 24

Number of voxels: 15

Peak MNI coordinate: -12 -16.5 19.5

Peak MNI coordinate region: // Left Cerebrum // Sub-lobar // Lateral Ventricle // Cerebro-Spinal Fluid // undefined // undefined

Peak intensity: 4.8951

# voxels structure

15 --TOTAL # VOXELS--

15 Left Cerebrum

15 Sub-lobar

11 Gray Matter

11 Thalamus

11 Thalamus_L (aal)

6 Ventral Anterior Nucleus

4 Cerebro-Spinal Fluid

4 Ventral Lateral Nucleus

4 Lateral Ventricle

----------------------

Cluster 25

Number of voxels: 19

Peak MNI coordinate: -18 9 21

Peak MNI coordinate region: // Left Cerebrum // Sub-lobar // Extra-Nuclear // White Matter // undefined // Caudate_L (aal)

Peak intensity: 4.779

# voxels structure

19 --TOTAL # VOXELS--

19 Left Cerebrum

19 Sub-lobar

19 White Matter

19 Extra-Nuclear

17 Caudate_L (aal)

----------------------

Cluster 26

Number of voxels: 12

Peak MNI coordinate: -45 31.5 27

Peak MNI coordinate region: // Left Cerebrum // Frontal Lobe // Middle Frontal Gyrus // White Matter // undefined // Frontal_Mid_L (aal)

Peak intensity: 4.2232

# voxels structure

12 --TOTAL # VOXELS--

12 Frontal Lobe

12 Left Cerebrum

12 Middle Frontal Gyrus

12 White Matter

6 Frontal_Inf_Tri_L (aal)

6 Frontal_Mid_L (aal)

----------------------

Cluster 27

Number of voxels: 18

Peak MNI coordinate: -6 51 30

Peak MNI coordinate region: // Left Cerebrum // Frontal Lobe // Superior Frontal Gyrus // Gray Matter // brodmann area 9 // Frontal_Sup_Medial_L (aal)

Peak intensity: 5.5981

# voxels structure

18 --TOTAL # VOXELS--

18 Frontal Lobe

18 Frontal_Sup_Medial_L (aal)

18 Left Cerebrum

16 Superior Frontal Gyrus

14 Gray Matter

14 brodmann area 9

4 White Matter

2 Medial Frontal Gyrus

----------------------

Cluster 28

Number of voxels: 12

Peak MNI coordinate: 7.5 49.5 34.5

Peak MNI coordinate region: // Right Cerebrum // Frontal Lobe // Superior Frontal Gyrus // Gray Matter // brodmann area 9 // Frontal_Sup_Medial_R (aal)

Peak intensity: 4.0678

# voxels structure

12 --TOTAL # VOXELS--

12 Frontal Lobe

12 Frontal_Sup_Medial_R (aal)

12 Right Cerebrum

9 Superior Frontal Gyrus

6 brodmann area 9

6 Gray Matter

5 White Matter

3 Medial Frontal Gyrus

----------------------

Cluster 29

Number of voxels: 18

Peak MNI coordinate: -6 33 52.5

Peak MNI coordinate region: // Left Cerebrum // Frontal Lobe // Superior Frontal Gyrus // White Matter // undefined // Frontal_Sup_Medial_L (aal)

Peak intensity: 4.8327

# voxels structure

18 --TOTAL # VOXELS--

18 Frontal Lobe

18 Frontal_Sup_Medial_L (aal)

18 Left Cerebrum

17 Superior Frontal Gyrus

11 White Matter

7 brodmann area 8

7 Gray Matter

1 Medial Frontal Gyrus

>>

**D.2 ANCOVA: Monolingual vs. bilingual contrast, controlling English proficiency**

*Contrast: Monolingual vs. bilingual. “Peak intensity” indicates *t* value at cluster peak, where positive/negative sign indicates direction of relationship (positive: Monolingual > Bilingual; negative: Monolingual < Bilingual). Results rendered using xjview toolbox.

***Monolingual < Bilingual (negative direction relationship)***

Type: T

df: 323

Threshold

-- p value = 0.00092601

-- intensity = 3.1389

-- cluster size = 10

Number of clusters found: 39

----------------------

Cluster 1

Number of voxels: 46

Peak MNI coordinate: 28.5 7.5 -46.5

Peak MNI coordinate region: // Right Cerebrum // Temporal Lobe // Superior Temporal Gyrus // Gray Matter // brodmann area 38 // Temporal_Inf_R (aal)

Peak intensity: -5.2815

# voxels structure

46 --TOTAL # VOXELS--

43 Right Cerebrum

33 Temporal Lobe

33 Superior Temporal Gyrus

30 Gray Matter

28 brodmann area 38

18 Fusiform_R (aal)

13 Temporal_Inf_R (aal)

13 White Matter

10 Limbic Lobe

8 Uncus

2 Inferior Temporal Gyrus

2 brodmann area 20

----------------------

Cluster 2

Number of voxels: 1837

Peak MNI coordinate: 51 15 -34.5

Peak MNI coordinate region: // undefined // undefined // undefined // undefined // undefined // Temporal_Pole_Mid_R (aal)

Peak intensity: -6.7963

# voxels structure

1837 --TOTAL # VOXELS--

1275 Temporal Lobe

1275 Right Cerebrum

739 Gray Matter

576 Middle Temporal Gyrus

543 Temporal_Inf_R (aal)

528 Temporal_Mid_R (aal)

495 Temporal_Pole_Mid_R (aal)

348 Superior Temporal Gyrus

325 brodmann area 21

278 brodmann area 38

277 Inferior Temporal Gyrus

273 White Matter

194 Temporal_Pole_Sup_R (aal)

136 brodmann area 20

58 Fusiform Gyrus

13 Fusiform_R (aal)

10 Temporal_Sup_R (aal)

4 Sub-Gyral

----------------------

Cluster 3

Number of voxels: 10

Peak MNI coordinate: 34.5 -6 -46.5

Peak MNI coordinate region: // Right Cerebrum // Temporal Lobe // Inferior Temporal Gyrus // Gray Matter // brodmann area 20 // Fusiform_R (aal)

Peak intensity: -3.7684

# voxels structure

10 --TOTAL # VOXELS--

3 Inferior Temporal Gyrus

3 Right Cerebrum

3 brodmann area 20

3 Gray Matter

2 Fusiform_R (aal)

2 Temporal Lobe

1 Frontal Lobe

----------------------

Cluster 4

Number of voxels: 12

Peak MNI coordinate: 43.5 -7.5 -45

Peak MNI coordinate region: // undefined // undefined // undefined // undefined // undefined // Temporal_Inf_R (aal)

Peak intensity: -4.0806

# voxels structure

12 --TOTAL # VOXELS--

12 Temporal_Inf_R (aal)

----------------------

Cluster 5

Number of voxels: 746

Peak MNI coordinate: -51 18 -16.5

Peak MNI coordinate region: // Left Cerebrum // Temporal Lobe // Superior Temporal Gyrus // Gray Matter // brodmann area 38 // Temporal_Pole_Sup_L (aal)

Peak intensity: -5.9639

# voxels structure

746 --TOTAL # VOXELS--

618 Temporal Lobe

618 Left Cerebrum

488 Superior Temporal Gyrus

430 Gray Matter

353 brodmann area 38

343 Temporal_Pole_Sup_L (aal)

293 Temporal_Pole_Mid_L (aal)

138 White Matter

126 Middle Temporal Gyrus

77 brodmann area 21

34 Temporal_Mid_L (aal)

27 Temporal_Inf_L (aal)

----------------------

Cluster 6

Number of voxels: 43

Peak MNI coordinate: 42 -13.5 -40.5

Peak MNI coordinate region: // undefined // undefined // undefined // undefined // undefined // Fusiform_R (aal)

Peak intensity: -4.3301

# voxels structure

43 --TOTAL # VOXELS--

36 Temporal Lobe

36 Right Cerebrum

26 Inferior Temporal Gyrus

22 Fusiform_R (aal)

19 Temporal_Inf_R (aal)

18 White Matter

18 brodmann area 20

18 Gray Matter

10 Fusiform Gyrus

----------------------

Cluster 7

Number of voxels: 76

Peak MNI coordinate: -52.5 -7.5 -39

Peak MNI coordinate region: // Left Cerebrum // Temporal Lobe // Inferior Temporal Gyrus // Gray Matter // brodmann area 20 // Temporal_Inf_L (aal)

Peak intensity: -4.2558

# voxels structure

76 --TOTAL # VOXELS--

76 Temporal_Inf_L (aal)

72 Temporal Lobe

72 Left Cerebrum

63 Inferior Temporal Gyrus

42 brodmann area 20

42 Gray Matter

24 White Matter

7 Fusiform Gyrus

1 Middle Temporal Gyrus

----------------------

Cluster 8

Number of voxels: 73

Peak MNI coordinate: -28.5 -7.5 -39

Peak MNI coordinate region: // Left Cerebrum // Limbic Lobe // Uncus // undefined // undefined // Fusiform_L (aal)

Peak intensity: -4.2579

# voxels structure

73 --TOTAL # VOXELS--

72 Fusiform_L (aal)

60 Limbic Lobe

60 Left Cerebrum

58 Uncus

34 Gray Matter

22 White Matter

18 brodmann area 36

10 brodmann area 20

6 brodmann area 28

2 Parahippocampa Gyrus

1 ParaHippocampal_L (aal)

----------------------

Cluster 9

Number of voxels: 10

Peak MNI coordinate: 39 -21 -33

Peak MNI coordinate region: // undefined // undefined // undefined // undefined // undefined // Fusiform_R (aal)

Peak intensity: -4.0762

# voxels structure

10 --TOTAL # VOXELS--

10 Fusiform_R (aal)

7 Temporal Lobe

7 Right Cerebrum

4 Inferior Temporal Gyrus

4 brodmann area 20

4 Gray Matter

3 Fusiform Gyrus

1 White Matter

----------------------

Cluster 10

Number of voxels: 37

Peak MNI coordinate: 31.5 -24 -30

Peak MNI coordinate region: // undefined // undefined // undefined // undefined // undefined // Fusiform_R (aal)

Peak intensity: -4.1959

# voxels structure

37 --TOTAL # VOXELS--

27 Parahippocampa Gyrus

27 Right Cerebrum

27 Limbic Lobe

16 Fusiform_R (aal)

13 brodmann area 36

13 Gray Matter

10 White Matter

8 ParaHippocampal_R (aal)

----------------------

Cluster 11

Number of voxels: 15

Peak MNI coordinate: -52.5 -15 -22.5

Peak MNI coordinate region: // Left Cerebrum // Temporal Lobe // Sub-Gyral // White Matter // undefined // Temporal_Mid_L (aal)

Peak intensity: -4.1706

# voxels structure

15 --TOTAL # VOXELS--

15 Left Cerebrum

15 Temporal Lobe

14 White Matter

10 Temporal_Inf_L (aal)

8 Fusiform Gyrus

5 Inferior Temporal Gyrus

5 Temporal_Mid_L (aal)

1 Sub-Gyral

1 Gray Matter

1 brodmann area 21

1 Middle Temporal Gyrus

----------------------

Cluster 12

Number of voxels: 127

Peak MNI coordinate: -63 -6 -25.5

Peak MNI coordinate region: // undefined // undefined // undefined // undefined // undefined // Temporal_Mid_L (aal)

Peak intensity: -5.2189

# voxels structure

127 --TOTAL # VOXELS--

119 Temporal_Mid_L (aal)

109 Left Cerebrum

109 Temporal Lobe

68 Middle Temporal Gyrus

55 Gray Matter

54 brodmann area 21

41 Inferior Temporal Gyrus

23 White Matter

5 Temporal_Inf_L (aal)

1 brodmann area 20

----------------------

Cluster 13

Number of voxels: 116

Peak MNI coordinate: 18 0 -22.5

Peak MNI coordinate region: // Right Cerebrum // Limbic Lobe // Parahippocampa Gyrus // Gray Matter // brodmann area 34 // ParaHippocampal_R (aal)

Peak intensity: -4.0557

# voxels structure

116 --TOTAL # VOXELS--

116 Limbic Lobe

116 Right Cerebrum

88 Gray Matter

77 Parahippocampa Gyrus

77 Amygdala

52 ParaHippocampal_R (aal)

39 Uncus

35 Amygdala_R (aal)

28 White Matter

22 Hippocampus_R (aal)

11 brodmann area 34

6 Temporal_Pole_Sup_R (aal)

----------------------

Cluster 14

Number of voxels: 269

Peak MNI coordinate: -37.5 18 -18

Peak MNI coordinate region: // Left Cerebrum // Frontal Lobe // Inferior Frontal Gyrus // Gray Matter // brodmann area 47 // Temporal_Pole_Sup_L (aal)

Peak intensity: -5.91

# voxels structure

269 --TOTAL # VOXELS--

183 Left Cerebrum

160 Frontal_Inf_Orb_L (aal)

149 Frontal Lobe

139 Inferior Frontal Gyrus

100 Gray Matter

96 brodmann area 47

56 Temporal_Pole_Sup_L (aal)

28 White Matter

23 Olfactory_L (aal)

23 Temporal Lobe

20 Superior Temporal Gyrus

13 Insula_L (aal)

9 Limbic Lobe

7 Subcallosal Gyrus

6 Parahippocampa Gyrus

2 brodmann area 34

2 brodmann area 38

1 Frontal_Sup_Orb_L (aal)

1 ParaHippocampal_L (aal)

----------------------

Cluster 15

Number of voxels: 190

Peak MNI coordinate: 22.5 13.5 -25.5

Peak MNI coordinate region: // Right Cerebrum // Frontal Lobe // Inferior Frontal Gyrus // undefined // undefined // Frontal_Inf_Orb_R (aal)

Peak intensity: -5.8481

# voxels structure

190 --TOTAL # VOXELS--

138 Frontal_Inf_Orb_R (aal)

137 Right Cerebrum

132 Frontal Lobe

128 Inferior Frontal Gyrus

67 brodmann area 47

67 Gray Matter

50 White Matter

28 Insula_R (aal)

7 Frontal_Sup_Orb_R (aal)

5 Temporal Lobe

5 Superior Temporal Gyrus

4 Rectal Gyrus

3 ParaHippocampal_R (aal)

2 Olfactory_R (aal)

----------------------

Cluster 16

Number of voxels: 22

Peak MNI coordinate: -27 -3 -18

Peak MNI coordinate region: // Left Cerebrum // Limbic Lobe // Parahippocampa Gyrus // Gray Matter // Amygdala // Amygdala_L (aal)

Peak intensity: -3.83

# voxels structure

22 --TOTAL # VOXELS--

22 Left Cerebrum

22 Limbic Lobe

21 Amygdala

21 Gray Matter

21 Parahippocampa Gyrus

20 Amygdala_L (aal)

1 Uncus

1 White Matter

1 Hippocampus_L (aal)

----------------------

Cluster 17

Number of voxels: 56

Peak MNI coordinate: -25.5 -9 -10.5

Peak MNI coordinate region: // Left Cerebrum // Sub-lobar // Extra-Nuclear // White Matter // undefined // undefined

Peak intensity: -4.4275

# voxels structure

56 --TOTAL # VOXELS--

56 Left Cerebrum

43 Sub-lobar

31 Hippocampus_L (aal)

21 White Matter

18 Gray Matter

17 Cerebro-Spinal Fluid

17 Lateral Ventricle

16 Amygdala

14 Extra-Nuclear

13 Parahippocampa Gyrus

13 Limbic Lobe

6 Amygdala_L (aal)

2 Putamen

2 Lentiform Nucleus

1 Putamen_L (aal)

----------------------

Cluster 18

Number of voxels: 10

Peak MNI coordinate: -67.5 -25.5 -12

Peak MNI coordinate region: // Left Cerebrum // Temporal Lobe // Middle Temporal Gyrus // undefined // undefined // Temporal_Mid_L (aal)

Peak intensity: -4.609

# voxels structure

10 --TOTAL # VOXELS--

9 Middle Temporal Gyrus

9 Temporal Lobe

9 Temporal_Mid_L (aal)

9 Left Cerebrum

4 brodmann area 21

4 Gray Matter

----------------------

Cluster 19

Number of voxels: 14

Peak MNI coordinate: -25.5 1.5 -13.5

Peak MNI coordinate region: // Left Cerebrum // Limbic Lobe // Parahippocampa Gyrus // White Matter // undefined // undefined

Peak intensity: -3.8682

# voxels structure

14 --TOTAL # VOXELS--

14 Left Cerebrum

10 White Matter

7 Extra-Nuclear

7 Sub-lobar

4 Limbic Lobe

4 Subcallosal Gyrus

3 Olfactory_L (aal)

3 Parahippocampa Gyrus

3 Frontal Lobe

2 brodmann area 34

2 Gray Matter

1 Putamen_L (aal)

1 Amygdala_L (aal)

----------------------

Cluster 20

Number of voxels: 10

Peak MNI coordinate: 10.5 -70.5 -4.5

Peak MNI coordinate region: // Right Cerebrum // Occipital Lobe // Lingual Gyrus // White Matter // undefined // Lingual_R (aal)

Peak intensity: -4.3876

# voxels structure

10 --TOTAL # VOXELS--

10 Lingual_R (aal)

10 Occipital Lobe

10 Right Cerebrum

9 Lingual Gyrus

6 Gray Matter

4 brodmann area 18

3 White Matter

----------------------

Cluster 21

Number of voxels: 128

Peak MNI coordinate: 12 22.5 1.5

Peak MNI coordinate region: // Right Cerebrum // Sub-lobar // Caudate // Gray Matter // Caudate Head // Caudate_R (aal)

Peak intensity: -4.5361

# voxels structure

128 --TOTAL # VOXELS--

128 Right Cerebrum

119 Caudate_R (aal)

105 White Matter

81 Sub-lobar

58 Extra-Nuclear

36 Sub-Gyral

36 Frontal Lobe

22 Gray Matter

20 Caudate Head

20 Caudate

11 Limbic Lobe

11 Anterior Cingulate

5 Putamen_R (aal)

3 Lentiform Nucleus

3 Putamen

1 Cerebro-Spinal Fluid

----------------------

Cluster 22

Number of voxels: 81

Peak MNI coordinate: -7.5 -55.5 0

Peak MNI coordinate region: // Left Cerebrum // Occipital Lobe // undefined // undefined // undefined // Lingual_L (aal)

Peak intensity: -5.6082

# voxels structure

81 --TOTAL # VOXELS--

76 Lingual_L (aal)

58 Left Cerebrum

54 Occipital Lobe

37 Lingual Gyrus

28 Gray Matter

16 brodmann area 18

12 Culmen

12 Cerebellum Anterior Lobe

12 Left Cerebellum

11 Inter-Hemispheric

8 brodmann area 19

5 Cuneus

4 Limbic Lobe

4 Calcarine_L (aal)

4 Posterior Cingulate

3 brodmann area 30

2 White Matter

----------------------

Cluster 23

Number of voxels: 18

Peak MNI coordinate: -10.5 22.5 -1.5

Peak MNI coordinate region: // Left Cerebrum // Limbic Lobe // Anterior Cingulate // White Matter // undefined // Caudate_L (aal)

Peak intensity: -4.6089

# voxels structure

18 --TOTAL # VOXELS--

18 Left Cerebrum

16 Caudate_L (aal)

14 White Matter

9 Anterior Cingulate

9 Limbic Lobe

6 Sub-lobar

4 Gray Matter

3 Sub-Gyral

3 Caudate Head

3 Caudate

3 Frontal Lobe

2 Extra-Nuclear

2 Putamen_L (aal)

1 Putamen

1 Lentiform Nucleus

----------------------

Cluster 24

Number of voxels: 10

Peak MNI coordinate: -19.5 12 0

Peak MNI coordinate region: // Left Cerebrum // Sub-lobar // Lentiform Nucleus // Gray Matter // Putamen // Putamen_L (aal)

Peak intensity: -3.4316

# voxels structure

10 --TOTAL # VOXELS--

10 Gray Matter

10 Left Cerebrum

10 Lentiform Nucleus

10 Putamen

10 Putamen_L (aal)

10 Sub-lobar

----------------------

Cluster 25

Number of voxels: 30

Peak MNI coordinate: 13.5 -31.5 9

Peak MNI coordinate region: // Right Cerebrum // Sub-lobar // Thalamus // Gray Matter // Pulvinar // Hippocampus_R (aal)

Peak intensity: -4.3657

# voxels structure

30 --TOTAL # VOXELS--

30 Right Cerebrum

30 Sub-lobar

26 Gray Matter

26 Pulvinar

26 Thalamus

17 Thalamus_R (aal)

10 Hippocampus_R (aal)

4 White Matter

4 Extra-Nuclear

----------------------

Cluster 26

Number of voxels: 63

Peak MNI coordinate: -54 27 13.5

Peak MNI coordinate region: // Left Cerebrum // Frontal Lobe // Inferior Frontal Gyrus // Gray Matter // brodmann area 46 // Frontal_Inf_Tri_L (aal)

Peak intensity: -4.3866

# voxels structure

63 --TOTAL # VOXELS--

63 Frontal Lobe

63 Frontal_Inf_Tri_L (aal)

63 Inferior Frontal Gyrus

63 Left Cerebrum

44 White Matter

19 Gray Matter

14 brodmann area 45

5 brodmann area 46

----------------------

Cluster 27

Number of voxels: 53

Peak MNI coordinate: 12 -66 13.5

Peak MNI coordinate region: // Right Cerebrum // Limbic Lobe // Posterior Cingulate // White Matter // undefined // Calcarine_R (aal)

Peak intensity: -5.0123

# voxels structure

53 --TOTAL # VOXELS--

53 Right Cerebrum

49 Calcarine_R (aal)

46 Posterior Cingulate

46 Limbic Lobe

30 White Matter

19 Gray Matter

13 brodmann area 31

7 Occipital Lobe

4 Cuneus

4 Lingual_R (aal)

3 brodmann area 30

3 Precuneus

3 brodmann area 18

----------------------

Cluster 28

Number of voxels: 316

Peak MNI coordinate: 22.5 -61.5 18

Peak MNI coordinate region: // Right Cerebrum // Temporal Lobe // Sub-Gyral // White Matter // undefined // Calcarine_R (aal)

Peak intensity: -5.6115

# voxels structure

316 --TOTAL # VOXELS--

316 Right Cerebrum

254 White Matter

165 Cuneus_R (aal)

144 Precuneus

140 Sub-Gyral

115 Temporal Lobe

99 Occipital Lobe

87 Precuneus_R (aal)

80 Parietal Lobe

55 Gray Matter

50 Calcarine_R (aal)

32 brodmann area 31

21 brodmann area 7

18 Limbic Lobe

16 Posterior Cingulate

12 Cuneus

4 Sub-lobar

4 Extra-Nuclear

2 brodmann area 19

----------------------

Cluster 29

Number of voxels: 115

Peak MNI coordinate: -16.5 -64.5 16.5

Peak MNI coordinate region: // Left Cerebrum // Occipital Lobe // Sub-Gyral // White Matter // undefined // Calcarine_L (aal)

Peak intensity: -6.3922

# voxels structure

115 --TOTAL # VOXELS--

115 Left Cerebrum

97 White Matter

68 Precuneus

47 Occipital Lobe

45 Sub-Gyral

43 Cuneus_L (aal)

35 Parietal Lobe

34 Calcarine_L (aal)

32 Temporal Lobe

22 Occipital_Sup_L (aal)

15 Gray Matter

14 brodmann area 31

1 Posterior Cingulate

1 Limbic Lobe

1 brodmann area 18

1 Precuneus_L (aal)

1 Cuneus

----------------------

Cluster 30

Number of voxels: 13

Peak MNI coordinate: -6 -63 19.5

Peak MNI coordinate region: // Left Cerebrum // Occipital Lobe // Precuneus // White Matter // undefined // Calcarine_L (aal)

Peak intensity: -3.5125

# voxels structure

13 --TOTAL # VOXELS--

13 Left Cerebrum

12 Cuneus_L (aal)

12 Precuneus

9 Occipital Lobe

7 Gray Matter

6 White Matter

6 brodmann area 31

4 Parietal Lobe

1 Cuneus

1 brodmann area 7

1 Calcarine_L (aal)

----------------------

Cluster 31

Number of voxels: 32

Peak MNI coordinate: -13.5 -76.5 24

Peak MNI coordinate region: // Left Cerebrum // Occipital Lobe // Precuneus // Gray Matter // brodmann area 31 // Cuneus_L (aal)

Peak intensity: -4.8474

# voxels structure

32 --TOTAL # VOXELS--

32 Left Cerebrum

28 Occipital Lobe

22 Cuneus_L (aal)

22 Gray Matter

18 Precuneus

14 Cuneus

10 brodmann area 31

10 Occipital_Sup_L (aal)

9 brodmann area 7

4 Parietal Lobe

3 brodmann area 18

2 White Matter

----------------------

Cluster 32

Number of voxels: 20

Peak MNI coordinate: -52.5 13.5 25.5

Peak MNI coordinate region: // Left Cerebrum // Frontal Lobe // Inferior Frontal Gyrus // White Matter // undefined // Frontal_Inf_Tri_L (aal)

Peak intensity: -4.3891

# voxels structure

20 --TOTAL # VOXELS--

20 Frontal Lobe

20 Frontal_Inf_Tri_L (aal)

20 Inferior Frontal Gyrus

20 Left Cerebrum

16 White Matter

4 brodmann area 9

4 Gray Matter

----------------------

Cluster 33

Number of voxels: 10

Peak MNI coordinate: 21 -76.5 49.5

Peak MNI coordinate region: // Right Cerebrum // Parietal Lobe // Precuneus // Gray Matter // brodmann area 7 // Parietal_Sup_R (aal)

Peak intensity: -4.0114

# voxels structure

10 --TOTAL # VOXELS--

10 Gray Matter

10 Parietal Lobe

10 Parietal_Sup_R (aal)

10 Precuneus

10 Right Cerebrum

10 brodmann area 7

----------------------

Cluster 34

Number of voxels: 11

Peak MNI coordinate: 10.5 -75 51

Peak MNI coordinate region: // Right Cerebrum // Parietal Lobe // Precuneus // White Matter // undefined // Precuneus_R (aal)

Peak intensity: -4.1378

# voxels structure

11 --TOTAL # VOXELS--

11 Parietal Lobe

11 Precuneus

11 Right Cerebrum

6 Precuneus_R (aal)

6 White Matter

5 Parietal_Sup_R (aal)

5 brodmann area 7

5 Gray Matter

----------------------

Cluster 35

Number of voxels: 12

Peak MNI coordinate: 4.5 -27 54

Peak MNI coordinate region: // Right Cerebrum // Frontal Lobe // Paracentral Lobule // Gray Matter // brodmann area 6 // Supp_Motor_Area_R (aal)

Peak intensity: -4.2801

# voxels structure

12 --TOTAL # VOXELS--

12 Frontal Lobe

12 Right Cerebrum

10 Gray Matter

10 brodmann area 6

7 Supp_Motor_Area_R (aal)

7 Medial Frontal Gyrus

5 Paracentral Lobule

4 Paracentral_Lobule_R (aal)

1 Cingulum_Mid_R (aal)

----------------------

Cluster 36

Number of voxels: 16

Peak MNI coordinate: 3 -22.5 60

Peak MNI coordinate region: // Right Cerebrum // Frontal Lobe // Medial Frontal Gyrus // Gray Matter // brodmann area 6 // Supp_Motor_Area_R (aal)

Peak intensity: -4.0589

# voxels structure

16 --TOTAL # VOXELS--

16 Frontal Lobe

16 Gray Matter

16 Medial Frontal Gyrus

16 Right Cerebrum

16 Supp_Motor_Area_R (aal)

16 brodmann area 6

----------------------

Cluster 37

Number of voxels: 13

Peak MNI coordinate: -21 -9 63

Peak MNI coordinate region: // Left Cerebrum // Frontal Lobe // Middle Frontal Gyrus // White Matter // undefined // Frontal_Sup_L (aal)

Peak intensity: -4.6891

# voxels structure

13 --TOTAL # VOXELS--

13 Frontal Lobe

13 Frontal_Sup_L (aal)

13 Left Cerebrum

10 Middle Frontal Gyrus

7 White Matter

6 brodmann area 6

6 Gray Matter

3 Sub-Gyral

----------------------

Cluster 38

Number of voxels: 16

Peak MNI coordinate: -24 0 66

Peak MNI coordinate region: // Left Cerebrum // Frontal Lobe // Middle Frontal Gyrus // White Matter // undefined // Frontal_Sup_L (aal)

Peak intensity: -4.3821

# voxels structure

16 --TOTAL # VOXELS--

16 Frontal_Sup_L (aal)

15 Left Cerebrum

15 Frontal Lobe

8 Superior Frontal Gyrus

7 Middle Frontal Gyrus

6 White Matter

6 brodmann area 6

6 Gray Matter

----------------------

Cluster 39

Number of voxels: 13

Peak MNI coordinate: -4.5 -1.5 72

Peak MNI coordinate region: // Left Cerebrum // Frontal Lobe // Superior Frontal Gyrus // Gray Matter // brodmann area 6 // Supp_Motor_Area_L (aal)

Peak intensity: -3.6762

# voxels structure

13 --TOTAL # VOXELS--

13 Frontal Lobe

13 Left Cerebrum

13 Supp_Motor_Area_L (aal)

12 Superior Frontal Gyrus

8 Gray Matter

8 brodmann area 6

5 White Matter

1 Medial Frontal Gyrus

>>
