# Supplementary material for: Age of acquisition impacts the brain differently depending on neuroanatomical metric
Source: Hum Brain Mapp. 2019 Oct 10;41(2):484–502. doi: 10.1002/hbm.24817 (PMC7267963; doi:10.1002/hbm.24817)
Supplement: Supplementary file 5 — Data S1. Outline of proficiency measures included in each study [file HBM-41-484-s002.docx]

Supplemental Materials

*Outline of proficiency measures included in each study*

| Study name |  | Proficiency measure | | | |
| --- | --- | --- | --- | --- | --- |
|  |  | Picture vocabulary^1^ | Listening comprehension^1^ | Passage comprehension^1^ | Boston Naming Test^2^ |
| Cognitive Control |  | **Yes** | No | **Yes** | No |
| P task |  | **Yes** | **Yes** | No | No |
| Phonemic Learning |  | **Yes** | **Yes** | No | No |
| Phonological Interference |  | **Yes** | **Yes** | No | No |
| S task |  | **Yes** | **Yes** | No | No |
| Word Learning 1† |  | **Yes** | **Yes** | No | No |
| Word Learning 2 |  | No | No | No | **Yes** |
| Word Reading |  | **Yes** | **Yes** | No | **Yes** |

| Monolinguals took each subtest in English; Bilinguals took each indicated test in English and Spanish.  ^1^Woodcock et al., 2005  ^2^Kaplan et al., 1983 |
| --- |

† Word Learning 1 included only monolinguals.
